# Catalogue of multimorbidity mean based severity and associational prevalence rates between 199+ chronic conditions—A nationwide register-based population study

**DOI:** 10.1371/journal.pone.0273850

**Published:** 2022-09-14

**Authors:** Michael Falk Hvidberg, Anne Frølich, Sanne Lykke Lundstrøm, Nina Kamstrup-Larsen

**Affiliations:** 1 Innovation and Research Center for Multimorbidity, Slagelse Hospital, Slagelse, Denmark; 2 University of York, York, United Kingdom; 3 The Research Unit for General Practice and Section of General Practice, Department of Public Health, University of Copenhagen, København, Denmark; 4 Center for Clinical Research and Prevention, Bispebjerg and Frederiksberg Hospital, The Capital Region of Denmark, København, Denmark; Washington University in St Louis Department of Psychiatry, UNITED STATES

## Abstract

**Background:**

Real-world data on multimorbidity represents an important but underutilised source of evidence for the planning of healthcare services, including prevention, treatments, and health economic modelling.

**Aims:**

This study aimed to estimate means of multimorbidity and provide associated prevalence rates and frequencies between 199 x 199 chronic conditions and disease groups based on the total adult Danish population and sex, age, and educational attainment. Thus, this study provides an off-the-shelf catalogue for use in treatments and planning by clinicians, decision-makers and researchers.

**Methods:**

The study population contained all Danish residents above 16 years on 1 January 2013 (n = 4,555,439). The data was based on the linkage of six national registers covering hospital contacts, services in general practice, filled-in out-of-hospital prescriptions, and educational attainments. The health registers were used to identify the 199 chronic conditions based on the ICD-10 classification system.

**Results:**

The mean number of chronic conditions (NCC) was 2.2. The mean increased with age, women had a higher mean than men, and there was a social gradient with the mean increasing with lower educational attainment. The mean NCC varied from 3.3–9.8 among all conditions. Across disease groups, the highest mean NCC were found within disease group N (chronic renal failure, mean = 8.8), D (in situ and benign neoplasms; mean = 6.5), K (diseases of the digestive system; mean = 5.7), and H (diseases of the eye and the ear; mean = 5.6). The highest mean NCC among the 29 common diseases was heart failure, ischemic heart diseases, angina pectoris, stroke, and dementia, with a mean above 6.5. Several prevalent conditions like hypertension, arthritis, chronic lower respiratory diseases, depression, type 2 diabetes, and overweight transcended other conditions regarding the associated prevalence rates. As one of the most frequent, hypertensive diseases were highly associated with arthritis (50.4%), depression (37.4%), type 2 diabetes (75.4%), cancers (49.7%), and being overweight (39.7%)–meaning that 50.4% of people with arthritis, 37.4% of people with depression and so on also had hypertensive diseases. The largest differences in means between individuals with no educational attainment and individuals with high educational attainment were found within disease groups J (diseases of the respiratory system, ratio = 1.8), Q (congenital malformations, deformations, and chromosomal abnormalities, ratio = 1.7), and B (viral hepatitis and human immunodeficiency virus disease, ratio = 1.7).

**Conclusions:**

The current study provides a nationwide off-the-shelf catalogue of multimorbidity means and real-world associations estimates of 199+ chronic conditions for future clinical treatments and health care systems planning. The findings described are just one example of numerous results and underline that multimorbidity is highly prevalent in the adult Danish population and that it is a vital condition transcending all future medical treatment. The data offer essential information on the multimorbidity burden of disease in future differentiated treatments, healthcare planning, and economic, aetiological, and other research.

## Introduction

Patients with multimorbidity, frequently defined as the coexistence of two or more chronic conditions within the same individual [1, 2], have a lower health-related quality of life [3, 4], higher mortality rates [5], decreased functional competence [6], and make more use of healthcare resources [7, 8]. Numerous studies have identified the unequal distribution of diseases across socioeconomic groups [7, 9, 10]. Moreover, a growing proportion of the worldwide population lives with chronic disease and multimorbidity due to ageing populations, better living conditions, and improved healthcare technology [11, 12]. For example, a recent study identified that 54.3 per cent of the Danish adult population had at least two chronic conditions and that 87.6 per cent of citizens above the age of 75 had multimorbidity with an average of 5.3 chronic conditions [12]. In addition, the disease-related–and increasing costs [13–23]–have been estimated to account for up to 80 per cent of the total healthcare expenditures for chronic conditions and multimorbidity [24–27]. Consequently, the challenges of multimorbidity are already high; and are only expected to rise in the decades to come [7, 11, 12].

The high prevalence of multimorbidity is particularly challenging for governments worldwide due to healthcare treatment structures. Healthcare systems worldwide are set up mainly for treating patients with single diseases; thus, most disease guidelines in the health system focus on single diseases [28]. This is contrary to an integrated approach and may amplify the risk of iatrogenic harm, increased drug interaction effects, and undesirable deficiencies in treatments and coordination for patients with multimorbidity [29]. Thus, any efforts to design future healthcare organisations to accommodate the growing number of patients with multimorbidity require detailed epidemiological data on multimorbidity and disease patterns. Moreover, decision-makers need access to reliable, real-world evidence of treatment patterns to handle the growing cost of healthcare [30, 31]. Hence, real-world evidence of disease burden, prevalence, and correlational patterns are crucial for accurate estimates, cost of illness, and budget-impact analysis on novel health care technologies [32, 33].

Multimorbidity is, however, a multifaceted, entangled, challenging subject to analyse. The Charlson Comorbidity Index [34], or simply counting conditions, may not provide sufficient details to understand complex disease patterns. Hence, much literature has investigated disease patterns using complex statistical methods [35–42]. For example, one study by Larsen et al. (2017) identified 6–7 disease groups from 15 conditions using latent class analysis [11]. Nonetheless, this illustrates some statistical difficulties in sufficiently describing disease patterns, as a reduction from 15 conditions to 6–7 disease groups might be considered relatively small; and researchers would most likely be able to find the same patterns by using simple prevalence estimates. Statistical pattern reduction is also particularly problematic as different statistical methods provide different results, are challenging to interpret and use, and there is no consensus on which statistical methods to use [11]. Thus, although statistical pattern recognition methods are useful for broad pattern recognition, further methodological work is needed.

Furthermore, for health professionals, raw, real-life, non-statistically reduced estimates are useful to obtain all details of the disease population of interest for either health care planning or clinical treatment. Another related study has, however, reported the prevalence rates of disease combinations but used self-reported conditions and was limited to 17 conditions [42]. Including a limited number of chronic conditions or using self-reported conditions is a limitation of many disease studies [7, 11, 27, 42–50]. This provides a boundary for real-world estimates of the full disease burden experienced by patients. But also as, different study methodologies limit the comparability of diseases prevalence estimates needed for decision-makers and others; thus, researchers and authorities have recommended using a uniform study methodology in disease burden studies across conditions for decades [14, 15, 51–56].

The current study aimed to estimate basic, descriptive, nationally representative means of multimorbidity and associated prevalence rates and frequencies of 199 x 199 chronic conditions of the total adult Danish population according to sex, age, and educational attainment. As one measure of severity, the mean NCC will enable researchers, health professionals, health economists, and decision-makers to identify, access, and compare the disease burden of the 199 chronic conditions. The correlational prevalence estimates between the 199 x 199 conditions will give real-world, detailed, unbiased, self-report estimates of the concrete multimorbidity for each of the 199 chronic conditions used in treatments and health care planning. Thus, the study provides an off-the-shelf catalogue and a comparative overview of multimorbidity across 199 chronic conditions. To the best of the authors’ knowledge, the current study provides the most comprehensive descriptive estimates of multimorbidity means and correlational prevalence of chronic conditions based on an entire country’s population, a uniform, comparable methodology and an exceptionally high number of chronic conditions.

## Methods

### Study population

The nationwide study population included 4,555,439 Danish residents aged 16 years or older alive on 1 January 2013. The study population consisted of 49.2% men, and the mean age was 46.7 years. Forty-five per cent were between 16–44 years old, 46% were between 45–74 years old, and 9% were 75 years old or older.

### The registers

In Denmark, there is a long tradition of reporting diseases, treatments, medications, and contact with the healthcare system, in national health registers. The registers were originally intended for data collection by government officials in public administration at the individual level [57]. All registers have a unique civil registration number that enables individual linkage across registers by the distinct personal identification number assigned to every resident in Denmark [58].

In the current study, six registers were applied and linked from Statistics Denmark. The National Patient Register (NPR) [59], the Danish Psychiatric Central Research Register (PCRR) [60], the National Prescription Register (TNPR) [61], and the National Health Service Register (NHSR) [62] held information on ICD-10 diagnoses, medicine prescriptions, and services in general practice. Educational attainments were obtained from the Population’s Education Register (PER) [63] based on the International Standard Classification of Education (ISCED2011). Sex and age originated from the Danish Civil Registration System [64]. The utilised registers and characteristics are described elsewhere [12, 65, 66].

### Defining ‘chronic condition’

A ‘chronic condition’ was defined in line with former studies if the *‘…condition had lasted or was expected to last twelve or more months and resulted in functional limitations and/or the need for functional limitations and/or the need for ongoing medical care’* [12, 67–69]. Using the Delphi method, a medical expert panel decided which ICD-10 diagnosis out of around 22,000 ICD-10 codes to be considered ‘chronic’ from the above definition [65]. The experts grouped the chosen chronic ICD-10 diagnosis into 199 conditions, of which some conditions encompassed subgroups of ICD-10 diagnosis. Hence, some identified conditions contained multiple different conditions within interrelated disease groups. Consequently, all ICD-10 conditions considered chronic based on the definition was contained in pursuit of including the full-population burden of chronic conditions [12]. A detailed description of the definitions, distinct phases and methodology are provided elsewhere [15, 65, 66].

### The data register algorithms used to identify the chronic conditions

Since numerous chronic conditions last longer than the 12 months used in the definition but do not persist for a lifetime, the ‘severity of chronicity’ was categorised into four categories depending on how long the conditions were expected to last [65]:

1. **Category I:** Stationary to progressive chronic conditions (no time limit equals inclusion time going back from the time of interest for as long as valid data were available. In the current study, this starting point was defined by the introduction of the ICD-10 diagnosis coding in Denmark in 1994);
2. **Category II:** Stationary to diminishing chronic conditions (10 years from register inclusion time to the time of interest);
3. **Category III:** Diminishing chronic conditions (5 years from register inclusion time to the time of interest); and
4. **Category IV:** Borderline chronic conditions (2 years from register inclusion time to the time of interest).

Adapted with permission from Hvidberg et al. (2016, 2019) [12, 65].

This method was designed to handle a renowned challenge of register-research: if a disease is only identified once, for instance, 5, 10, or 30 years back in time from a specific date, is it then expected that the patient still suffers from the condition? Hence, the expert panel assigned all of the 199 chronic conditions into one of the four categories. The allocation into one of the four categories was based on a medical judgement on how long time the various ICD-10 diagnoses identified as ‘chronic’, with the best possible clinical conviction, would still have the disease from a time of interest. This systematic approach was employed as a proxy for disease severity. An algorithm based on the medical experts’ definitions identified ICD-10 codes and allocated each of the 199 chronic conditions into the four chronicity categories that were utilised for data collection. However, for 35 of the 199 chronic conditions, the medical experts did not expect the ICD-10 diagnosis to be representative alone. Thus, 35 algorithms were developed based on multiple registers comprising medicine, hospital treatments, and services in general practice [12, 65, 66]. Additional details of the 199 distinctive definitions, including the 35 diagnostic algorithms, the medical experts and the panel process, and the four categories’ assignment, are described earlier [65, 66].

### Statistical analysis

Means of chronic conditions and per cent prevalence were calculated for each of the 199 chronic conditions. Means were calculated as the sum of all subjects’ multimorbidity within the disease of interest, divided by the number of subjects within the disease group and elaborating variables of interest. We used the following elaborating variables: sex, age groups (16–44, 45–74, and 75+) and educational attainment (no education vs higher education). Per cent prevalence was calculated within diseases of elaborating variables as the number of subjects of the elaborating variable of interest, divided by the total subjects of the disease, multiplied by a hundred. Direct standardised means and prevalence estimates were presented and calculated based on the national proportion of sex and age on 1 January 2013, as referenced [70, 71] were applicable. Ratios as a measure of social disparity in multimorbidity were calculated by dividing the mean number of chronic conditions (NCCs) of individuals with no education by the means among individuals with high education attainment for all conditions. Standard deviations (SD) of means were provided.

All conditions were given ranks according to their NCCs, with one indicating the highest NCCs based on the unstandardised means. To provide the reader with an overview of the comprehensive material, 14 *disease groups* referring to the ICD-10 system and described in detail elsewhere [12] and 29 *common conditions* plus overweight are presented and commented on in the result section. The *common conditions* comprise the conditions measured in the National Population Health Surveys every fourth year [72], among others. “Overweight” is included due to its general importance, although not consistently considered a chronic condition in the literature.

Data management and analysis were done using SAS 9.4 from Statistics Denmark’s remote research servers.

### Compliance with ethical standards

Declaration and approval to conduct the study were obtained from the Danish Data Protection Agency and the Secretariat for Research Processing Records, Data and Development Support, Region Zealand (REG-142-2021). No informed consent was required. Statistics Denmark anonymized all register-data before the data were made available on their secured server.

## Results

**Fig 1:**
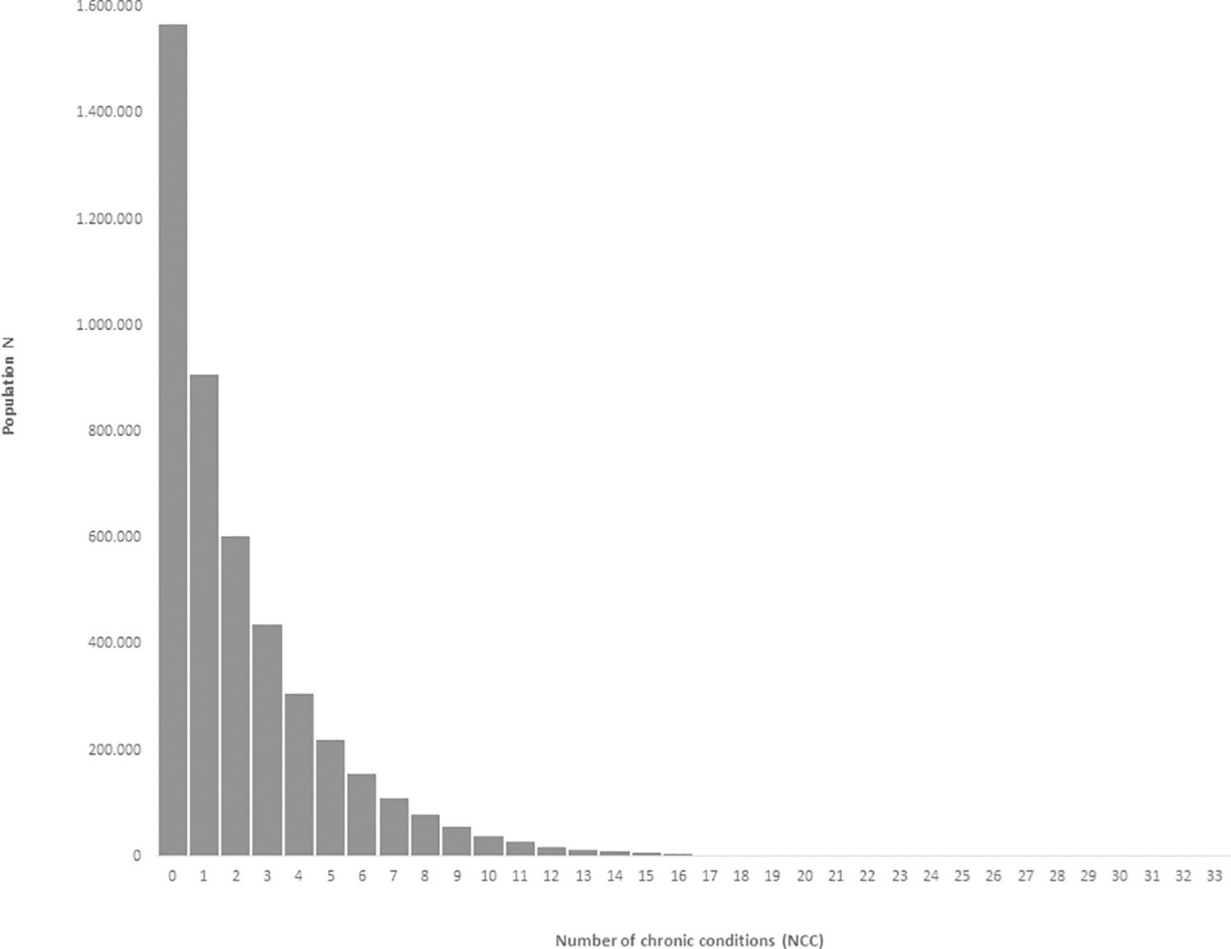
NCC in the Danish population.

The NCCs ranged from 0 to 32 conditions with a highly left-skewed distribution for the population (Fig 1). Overall, 34.4% of the population had no chronic condition, and 65.6% had one or more chronic conditions–e.g. 6.6% had seven or more chronic conditions, and 1.9% had ten or more chronic conditions (S1 Table).

**Fig 2:**
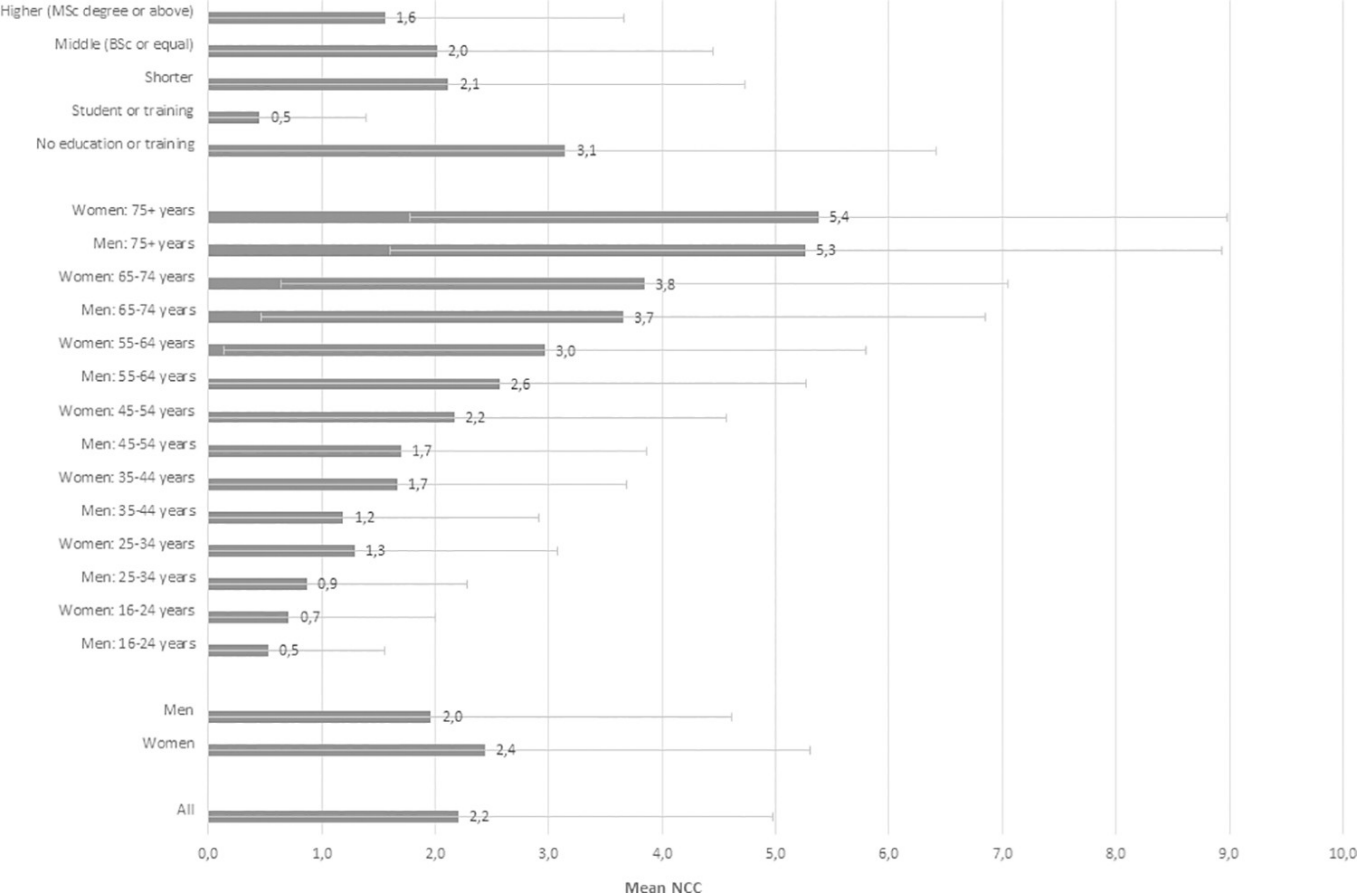
Mean NCC and one +/- standard deviation (SD)–for the entire population, sex, age groups and educational attainment.

Overall, the mean NCCs in the population was 2.2 –with a mean of 2.4 among women and 2.0 among men. The mean NCCs increased by age, and women had a higher mean of chronic conditions than men, although this gap narrowed with age (Fig 2). We found a social gradient with the mean of chronic conditions increasing with lower educational attainment. Thus, individuals with no education had the highest mean of chronic conditions (mean = 3.1), and individuals with higher education had the smallest mean (mean = 1.6)–except for the student category, where the mean was 0.5 (Fig 2).

**Fig 3:**
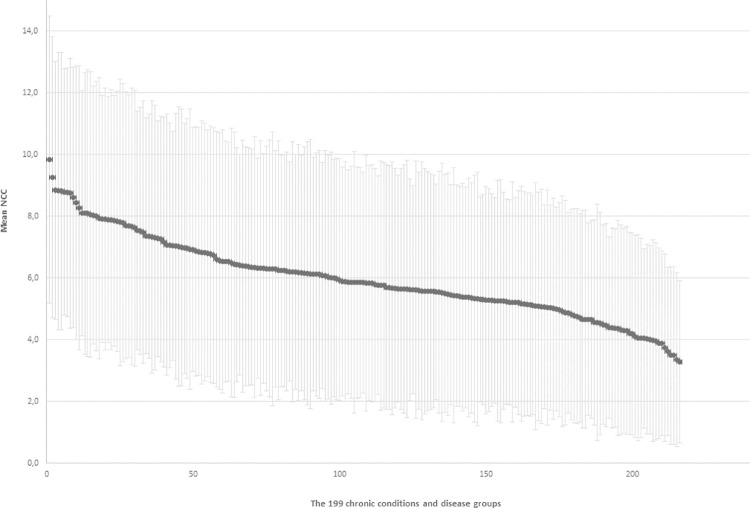
Mean NCC and one +/- SD within the 199 conditions and disease groups. The black dotted line is the national population mean of 2.2, and the blue dotted line indicates the average of 3.4 among those with one or more chronic conditions.

The mean NCCs across the 199 chronic conditions and the disease groups range from around 3 to 9, with the main proportion of conditions having a mean between 5 and 7 chronic conditions (Fig 3).

**Table 1** displays the mean NCC for the 14 disease groups and sex and age. Disease group N (chronic renal failure) had the highest mean NCCs (mean = 8.8), followed by disease group D (in situ and benign neoplasms; mean = 6.5), K (diseases of the digestive system; mean = 5.7), and H (diseases of the eye and adnexa and diseases of the ear and mastoid process; mean = 5.6). Disease group C (cancers), followed by disease group E (endocrine, nutritional and metabolic diseases), G (diseases of the nervous system), I (diseases of the circulatory system), and F (mental and behavioural disorders), had a mean of NCCs ranging from 4.8–5.4. Finally, disease group L (diseases of the skin and subcutaneous tissue), M (diseases of the musculoskeletal system and connective tissue), J (diseases of the respiratory system), and Q (congenital malformations) had a mean of NCC ranging from 4.0–4.7. Of the 14 disease groups, sex differences were among others found in disease group D (female = 6.2 vs male = 7.0), K (female = 5.9 vs male = 5.4) and J (female = 4.4 vs male = 4.0). For more details about the overall mean NCCs of the 199 chronic conditions and means by sex and age, see S2 Table.

**Table 1:** Overview of mean NCCs and SD of disease groups and medicines: The number of patients, overall mean NCCs, and by age and sex in Denmark on 1 January 2013.

Name of condition	ICD-10 code / definition	Total Population					Sex and Age									
							Female		Male		Age 16–44		Age 45–74		Age 75+	
		*N*	*Mean*	*Std*.	*SD*	*Rank*	*Raw*	*SD*	*Raw*	*SD*	*Raw*	*SD*	*Raw*	*SD*	*Raw*	*SD*
B–Viral hepatitis and human immunodeficiency virus [HIV] disease	B18, B20–B24	8,500	4.4	(4.7)	3.5	206	4.3	3.6	4.5	3.5	3.6	3.1	5.0	3.7	8.5	4.2
C–Malignant neoplasms	C00–C99; D32–D33; D35.2–D35.4; D42–D44	229,331	5.4	(4.2)	3.6	5	5.4	3.6	5.5	3.6	3.2	2.4	5.0	3.4	7.0	3.8
D–In situ and benign neoplasms, and neoplasms of uncertain or unknown behaviour and diseases of the blood and blood-forming organs and certain disorders involving the immune mechanism	D00–D09; D55–D59; D60–D67; D80–D89	116,560	6.5	(5.5)	4.3	2	6.2	4.3	7.0	4.4	3.6	2.8	6.5	4.2	8.9	4.2
E–Endocrine, nutritional and metabolic diseases	E00–E14; E20–E29; E31–35; E70–E78; E84–E85; E88–E89	877,433	5.3	(4.5)	3.3	6	5.3	3.4	5.2	3.3	3.5	2.6	5.0	3.1	6.8	3.6
G–Diseases of the nervous system	G00–G14; G20–G32; G35–G37; G40–47; G50–64; G70–73; G80–G83; G90–G99	561,054	5.1	(4.7)	3.6	7	5.1	3.6	5.1	3.6	3.5	2.6	5.2	3.5	7.9	3.9
H–Diseases of the eye and adnexa and diseases of the ear and mastoid process	H02–H06; H17–H18; H25–H28; H31–H32; H34–H36; H40–55; H57; H80,H810; H93, H90–H93	448,176	5.6	(4.5)	3.6	4	5.8	3.6	5.4	3.6	3.4	2.6	5.3	3.4	6.8	3.7
I–Diseases of the circulatory system	I05–I06; I10–28; I30–33; I36–141; I44–I52; I60–I88; I90–I94; I96–I99	1,254,427	4.9	(4.3)	3.3	8	4.9	3.3	4.8	3.2	3.3	2.5	4.6	3.1	6.2	3.5
J–Diseases of the respiratory system	J30.1; J40–J47; J60–J84; J95, J97–J99	1,210,598	4.2	(3.9)	3.3	13	4.4	3.3	4.0	3.2	2.6	2.0	4.6	3.2	7.3	3.7
K–Diseases of the digestive system	K25–K27; K40, K43, K50–52; K58–K59; K71–K77; K86–K87	329,337	5.7	(5.0)	4.0	3	5.9	4.0	5.4	3.9	3.4	2.7	5.8	3.8	8.3	4.1
L–Diseases of the skin and subcutaneous tissue	L40	65,469	4.7	(4.1)	3.5	10	4.9	3.6	4.4	3.4	2.8	2.2	4.8	3.4	7.6	4.0
M–Diseases of the musculoskeletal system and connective tissue	M01–M25; M30–M36; M40–M54; M60.1–M99	1,032,808	4.7	(4.1)	3.4	11	4.9	3.4	4.4	3.4	2.9	2.2	4.7	3.2	6.9	3.7
N–Diseases of the genitourinary system	N18	20,162	8.8	(7.3)	4.5	1	9.0	4.5	8.7	4.5	5.4	3.6	8.6	4.4	10.0	4.3
Q–Congenital malformations, deformations, and chromosomal abnormalities	Q00–Q56; Q60–Q99	124,898	4.0	(4.2)	3.3	14	4.1	3.3	3.8	3.2	2.8	2.2	5.0	3.5	8.2	4.2
F–Mental and behavioral disorders	F00–99	683,194	4.8	(4.6)	3.5	9	4.9	3.5	4.5	3.5	3.2	2.4	5.3	3.5	7.6	3.9
**Having one or more chronic conditions**		**2,989,441**	**3.4**	(**3.1**)	**2.8**	**n/a**	**3.5**	**2.8**	**3.2**	**2.7**	**2.2**	**1.8**	**3.5**	**2.7**	**5.6**	**3.5**
Depression medicine ^c^ **	ATC: N06A	529,918	4.8	(4.4)	3.7	5	4.9	3.6	4.7	3.7	3.1	2.6	5.2	3.6	7.6	3.9
Antipsychotic medicine ^c^ **	ATC: N05A	138,625	5.5	(5.3)	3.8	3	5.9	3.9	5.0	3.6	4.4	3.1	6.0	3.9	7.3	4.0
Indication prescribed anxiety medicine^c^ **	All prescrib. w.indication codes 163 (for anxiety) or 371 (for anxiety, addictive)	102,568	4.9	(4.6)	3.8	4	5.0	3.8	4.7	3.8	3.5	2.9	5.3	3.9	7.7	4.1
Heart failure medication ^c^ **	ATC: C01AA05, C03, C07 or C09A with indication code 430 (for heart failure)	7,468	8.0	(6.4)	4.1	1	8.3	4.3	7.9	3.9	5.7	3.6	7.5	3.9	9.0	4.1
Ischaemic heart medication ^c^ **	ATC: C01A, C01B, C01D, C01E	129,484	7.4	(5.6)	4.1	2	7.6	4.1	7.2	4.0	4.7	3.8	6.9	4.0	8.0	4.0
**All five types of the medicine above**		**688,006**	**5.1**	** (4.4)**	**3.7**	**n/a**	**5.1**	**3.7**	**5.0**	**3.7**	**3.2**	**2.6**	**5.3**	**3.6**	**7.4**	**3.9**
**Total population**		**4,555,439**	**2.2**	(**2.2)**	**2.8**	**n/a**	**2.4**	**2.9**	**2.0**	**2.6**	**1.1**	**1.6**	**2.7**	**2.8**	**5.3**	**3.6**

Gender and age-standardised estimates (Std.) are in brackets.

ICD-10 International Statistical Classification of Diseases, 10^th^ Revision

^c^ = complex defined conditions; see reference for further details [65].

** 2-year prevalence. n/a: not available.

**Fig 4:**
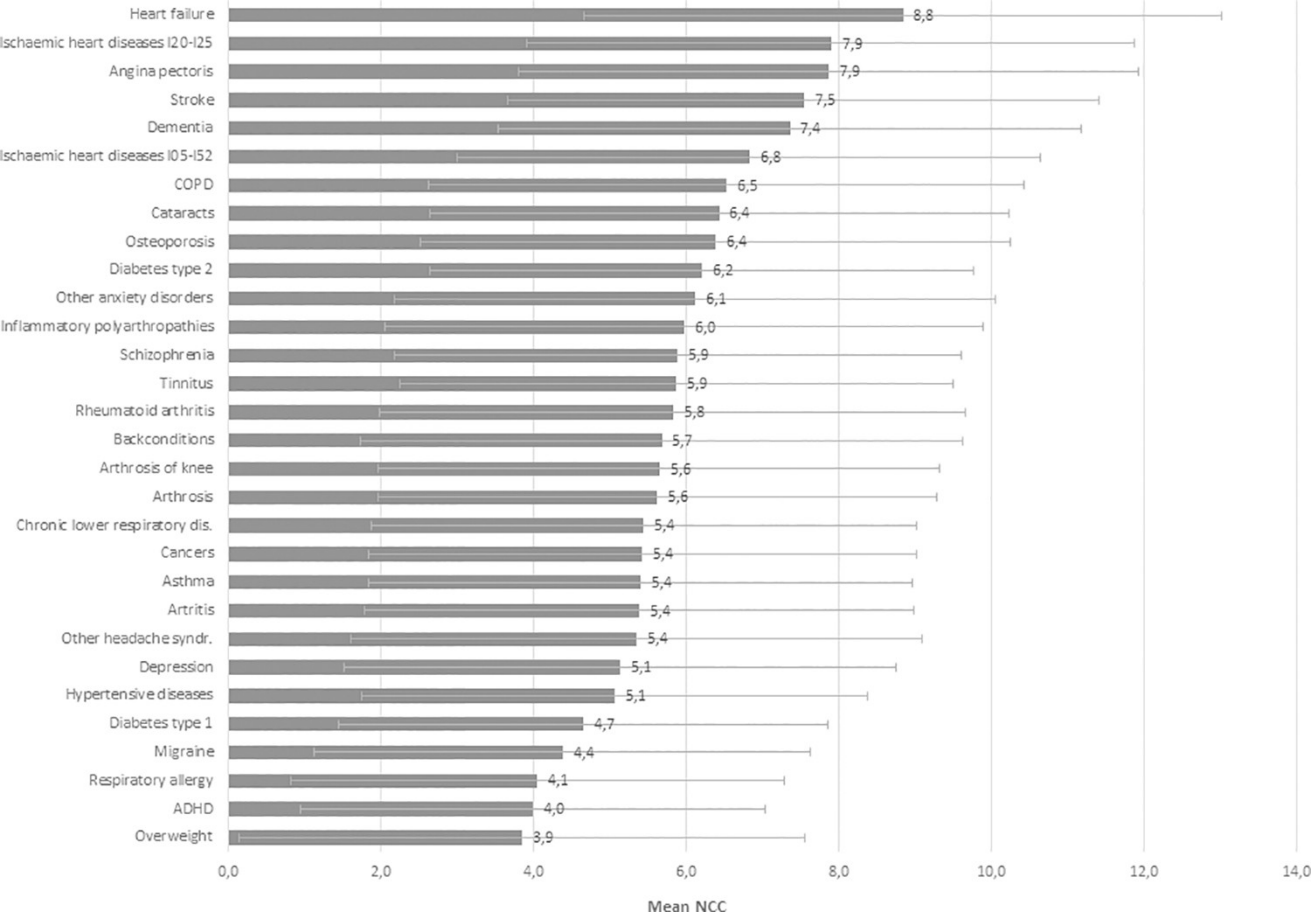
Mean NCCs and one +/- SD for the 29 common conditions and overweight.

Among the 29 most common chronic conditions and overweight, heart disease, stroke, and dementia had more than seven other chronic conditions (Fig 4). Further, chronic obstructive pulmonary disease (COPD), cataracts, osteoporosis, type 2 diabetes, anxiety disorders, and inflammatory polyarthropathy had relatively high NCCs, with a mean above six. The 29 common conditions and overweight had a mean of four chronic conditions or more. S2 and S3 Tables show the prevalence (N), overall mean NCCs, means by sex and age, of the total 199 chronic conditions and the 29 common conditions.

**Table 2** shows the 30 x 30 cross-tabulated prevalence rates between the 29 most common chronic conditions and overweight. The prevalence rates indicate how many per cent within the row condition have the condition in the column. The following commentary is delimited to selected, highly prevalent conditions: hypertensive diseases, arthritis, chronic lower respiratory diseases, depression, type 2 diabetes, cancers and overweight. Hypertensive diseases were associated with respiratory allergy (25.3%), arthritis (24.0%) and ischemic heart diseases (22.6%)–meaning that of the persons with hypertensive diseases, 25.3% also had a respiratory allergy, 24.0% had arthritis, and 22.6 had heart disease. Type 2 diabetes (17.2%), arthrosis (17.0%), depression (16.0%), and chronic lower respiratory diseases (15.2%) were also associated with hypertensive diseases. Arthritis was highly associated with hypertensive diseases (50.4%), gonarthrosis (35.4%), and inflammatory polyarthropathies (32.1%). Moreover, the prevalence of respiratory allergies was high among people with arthritis (26.2%). Chronic lower respiratory diseases were highly associated with asthma (57.7%) and respiratory allergy (43.1%). But high prevalence was also found among hypertensive diseases (38.5%), COPD (29.0%), and arthritis (18.4%). Depression was associated with hypertensive diseases (37.4%), respiratory allergy (27.0%), chronic lower respiratory diseases (15.7%), and arthritis (16.4%). Type 2 diabetes was highly associated with hypertensive diseases (75.4%) as well as diseases such as arthritis (26.3%), respiratory allergy (24.7%), and ischaemic heart diseases (23.4%). Cancers were associated with hypertensive diseases (49.7%), respiratory allergy (23.9%), arthritis (22.2%), and ischaemic heart diseases (17.9%). Moreover, diagnoses like arthrosis (15.8%), depression (14.9%), and chronic lower respiratory diseases (14.8%) were common among patients diagnosed with cancer. Being overweight was associated with hypertensive diseases (39.7%), respiratory allergy (26.6%), depression (19.9%), and arthritis (19.3%). Finally, diagnoses such as type 2 diabetes (17.7%) and chronic lower respiratory diseases (17.0%) were common among people with a BMI higher than 35.

**Table 2:** Catalogue of comorbidity prevalence (per cent within conditions) of 29 common conditions and overweight in Denmark on 1st January 2013.

Name of condition	Cancers	Type 1 diabetes	Type 2 diabetes	Migraine	Other headache syndromes	Diseases of the eye lens (cataracts)	Tinnitus	Ischaemic heart diseases broad	Hypertensive diseases ^c^	Heart failure	Ischaemic heart diseases specific	Angina pectoris	Stroke	Respiratory allergy c	Chronic lower respiratory diseases c	Chronic obstructive lung disease (COPD) c	Asthma, status asthmaticus	Arthritis	Inflammatory polyarthropathies and ankylosing spondylitis c	Rheumatoid arthritis	Arthrosis	Gonarthrosis [arthrosis of knee]	Back conditions	Osteoporosis	Dementia	Schizophrenia	Depression	Other anxiety disorders	Hyperkinetic disorders (ADHD) c	Overweight, clinical (BMI >35)	The rank of all 199+ conditions
Cancers	100.0	0.5	11.2	4.6	0.4	4.2	1.9	17.9	49.7	2.3	7.8	4.0	4.0	23.9	14.8	10.7	11.3	22.2	7.5	3.0	15.8	8.1	7.9	10.5	2.1	0.5	14.9	0.9	0.4	5.9	147
Type 1 diabetes ^c^	4.8	100.0	0.0	2.3	0.3	4.1	0.8	9.7	44.8	1.3	5.7	3.3	2.7	19.3	9.0	4.3	7.7	12.2	5.8	2.5	6.6	2.8	4.6	4.2	0.8	0.9	13.2	0.8	1.1	5.6	197
Type 2 diabetes ^c^	10.6	0.0	100.0	3.1	0.4	5.0	1.7	23.4	75.4	4.2	12.9	7.1	5.4	24.7	16.5	11.2	12.9	26.3	11.2	3.0	17.8	10.2	8.6	6.6	2.3	1.2	17.3	1.1	0.4	16.2	86
Migraine ^c^	7.0	0.4	5.1	100.0	3.9	1.4	1.3	7.2	39.6	0.5	3.2	2.2	1.9	31.2	15.4	6.4	12.6	15.8	4.7	3.0	10.5	5.5	9.0	5.2	0.5	0.7	20.9	1.9	1.4	9.7	204
Other headache syndromes	6.0	0.5	5.4	35.8	100.0	1.6	2.1	9.6	39.6	0.7	5.0	3.6	4.1	29.9	14.2	6.6	12.2	15.0	4.9	2.9	9.9	5.1	12.9	3.9	0.6	0.9	26.0	3.5	1.7	9.7	153
Diseases of the eye lens (cataracts)	14.2	1.4	17.9	3.1	0.4	100.0	1.9	23.6	64.0	3.5	11.1	6.0	5.4	26.6	17.4	14.0	13.6	28.3	9.1	3.7	21.3	11.0	9.3	14.9	2.9	0.4	15.5	0.7	0.2	5.0	68
Tinnitus	10.9	0.4	10.0	5.0	0.9	3.3	100.0	14.7	44.3	1.6	7.3	4.5	3.2	29.6	14.5	8.7	11.8	22.3	6.9	2.9	16.4	8.9	10.1	6.9	1.6	0.7	16.8	1.4	0.7	5.3	107
Ischaemic Heart Diseases broad	13.0	0.7	18.0	3.4	0.5	5.1	1.9	100.0	75.8	11.9	44.1	24.8	7.2	24.9	17.6	14.5	14.0	27.3	10.6	3.4	19.1	10.2	9.9	10.2	3.3	0.6	17.3	1.3	0.5	8.3	57
Hypertensive diseases ^c^	10.8	1.0	17.2	5.6	0.6	4.1	1.7	22.6	100.0	3.4	11.0	6.1	5.1	25.3	15.2	10.1	11.9	24.0	8.4	3.0	17.0	9.2	8.4	8.4	2.3	0.5	16.0	1.0	0.4	8.3	179
Heart failure ^c^	14.1	0.8	26.8	2.1	0.3	6.4	1.7	100.0	96.0	100.0	46.3	19.6	8.0	24.4	22.8	22.4	18.0	32.8	17.7	3.7	19.9	10.4	9.6	11.0	3.5	0.7	18.5	1.2	0.3	10.6	3
Ischaemic heart diseases specific	12.9	0.9	22.4	3.5	0.6	5.4	2.1	100.0	83.8	12.5	100.0	56.4	7.1	25.7	18.9	16.5	15.1	29.0	11.6	3.6	20.3	10.6	11.6	9.9	2.9	0.6	18.7	1.3	0.4	10.0	22
Angina pectoris	11.8	1.0	21.8	4.2	0.8	5.2	2.3	100.0	82.0	9.4	100.0	100.0	5.8	27.0	19.2	15.9	15.3	28.9	11.0	3.7	20.5	10.9	12.4	9.1	1.7	0.6	18.4	1.5	0.4	10.6	24
Stroke	12.5	0.9	17.9	4.0	0.9	5.1	1.8	31.3	73.8	4.1	13.6	6.2	100.0	23.3	15.3	12.3	11.5	24.6	9.2	3.1	17.2	8.7	9.5	10.6	5.6	0.7	28.5	1.3	0.4	6.1	32
Respiratory allergy ^c^	6.5	0.5	7.1	5.6	0.6	2.2	1.4	9.3	31.9	1.1	4.3	2.5	2.0	100.0	21.4	8.9	20.0	15.7	5.2	2.5	10.7	5.7	7.0	5.5	1.1	0.7	14.6	1.2	1.0	7.0	214
Chronic lower respiratory diseases ^c^	8.1	0.5	9.6	5.5	0.6	2.8	1.4	13.3	38.5	2.0	6.3	3.6	2.7	43.1	100.0	29.0	57.7	18.4	6.4	2.9	12.6	6.6	8.2	8.2	1.3	0.9	17.1	1.5	1.1	9.0	145
Chronic obstructive lung disease (COPD) ^c^	11.3	0.5	12.5	4.5	0.5	4.4	1.6	21.2	49.6	3.9	10.6	5.8	4.1	34.8	56.1	100.0	56.2	21.5	7.8	3.3	15.0	7.4	9.4	12.8	2.1	1.1	19.8	1.5	0.8	7.7	64
Asthma, status asthmaticus^c^	7.2	0.5	8.6	5.2	0.6	2.6	1.3	12.2	34.9	1.9	5.8	3.3	2.3	46.7	66.8	33.6	100.0	16.6	5.7	2.6	11.4	5.9	7.5	7.6	1.1	1.0	16.9	1.5	1.4	8.8	148
Artritis	10.0	0.6	12.6	4.7	0.5	3.8	1.8	17.0	50.4	2.4	8.0	4.5	3.5	26.2	15.2	9.2	11.8	100.0	32.1	15.3	66.9	35.4	12.1	9.7	1.9	0.4	14.8	0.9	0.5	8.4	149
Inflammatory polyarthropathies and ankylosing spondylitis ^c^	10.3	0.8	16.3	4.2	0.5	3.7	1.7	20.2	53.9	4.0	9.7	5.2	4.0	26.3	16.1	10.2	12.5	97.8	100.0	46.6	23.1	12.1	12.1	11.1	1.7	0.4	14.5	0.8	0.4	8.7	103
Rheumatoid arthritis ^c^	9.0	0.7	9.3	5.8	0.6	3.3	1.5	14.1	41.7	1.8	6.6	3.7	2.9	27.4	15.9	9.2	12.3	100.0	100.0	100.0	23.1	11.2	13.4	16.0	1.4	0.4	14.5	1.0	0.5	7.5	115
Arthrosis	10.7	0.4	12.7	4.7	0.5	4.3	1.9	17.8	53.3	2.2	8.3	4.8	3.7	26.7	15.6	9.6	12.1	100.0	11.3	5.3	100.0	52.9	13.4	10.2	2.2	0.3	15.2	0.9	0.4	9.1	130
Gonarthrosis [arthrosis of knee]	10.4	0.4	13.8	4.6	0.5	4.2	2.0	18.0	54.5	2.2	8.3	4.8	3.5	27.0	15.4	9.0	11.9	100.0	11.2	4.8	100.0	100.0	12.3	9.2	2.1	0.3	14.6	0.8	0.4	10.9	125
Backconditions	8.5	0.5	9.8	6.3	1.0	3.0	1.9	14.7	41.9	1.7	7.6	4.6	3.2	27.7	16.1	9.6	12.8	28.7	9.5	4.9	21.3	10.3	100.0	9.6	1.5	0.5	18.9	1.3	0.9	8.4	122
Osteoporosis ^c^	15.1	0.6	10.0	4.9	0.4	6.4	1.8	20.3	56.2	2.6	8.7	4.5	4.9	29.3	21.5	17.4	17.3	30.8	11.6	7.8	21.7	10.3	12.9	100.0	4.5	0.4	19.8	0.9	0.3	3.8	71
Dementia^c^	12.9	0.5	15.4	1.9	0.3	5.4	1.7	27.9	65.5	3.6	11.0	3.6	11.1	26.2	15.0	12.1	10.7	26.4	7.8	2.9	20.4	10.1	8.9	19.3	100.0	0.9	51.0	1.6	0.4	2.9	36
Schizophrenia ^c^	3.7	0.7	10.0	3.5	0.5	0.8	0.9	6.5	18.9	0.9	2.6	1.5	1.7	19.2	12.7	8.0	12.5	6.1	2.1	1.0	3.6	1.7	3.4	1.9	1.1	100.0	37.6	6.9	4.4	11.2	105
Depression ^c^	7.5	0.7	9.2	6.9	0.9	2.3	1.5	12.0	37.4	1.5	5.7	3.2	4.5	27.0	15.7	9.4	13.4	16.4	5.3	2.5	11.3	5.8	8.8	6.9	4.1	2.4	100.0	5.1	3.5	9.7	173
Other anxiety disorders	5.6	0.5	7.0	7.4	1.5	1.3	1.5	10.8	29.0	1.2	4.9	3.1	2.5	26.9	16.3	8.8	14.7	11.5	3.7	1.9	7.6	3.7	7.5	3.9	1.5	5.4	61.3	100.0	6.0	12.0	95
Hyperkinetic disorders (ADHD) ^c^	2.1	0.6	2.4	4.7	0.6	0.3	0.6	3.5	11.0	0.2	1.2	0.8	0.7	19.6	10.6	4.2	12.1	5.5	1.7	0.9	3.3	1.5	4.6	0.9	0.3	3.0	37.3	5.3	100.0	7.2	217
Overweight, clinical (BMI >35)	6.2	0.6	17.7	6.6	0.7	1.6	1.0	11.8	39.7	1.8	6.3	3.8	2.0	26.6	17.0	7.5	14.4	19.3	6.5	2.6	13.9	8.8	8.1	2.8	0.5	1.5	19.9	2.1	1.4	100.0	222
**All the above conditions**	**8.9**	**0.9**	**9.4**	**5.8**	**0.6**	**2.7**	**1.6**	**12.3**	**41.3**	**1.5**	**5.4**	**3.1**	**2.8**	**32.8**	**16.3**	**8.4**	**14.1**	**19.7**	**6.5**	**3.0**	**13.2**	**7.0**	**8.3**	**6.2**	**1.4**	**1.1**	**17.7**	**1.5**	**1.7**	**8.6**	**n/a**
**Total population**	**7.7**	**0.8**	**8.1**	**5.0**	**0.6**	**2.3**	**1.3**	**10.6**	**35.5**	**1.3**	**4.7**	**2.6**	**2.4**	**28.2**	**14.0**	**7.2**	**12.1**	**16.9**	**5.5**	**2.6**	**11.3**	**6.0**	**7.1**	**5.3**	**1.2**	**1.0**	**15.2**	**1.3**	**1.4**	**6.2**	**n/a**

^c^ = complex defined conditions; see reference for further details [65]. n/a: not available.

For further details, the S4 Table shows the frequencies and percentages of the 199 conditions cross-tabulated with the 29 most common conditions. S5 Table (spreadsheet) shows the frequencies and percentages of the 199 conditions cross-tabulated with all 199 chronic conditions, disease groups, and common medicines.

**Table 3** presents the overall mean NCCs, the mean NCCs for patients with no education and patients with higher educational attainment, and the prevalence of having 1, 2, 3, 4, 5, 6, or 7+ conditions within each disease across all 199 chronic conditions, disease groups, and overweight. S6 Table shows the means of the 199 conditions and all five levels of educational attainment.

**Table 3:** Catalogue of means, SD of the NCCs and prevalence (per cent within conditions) for the 199 chronic conditions: Overall population means, means by educational levels and prevalence by NCCs in Denmark on 1 January 2013. Sorted by ICD10 diagnosis.

			Overall, NCCs of the population				Education							NCCs in Per Cent													
No.	Name of condition	ICD-10 code / definition					No education or training			Higher (MSc degree or doctorate)				1		2		3		4		5		6		7+	
				Means			Means			Means			Ratio	Per Cent		Per Cent		Per Cent		Per Cent		Per Cent		Per Cent		Per Cent	
			N*	*Raw*	*Std*.	*SD*	*Raw*	*Std*.	*SD*	*Raw*	*Std*.	*SD*		*Raw*	*Std*.	*Raw*	*Std*.	*Raw*	*Std*.	*Raw*	*Std*.	*Raw*	*Std*.	*Raw*	*Std*.	*Raw*	*Std*.
	**B–Viral hepatitis and human immunodeficiency virus [HIV] disease**	**B18, B20–B24**	**8,500**	**4.4**	**(4.7)**	**3.5**	**5.3**	**(5.6)**	**3.8**	**3.1**	**(3.4)**	**2.5**	**1.7**	**19.6**	**(20.4)**	**17.7**	**(16.5)**	**14.6**	**(12.9)**	**10.3**	**(10.0)**	**8.6**	**(8.0)**	**7.0**	**(6.9)**	**22.3**	**(25.3)**
1	Chronic viral hepatitis	B18	4,584	5.0	(5.3)	3.8	5.8	(6.1)	4.0	3.5	(3.9)	3.1	1.7	18.7	(17.8)	17.7	(14.5)	14.2	(10.9)	11.6	(10.1)	9.9	(7.5)	9.6	(8.0)	18.5	(31.3)
2	Human immunodeficiency virus [HIV] disease	B20–24	4,229	3.9	(4.2)	3.2	4.8	(5.0)	3.7	3.2	(3.2)	2.4	1.5	23.2	(21.8)	21.2	(18.6)	17.8	(15.1)	11.5	(9.7)	9.5	(8.8)	6.2	(5.6)	10.7	(20.4)
	**C–Malignant neoplasms**	**C00–C99; D32–D33; D35.2–D35.4; D42–D44**	**229,331**	**5.4**	**(4.2)**	**3.6**	**6.2**	**(4.6)**	**3.8**	**4.3**	**(3.5)**	**3.1**	**1.4**	**10.2**	**(17.8)**	**14.7**	**(19.4)**	**15.5**	**(15.5)**	**14.8**	**(12.6)**	**12.9**	**(9.3)**	**10.9**	**(7.0)**	**21.1**	**(18.3)**
3	Malignant neoplasms of other and unspecified localizations	C00–C14; C30–C33; C37–C42; C45–C49; C69; C73–74; C754–C759	20,557	5.9	(4.7)	3.7	6.6	(5.2)	3.9	4.6	(4.0)	3.1	1.4	6.7	(10.9)	13.4	(17.1)	16.1	(16.6)	15.4	(13.8)	13.3	(10.4)	11.6	(7.9)	23.4	(23.3)
4	Malignant neoplasms of digestive organs	C15–C17; C22–C26	4,839	6.8	(5.4)	4.0	7.2	(5.7)	4.1	5.9	(4.6)	3.8	1.2	4.8	(7.9)	9.9	(14.4)	12.2	(12.9)	14.4	(15.8)	14.2	(9.7)	13.6	(9.2)	30.9	(30.2)
5	Malignant neoplasm of colon	C18	18,826	6.4	(4.5)	3.9	6.9	(4.9)	4.0	5.1	(3.6)	3.2	1.4	5.8	(14.9)	10.8	(18.6)	13.8	(16.7)	14.6	(11.0)	14.6	(10.4)	12.7	(6.7)	27.6	(21.8)
6	Malignant neoplasms of rectosigmoid junction, rectum, anus and anal canal	C19–C21	10,680	5.8	(4.4)	3.5	6.2	(4.7)	3.7	4.9	(4.8)	3.1	1.3	7.0	(12.5)	12.4	(18.2)	15.1	(17.3)	15.7	(15.4)	14.1	(10.2)	12.0	(6.8)	23.7	(19.6)
7	Malignant neoplasm of bronchus and lung	C34	14,762	7.2	(5.5)	4.1	7.6	(5.9)	4.1	5.9	(4.5)	3.4	1.3	5.0	(9.4)	8.1	(12.4)	11.1	(14.5)	13.8	(13.2)	14.5	(10.7)	13.6	(8.0)	33.8	(31.9)
8	Malignant melanoma of skin	C43	19,636	4.4	(3.5)	3.2	5.4	(3.9)	3.6	3.4	(3.0)	2.7	1.6	17.5	(25.4)	19.6	(22.6)	16.4	(15.0)	13.8	(11.3)	10.6	(7.5)	8.1	(5.5)	14.0	(12.8)
9	Other malignant neoplasms of skin	C44	15,597	5.8	(3.9)	3.8	6.5	(4.3)	4.0	4.8	(3.3)	3.4	1.4	10.0	(23.2)	13.3	(19.7)	14.3	(12.1)	14.2	(14.8)	13.0	(7.7)	11.1	(5.6)	24.0	(16.8)
10	Malignant neoplasm of breast	C50	50,687	5.2	(4.3)	3.4	5.9	n/a	3.6	4.0	n/a	2.8	1.5	9.5	(13.6)	15.3	(14.7)	16.4	(13.6)	15.4	(10.1)	13.0	(16.3)	10.7	(7.2)	19.7	(19.3)
11	Malignant neoplasms of female genital organs	C51–C52; C56–C58	7,245	5.3	(4.2)	3.4	6.1	n/a	3.6	3.8	n/a	2.6	1.6	10.6	(7.8)	15.0	(8.1)	15.5	(12.0)	14.7	(6.4)	12.3	(4.5)	11.2	(17.9)	20.6	(23.7)
12	Malignant neoplasm of cervix uteri, corpus uteri and part unspecified	C53–C55	11,608	5.0	(2.0)	3.3	5.8	n/a	3.5	3.6	n/a	2.7	1.6	11.4	(9.5)	15.8	(8.9)	16.4	(8.6)	14.6	(6.3)	12.6	(4.6)	10.5	(3.1)	18.7	(8.4)
13	Malignant tumor of male genitalia	C60, C62–C63	5,194	3.5	(4.3)	2.9	4.4	n/a	3.4	2.9	n/a	2.3	1.5	27.1	(12.5)	23.2	(10.4)	16.6	(11.0)	11.5	(5.5)	7.5	(7.1)	5.3	(2.7)	8.9	(31.1)
14	Malignant neoplasm of prostate	C61	26,697	5.5	(4.7)	3.5	6.0	n/a	3.6	4.9	n/a	3.2	1.2	8.3	(5.4)	13.2	(11.3)	15.4	(11.1)	14.8	(11.9)	13.8	(8.5)	12.0	(6.6)	22.6	(25.6)
15	Malignant neoplasms of urinary tract	C64–C68	10,319	6.2	(4.6)	3.7	6.7	(4.8)	3.8	5.4	(4.0)	3.5	1.2	6.6	(16.0)	10.9	(16.9)	13.4	(12.2)	14.4	(14.4)	14.0	(9.5)	12.7	(8.1)	27.9	(22.9)
16	Brain cancer ^c^	C71, C75.1–C75.3, D33.0–D33.2, D35.2–D35.4, D43.0–D43.2, D44.3–D44.5 (brain). C70, D32, D42 (brain membrane). C72, D33.3–D33.9, D43.3–D43.9 (cranial nerve, spinal cord)	15,310	6.2	(5.4)	3.8	7.0	(5.9)	3.9	5.0	(4.5)	3.2	1.4	4.2	(6.2)	11.7	(13.9)	15.0	(15.5)	15.7	(14.2)	14.3	(11.6)	12.4	(9.3)	26.6	(29.3)
17	Malignant neoplasms of ill-defined, secondary and unspecified sites, and of independent (primary) multiple sites	C76–C80, C97	25,619	6.4	(5.2)	3.6	7.1	(5.5)	3.7	5.3	(4.5)	3.1	1.3	1.3	(3.5)	10.2	(16.1)	14.8	(17.1)	16.3	(15.7)	15.2	(12.2)	13.5	(9.1)	28.6	(26.4)
18	Malignant neoplasms, stated or presumed to be primary, of lymphoid, haematopoietic and related tissue	C81–C96	19,712	5.8	(4.6)	3.8	6.6	(5.0)	3.9	4.7	(4.0)	3.3	1.4	9.9	(15.3)	13.7	(17.1)	14.1	(14.4)	14.5	(12.6)	13.2	(10.2)	11.4	(7.9)	23.2	(22.5)
	**D–In situ and benign neoplasms, and neoplasms of uncertain or unknown behavior and diseases of the blood and blood-forming organs and certain disorders involving the immune mechanism**	**D00–D09; D55–D59; D60–D67; D80–D89**	**116,560**	**6.5**	**(5.2)**	**4.3**	**7.7**	**(5.8)**	**4.4**	**4.7**	**(4.2)**	**3.7**	**1.6**	**10.9**	**(14.7)**	**13.4**	**(14.9)**	**13.2**	**(13.0)**	**12.7**	**(10.8)**	**12.2**	**(9.4)**	**11.2**	**(7.7)**	**26.4**	**(29.5)**
19	In situ neoplasms	D00–D09	19,810	4.8	(4.1)	3.5	5.9	(4.7)	3.7	3.5	(3.3)	2.7	1.7	15.4	(20.2)	18.1	(19.3)	15.8	(14.2)	14.1	(12.4)	11.3	(9.5)	9.2	(6.4)	16.2	(18.0)
20	Hemolytic anemias	D55–D59	3,055	5.5	(5.2)	4.2	6.7	(5.8)	4.5	4.3	(4.3)	3.4	1.5	16.7	(15.6)	16.8	(15.0)	14.1	(12.5)	12.1	(10.5)	11.7	(9.7)	8.7	(7.3)	19.9	(29.6)
21	Aplastic and other anemias	D60–D63	14,918	8.1	(6.2)	4.7	8.9	(6.7)	4.6	6.7	(5.4)	4.5	1.3	5.8	(8.3)	9.1	(12.3)	10.7	(11.6)	11.5	(10.9)	12.4	(9.7)	13.4	(8.7)	37.1	(38.4)
22	Other anemias	D64	46,613	8.1	(6.1)	4.6	8.7	(6.5)	4.5	6.9	(5.4)	4.6	1.3	5.6	(10.3)	8.4	(12.5)	9.8	(11.4)	11.3	(9.6)	13.2	(9.8)	13.6	(8.1)	38.1	(38.2)
23	Coagulation defects, purpura and other hemorrhagic conditions	D65–D69	25,376	5.6	(5.3)	4.2	7.0	(6.1)	4.5	4.0	(4.3)	3.3	1.7	15.0	(14.7)	16.5	(14.6)	14.8	(12.6)	13.0	(10.6)	11.4	(9.3)	9.7	(7.8)	19.5	(30.3)
24	Other diseases of blood and blood-forming organs	D70–D77	8,896	6.6	(5.7)	4.1	7.6	(6.4)	4.4	5.1	(4.5)	3.7	1.5	7.7	(10.8)	11.5	(12.7)	13.4	(12.2)	14.0	(11.6)	13.1	(10.4)	11.9	(8.3)	28.2	(34.1)
25	Certain disorders involving the immune mechanism	D80–D89	7,660	5.8	(5.6)	4.0	6.9	(6.2)	4.4	4.6	(4.7)	3.4	1.5	11.1	(10.8)	14.9	(13.3)	15.3	(13.5)	13.7	(11.6)	12.8	(10.4)	10.9	(8.6)	21.3	(31.9)
	**E–Endocrine, nutritional and metabolic diseases**	**E00–E14; E20–E29; E31–35; E70–E78; E84–E85; E88–E89**	**877,433**	**5.3**	(4.3)	**3.3**	**5.8**	**(4.6)**	**3.5**	**4.4**	**(3.7)**	**3.0**	**1.3**	**7.9**	**(16.1)**	**14.8**	**(18.2)**	**16.9**	**(16.1)**	**15.8**	**(13.0)**	**13.6**	**(10.1)**	**11.0**	**(7.6)**	**19.9**	**(18.9)**
26	Diseases of the thyroid ^c^	E00–E04, E06, E07	131,908	5.1	(4.3)	3.5	6.1	(4.8)	3.8	3.8	(3.6)	2.9	1.6	12.3	(18.3)	16.2	(18.1)	15.9	(14.8)	14.5	(12.4)	12.5	(9.6)	10.1	(7.4)	18.5	(19.4)
27	Thyrotoxicosis ^c^	E05	41,374	5.3	(4.2)	3.6	6.2	(4.6)	3.8	3.6	(3.6)	2.8	1.7	11.9	(18.9)	15.9	(19.1)	15.6	(15.2)	14.3	(11.4)	12.3	(9.1)	10.2	(6.9)	19.8	(19.4)
28	Diabetes type 1 ^c^	E10	23,062	4.7	(4.7)	3.2	5.4	(5.2)	3.4	4.0	(4.0)	2.7	1.3	14.8	(13.9)	17.0	(15.3)	16.2	(14.3)	14.3	(12.7)	12.3	(11.0)	9.2	(8.4)	16.2	(24.3)
29	Diabetes type 2 ^c^	E11	242,177	6.2	(5.1)	3.6	6.6	(5.3)	3.7	5.3	(4.5)	3.2	1.3	3.1	(10.2)	8.7	(13.8)	15.7	(15.4)	16.9	(13.6)	15.5	(11.3)	13.6	(9.2)	26.5	(26.5)
30	Diabetes others ^c^	E12–E14	1,117	6.3	(5.4)	4.4	7.3	(6.0)	4.7	5.9	(5.3)	5.0	1.2	12.7	(14.9)	13.7	(14.0)	12.5	(11.4)	15.1	(12.4)	11.6	(9.2)	9.8	(7.0)	24.5	(31.2)
31	Disorders of other endocrine glands	E20–E35, except E30	28,650	5.6	(5.5)	4.2	7.1	(6.3)	4.4	4.4	(4.6)	3.4	1.6	14.8	(12.1)	15.6	(13.3)	15.1	(13.0)	13.0	(11.2)	11.2	(9.7)	10.1	(8.5)	20.3	(32.2)
32	Metabolic disorders	E70–E77; E79–E83; E85, E88–E89;	23,690	6.3	(5.7)	4.2	7.4	(6.3)	4.4	4.5	(4.4)	3.4	1.7	8.8	(10.5)	13.2	(13.1)	14.5	(12.9)	13.8	(11.3)	12.9	(10.2)	11.5	(8.7)	25.2	(33.4)
33	Disturbances in lipoprotein circulation and other lipids ^c^	E78	652,242	5.6	(4.8)	3.4	6.1	(5.1)	3.5	4.9	(4.3)	3.0	1.2	5.0	(10.6)	12.9	(15.6)	16.7	(16.0)	16.4	(13.8)	14.6	(11.1)	12.1	(9.0)	22.3	(23.9)
34	Cystic fibrosis ^c^	E84	947	4.2	(4.9)	3.3	5.8	(5.9)	3.8	2.9	(3.9)	2.4	2.0	18.0	(13.3)	23.0	(17.6)	16.9	(14.1)	13.1	(12.3)	9.1	(9.4)	7.4	(7.7)	12.6	(25.7)
	**G–Diseases of the nervous system**	**G00–G14; G20–G32; G35–G37; G40–47; G50–64; G70–73; G80–G83; G90–G99**	**561,054**	**5.1**	**(4.6)**	**3.6**	**6.0**	**(5.1)**	**3.8**	**4.1**	**(3.7)**	**3.0**	**1.5**	**13.2**	**(16.3)**	**16.2**	**(16.9)**	**15.6**	**(14.5)**	**14.0**	**(12.0)**	**12.0**	**(9.7)**	**9.9**	**(7.7)**	**18.9**	**(22.8)**
35	Inflammatory diseases of the central nervous system	G00–G09	7,642	5.6	(5.2)	4.0	6.7	(6.0)	4.3	4.5	(4.3)	3.6	1.5	13.6	(14.7)	15.5	(14.9)	14.3	(12.4)	13.6	(11.7)	11.4	(9.2)	9.7	(7.5)	21.7	(29.6)
36	Systemic atrophies primarily affecting the central nervous system and other degenerative diseases	G10–G14, G30–G32	10,401	7.3	(5.5)	4.0	7.7	(5.9)	4.0	6.1	(4.7)	4.0	1.3	4.0	(10.2)	7.4	(12.9)	10.8	(13.6)	13.3	(12.1)	14.0	(9.8)	14.7	(9.7)	35.7	(31.6)
37	Parkinson’s disease ^c^	G20, G21, G22, F02.3	57,583	7.0	(6.5)	4.1	7.4	(6.8)	4.1	5.8	(5.4)	3.6	1.3	4.3	(4.0)	9.0	(8.2)	12.4	(10.9)	14.0	(11.5)	14.8	(12.1)	14.0	(11.6)	31.5	(41.7)
38	Extrapyramidal and movement disorders	G23–G26	10,837	6.8	(5.8)	4.3	7.6	(6.4)	4.4	5.6	(4.9)	4.0	1.4	6.9	(9.6)	11.0	(12.4)	13.0	(12.1)	13.6	(11.8)	13.4	(10.5)	12.1	(8.7)	30.0	(35.0)
39	Sclerosis	G35	13,284	4.7	(4.4)	3.1	5.4	(5.0)	3.5	3.8	(3.7)	2.3	1.4	10.5	(11.6)	17.6	(18.0)	18.4	(17.2)	16.5	(15.2)	12.9	(10.7)	9.6	(8.0)	14.7	(19.4)
40	Demyelinating diseases of the central nervous system	G36–G37	4,571	5.3	(5.3)	3.4	6.4	(6.1)	3.9	4.2	(4.4)	2.7	1.5	7.0	(7.0)	15.1	(14.2)	17.4	(15.4)	16.1	(14.3)	14.3	(11.2)	11.4	(9.2)	18.7	(28.7)
41	Epilepsy ^c^	G40–G41	61,695	6.2	(5.7)	4.2	6.7	(6.1)	4.3	5.1	(4.6)	3.7	1.3	11.9	(12.4)	13.5	(12.8)	13.2	(11.5)	12.8	(10.5)	12.3	(9.6)	11.1	(8.5)	25.2	(34.7)
42	Migraine ^c^	G43	149,866	4.4	(4.3)	3.2	5.5	(5.1)	3.8	3.3	(3.4)	2.5	1.7	16.5	(18.4)	19.7	(18.4)	17.4	(15.4)	14.0	(12.0)	10.8	(9.2)	8.1	(6.9)	13.4	(19.8)
43	Other headache syndromes	G44	16,469	5.4	(5.6)	3.7	6.4	(6.3)	4.3	4.4	(4.6)	2.9	1.5	9.9	(9.2)	15.8	(13.9)	16.6	(13.5)	15.2	(12.4)	12.8	(10.5)	10.6	(8.9)	19.1	(31.6)
44	Transient cerebral ischemic attacks and related syndromes and vascular syndromes of brain in cerebrovascular diseases	G45–G46	43,977	7.1	(5.6)	4.0	7.7	(6.0)	4.1	6.0	(4.8)	3.7	1.3	3.3	(7.6)	7.7	(12.7)	12.2	(13.7)	14.5	(12.2)	15.1	(11.3)	14.4	(9.8)	32.9	(32.7)
45	Sleep disorders	G47	36,806	5.6	(5.4)	3.8	6.4	(6.1)	4.2	4.7	(4.4)	3.4	1.4	11.0	(11.4)	14.9	(14.2)	15.3	(13.5)	14.0	(11.7)	12.8	(10.6)	10.8	(8.2)	21.2	(30.4)
46	Disorders of trigeminal nerve and facial nerve disorders	G50–G51	21,488	5.3	(4.5)	3.9	6.3	(5.1)	4.3	3.7	(3.5)	3.0	1.7	16.3	(20.4)	16.3	(16.8)	14.9	(13.5)	12.9	(10.6)	11.3	(8.6)	9.3	(6.7)	19.0	(23.3)
47	Disorders of other cranial nerves, cranial nerve disorders in diseases classified elsewhere, nerve root and plexus disorders and nerve root and plexus compressions in diseases classified elsewhere	G52–G55	12,429	6.0	(5.3)	4.0	6.8	(5.8)	4.3	5.2	(4.7)	3.7	1.3	7.6	(9.1)	13.5	(14.3)	14.8	(14.8)	15.0	(13.1)	13.0	(10.3)	12.1	(9.4)	24.0	(28.9)
48	Mononeuropathies of upper limb	G56	122,395	5.2	(4.5)	3.6	5.9	(4.9)	3.9	4.3	(3.7)	3.2	1.4	13.2	(17.8)	16.2	(17.2)	15.5	(14.3)	14.2	(12.0)	12.1	(9.5)	10.1	(7.5)	18.7	(21.8)
49	Mononeuropathies of lower limb, other mononeuropathies and mononeuropathy in diseases classified elsewhere	G57–G59	18,627	5.7	(4.9)	3.9	6.5	(5.5)	4.1	4.6	(4.2)	3.6	1.4	10.6	(14.1)	14.7	(16.1)	14.9	(14.0)	14.4	(12.1)	12.7	(9.9)	11.0	(7.9)	21.7	(25.9)
50	Polyneuropathies and other disorders of the peripheral nervous system	G60–G64	30,289	7.3	(5.9)	4.4	8.1	(6.4)	4.5	6.0	(4.8)	4.1	1.3	5.7	(9.6)	9.4	(12.6)	11.9	(12.6)	13.3	(11.3)	13.3	(9.7)	13.3	(8.9)	33.1	(35.4)
51	Diseases of myoneural junction and muscle	G70–G73	5,758	5.8	(5.4)	4.1	6.6	(6.0)	4.4	4.8	(4.6)	3.5	1.4	12.1	(12.7)	14.8	(13.9)	15.0	(13.4)	13.8	(11.7)	11.7	(9.6)	10.4	(8.3)	22.2	(30.4)
52	Cerebral palsy and other paralytic syndromes	G80–G83	14,410	6.0	(5.9)	4.1	6.2	(6.2)	4.1	5.5	(5.2)	4.0	1.1	9.5	(8.3)	13.6	(11.8)	14.9	(12.6)	14.1	(11.9)	12.9	(10.7)	11.5	(9.5)	23.4	(35.3)
53	Other disorders of the nervous system	G90–G99	44,394	6.4	(5.6)	4.1	7.1	(6.2)	4.3	5.2	(4.7)	3.7	1.4	7.4	(8.4)	12.3	(13.2)	13.7	(13.1)	14.2	(12.3)	13.5	(10.8)	12.2	(9.1)	26.7	(33.1)
	**H–Diseases of the eye and adnexa and diseases of the ear and mastoid process**	**H02–H06; H17–H18; H25–H28; H31–H32; H34–H36; H40–55; H57; H80,H810; H93, H90–H93**	**448,176**	**5.6**	**(4.4)**	**3.6**	**6.3**	**(4.8)**	**3.7**	**4.4**	**(3.6)**	**3.1**	**1.4**	**9.4**	**(16.9)**	**13.3**	**(17.9)**	**14.7**	**(15.1)**	**14.7**	**(12.4)**	**13.5**	**(9.8)**	**11.6**	**(7.5)**	**22.7**	**(20.4)**
54	Disorders of eyelid, lacrimal system and orbit	H02–H06	13,191	5.6	(4.3)	3.8	6.5	(4.9)	4.0	4.1	(3.4)	2.9	1.6	10.9	(18.2)	15.6	(19.4)	15.1	(14.5)	13.7	(11.1)	12.5	(8.8)	10.1	(6.9)	22.1	(21.1)
55	Corneal scars and opacities	H17	2,173	5.6	(4.6)	4.0	6.5	(5.1)	4.2	4.5	(4.1)	4.3	1.4	13.7	(17.0)	15.8	(17.3)	14.9	(15.3)	13.9	(11.4)	10.0	(7.9)	10.4	(7.7)	21.3	(23.4)
56	Other disorders of cornea	H18	9,473	5.6	(4.3)	3.8	6.6	(4.9)	4.0	4.2	(3.7)	3.2	1.6	12.9	(19.2)	14.9	(18.1)	14.0	(14.6)	13.0	(11.2)	11.9	(8.8)	10.9	(7.5)	22.4	(20.7)
57	Diseases of the eye lens (cataracts)	H25–H28	68,009	6.4	(5.0)	3.8	6.8	(5.4)	3.9	5.6	(4.3)	3.5	1.2	5.6	(12.8)	10.0	(15.4)	12.9	(13.5)	14.5	(12.3)	14.7	(10.6)	13.6	(8.7)	28.7	(26.6)
58	Disorders of the choroid and retina	H31–H32	1,900	5.6	(4.5)	3.9	6.5	(5.1)	4.1	3.7	(3.4)	2.8	1.7	13.1	(18.0)	13.9	(16.1)	16.8	(15.3)	13.1	(11.1)	11.1	(8.8)	10.1	(7.9)	22.0	(22.8)
59	Retinal vascular occlusions	H34	10,358	6.9	(5.0)	3.9	7.5	(5.4)	4.0	5.7	(4.2)	3.4	1.3	3.9	(10.9)	8.1	(15.8)	12.6	(14.8)	14.8	(11.2)	15.2	(10.7)	14.4	(10.5)	31.0	(26.1)
60	Other retinal disorders	H35	68,485	6.5	(4.7)	3.9	7.1	(5.1)	4.0	5.3	(4.0)	3.6	1.3	6.2	(15.0)	10.0	(15.7)	12.6	(14.6)	14.1	(12.7)	14.5	(10.1)	13.4	(8.0)	29.3	(23.8)
61	Retinal disorders in diseases classified elsewhere	H36	19,279	7.3	(6.2)	3.8	8.0	(6.6)	3.9	5.9	(5.3)	3.4	1.3	0.2	(0.3)	6.3	(12.8)	10.4	(13.2)	15.8	(13.4)	17.2	(12.3)	15.8	(10.6)	34.3	(37.3)
62	Glaucoma ^c^	H40–H42	67,310	5.9	(4.5)	3.6	6.4	(5.0)	3.7	4.9	(4.0)	3.3	1.3	7.7	(15.4)	11.7	(16.7)	14.0	(14.7)	15.2	(12.6)	14.1	(10.4)	12.3	(8.1)	24.9	(22.1)
63	Disorders of the vitreous body and globe	H43–H45	7,572	5.6	(4.6)	3.9	7.0	(5.5)	4.2	4.1	(3.4)	3.1	1.7	11.0	(16.3)	15.8	(18.6)	15.0	(14.4)	13.7	(11.0)	12.2	(9.2)	10.7	(7.2)	21.5	(23.2)
64	Disorders of optic nerve and visual pathways	H46–H48	6,184	5.4	(5.3)	3.6	6.3	(6.0)	3.9	4.2	(4.2)	2.9	1.5	9.6	(9.2)	14.9	(13.6)	15.9	(14.3)	15.5	(13.2)	13.6	(11.8)	10.5	(9.1)	20.0	(28.8)
65	Disorders of ocular muscles, binocular movement, accommodation and refraction	H49–H52	18,247	4.1	(4.3)	3.3	5.5	(5.2)	3.9	3.0	(3.5)	2.4	1.8	24.6	(21.9)	20.9	(18.2)	15.2	(13.4)	11.7	(10.6)	8.8	(8.1)	7.0	(6.9)	11.9	(20.9)
66	Visual disturbances	H53	22,232	6.2	(5.3)	4.1	7.1	(5.9)	4.2	4.8	(4.3)	3.6	1.5	9.7	(12.1)	13.2	(14.8)	13.9	(13.3)	13.9	(12.0)	13.0	(9.9)	11.4	(8.3)	24.9	(29.5)
67	Blindness and partial sight	H54	6,614	7.8	(6.5)	4.6	8.3	(6.9)	4.7	6.4	(5.6)	4.3	1.3	5.5	(6.8)	9.2	(10.3)	10.9	(10.1)	13.2	(11.6)	13.0	(10.0)	13.7	(9.9)	34.5	(41.2)
68	Nystagmus and other irregular eye movements and other disorders of eye and adnexa	H55, H57	11,133	5.7	(5.1)	4.0	6.7	(5.9)	4.2	4.5	(4.2)	3.2	1.5	10.9	(12.0)	15.1	(15.6)	14.9	(13.9)	13.8	(11.7)	12.4	(10.2)	10.6	(8.4)	22.3	(28.2)
69	Otosclerosis	H80	10,360	5.3	(4.2)	3.5	6.2	(4.7)	3.7	3.9	(3.5)	3.0	1.6	10.4	(15.6)	15.9	(20.6)	16.2	(16.0)	14.5	(12.6)	12.4	(9.1)	10.9	(7.6)	19.8	(18.5)
70	Ménière’s disease ^c^	H810	10,003	6.2	(4.8)	3.8	7.0	(5.2)	3.9	4.9	(4.1)	3.5	1.4	7.0	(11.5)	11.2	(16.2)	14.0	(16.8)	13.8	(12.4)	13.8	(11.2)	13.9	(8.7)	26.2	(23.1)
71	Other diseases of the inner ear	H83	29,865	6.3	(5.1)	3.6	6.8	(5.3)	3.7	5.7	(4.8)	3.5	1.2	3.5	(7.6)	9.8	(15.8)	14.1	(15.2)	15.1	(14.3)	15.4	(10.8)	13.8	(9.2)	28.4	(27.1)
72	Conductive and sensorineural hearing loss	H90	43,238	5.9	(4.6)	3.7	6.6	(5.1)	3.9	4.7	(3.8)	3.2	1.4	8.2	(13.1)	13.3	(17.3)	14.7	(15.7)	14.4	(12.8)	13.7	(10.2)	11.9	(8.0)	23.9	(22.8)
73	Other hearing loss and other disorders of ear, not elsewhere classified	H910, H912, H913, H918, H930, H932, H933	8,306	6.3	(5.3)	3.8	7.0	(5.7)	4.0	5.0	(4.4)	3.3	1.4	5.7	(8.4)	11.2	(13.7)	14.0	(14.7)	15.0	(13.4)	14.6	(12.4)	12.6	(8.6)	26.9	(28.9)
74	Presbycusis (age-related hearing loss)	H911	80,659	7.0	(5.0)	3.7	7.3	(5.2)	3.8	6.4	(4.5)	3.6	1.2	2.6	(10.1)	6.7	(14.0)	11.0	(15.8)	14.2	(14.3)	15.7	(10.8)	15.3	(9.0)	34.6	(25.9)
75	Hearing loss, unspecified	H919	87,806	6.3	(4.8)	3.7	7.0	(5.3)	3.9	5.1	(4.0)	3.2	1.4	4.7	(9.9)	10.7	(16.6)	14.3	(16.7)	14.8	(13.4)	14.7	(10.8)	13.3	(8.3)	27.6	(24.3)
76	Tinnitus	H931	40,124	5.9	(4.8)	3.6	6.8	(5.4)	3.8	4.3	(3.9)	3.0	1.6	6.5	(11.4)	12.3	(16.6)	15.2	(15.5)	15.2	(12.9)	14.2	(10.5)	12.3	(8.5)	24.3	(24.6)
77	Other specified disorders of ear	H938	20,537	6.1	(4.7)	3.7	6.7	(5.1)	3.8	5.0	(4.0)	3.2	1.3	5.8	(12.4)	11.1	(17.0)	14.8	(16.1)	15.3	(13.3)	14.5	(10.9)	13.0	(7.8)	25.5	(22.6)
	**I–Diseases of the circulatory system**	**I05–I06; I10–28; I30–33;I36–141; I44–I52; I60–I88; I90–I94; I96–I99**	**1,254,427**	**4.9**	**(4.0)**	**3.3**	**5.4**	**(4.4)**	**3.4**	**4.0**	**(3.4)**	**2.8**	**1.4**	**11.7**	**(18.9)**	**16.4**	**(19.2)**	**17.0**	**(16.2)**	**15.1**	**(12.7)**	**12.6**	**(9.5)**	**9.9**	**(7.0)**	**17.3**	**(16.6)**
78	Aortic and mitral valve disease^c^	I05, I06, I34, I35	30,123	8.0	(5.8)	4.2	8.6	(6.2)	4.2	6.7	(5.1)	3.9	1.3	2.3	(8.6)	5.9	(13.2)	9.2	(12.5)	12.2	(11.4)	14.4	(10.2)	15.0	(8.7)	40.9	(35.4)
79	Hypertensive diseases ^c^	I10–I15	1,060,046	5.1	(4.2)	3.3	5.6	(4.5)	3.5	4.3	(3.7)	2.9	1.3	10.1	(15.6)	15.5	(18.6)	16.8	(16.6)	15.3	(13.3)	13.1	(10.2)	10.5	(7.5)	18.6	(18.2)
80	Heart failure ^c^	I11.0, I13.0, I13.2, I42.0, I42.6, I42.7, I42.9, I50.0, I50.1, I50.9	37,540	8.8	(7.0)	4.2	9.2	(7.3)	4.3	8.0	(6.4)	3.9	1.1	0.5	(3.1)	2.4	(6.2)	6.9	(9.9)	11.1	(11.4)	15.0	(11.2)	16.8	(11.4)	47.3	(46.7)
80A	Ischemic heart diseases	I20–I25	139,173	7.9	(6.2)	4.0	8.5	(6.5)	4.1	6.8	(5.3)	3.6	1.2	1.8	(7.4)	4.2	(10.0)	8.5	(10.9)	13.0	(12.0)	15.7	(10.8)	16.3	(10.0)	40.5	(38.8)
81	Angina pectoris	I20	78,476	7.9	(6.1)	4.1	8.5	(6.6)	4.2	6.7	(5.1)	3.6	1.3	2.3	(8.8)	4.8	(10.1)	8.7	(11.1)	12.6	(11.0)	15.6	(10.5)	16.0	(9.1)	40.0	(39.3)
82	Acute myocardial infarction and subsequent myocardial infarction	I21–I22	36,654	8.1	(6.7)	4.0	8.7	(7.0)	4.1	7.0	(5.9)	3.4	1.2	0.6	(4.0)	2.9	(8.8)	7.4	(8.7)	13.3	(11.8)	16.3	(11.0)	17.4	(11.9)	42.2	(43.7)
83	AMI complex/other	I23–I24	2,969	9.3	(7.1)	4.6	9.8	(7.4)	4.4	8.7	(6.9)	5.4	1.1	2.6	(6.2)	4.7	(9.8)	5.4	(7.3)	10.2	(11.1)	12.9	(7.9)	15.6	(9.9)	48.6	(47.8)
84	Chronic ischemic heart disease	I25	84,592	8.8	(7.1)	4.0	9.3	(7.5)	4.1	7.8	(6.4)	3.6	1.2	0.4	(3.4)	1.4	(5.5)	4.7	(8.2)	10.8	(11.4)	15.7	(12.0)	17.9	(11.9)	49.1	(47.6)
85	Pulmonary heart disease and diseases of pulmonary circulation	I26–I28	15,352	7.7	(6.1)	4.5	8.5	(6.6)	4.6	5.9	(4.9)	4.1	1.4	4.8	(7.7)	8.8	(11.5)	11.8	(13.3)	12.9	(11.8)	13.1	(9.9)	14.0	(9.2)	34.6	(36.6)
86	Acute pericarditis	I30	5,563	5.1	(5.1)	4.0	6.1	(5.7)	4.5	4.0	(4.0)	2.9	1.5	17.9	(15.7)	18.3	(16.1)	15.3	(13.5)	12.0	(10.4)	9.9	(9.1)	9.0	(7.9)	17.5	(27.4)
87	Other forms of heart disease	I31–I43, except I34–I35 and I42	8,119	8.0	(6.6)	4.6	8.9	(7.1)	4.7	6.7	(5.5)	4.1	1.3	4.9	(6.7)	9.8	(12.6)	10.1	(9.6)	11.3	(9.8)	13.2	(9.6)	13.2	(8.6)	37.5	(43.0)
88	Atrioventricular and left bundle branch block	I44	14,604	7.9	(5.6)	4.2	8.4	(6.0)	4.3	6.6	(4.7)	3.9	1.3	3.6	(10.5)	6.8	(13.3)	9.7	(13.3)	11.9	(11.7)	13.6	(9.1)	15.1	(8.7)	39.3	(33.4)
89	Other conduction disorders	I45–46	11,823	7.6	(6.0)	4.5	8.7	(6.7)	4.6	6.2	(5.2)	4.1	1.4	5.2	(8.3)	9.3	(12.1)	11.5	(13.0)	12.8	(11.4)	12.9	(9.8)	13.4	(8.8)	35.0	(36.6)
90	Paroxysmal tachycardia	I47	39,510	6.7	(5.3)	4.1	7.7	(5.8)	4.3	5.5	(4.4)	3.7	1.4	6.9	(11.5)	10.7	(14.3)	13.2	(13.9)	13.5	(11.8)	13.7	(10.1)	12.7	(8.5)	29.3	(29.8)
91	Atrial fibrillation and flutter	I48	112,342	7.4	(5.4)	3.9	7.9	(5.7)	4.0	6.2	(4.7)	3.7	1.3	2.7	(9.6)	6.7	(12.3)	11.2	(14.6)	13.6	(13.0)	15.0	(11.2)	14.9	(9.0)	36.0	(30.3)
92	Other cardiac arrhythmias	I49	34,418	7.1	(5.3)	4.2	8.1	(5.8)	4.3	5.9	(4.5)	3.9	1.4	6.3	(11.7)	9.8	(14.1)	12.0	(14.1)	13.4	(12.4)	13.2	(9.9)	13.2	(8.5)	32.1	(29.4)
93	Complications and ill-defined descriptions of heart disease and other heart disorders in diseases classified elsewhere	I51–52	7,337	8.3	(6.5)	4.6	9.2	(7.0)	4.6	6.2	(5.1)	4.1	1.5	3.7	(6.2)	7.2	(10.4)	10.3	(11.6)	12.6	(11.5)	14.0	(10.2)	13.6	(9.3)	38.7	(40.8)
94	Stroke	I60, I61,I63–I64, Z501 (rehabilitation)	72,606	7.5	(6.2)	3.9	7.9	(6.4)	4.0	6.8	(5.7)	3.7	1.2	1.6	(4.5)	4.8	(9.8)	10.1	(12.0)	14.0	(12.5)	15.8	(12.2)	15.6	(10.2)	38.2	(38.8)
95	Cerebrovascular diseases	I62, I65–I68	17,308	7.8	(6.1)	4.2	8.6	(6.6)	4.3	6.5	(5.0)	3.8	1.3	3.5	(8.3)	6.1	(10.6)	9.2	(11.5)	12.3	(10.7)	14.9	(11.1)	15.0	(9.7)	38.9	(38.2)
96	Sequelae of cerebrovascular disease	I69	50,952	8.8	(7.3)	4.0	9.1	(7.5)	4.1	8.1	(6.8)	3.8	1.1	0.5	(1.4)	2.1	(5.1)	5.5	(8.6)	10.7	(10.4)	15.1	(12.4)	16.7	(11.3)	49.4	(51.0)
97	Atherosclerosis	I70	32,064	8.7	(6.7)	4.4	9.0	(6.9)	4.4	7.9	(6.0)	4.3	1.1	1.7	(7.6)	4.3	(9.8)	7.7	(9.4)	11.8	(11.1)	14.2	(9.4)	15.7	(9.6)	44.6	(43.3)
98	Aortic aneurysm and aortic dissection	I71	10,296	7.9	(5.8)	4.0	8.3	(6.1)	4.0	6.7	(5.2)	3.7	1.2	2.0	(8.2)	4.8	(10.6)	8.9	(10.5)	12.7	(12.6)	15.6	(13.1)	15.7	(10.1)	40.4	(35.0)
99	Diseases of arteries, arterioles and capillaries	I72, I74, I77–I79	11,830	7.0	(5.6)	4.5	8.4	(6.5)	4.6	5.2	(4.4)	3.7	1.6	9.6	(14.2)	12.1	(13.9)	11.9	(11.7)	12.9	(10.5)	12.9	(8.9)	11.3	(7.5)	29.3	(33.2)
100	Other peripheral vascular diseases	I73	28,508	7.9	(5.7)	4.2	8.3	(6.1)	4.2	6.9	(5.1)	4.2	1.2	2.6	(10.5)	5.8	(12.9)	10.2	(12.1)	13.0	(11.2)	14.9	(10.1)	15.0	(8.9)	38.4	(34.4)
101	Phlebitis, thrombosis of the portal vein and others	I80–I82	37,388	6.2	(5.1)	4.1	7.1	(5.7)	4.3	4.6	(4.1)	3.4	1.5	9.3	(12.5)	13.2	(15.2)	14.7	(14.7)	14.1	(12.2)	12.7	(9.8)	11.5	(7.8)	24.5	(27.8)
102	Varicose veins of lower extremities	I83	23,530	4.3	(3.8)	3.4	5.4	(4.3)	3.8	3.3	(3.2)	2.8	1.6	20.1	(25.7)	20.2	(20.0)	16.7	(14.8)	13.0	(10.4)	9.8	(7.5)	7.4	(5.8)	12.8	(15.8)
103	Hemorrhoids ^c^	I84	74,285	4.3	(4.1)	3.4	5.6	(4.7)	4.0	3.1	(3.4)	2.5	1.8	20.9	(22.6)	19.8	(18.5)	15.9	(14.2)	12.7	(11.0)	10.0	(8.6)	7.6	(6.5)	13.1	(18.6)
104	Oesophageal varices (chronic), varicose veins of other sites, other disorders of veins, non-specific lymphadenitis, other non-infective disorders of lymphatic vessels and lymph nodes and other and unspecified disorders of the circulatory system	I85–I99, except I89 and I95	15,194	6.1	(5.3)	4.4	7.4	(6.0)	4.5	4.5	(4.4)	3.9	1.6	14.1	(15.8)	14.5	(14.3)	13.5	(12.3)	12.3	(10.4)	10.9	(8.6)	10.8	(7.9)	23.9	(30.7)
	**J–Diseases of the respiratory system**	**J30.1; J40–J47; J60–J84; J95, J97–J99**	**1,210,598**	**4.2**	**(3.8)**	**3.3**	**5.4**	**(4.5)**	**3.7**	**3.0**	**(3.1)**	**2.4**	**1.8**	**22.4**	**(24.9)**	**18.9**	**(19.0)**	**16.1**	**(15.0)**	**12.5**	**(10.9)**	**9.6**	**(8.0)**	**7.4**	**(6.0)**	**13.1**	**(16.3)**
105	Respiratory allergy ^c^	J30, except J30.0	841,685	4.1	(3.7)	3.2	5.3	(4.4)	3.7	3.0	(3.1)	2.4	1.8	24.5	(26.5)	18.6	(18.3)	16.1	(14.9)	12.4	(10.9)	9.3	(7.9)	7.0	(5.8)	12.1	(15.6)
105A	Chronic lower respiratory diseases ^c^	J40–J43, J47	418,120	5.4	(4.8)	3.6	6.6	(5.4)	3.9	4.0	(4.0)	2.6	1.6	7.5	(8.1)	14.8	(16.7)	18.6	(19.5)	15.8	(14.4)	12.9	(10.4)	10.4	(7.8)	20.1	(23.2)
106	Bronchitis, not specified as acute or chronic, simple and mucopurulent chronic bronchitis and unspecified chronic bronchitis	J40–J42	12,790	9.8	(7.5)	4.7	10.4	(7.9)	4.6	8.0	(6.4)	4.5	1.3	0.0	(0.0)	3.6	(7.8)	6.2	(9.7)	10.8	(12.5)	12.4	(10.8)	16.3	(10.4)	50.6	(48.7)
107	Emphysema	J43	5,557	8.6	(6.8)	4.2	9.1	(7.1)	4.2	7.5	(6.1)	4.3	1.2	0.0	(0.0)	4.7	(9.7)	7.7	(12.6)	12.0	(12.1)	15.6	(11.6)	16.4	(10.6)	43.5	(43.5)
108	Chronic obstructive lung disease (COPD) ^c^	J44, J96, J13–J18	216,184	6.5	(5.3)	3.9	7.3	(5.7)	4.0	5.2	(4.5)	3.4	1.4	5.3	(7.8)	9.9	(13.5)	13.7	(15.9)	15.5	(15.1)	14.3	(11.2)	13.0	(8.7)	28.3	(27.8)
109	Asthma, status asthmaticus ^c^	J45–J46	361,129	5.4	(5.0)	3.6	6.6	(5.5)	3.9	4.1	(4.2)	2.5	1.6	6.8	(7.7)	15.7	(16.2)	19.2	(18.5)	15.8	(14.1)	12.7	(10.5)	10.2	(8.1)	19.6	(24.9)
110	Bronchiectasis	J47	4,362	7.5	(6.5)	4.0	8.4	(7.1)	4.3	6.3	(5.5)	3.4	1.3	0.0	(0.0)	5.9	(6.9)	11.1	(13.6)	15.4	(14.1)	16.7	(14.3)	14.8	(10.7)	36.1	(40.4)
111	Other diseases of the respiratory system	J60–J84; J95, J97–J99	21,993	7.9	(6.4)	4.6	8.6	(6.8)	4.6	6.9	(5.7)	4.4	1.3	5.8	(8.6)	8.4	(10.5)	10.1	(10.2)	12.5	(11.0)	13.5	(9.7)	13.1	(8.8)	36.6	(41.2)
	**K–Diseases of the digestive system**	**K25–K27; K40, K43, K50–52; K58–K59; K71–K77; K86–K87**	**329,337**	**5.7**	**(4.8)**	**4.0**	**6.7**	**(5.3)**	**4.1**	**4.4**	**(3.9)**	**3.4**	**1.5**	**13.0**	**(16.9)**	**14.8**	**(16.2)**	**14.3**	**(13.5)**	**13.3**	**(11.2)**	**12.0**	**(9.3)**	**10.4**	**(7.5)**	**22.2**	**(25.4)**
112	Ulcers ^c^	K25–K27	157,379	6.3	(5.1)	4.1	7.1	(5.6)	4.2	5.2	(4.3)	3.7	1.4	10.5	(16.0)	12.3	(14.6)	13.1	(12.5)	13.2	(10.9)	12.7	(9.4)	11.6	(7.8)	26.6	(28.8)
113	Inguinal hernia	K40	25,032	4.3	(3.8)	3.3	5.0	(4.1)	3.6	3.8	(3.3)	3.0	1.3	21.4	(26.8)	19.4	(19.3)	15.8	(14.0)	12.5	(10.7)	9.8	(7.7)	7.5	(5.6)	13.6	(15.9)
114	Ventral hernia	K43	7,941	6.5	(5.3)	4.3	7.3	(5.8)	4.3	5.3	(4.5)	3.9	1.4	9.0	(15.3)	12.0	(13.7)	13.6	(11.9)	13.2	(11.4)	13.0	(9.5)	11.5	(7.5)	27.6	(30.6)
115	Crohn’s disease	K50	18,913	4.9	(4.9)	3.6	6.0	(5.6)	4.1	3.8	(4.2)	2.8	1.6	14.2	(13.2)	19.2	(17.1)	17.1	(14.9)	13.4	(11.6)	11.3	(9.8)	8.8	(7.6)	15.9	(25.6)
116	Ulcerative colitis	K51	29,538	4.9	(4.6)	3.7	6.3	(5.3)	4.3	3.5	(3.8)	2.6	1.8	15.7	(16.0)	18.5	(17.7)	16.4	(15.0)	13.8	(12.2)	10.8	(9.2)	8.7	(7.1)	16.1	(22.7)
117	Other non-infective gastroenteritis and colitis	K52	20,844	7.0	(5.8)	4.5	8.1	(6.4)	4.6	5.3	(4.7)	3.8	1.5	7.5	(9.5)	11.9	(13.3)	13.6	(13.6)	13.0	(11.1)	13.0	(10.1)	12.3	(8.4)	28.7	(33.9)
118	Irritable bowel syndrome (IBS)	K58	37,593	5.2	(4.9)	3.8	6.5	(5.6)	4.3	3.9	(3.9)	3.0	1.7	13.9	(14.8)	17.8	(17.1)	16.0	(14.2)	14.0	(11.8)	11.4	(9.4)	9.3	(7.4)	17.7	(25.3)
119	Other functional intestinal disorders	K59	51,933	6.9	(5.7)	4.5	8.0	(6.5)	4.6	5.4	(4.6)	4.0	1.5	9.0	(11.4)	12.3	(13.7)	13.0	(12.3)	13.0	(10.9)	12.6	(9.6)	11.7	(8.1)	28.3	(34.0)
120	Diseases of liver, biliary tract and pancreas	K71–K77; K86–K87	26,956	6.6	(5.7)	4.2	7.3	(6.3)	4.3	5.4	(4.7)	3.8	1.3	7.8	(10.8)	11.2	(12.9)	13.7	(12.9)	13.7	(11.2)	13.0	(9.9)	12.5	(8.7)	28.0	(33.7)
	**L–Diseases of the skin and subcutaneous tissue**	**L40**	**65,469**	**4.7**	(4.0)	**3.5**	**5.7**	**(4.6)**	**3.9**	**3.5**	**(3.2)**	**2.8**	**1.6**	**19.2**	**(24.9)**	**17.7**	**(18.3)**	**15.3**	**(13.8)**	**13.1**	**(10.8)**	**10.7**	**(8.1)**	**8.5**	**(6.2)**	**15.6**	**(17.9)**
121	Psoriasis ^c^	L40	65,469	4.7	(4.0)	3.5	5.7	(4.6)	3.9	3.5	(3.2)	2.8	1.6	19.2	(24.9)	17.7	(18.3)	15.3	(13.8)	13.1	(10.8)	10.7	(8.1)	8.5	(6.2)	15.6	(17.9)
	**M–Diseases of the musculoskeletal system and connective tissue**	**M01–M25; M30–M36; M40–M54; M60.1–M99**	**1,032,808**	**4.7**	(3.9)	**3.4**	**5.6**	**(4.4)**	**3.6**	**3.7**	**(3.3)**	**2.8**	**1.5**	**15.9**	**(21.5)**	**17.5**	**(19.5)**	**16.0**	**(15.2)**	**13.8**	**(11.7)**	**11.4**	**(8.8)**	**9.1**	**(6.5)**	**16.3**	**(16.8)**
122	Infectious arthropathies	M01–M03	9,402	5.1	(4.9)	3.7	6.2	(5.5)	4.2	4.1	(4.2)	3.0	1.5	13.7	(13.8)	15.9	(15.2)	17.0	(15.4)	14.2	(12.5)	11.9	(10.1)	9.4	(7.9)	17.9	(25.2)
122A	Inflammatory polyarthropathies and ankylosing spondylitis ^c^	M05–M14, M45	165,944	6.0	(4.8)	3.9	6.9	(5.3)	4.1	4.4	(3.9)	3.3	1.6	8.7	(12.6)	13.3	(16.7)	14.7	(15.2)	14.5	(12.8)	13.2	(10.0)	11.6	(8.0)	23.7	(24.6)
123	Rheumatoid arthritis ^c^	M05, M06, M07.1, M07.2, M07.3, M08, M09	77,345	5.8	(4.9)	3.8	6.9	(5.4)	4.1	4.0	(3.8)	3.1	1.7	8.4	(11.4)	14.1	(16.7)	15.5	(15.8)	14.6	(13.1)	13.1	(10.2)	11.6	(8.2)	22.6	(24.6)
124	Inflammatory polyarthropathies–except rheumatoid arthritis ^c^	M074–M079, M10–M14, M45	115,945	6.3	(5.2)	4.0	7.1	(5.6)	4.2	5.1	(4.5)	3.4	1.4	6.5	(8.6)	12.0	(15.8)	14.4	(15.5)	14.9	(13.4)	13.9	(10.8)	12.3	(8.6)	26.0	(27.3)
125	Polyarthrosis [arthrosis]	M15	16,935	7.7	(5.7)	4.3	8.4	(6.2)	4.5	6.7	(5.1)	4.0	1.3	3.3	(8.9)	8.0	(12.2)	10.7	(12.9)	13.3	(12.4)	14.0	(10.6)	14.4	(9.3)	36.3	(33.6)
126	Coxarthrosis [arthrosis of hip]	M16	104,115	6.2	(4.5)	3.8	6.7	(4.8)	3.9	5.1	(3.9)	3.4	1.3	7.4	(14.8)	11.6	(17.3)	14.0	(16.1)	14.6	(13.2)	13.8	(9.8)	12.3	(7.3)	26.3	(21.5)
127	Gonarthrosis [arthrosis of knee]	M17	178,811	5.6	(4.3)	3.7	6.4	(4.7)	3.9	4.6	(3.7)	3.2	1.4	9.5	(16.1)	13.4	(18.6)	15.0	(16.0)	14.7	(12.6)	13.4	(9.7)	11.4	(7.1)	22.6	(19.8)
128	Arthrosis of first carpometacarpal joint and other arthrosis	M18–M19	91,101	6.1	(4.8)	4.0	6.9	(5.3)	4.2	5.2	(4.1)	3.6	1.3	8.6	(14.4)	12.9	(16.8)	14.5	(14.4)	14.4	(12.6)	13.2	(9.6)	11.9	(7.8)	24.6	(24.3)
129	Acquired deformities of fingers and toes	M20	55,730	5.0	(4.2)	3.6	6.0	(4.8)	3.9	3.8	(3.4)	2.9	1.6	14.1	(20.4)	17.1	(19.1)	15.8	(14.4)	14.0	(11.3)	11.6	(8.5)	9.5	(6.6)	17.8	(19.7)
130	Other acquired deformities of limbs	M21	20,584	5.5	(4.8)	3.8	6.6	(5.4)	4.2	4.3	(3.9)	3.2	1.5	12.2	(14.9)	15.4	(16.7)	15.6	(14.7)	13.9	(11.8)	11.9	(9.5)	10.4	(7.7)	20.7	(24.7)
131	Disorders of patella (knee cap)	M22	38,999	3.3	(4.1)	2.6	4.2	(4.9)	3.2	2.6	(3.3)	1.8	1.6	28.4	(20.7)	23.5	(18.8)	16.7	(15.1)	11.2	(11.1)	7.7	(8.9)	5.3	(6.8)	7.2	(18.6)
132	Internal derangement of knee	M230, M231, M233, M235, M236, M238	9,192	3.6	(4.4)	2.7	4.6	(5.1)	3.5	3.2	(3.8)	2.2	1.5	19.5	(14.2)	24.9	(19.1)	19.2	(16.6)	13.3	(13.0)	9.2	(10.0)	5.4	(6.8)	8.6	(20.2)
133	Derangement of meniscus due to old tear or injury	M232	36,374	4.0	(4.2)	2.9	4.9	(4.8)	3.4	3.3	(3.5)	2.3	1.5	16.8	(16.2)	21.3	(19.8)	18.6	(16.6)	13.7	(12.5)	10.3	(9.4)	7.4	(7.1)	11.9	(18.5)
134	Internal derangement of knee, unspecified	M239	28,206	3.9	(4.3)	3.0	4.9	(5.0)	3.6	3.2	(3.5)	2.3	1.5	21.1	(17.9)	21.9	(19.0)	17.4	(15.5)	12.9	(12.1)	9.3	(9.2)	6.9	(7.1)	10.5	(19.2)
135	Other specific joint derangements	M24, except M240–M241	5,923	3.7	(4.6)	3.0	4.8	(5.3)	3.7	3.0	(3.8)	2.2	1.6	25.0	(16.1)	22.1	(16.9)	17.5	(15.5)	11.8	(11.9)	8.0	(8.6)	5.7	(7.0)	10.1	(23.9)
136	Other joint disorders, not elsewhere classified	M25	12,043	5.3	(5.2)	3.7	6.2	(5.7)	4.0	4.4	(4.4)	3.3	1.4	11.4	(11.8)	16.1	(15.0)	16.1	(14.3)	14.8	(12.5)	12.2	(9.8)	10.0	(8.3)	19.4	(28.2)
137	Systemic connective tissue disorders	M30–M36, except M32,M34	42,631	6.8	(5.5)	4.2	7.7	(5.9)	4.3	5.4	(4.7)	3.7	1.4	6.1	(10.0)	10.5	(13.9)	13.0	(13.4)	13.9	(12.0)	13.8	(10.5)	13.0	(8.8)	29.7	(31.4)
138	Systemic lupus erythematosus	M32	3,376	7.5	(7.1)	4.3	8.5	(7.7)	4.6	6.0	(6.0)	3.1	1.4	3.3	(2.9)	7.2	(6.7)	11.1	(10.1)	14.4	(11.5)	14.6	(11.5)	15.5	(10.4)	33.9	(46.9)
139	Dermatopolymyositis	M33	1,137	7.0	(5.9)	4.3	7.7	(6.2)	4.3	4.8	(4.4)	3.2	1.6	6.0	(10.3)	10.7	(12.8)	13.9	(12.4)	12.9	(9.2)	11.0	(8.3)	12.7	(9.3)	32.8	(37.6)
140	Systemic sclerosis	M34	1,675	7.8	(6.5)	4.5	8.7	(7.0)	4.5	7.0	(6.2)	4.8	1.2	5.3	(11.0)	8.0	(10.0)	10.7	(9.2)	11.8	(10.0)	12.9	(7.9)	15.6	(8.9)	35.7	(43.1)
141	Kyphosis, lordosis	M40	4,160	5.2	(5.1)	3.9	6.0	(5.6)	4.2	3.9	(4.3)	3.0	1.5	13.7	(13.1)	16.7	(15.4)	16.0	(14.1)	14.9	(12.9)	12.0	(9.9)	9.0	(7.6)	17.7	(27.0)
142	Scoliosis	M41	17,686	4.6	(4.9)	3.8	5.9	(5.6)	4.2	3.6	(4.1)	3.1	1.6	24.5	(17.4)	18.9	(15.3)	14.9	(13.3)	11.7	(11.3)	9.0	(9.0)	7.1	(7.2)	13.8	(26.5)
143	Spinal osteochondrosis	M42	8,034	5.1	(5.1)	3.8	5.8	(5.6)	4.0	4.0	(4.3)	3.4	1.5	14.1	(13.3)	17.9	(16.2)	16.8	(14.5)	14.8	(12.5)	11.1	(9.1)	8.9	(7.3)	16.5	(27.1)
144	Other deforming dorsopathies	M43	23,756	6.2	(5.0)	4.2	7.1	(5.4)	4.3	5.1	(4.3)	3.7	1.4	10.1	(15.1)	13.4	(15.6)	14.0	(13.8)	13.7	(11.5)	12.4	(9.0)	11.5	(7.8)	24.8	(27.1)
145	Other inflammatory spondylopathies	M46	7,086	6.1	(5.5)	4.1	7.0	(6.0)	4.4	4.9	(4.8)	3.5	1.4	8.6	(9.2)	12.9	(13.2)	15.7	(14.4)	14.9	(13.2)	13.4	(10.2)	10.6	(8.0)	23.9	(31.7)
146	Spondylosis	M47	61,999	6.8	(5.4)	4.2	7.5	(5.8)	4.3	5.8	(4.6)	3.8	1.3	6.0	(9.9)	10.6	(14.2)	13.1	(14.0)	14.3	(12.6)	13.9	(10.4)	12.7	(8.8)	29.4	(30.2)
147	Other spondylopathies and spondylopathies in diseases classified elsewhere	M48, M49	50,805	7.7	(5.7)	4.3	8.3	(6.1)	4.3	6.7	(4.8)	3.9	1.2	3.3	(6.8)	7.7	(14.0)	10.5	(13.2)	13.0	(12.5)	14.2	(11.2)	14.4	(9.3)	36.9	(33.0)
148	Cervical disc disorders	M50	11,476	5.4	(5.3)	3.7	6.4	(6.0)	4.0	4.2	(4.3)	3.1	1.5	9.8	(10.7)	15.0	(13.9)	16.9	(14.0)	15.2	(13.5)	12.9	(10.9)	10.4	(8.1)	19.8	(28.8)
149	Other intervertebral disc disorders	M51	40,161	5.4	(5.1)	3.9	6.4	(5.7)	4.2	4.2	(4.2)	3.3	1.5	12.8	(14.0)	16.6	(16.0)	15.9	(14.1)	14.1	(11.5)	11.6	(9.1)	9.9	(7.7)	19.2	(27.7)
150	Other dorsopathies, not elsewhere classified	M53	7,246	5.5	(5.4)	3.9	6.6	(6.0)	4.3	4.6	(4.4)	3.1	1.5	11.0	(11.9)	15.4	(14.5)	16.4	(14.0)	14.2	(11.3)	12.2	(9.8)	10.5	(8.2)	20.2	(30.3)
151	Dorsalgia	M54	40,780	5.7	(5.3)	4.1	6.7	(5.9)	4.5	4.4	(4.2)	3.4	1.5	11.9	(12.9)	15.6	(14.7)	16.2	(14.2)	14.0	(11.5)	11.9	(9.5)	10.1	(7.9)	20.3	(29.4)
152	Soft tissue disorders	M60–M63, except M60.0	13,422	5.3	(5.4)	3.9	6.4	(6.2)	4.4	4.0	(4.2)	2.9	1.6	14.3	(13.0)	17.2	(14.7)	16.3	(13.4)	13.6	(11.0)	10.9	(9.0)	9.6	(8.2)	18.2	(30.7)
153	Synovitis and tenosynovitis	M65	19,104	4.8	(4.5)	3.4	5.8	(5.1)	3.8	4.0	(3.9)	2.9	1.4	12.9	(14.0)	17.7	(17.8)	17.5	(16.4)	14.4	(12.8)	11.7	(9.9)	9.6	(7.9)	16.2	(21.3)
154	Disorders of synovium and tendon	M66–68	19,669	4.0	(4.3)	3.1	4.9	(4.9)	3.6	3.5	(3.7)	2.5	1.4	19.8	(17.6)	21.0	(18.7)	17.7	(16.1)	13.2	(12.2)	9.7	(9.2)	7.0	(6.9)	11.5	(19.4)
155	Soft tissue disorders related to use, overuse and pressure	M70	11,090	5.5	(4.9)	3.9	6.8	(5.5)	4.2	4.0	(4.1)	2.8	1.7	13.0	(14.8)	15.9	(16.1)	15.5	(14.6)	14.0	(12.0)	12.0	(9.7)	10.0	(7.6)	19.6	(25.1)
156	Fibroblastic disorders	M72	43,600	5.0	(4.0)	3.5	5.7	(4.5)	3.8	4.1	(3.4)	3.0	1.4	14.1	(20.9)	16.2	(19.6)	15.5	(14.9)	14.5	(12.2)	11.7	(8.6)	9.6	(6.2)	18.2	(17.6)
157	Shoulder lesions	M75	58,112	4.6	(4.3)	3.3	5.4	(4.8)	3.7	3.7	(3.5)	2.7	1.5	16.2	(19.0)	18.4	(18.5)	16.4	(14.5)	14.1	(12.0)	11.0	(9.0)	8.8	(7.0)	15.0	(19.9)
158	Enthesopathies of lower limb, excluding foot	M76	11,223	3.9	(4.3)	3.1	5.2	(5.0)	3.8	3.1	(3.7)	2.3	1.7	22.6	(19.4)	21.5	(18.4)	17.3	(15.3)	13.1	(12.1)	8.4	(8.0)	6.3	(6.4)	10.8	(20.5)
159	Other enthesopathies	M77	10,500	4.5	(4.5)	3.2	5.4	(5.1)	3.6	3.4	(3.8)	2.7	1.6	15.0	(15.9)	18.8	(17.5)	17.0	(14.5)	15.3	(13.2)	11.0	(9.2)	8.9	(7.9)	13.9	(21.8)
160	Rheumatism, unspecified	M790	6,852	7.0	(6.1)	4.2	7.4	(6.3)	4.3	6.0	(5.4)	4.1	1.2	5.4	(8.5)	9.6	(11.4)	13.1	(13.1)	13.9	(11.0)	13.4	(9.0)	13.8	(9.5)	30.8	(37.6)
161	Myalgia	M791	10,168	6.1	(5.5)	4.3	7.1	(6.1)	4.6	4.8	(4.5)	3.6	1.5	10.6	(12.8)	13.6	(14.0)	14.5	(13.5)	14.1	(11.0)	13.3	(9.8)	10.1	(7.4)	23.7	(31.4)
162	Other soft tissue disorders, not elsewhere classified	M792– M794; M798–M799	7,939	5.6	(5.3)	4.2	7.0	(6.1)	4.6	3.4	(4.0)	2.8	2.0	13.1	(14.2)	16.5	(15.8)	15.1	(13.0)	13.3	(10.9)	12.0	(9.6)	10.5	(7.9)	19.5	(28.6)
163	Other soft tissue disorders, not elsewhere classified: pain in limb	M796	22,201	5.3	(4.9)	4.0	6.6	(5.6)	4.4	4.0	(4.0)	3.1	1.6	14.7	(15.1)	16.9	(16.2)	15.6	(14.0)	13.9	(11.8)	11.2	(9.2)	9.3	(7.4)	18.4	(26.1)
164	Fibromyalgia	M797	3,399	6.9	(6.7)	4.0	7.5	(7.1)	4.3	6.3	(6.1)	3.2	1.2	3.6	(6.0)	9.2	(8.2)	12.4	(10.3)	15.0	(11.5)	15.2	(8.3)	14.7	(10.4)	29.8	(45.3)
165	Osteoporosis ^c^	M80–M81	158,813	6.4	(6.0)	3.9	6.9	(6.5)	3.9	5.3	(5.1)	3.5	1.3	6.1	(4.8)	11.1	(10.4)	13.5	(12.1)	14.4	(12.2)	14.1	(12.4)	13.0	(12.0)	27.8	(36.0)
166	Osteoporosis in diseases classified elsewhere	M82	1,007	8.4	(7.0)	4.4	9.0	(7.5)	4.2	6.6	(5.2)	3.2	1.4	2.0	(2.5)	5.7	(8.8)	9.3	(9.4)	12.5	(13.0)	13.4	(9.5)	16.8	(10.5)	40.2	(46.3)
167	Adult osteomalacia and other disorders of bone density and structure	M83, M85, except M833	43,271	6.0	(5.0)	3.8	6.9	(5.7)	4.1	4.7	(4.1)	3.3	1.5	7.7	(11.9)	12.9	(16.3)	15.2	(15.1)	14.6	(12.1)	13.6	(10.0)	11.6	(8.0)	24.4	(26.6)
168	Disorders of continuity of bone	M84	1,865	5.3	(5.1)	4.1	6.5	(6.0)	4.5	4.3	(3.7)	4.0	1.5	19.0	(16.1)	16.7	(14.9)	13.4	(12.8)	12.9	(11.6)	10.7	(8.9)	8.3	(7.1)	19.0	(28.6)
169	Other osteopathies	M86–M90	24,251	6.3	(5.2)	4.2	7.2	(5.7)	4.3	4.9	(4.2)	3.6	1.5	9.1	(13.5)	12.8	(15.2)	13.8	(13.2)	13.9	(11.8)	13.0	(9.6)	11.7	(8.1)	25.6	(28.5)
170	Other disorders of the musculoskeletal system and connective tissue	M95–M99	30,038	5.4	(5.1)	4.1	6.5	(5.6)	4.5	3.9	(4.1)	3.3	1.7	16.4	(16.4)	16.9	(15.9)	14.9	(13.1)	12.8	(10.7)	11.0	(8.9)	9.2	(7.2)	18.8	(27.9)
	**N–Diseases of the genitourinary system**	**N18**	**20,162**	**8.8**	**(6.9)**	**4.5**	**9.4**	**(7.3)**	**4.5**	**7.5**	**(6.1)**	**4.2**	**1.2**	**2.1**	**(5.2)**	**5.4**	**(8.8)**	**8.9**	**(10.1)**	**11.5**	**(10.0)**	**13.6**	**(10.3)**	**14.9**	**(9.3)**	**43.7**	**(46.2)**
171	Chronic renal failure (CRF) ^c^	N18	20,162	8.8	(6.9)	4.5	9.4	(7.3)	4.5	7.5	(6.1)	4.2	1.2	2.1	(5.2)	5.4	(8.8)	8.9	(10.1)	11.5	(10.0)	13.6	(10.3)	14.9	(9.3)	43.7	(46.2)
	Q–Congenital malformations, deformations and chromosomal abnormalities	Q00–Q56; Q60–Q99	124,898	4.0	(4.3)	3.3	5.1	(5.0)	3.8	3.0	(3.5)	2.4	1.7	24.5	(20.8)	21.2	(18.1)	15.8	(14.0)	11.8	(10.8)	8.7	(8.5)	6.7	(6.8)	11.2	(21.2)
172	Congenital malformations: of the nervous, circulatory and respiratory systems, cleft palate and cleft lip, urinary tract, bones and muscles, other and chromosomal abnormalities not elsewhere classified	Q00–Q07; Q20–Q37; Q60–Q99	85,534	4.1	(4.5)	3.3	5.1	(5.3)	3.7	3.1	(3.7)	2.5	1.7	22.3	(18.3)	20.9	(17.4)	16.4	(14.3)	12.2	(11.1)	9.1	(8.8)	7.1	(7.3)	12.0	(22.9)
173	Congenital malformations of eye, ear, face and neck	Q10–Q18	19,689	3.4	(3.9)	2.8	4.4	(4.6)	3.5	2.7	(3.3)	2.1	1.7	29.9	(24.3)	23.4	(19.7)	15.4	(14.0)	10.9	(10.9)	7.4	(7.9)	5.3	(6.2)	7.8	(17.0)
174	Other congenital malformations of the digestive system	Q38–Q45	6,481	5.9	(5.0)	4.2	6.9	(5.7)	4.5	4.1	(4.0)	3.2	1.7	12.8	(14.7)	16.1	(16.9)	13.9	(12.9)	13.1	(11.4)	11.9	(9.6)	10.4	(7.8)	21.6	(26.8)
175	Congenital malformations of the sexual organs	Q50–Q56	16,192	3.5	(3.8)	2.9	4.5	(4.5)	3.4	2.5	(3.0)	2.0	1.8	29.1	(25.3)	22.6	(19.9)	15.2	(14.1)	10.9	(10.9)	8.0	(8.1)	5.4	(5.7)	8.9	(15.9)
	**F–Mental and behavioral disorders**	**F00–99**	**683,194**	**4.8**	**(4.5)**	**3.5**	**5.3**	**(5.0)**	**3.7**	**3.9**	**(3.8)**	**3.0**	**1.4**	**16.4**	**(16.5)**	**17.6**	**(16.8)**	**16.0**	**(14.7)**	**13.6**	**(12.2)**	**11.2**	**(9.8)**	**8.9**	**(7.6)**	**16.3**	**(22.4)**
176	Dementia ^c^	F00, G30, F01, F02.0, F03.9, G31.8B, G31.8E, G31.9, G31.0B	36,803	7.4	(6.8)	3.8	7.5	(6.9)	3.9	7.2	(6.6)	3.7	1.0	2.0	(1.3)	6.0	(5.6)	10.2	(11.9)	13.8	(13.1)	15.9	(14.3)	15.5	(9.2)	36.6	(44.5)
177	Organic, including symptomatic, mental disorders	F04–F09	26,430	8.0	(7.1)	4.4	8.3	(7.4)	4.4	7.0	(6.0)	4.2	1.2	3.6	(4.1)	6.6	(7.0)	9.8	(9.4)	12.3	(10.4)	14.2	(11.1)	14.6	(10.1)	38.9	(47.9)
178	Mental and behavioral disorders due to use of alcohol	F10	59,143	5.9	(5.9)	3.9	6.1	(6.2)	3.9	6.1	(5.4)	3.9	1.0	10.9	(10.3)	12.4	(10.7)	13.7	(11.2)	13.9	(11.2)	13.1	(10.6)	11.9	(9.6)	24.2	(36.3)
179	Mental and behavioral disorders due to psychoactive substance use	F11–F19	53,669	5.8	(6.0)	4.0	6.0	(6.5)	4.0	5.7	(5.2)	3.9	1.1	10.6	(8.3)	14.3	(11.3)	15.2	(12.1)	14.4	(11.6)	12.8	(10.6)	11.0	(9.3)	21.7	(36.9)
180	Schizophrenia ^c^	F20	29,422	5.9	(6.1)	3.7	6.1	(6.4)	3.8	5.0	(5.0)	3.2	1.2	5.1	(4.0)	12.3	(9.8)	16.2	(13.2)	16.3	(13.6)	14.7	(12.3)	12.6	(11.0)	22.8	(36.0)
181	Schizotypal and delusional disorders	F21–F29	39,694	6.1	(6.2)	3.8	6.5	(6.7)	3.9	5.0	(4.9)	3.2	1.3	5.1	(4.2)	11.3	(9.2)	15.2	(12.5)	15.8	(13.0)	14.8	(12.3)	12.9	(10.9)	24.8	(38.0)
182	Bipolar affective disorder ^c^	F30–F31	22,669	6.9	(6.5)	4.0	7.6	(7.2)	4.1	5.6	(5.2)	3.5	1.3	3.2	(3.0)	9.0	(8.3)	13.5	(11.9)	15.1	(12.6)	15.1	(12.1)	14.2	(11.1)	29.8	(41.0)
183	Depression ^c^	F32, F33, F34.1, F06.32	454,933	5.1	(4.8)	3.6	5.9	(5.3)	3.8	4.1	(3.9)	3.0	1.5	13.4	(14.2)	16.2	(15.9)	15.7	(14.6)	14.1	(12.6)	12.0	(10.2)	9.8	(8.1)	18.6	(24.4)
184	Mood (affective) disorders	F340, F348–F349, F38–F39	6,887	7.3	(7.0)	4.3	7.9	(7.6)	4.4	6.0	(5.7)	3.8	1.3	3.9	(3.1)	8.6	(7.3)	11.6	(9.5)	14.1	(11.7)	14.9	(11.4)	14.2	(10.3)	32.7	(46.7)
185	Phobic anxiety disorders	F40	14,324	5.2	(6.1)	3.3	5.7	(6.6)	3.4	4.0	(4.7)	2.8	1.4	7.8	(5.1)	14.4	(10.5)	17.0	(13.0)	16.1	(12.7)	13.9	(11.1)	11.3	(9.9)	19.6	(37.7)
186	Other anxiety disorders	F41	38,079	6.1	(6.5)	3.9	6.8	(7.1)	4.2	4.6	(5.1)	3.3	1.5	6.2	(4.9)	11.9	(9.2)	15.8	(12.1)	15.5	(12.1)	14.1	(11.2)	12.5	(10.2)	24.0	(40.4)
187	Obsessive compulsive disorder (OCD) ^c^	F42	10,062	5.0	(5.9)	3.3	5.9	(6.9)	3.6	3.8	(4.4)	2.5	1.6	9.4	(6.3)	15.7	(10.9)	18.0	(12.9)	16.1	(13.5)	13.1	(11.3)	10.4	(9.9)	17.3	(35.3)
188	Post-traumatic stress disorder	F431	16,055	5.2	(5.6)	3.3	5.6	(6.1)	3.6	4.9	(5.1)	3.1	1.1	7.9	(7.3)	13.9	(11.5)	17.3	(14.4)	16.1	(13.1)	14.2	(12.2)	11.5	(9.9)	19.0	(31.7)
189	Reactions to severe stress and adjustment disorders	F432–F439	61,701	5.2	(5.9)	3.6	5.8	(6.6)	3.9	4.5	(4.7)	3.2	1.3	10.4	(7.5)	16.0	(12.0)	17.2	(13.3)	15.3	(12.3)	12.7	(10.8)	10.4	(9.3)	18.0	(34.7)
190	Dissociative (conversion) disorders, somatoform disorders and other neurotic disorders	F44, F45, F48	21,420	6.4	(6.4)	4.3	7.3	(7.2)	4.6	5.0	(5.1)	3.4	1.5	8.0	(7.2)	12.0	(10.3)	14.5	(11.6)	14.0	(11.0)	13.6	(10.6)	12.4	(9.7)	25.5	(39.6)
191	Eating disorders	F50	7,751	4.5	(7.0)	3.3	5.8	(8.2)	3.7	4.1	(5.6)	3.1	1.4	16.0	(6.5)	18.9	(8.3)	17.6	(10.0)	14.0	(10.2)	10.7	(10.9)	9.4	(9.8)	13.4	(44.2)
192	Behavioral syndromes associated with physiological disturbances and physical factors	F51–F59	6,163	4.5	(5.3)	3.6	6.0	(6.6)	4.3	3.3	(4.0)	2.6	1.8	19.1	(14.4)	19.6	(15.0)	16.3	(13.0)	13.7	(11.7)	10.1	(9.1)	7.5	(7.1)	13.9	(29.8)
193	Emotionally unstable personality disorder	F603	21,848	6.4	(7.2)	3.8	6.7	(7.7)	3.9	5.0	(5.4)	3.2	1.3	4.2	(3.0)	10.2	(7.5)	14.2	(10.1)	15.2	(10.4)	15.2	(11.1)	14.0	(11.3)	27.0	(46.5)
194	Specific personality disorders	F602, F604–F609	50,415	5.9	(6.3)	3.8	6.3	(6.8)	3.9	4.5	(4.8)	3.0	1.4	6.6	(5.0)	12.5	(9.8)	15.5	(12.1)	15.6	(12.4)	14.7	(12.0)	12.1	(10.0)	23.0	(38.6)
195	Disorders of adult personality and behavior	F61–F69	17,533	6.2	(6.7)	3.9	6.6	(7.2)	4.0	5.0	(5.3)	3.3	1.3	6.0	(4.8)	10.8	(8.4)	14.1	(11.1)	15.4	(11.9)	14.8	(11.6)	13.3	(10.4)	25.5	(41.9)
196	Mental retardation	F70–F79	13,822	5.3	(5.6)	3.3	5.4	(5.6)	3.3	5.9	(5.7)	4.2	0.9	6.4	(5.1)	14.1	(11.2)	16.7	(14.0)	17.0	(14.8)	14.5	(13.1)	11.6	(10.7)	19.7	(31.1)
197	Disorders of psychological development	F80–F89	9,911	4.4	(5.8)	2.9	4.7	(6.0)	3.1	4.0	(4.4)	2.8	1.2	11.1	(5.1)	19.5	(10.9)	20.0	(14.3)	15.9	(13.6)	12.1	(12.3)	8.6	(9.2)	12.8	(34.5)
198	Hyperkinetic disorders (ADHD) ^c^	F90	42,908	4.0	(5.5)	3.0	4.2	(5.9)	3.1	4.8	(4.8)	3.3	0.9	22.1	(10.4)	19.5	(12.8)	16.3	(12.7)	13.1	(12.5)	9.7	(10.5)	7.6	(9.4)	11.7	(31.6)
199	Behavioral and emotional disorders with onset usually occurring in childhood and adolescence	F91–F99	39,602	5.9	(6.5)	3.9	6.3	(7.0)	4.0	5.1	(5.2)	3.4	1.2	7.4	(5.0)	13.4	(9.6)	16.0	(12.0)	15.1	(11.8)	13.9	(11.3)	11.6	(9.8)	22.6	(40.5)
	**Having one or more chronic conditions**		**2,989,441**	**3.4**	**(3.1)**	**2.8**	**4.1**	**(3.5)**	**3.2**	**2.6**	**(2.5)**	**2.2**	**1.6**	**31.6**	**(34.9)**	**21.0**	**(20.9)**	**15.2**	**(14.1)**	**10.7**	**(9.4)**	**7.6**	**(6.4)**	**5.4**	**(4.4)**	**8.4**	**(9.8)**
	Depression medicine ^c^ **	ATC: N06A	529,918	4.8	(4.4)	3.7	5.6	(4.8)	3.9	3.8	(3.6)	3.1	1.5	13.9	(14.8)	15.5	(15.2)	14.7	(13.6)	13.2	(11.6)	11.2	(9.3)	9.2	(7.4)	17.4	(22.2)
	Antipsychotic medicine ^c^ **	ATC: N05A	138,625	5.5	(5.3)	3.8	5.8	(5.7)	3.9	4.9	(4.5)	3.4	1.2	8.5	(7.9)	12.7	(11.5)	14.8	(13.3)	14.5	(12.7)	13.3	(11.4)	11.4	(9.6)	21.4	(30.2)
	Indication prescribed anxiety medicine ^c^ **	All prescrib. w.indication codes 163 (for anxiety) or 371 (for anxiety, addictive)	102,568	4.9	(4.7)	3.8	5.6	(5.2)	4.1	3.7	(3.7)	3.2	1.5	13.2	(12.7)	15.0	(13.8)	14.6	(13.1)	13.0	(11.3)	11.2	(9.5)	9.1	(7.8)	17.1	(24.8)
	Heart failure medication ^c^ **	ATC: C01AA05, C03, C07 or C09A with indication code 430 (for heart failure)	7,468	8.0	(6.4)	4.1	8.4	(6.6)	4.2	7.0	(5.5)	3.6	1.2	1.6	(3.9)	4.7	(8.5)	8.9	(10.1)	12.7	(12.2)	15.3	(11.2)	14.8	(10.0)	41.8	(42.3)
	Ischemic heart medication ^c^ **	ATC: C01A, C01B, C01D, C01E.	129,484	7.4	(5.6)	4.1	7.8	(5.9)	4.1	6.4	(4.8)	3.8	1.2	3.2	(7.3)	6.6	(9.3)	10.4	(11.0)	13.1	(11.4)	14.9	(10.4)	14.6	(8.9)	36.0	(34.9)
	**All five types of the medicine above**		**688,006**	**5.1**	**(4.4)**	**3.7**	**5.7**	**(4.8)**	**3.9**	**4.1**	**(3.6)**	**3.2**	**1.4**	**12.3**	**(14.1)**	**14.4**	**(14.9)**	**14.4**	**(13.7)**	**13.3**	**(11.7)**	**11.8**	**(9.7)**	**9.9**	**(7.7)**	**19.2**	**(22.4)**
	**Total population**		**4,555,439**	**2.2**	**(2.2)**	**2.8**	**3.1**	**(2.6)**	**3.3**	**1.6**	**(1.7)**	**2.1**	**1.6**	**20.4**	**(19.9)**	**13.6**	**(13.2)**	**9.8**	**(9.6)**	**6.9**	**(6.7)**	**4.9**	**(4.8)**	**3.5**	**(3.4)**	**5.5**	**(8.0)**
	**Extra**																										
	Ischemic Heart Diseases	I05-I06; I11-I13; I20-I28; I30-I52	315,901	6.8	(5.2)	3.8	7.5	(5.7)	3.9	5.6	(4.4)	3.4	1.3	4.4	(11.7)	8.0	(13.6)	11.8	(13.7)	14.2	(12.3)	15.0	(10.7)	14.3	(9.0)	32.3	(29.1)
	Arthritis	M01-M03; M5-M9; M7-M14; M15-M20; M45	505,792	5.4	(4.3)	3.6	6.1	(4.7)	3.8	4.3	(3.6)	3.1	1.4	10.7	(17.0)	14.8	(18.7)	15.5	(15.6)	14.6	(12.5)	12.9	(9.5)	10.8	(7.2)	20.7	(19.4)
	Arthrosis	M15-M19	338,166	5.6	(4.3)	3.7	6.3	(4.7)	3.8	4.7	(3.7)	3.3	1.3	9.5	(16.0)	13.6	(18.2)	15.1	(15.8)	14.7	(12.9)	13.3	(9.7)	11.4	(7.3)	22.4	(20.0)
	Back conditions	M32-34;M41-M43;M46-49;M50-51;M53-M54	212,948	5.7	(4.8)	4.0	6.6	(5.3)	4.2	4.5	(4.0)	3.4	1.5	12.4	(16.3)	14.8	(16.0)	14.8	(14.0)	13.7	(11.6)	12.1	(9.3)	10.4	(7.6)	21.8	(25.3)
	Overweight	E66	220,928	3.9	(4.3)	3.7	5.1	(4.8)	4.1	2.5	(3.3)	2.9	2.0	12.4	(14.2)	14.8	(12.6)	14.8	(11.6)	13.7	(9.7)	12.1	(8.2)	10.4	(6.9)	21.8	(23.1)
	Endometriosis	N80	29,190	3.0	(2.0)	2.9	4.0	n/a	3.5	1.9	n/a	2.1	2.1	21.4	(9.1)	17.2	(7.5)	13.4	(5.9)	9.7	(4.4)	7.1	(11.0)	4.9	(2.5)	7.1	(7.2)

Gender and age-standardised estimates (Std.) are in brackets.

ICD-10 International Statistical Classification of Diseases, 10^th^ Revision

^c^ = complex defined conditions, see reference for further details [65].

* Overall population frequencies and prevalence adapted from Hvidberg et al. 2019 [12].

** 2-year prevalence. n/a: not available

In total, 47 conditions had a mean of 7 or more chronic conditions. Among the 50 chronic conditions with the highest NCCs, 22 conditions were found within disease group I (diseases of the circulatory system) and seven conditions within disease group M (diseases of the musculoskeletal system and connective tissue). The twenty conditions with the highest mean NCCs were: bronchitis (J40-J42, mean = 9.8), AMI complex (I23-I24, mean = 9.3), heart failure (I11-I13, mean = 8.8), CRF (N18, mean = 8.8), chronic ischemic heart disease (I25, mean = 8.8), sequelae of cerebrovascular disease (I69, mean = 8.8), atherosclerosis (I70, mean = 8.7), emphysema (J43, mean = 8.6), osteoporosis in diseases classified elsewhere (M82, mean = 8.4), complications and ill-defined descriptions of heart disease (I51-I52, mean = 8.3), AMI (I21-I22, mean = 8.1), other anaemias (D64, mean = 8.1), aplastic and other anaemias (D60-D63, mean = 8.1), other forms of heart disease (I31-I43, mean = 8.0), aortic (I05-I06, mean = 8.0), organic, including symptomatic, mental disorders (F04-F09, mean = 8.0), other diseases of the respiratory system (J60-J84, mean = 7.9), aortic aneurysm and aortic dissection (I71, mean = 7.9), atrioventricular and left bundle branch block (I44, mean = 7.9), and ischemic heart diseases (I20-I25, mean = 7.9).

The largest differences in means between individuals with no educational attainment and individuals with higher educational attainment were found within disease group J (ratio = 1.8)–meaning that individuals with no education had a nearly two times higher mean NCC than individuals with higher educational attainment. The remaining disease group ratios were as follows: Q (ratio = 1.7), B (ratio = 1.7), D (ratio = 1.6), L (ratio = 1.6), K (ratio = 1.5), M (ratio = 1.5), C (ratio = 1.4), H (ratio = 1.4), I (ratio = 1.4), F (ratio = 1.4), E (ratio = 1.3), and N (ratio = 1.2).

Among the 50 chronic conditions with the largest differences in means between individuals with no educational attainment and individuals with higher educational attainment, 13 conditions were found within disease group M, seven conditions within disease group H, six conditions within disease group E, four within disease group I and Q, three within disease group C, J and K, two within D and G, and one within F, L, and B. The twenty conditions with the largest differences in means according to educational attainment were: other soft tissue disorders (M792-M79, ratio = 2.0), cystic fibrosis (E84, ratio = 2.0), behavioural syndromes (F51-F59, ratio = 1.8), disorders of ocular muscles (H49-H52, ratio = 1.8), haemorrhoids (I84, ratio = 1.8), ulcerative colitis (K51, ratio = 1.8), congenital malformations of the sexual organs (Q50_Q56, ratio = 1.8), allergy (J30, ratio = 1.8), disorders of the choroid and retina (H31-H32, ratio = 1.7), coagulation defects (D65-D69, ratio = 1.7), rheumatoid arthritis (M05-M09, ratio = 1.7), soft tissue arthritis (M70, ratio = 1.7), other congenital malformations of the digestive system (Q38-Q45, ratio = 1.7), disorders of the vitreous body and globe (H43-H45, ratio = 1.7), thyrotoxicosis (E05, ratio = 1.7), disorders of trigeminal nerve and facial nerve disorders (G50-G51, ratio = 1.7), enthesopathies of lower limb (M76, ratio = 1.7), in situ neoplasms (D00-D09, ratio = 1.7), other disorders of the musculoskeletal system (M95-M99, ratio = 1.7), IBS (K58, ratio = 1.7), and hepatitis (B18, ratio = 1.7).

Finally, endometriosis (N80) and overweight (E66), which were not defined as *chronic* conditions, had the highest- and third-highest ratios (2.1 and 2.0) among individuals with no educational attainment and individuals with higher educational attainment.

## Discussion

To the best of the authors’ knowledge, the present study is the first and most comprehensive register-based attempt to estimate the multimorbidity disease burden of chronic conditions from a nationwide population using a comparable, uniform methodology across a larger number of chronic conditions. The result section only shows a small fraction of possible examples for some multimorbid data that can be extracted from the catalogues or tables. For instance, and in summary, we investigated the mean NCCs and associations of 14 disease groups, 29 common chronic conditions (to provide the reader with an overview), and 199 chronic conditions for the entire Danish adult population, including differences in sex, age groups, and educational attainments. Our study showed that most people in the Danish population had one or more chronic conditions and that multimorbidity is common. This is in line with previous national and international research [7, 12, 73]. The overall mean of NCCs for the population was 2.2 and 3.4 for patients with one or more chronic conditions. The mean NCCs increased by age, and women had a higher mean of chronic conditions than men.

Furthermore, we found a social gradient in the mean of NCCs–with individuals with lower educational attainments having a higher mean. For instance, the largest difference in means of NCCs between individuals with no education and individuals with higher educational attainment was found in disease group J (diseases of the respiratory system). This, increasing NCC by age, higher rates in women and increasing rates of NCC with lower educational attainment are also following earlier studies [7]. We found large variations in the mean of NCCs between conditions ranging from a mean of 3.3 to a mean of 9.8 in chronic conditions. The diseases with the highest NCC were overall found within disease groups N–diseases of the genitourinary system (mean = 8.8), D–in situ and benign neoplasms (6.5), K–diseases in the digestive system (5.7), and H–a disease of the eye and the ear (5.6). The most common chronic conditions are also complicated by high mean rates of multimorbidity, including hypertensive diseases, respiratory allergy, chronic lower respiratory diseases, type 2 diabetes, and depression. Persons with heart failure, ischemic heart diseases, angina pectoris, and stroke had the highest NCC, all with a mean above 6.5 chronic conditions but less hypertension. Furthermore, individuals with COPD, cataracts, osteoporosis, type 2 diabetes, anxiety disorders, and inflammatory polyartropathies had high NCCs–above six chronic conditions. Most individuals with one of the 29 common conditions had above five NCCs. Other conditions are characterised by little multimorbidity rates, such as type 1 diabetes, tinnitus, and other headache syndromes, most likely because the conditions are typically diagnosed in younger patients [74].

When looking into some examples of associations between the chronic conditions, we found that conditions, not surprisingly, seem to be particularly associated with other diseases within the same disease groups; for example, chronic lower respiratory diseases were highly associated with asthma and respiratory allergy. However, conditions also often transcend disease groups. For instance, hypertensive diseases were also associated with type 2 diabetes and depression, which might be explained by the high prevalence of the three conditions. We found that prevalence rates of depression varied between 14.5% to 51.0% in the 29 conditions–following other evidence showing that depression is a common comorbidity to several chronic conditions [75, 76]. Further, type 2 diabetes is associated with ischaemic heart diseases–both common conditions and also linked to the same underlying pathology. This is consistent with common medical knowledge and another study by Breinholt et al. (2017) looking at correlations of 15 chronic diseases. Six disease classes were identified here, and heart diseases, particularly hypertension, were associated with at least four other conditions [11]. Other prevalent conditions like arthritis, chronic lower respiratory diseases, depression, and overweight also transcended to other disease groups.

### The catalogue in summary—and future use

The above results, underlined, are not exhaustive but just a few of many possible data extractions. Hence, the main aim of this study was to provide a detailed off-the-shelf catalogue for others to explore their specific interests and needs. In summary, we provided nine comprehensive catalogues (Tables 1–3 and S2–S7 Tables) that can be used to explore how the *severity* and *associations* of multimorbidity are distributed, including differences in age, sex, and educational attainment across the 199 chronic conditions as described below:

We measured disease burden *severity* in terms of the crude mean NCCs. Here, the following tables provide overall *mean NCCs*, including differences in age groups and sex: Table 1 by disease groups and medicines, S2 Table for all the 199 conditions, S3 Table by the 29 common conditions and overweight, and S7 Table show the raw mean NCCs of 199 conditions by age and sex in 14 categories for further detailed analysis. Moreover, Table 3 provides an overview of mean NCCs and prevalence for the 199 chronic conditions regarding the overall population and means by social equality measured by high and low educational attainments. Finally, the S6 Table shows the mean NCCs of the 199 chronic conditions by all five educational attainments.

While the means provide a crude estimate of severity, the following tables provide the *associational prevalence* of the chronic conditions. Table 2 shows the comorbidity prevalence between the 29 common conditions and overweight, and the S4 Table shows the correlational prevalence rates of the 199 chronic conditions, disease groups, medicines, and overweight by the 29 common conditions and overweight. Finally, the S5 Table shows the correlational prevalence rates of all 53,361 combinations between the 199 chronic conditions, disease groups, medicines, and overweight.

We see three main potential uses of the catalogue: First, it can support and inform on-the-floor health care specialists of possible multimorbidity concerns to be considered within treatments. Although knowledge about possible multimorbidity is not unknown within medical practice, healthcare systems worldwide are constructed to treat patients with single diseases [28]. This is a fact even though multimorbidity is the norm for 69.7 per cent of patients with a chronic disease or 45.7 per cent of the adult population within the present study (see S1 Table). In contrast, socioeconomic disparities within health behaviours like smoking, drinking, and exercise routines have long been used to *differentiate* treatments [77] and, for example, to a lesser extent, within the rehabilitation of cardiac diseases [78–80]. We propose that future treatments are, to a greater extent, also *differentiated* a prior due to the multimorbidity severity, disease associations, and clusters of common comorbidities, using evidence like the current catalogue. This will make future interventions more data-driven in real-world evidence and multimorbidity directly embedded in medical practice.

Second, we propose that the catalogues are also used to identify and prioritise diseases for treatment based on severity, related disease associations, and clusters of high severity conditions. However, as multimorbidity is only one facet of disease burden, prioritisation should be done in conjunction with other aspects of disease burden, including health-related quality of life [15], overall disease prevalence, socioeconomic characteristics [12], and socioeconomic disparities [9].

Third, we propose that knowledge of multimorbidity severity, chronic condition associations, and clusters of common comorbidities are also used by health care planners to model the future health care systems. We suggest that diseases are seen in a more holistic view, comprising clusters of conditions and that interventions are set up systemically to threaten known and firstly prevalent clusters of conditions; moreover, high severity, multimorbidity, and less prevalent conditions known to be costly with low patient outcomes should be addressed in specialised centres. We propose that the current catalogue is used further to identify relevant clusters of diseases within medical specialities. For instance, the detailed spreadsheet in the S5 Table provides aggregated, detailed data of multimorbidity for all 199 x 199 chronic conditions that can be used to identify clusters. As health care systems are currently mainly set up to treat single diseases, future health care planning needs to address and incorporate the real-world norm of multimorbidity.

### Strengths and limitations

One of the main strengths of this study is the data, e.i. the application of data from six nationwide, high-quality registers and the use of the total nationwide population. A second strength is the application of a uniform and comparable methodology as recommended by WHO and researchers [20, 52, 53, 81, 82], e.i. the use of medical ratified definitions and algorithms applied to the unique data and the high number of chronic conditions comprised within a single study. This enables reliable comparisons across an extraordinary number of conditions. A third strength is the identified variation in the means and types of comorbidities. For example, the prevalence of overweight differs within the same disease groups and across individual chronic conditions. Some conditions have a high prevalence of overweight within the same disease group (Schizophrenia); others do not (Dementia). This and similar information could prove crucial in planning future health care interventions across different diseases, targeting different issues dependent on disease. This detailed variation might be lost using classical statistical methods like latent class analysis, factor analysis, or correspondence analysis. However, as we provide detailed, raw descriptive data, the current study can be used to identify such detailed differences useful in concrete interventions.

There are, however, also some methodological limitations in the present study. One limitation concerns the methodological issue of defining ‘chronic’. Should ‘chronic’ be understood literally as ‘forever’, and should only ‘severe’ (not in the sense of high mean NCC) diseases be included as suggested by critics [83, 84]? These choices impact the size of the disease burden and include conditions. However, defining ‘severe’ possess some of the same issues as defining ‘chronic’. And defining chronic strictly as ‘forever’ would lead to the exclusion of many diseases, such as type 2 diabetes, some heart diseases, and cancers, broadly accepted as chronic diseases, as, in fact, many commonly perceived chronic conditions do not last forever. This was why earlier studies suggested a differentiated approach based on the previously mentioned four categorisations of chronicity or severity [15, 65, 66].

Moreover, labelling ICD-10 conditions as chronic or something else to not change the real-world disease burden but merely how we conceptualise it. However, the debate and varying severity highlight the complexity of chronic conditions. Notably, non-communicable diseases or long-term illness may be a better term than ‘chronic condition’, as ‘chronic’ is often understood ‘forever’ in everyday understanding, thus causing confusion or even reluctance.

Furthermore, our study showed a lower mean of NCCs for mental conditions like schizophrenia and ADHD and a lower prevalence of cardiovascular diseases like hypertension. There is, however, no clinical reason why mental conditions should have a mean and prevalence below the national averages for hypertension. This indicates that the comorbidities regarding, for example, heart diseases are underreported, and comorbidity could be even higher for conditions within disease group F. Other studies have already discussed similar limitations in underreports of diseases in register data [12, 65].

Finally, we recognize that there are other ways to measure disease burden *severity* than in terms of crude NCCs [15]. And that NCCs and associations are merely a proxy of severity regarding health-related quality of life, death risks, and disabilities and should not stand alone but be used with a range of different disease burden measures, including the earlier mentioned and health behaviours. Nevertheless, it is a way to provide indications and an overview of possible disease severities quickly.

### Implications for research

It is challenging to provide a broad overview of tendencies and clusters of conditions using solely raw descriptive statistics, particularly for a large number of conditions as in the present study. However, this study provides real-life, detailed estimates without statistical loss of data, particularly for ground health professionals, health care planners and clinicians who need to know their detailed disease population as a first step. Statistical methods, nonetheless, such as latent cluster analysis, factor analysis, multiple pattern analysis and artificial intelligence (AI), might provide a clearer overview as a second step. Although we recognize these statistical methods might have trouble identifying detailed variations and thus identifying subtle tendencies within data, they are still useful to supplement the current catalogue with broader, reduced statistical estimates for overall planning and research purposes. Hence, there is a need for future research to the use and develop consensus on more advanced methods and thereby identify broader clusters of comorbidities, and subtle, possible non-statistical tendencies across conditions and disease groups. Finally, future studies could also investigate how the classic statistical methods perform when identifying clusters and tendencies and comparing these.

## Conclusions

The current study provides an off-the-shelf catalogue of multimorbidity means, correlational disease prevalence showing the specific disease proportions for 199 different chronic conditions and groups of conditions by gender, age, and educational attainments, based on a complete nationwide population sample. The findings underline that multimorbidity is the rule and not the exception and that multimorbidity is a fundamental condition transcending disease burden and impacting all future treatments. However, current disease guidelines only include multimorbidity at a sporadic level. We argue that having reliable, real-world evidence of multimorbid disease burden is crucial for on-the-floor interventions and health care planners as provided within current study in a raw, descriptive format for others to use. We further suggest that future research identify multimorbidity clusters and investigate how these could best be identified. To the best of the authors’ knowledge, the present study provides the most comprehensive descriptive register study of the means of multimorbidity and correlational prevalence of chronic conditions.

## Supporting information

S1 Table Frequency table of the number of comorbidities. (DOC)

S2 Table Catalogue of mean NCCs and SDs of 199 conditions. Number of patients, overall mean number of comorbidities and means by sex and age in Denmark on 1 January 2013. Sorted by ICD-10 codes.
(DOC)

S3 Table Catalogue of mean NCCs and SDs of 29 common conditions and overweight. Number of patients, overall mean number of comorbidities and by sex and age in Denmark on 1 January 2013. Sorted by ICD-10 codes.
(DOC)

S4 Table Catalogue of correlational prevalence rates (per cent within conditions) of 199 chronic conditions, disease groups, medicines and overweight by common conditions in Denmark on 1 January 2013. Sorted by ICD-10 diagnosis.
(DOCX)

S5 Table Catalogue of correlational prevalence (per cent within conditions) and frequencies among the 199 chronic conditions, disease groups, common conditions, medicines and overweight in Denmark on 1st January 2013. Sorted by ICD-10 diagnosis.
(XLSX)

S6 Table Catalogue of mean NCCs and SDs of the 199 chronic conditions. Overall population estimates and by all educational levels in Denmark on 1 January 2013. Sorted by ICD-10 diagnosis.
(DOCX)

S7 Table Catalogue of raw means NCCs and SDs of 199 conditions. Means by age and sex groups in Denmark on 1 January 2013. Sorted by ICD-10 codes.
(DOC)
