# Supplementary material for: Catalogue of multimorbidity mean based severity and associational prevalence rates between 199+ chronic conditions—A nationwide register-based population study
Source: PLoS One. 2022 Sep 14;17(9):e0273850. doi: 10.1371/journal.pone.0273850 (PMC9473636; doi:10.1371/journal.pone.0273850)
Supplement: S1 Table — (DOC) [file pone.0273850.s001.doc]

***S1 Table.*** Frequency table of the number of comorbidities.

|  | **Frequency** | **Percent** | **Cumulative Frequency** | **Cumulative Percent** |
| --- | --- | --- | --- | --- |
| **0** | 1565998 | 34.38 | 1565998 | 34.38 |
| **1** | 906365 | 19.90 | 2472363 | 54.27 |
| **2** | 601767 | 13.21 | 3074130 | 67.48 |
| **3** | 435614 | 9.56 | 3509744 | 77.05 |
| **4** | 306882 | 6.74 | 3816626 | 83.78 |
| **5** | 218183 | 4.79 | 4034809 | 88.57 |
| **6** | 155685 | 3.42 | 4190494 | 91.99 |
| **7** | 109688 | 2.41 | 4300182 | 94.40 |
| **8** | 77563 | 1.70 | 4377745 | 96.10 |
| **9** | 54429 | 1.19 | 4432174 | 97.29 |
| **10** | 38209 | 0.84 | 4470383 | 98.13 |
| **11** | 26533 | 0.58 | 4496916 | 98.72 |
| **12** | 18352 | 0.40 | 4515268 | 99.12 |
| **13** | 12558 | 0.28 | 4527826 | 99.39 |
| **14** | 8844 | 0.19 | 4536670 | 99.59 |
| **15** | 6011 | 0.13 | 4542681 | 99.72 |
| **16** | 4150 | 0.09 | 4546831 | 99.81 |
| **17** | 2814 | 0.06 | 4549645 | 99.87 |
| **18** | 1922 | 0.04 | 4551567 | 99.92 |
| **19** | 1242 | 0.03 | 4552809 | 99.94 |
| **20** | 896 | 0.02 | 4553705 | 99.96 |
| **21** | 564 | 0.01 | 4554269 | 99.97 |
| **22** | 413 | 0.01 | 4554682 | 99.98 |
| **23** | 259 | 0.01 | 4554941 | 99.99 |
| **24** | 172 | 0.00 | 4555113 | 99.99 |
| **25** | 123 | 0.00 | 4555236 | 100.00 |
| **26** | 60 | 0.00 | 4555296 | 100.00 |
| **27** | 57 | 0.00 | 4555353 | 100.00 |
| **28** | 40 | 0.00 | 4555393 | 100.00 |
| **29** | 12 | 0.00 | 4555405 | 100.00 |
| **30** | 11 | 0.00 | 4555416 | 100.00 |
| **31** | 11 | 0.00 | 4555427 | 100.00 |
| **32** | 12 | 0.00 | 4555439 | 100.00 |
