# Supplementary material for: Catalogue of multimorbidity mean based severity and associational prevalence rates between 199+ chronic conditions—A nationwide register-based population study
Source: PLoS One. 2022 Sep 14;17(9):e0273850. doi: 10.1371/journal.pone.0273850 (PMC9473636; doi:10.1371/journal.pone.0273850)
Supplement: S2 Table — Number of patients, overall mean number of comorbidities and means by sex and age in Denmark on 1 January 2013. Sorted by ICD-10 codes. (DOC) [file pone.0273850.s002.doc]

***S2 Table.*** Catalogue of mean NCCs and SDs of 199 conditions: number of patients, overall mean number of comorbidities and means by sex and age in Denmark on 1 January 2013. Sorted by ICD-10 codes.

|  |  |  |  | **Overall NCC of population** | | | | | **Sex and age** | | | | | | | | | |
| --- | --- | --- | --- | --- | --- | --- | --- | --- | --- | --- | --- | --- | --- | --- | --- | --- | --- | --- |
| **No.** | **Name of condition** | **ICD-10 code / definition** | **Severity** |  | Means | | |  | Female | | Male | | Age 16-44 | | Age 45-74 | | Age 75+ | |
|  |  |  |  | *N* | *Raw* | *Std.* | *SD* | *Rank* | *Raw* | *SD* | *Raw* | *SD* | *Raw* | *SD* | *Raw* | *SD* | *Raw* | *SD* |
|  | **B – Viral hepatitis and human immunodeficiency virus [HIV] disease** | **B18, B20–B24** | **Cat I** | **8,500** | **4.4** | **(4.7)** | **3.5** | **206** | **4.3** | **3.6** | **4.5** | **3.5** | **3.6** | **3.1** | **5.0** | **3.7** | **8.5** | **4.2** |
| 1 | Chronic viral hepatitis | B18 | Cat IV | 4,584 | 5.0 | (5.3) | 3.8 | 180 | 4.9 | 3.9 | 5.2 | 3.7 | 4.0 | 3.4 | 5.8 | 3.9 | 9.9 | 4.6 |
| 2 | Human immunodeficiency virus [HIV] disease | B20–24 | Cat I | 4,229 | 3.9 | (4.2) | 3.2 | 219 | 3.8 | 3.4 | 4.0 | 3.1 | 3.2 | 2.8 | 4.4 | 3.3 | 7.4 | 3.3 |
|  | **C – Malignant neoplasms** | **C00–C99; D32–D33; D35.2–D35.4; D42–D44** |  | **229,331** | **5.4** | **(4.2)** | **3.6** | **147** | **5.4** | **3.6** | **5.5** | **3.6** | **3.2** | **2.4** | **5.0** | **3.4** | **7.0** | **3.8** |
| 3 | Malignant neoplasms of other and unspecified localizations | C00–C14; C30–C33; C37–C42; C45–C49; C69; C73–74; C754–C759 | Cat II | 20,557 | 5.9 | (4.7) | 3.7 | 111 | 5.9 | 3.8 | 5.8 | 3.7 | 3.6 | 2.4 | 5.7 | 3.6 | 7.7 | 3.9 |
| 4 | Malignant neoplasms of digestive organs | C15–C17; C22–C26 | Cat II | 4,839 | 6.8 | (5.4) | 4.0 | 58 | 6.9 | 4.0 | 6.7 | 4.0 | 4.5 | 2.9 | 6.4 | 3.9 | 8.1 | 4.1 |
| 5 | Malignant neoplasm of colon | C18 | Cat II | 18,826 | 6.4 | (4.5) | 3.9 | 72 | 6.4 | 3.8 | 6.3 | 3.9 | 3.7 | 2.7 | 5.6 | 3.6 | 7.5 | 3.9 |
| 6 | Malignant neoplasms of rectosigmoid junction, rectum, anus and anal canal | C19–C21 | Cat II | 10,680 | 5.8 | (4.4) | 3.5 | 117 | 5.8 | 3.6 | 5.7 | 3.5 | 3.6 | 2.4 | 5.2 | 3.3 | 6.9 | 3.7 |
| 7 | Malignant neoplasm of bronchus and lung | C34 | Cat II | 14,762 | 7.2 | (5.5) | 4.1 | 42 | 7.4 | 4.1 | 6.9 | 4.0 | 4.4 | 3.1 | 6.7 | 3.9 | 8.5 | 4.1 |
| 8 | Malignant melanoma of skin | C43 | Cat II | 19,636 | 4.4 | (3.5) | 3.2 | 205 | 4.2 | 3.1 | 4.6 | 3.3 | 2.6 | 1.8 | 4.2 | 3.0 | 6.9 | 3.7 |
| 9 | Other malignant neoplasms of skin | C44 | Cat IV | 15,597 | 5.8 | (3.9) | 3.8 | 114 | 5.9 | 3.8 | 5.8 | 3.8 | 2.9 | 2.3 | 5.0 | 3.5 | 7.1 | 3.9 |
| 10 | Malignant neoplasm of breast | C50 | Cat II | 50,687 | 5.2 | (4.3) | 3.4 | 168 | 5.2 | 3.4 | 5.8 | 3.7 | 3.2 | 2.2 | 4.7 | 3.1 | 7.0 | 3.7 |
| 11 | Malignant neoplasms of female genital organs | C51–C52; C56–C58 | Cat II | 7,245 | 5.3 | (4.2) | 3.4 | 160 | 5.3 | 3.4 | 5.6 | 1.9 | 3.4 | 2.5 | 5.0 | 3.2 | 7.1 | 3.7 |
| 12 | Malignant neoplasm of cervix uteri, corpus uteri and part unspecified | C53–C55 | Cat II | 11,608 | 5.0 | (2.0) | 3.3 | 181 | 5.0 | 3.3 | 0.0 | 0.0 | 3.2 | 2.3 | 4.7 | 3.1 | 6.8 | 3.5 |
| 13 | Malignant tumour of male genitalia | C60, C62–C63 | Cat II | 5,194 | 3.5 | (4.3) | 2.9 | 226 | 7.5 | 2.9 | 3.5 | 2.9 | 2.6 | 2.0 | 4.0 | 3.0 | 7.3 | 4.3 |
| 14 | Malignant neoplasm of prostate | C61 | Cat II | 26,697 | 5.5 | (4.7) | 3.5 | 139 | 7.3 | 4.9 | 5.5 | 3.5 | 3.5 | 2.0 | 4.8 | 3.1 | 6.6 | 3.7 |
| 15 | Malignant neoplasms of urinary tract | C64–C68 | Cat II | 10,319 | 6.2 | (4.6) | 3.7 | 83 | 6.6 | 3.8 | 6.1 | 3.7 | 3.6 | 2.4 | 5.7 | 3.5 | 7.3 | 3.8 |
| 16 | Brain cancer ^c^ | C71, C75.1–C75.3, D33.0–D33.2, D35.2–D35.4, D43.0–D43.2, D44.3–D44.5 (brain). C70, D32, D42 (brain membrane). C72, D33.3–D33.9, D43.3–D43.9 (cranial nerve, spinal cord) |  | 15,310 | 6.2 | (5.4) | 3.8 | 84 | 6.3 | 3.8 | 6.1 | 3.7 | 4.3 | 2.8 | 6.3 | 3.7 | 8.3 | 4.0 |
| 17 | Malignant neoplasms of ill-defined, secondary and unspecified sites, and of independent (primary) multiple sites | C76–C80, C97 | Cat II | 25,619 | 6.4 | (5.2) | 3.6 | 69 | 6.5 | 3.6 | 6.4 | 3.6 | 4.1 | 2.4 | 6.2 | 3.4 | 8.1 | 3.7 |
| 18 | Malignant neoplasms, stated or presumed to be primary, of lymphoid, haematopoietic and related tissue | C81–C96 | Cat II | 19,712 | 5.8 | (4.6) | 3.8 | 116 | 6.0 | 3.8 | 5.6 | 3.7 | 3.4 | 2.5 | 5.6 | 3.6 | 7.6 | 3.9 |
|  | **D – In situ and benign neoplasms, and neoplasms of uncertain or unknown behaviour and diseases of the blood and blood-forming organs and certain disorders involving the immune mechanism** | **D00–D09; D55–D59; D60–D67; D80–D89** |  | **116,560** | **6.5** | **(5.2)** | **4.3** | **66** | **6.2** | **4.3** | **7.0** | **4.4** | **3.6** | **2.8** | **6.5** | **4.2** | **8.9** | **4.2** |
| 19 | In situ neoplasms | D00–D09 | Cat II | 19,810 | 4.8 | (4.1) | 3.5 | 191 | 4.5 | 3.3 | 5.9 | 3.9 | 2.9 | 2.2 | 4.8 | 3.3 | 7.5 | 3.9 |
| 20 | Haemolytic anaemias | D55–D59 | Cat II | 3,055 | 5.5 | (5.2) | 4.2 | 141 | 5.3 | 4.1 | 5.9 | 4.3 | 3.3 | 2.6 | 6.6 | 4.3 | 9.0 | 4.0 |
| 21 | Aplastic and other anaemias | D60–D63 | Cat II | 14,918 | 8.1 | (6.2) | 4.7 | 14 | 7.7 | 4.7 | 8.7 | 4.5 | 4.2 | 3.3 | 7.7 | 4.6 | 9.7 | 4.3 |
| 22 | Other anaemias | D64 | Cat II | 46,613 | 8.1 | (6.1) | 4.6 | 13 | 7.8 | 4.6 | 8.6 | 4.5 | 4.4 | 3.5 | 7.7 | 4.6 | 9.3 | 4.2 |
| 23 | Coagulation defects, purpura and other haemorrhagic conditions | D65–D69 | Cat II | 25,376 | 5.6 | (5.3) | 4.2 | 134 | 5.3 | 4.1 | 6.0 | 4.2 | 3.5 | 2.8 | 6.5 | 4.2 | 9.4 | 4.4 |
| 24 | Other diseases of blood and blood-forming organs | D70–D77 | Cat II | 8,896 | 6.6 | (5.7) | 4.1 | 61 | 6.7 | 4.2 | 6.5 | 4.1 | 4.4 | 3.3 | 6.8 | 4.0 | 8.9 | 4.2 |
| 25 | Certain disorders involving the immune mechanism | D80–D89 | Cat III | 7,660 | 5.8 | (5.6) | 4.0 | 118 | 6.2 | 4.1 | 5.3 | 3.9 | 3.9 | 2.8 | 6.6 | 4.1 | 9.4 | 4.4 |
|  | **E – Endocrine, nutritional and metabolic diseases** | **E00–E14; E20–E29; E31–35; E70–E78; E84–E85; E88–E89** |  | **877,433** | **5.3** | (4.3) | **3.3** | **161** | **5.3** | **3.4** | **5.2** | **3.3** | **3.5** | **2.6** | **5.0** | **3.1** | **6.8** | **3.6** |
| 26 | Diseases of the thyroid ^c^ | E00–E04, E06, E07 | Cat III | 131,908 | 5.1 | (4.3) | 3.5 | 174 | 5.1 | 3.5 | 5.4 | 3.8 | 3.2 | 2.4 | 5.0 | 3.3 | 7.3 | 3.8 |
| 27 | Thyrotoxicosis ^c^ | E05 | Cat III | 41,374 | 5.3 | (4.2) | 3.6 | 159 | 5.2 | 3.5 | 5.7 | 4.0 | 3.1 | 2.3 | 5.1 | 3.4 | 7.3 | 3.8 |
| 28 | Diabetes type 1 ^c^ | E10 | Cat I | 23,062 | 4.7 | (4.7) | 3.2 | 197 | 5.1 | 3.4 | 4.3 | 3.0 | 3.2 | 2.5 | 5.5 | 3.2 | 7.4 | 3.4 |
| 29 | Diabetes type 2 ^c^ | E11 | Cat II | 242,177 | 6.2 | (5.1) | 3.6 | 86 | 6.4 | 3.6 | 6.0 | 3.5 | 4.2 | 3.0 | 5.9 | 3.4 | 7.5 | 3.7 |
| 30 | Diabetes others ^c^ | E12–E14 | Cat III | 1,117 | 6.3 | (5.4) | 4.4 | 80 | 6.3 | 4.7 | 6.3 | 4.1 | 3.3 | 2.5 | 7.0 | 4.3 | 9.2 | 4.5 |
| 31 | Disorders of other endocrine glands | E20–E35, except E30 | Cat III | 28,650 | 5.6 | (5.5) | 4.2 | 128 | 5.4 | 4.1 | 6.4 | 4.3 | 3.6 | 2.8 | 6.8 | 4.1 | 9.5 | 4.3 |
| 32 | Metabolic disorders | E70–E77; E79–E83; E85, E88–E89; | Cat II | 23,690 | 6.3 | (5.7) | 4.2 | 74 | 6.4 | 4.2 | 6.2 | 4.3 | 4.1 | 3.0 | 6.9 | 4.2 | 9.4 | 4.2 |
| 33 | Disturbances in lipoprotein circulation and other lipids ^c^ | E78 | Cat III | 652,242 | 5.6 | (4.8) | 3.4 | 131 | 5.8 | 3.4 | 5.4 | 3.3 | 4.4 | 3.1 | 5.2 | 3.2 | 6.9 | 3.6 |
| 34 | Cystic fibrosis ^c^ | E84 | Cat I | 947 | 4.2 | (4.9) | 3.3 | 211 | 4.3 | 3.4 | 4.0 | 3.0 | 3.3 | 2.4 | 5.8 | 4.0 | 8.1 | 3.9 |
|  | **G – Diseases of the nervous system** | **G00–G14; G20–G32; G35–G37; G40–47; G50–64; G70–73; G80–G83; G90–G99** |  | **561,054** | **5.1** | **(4.6)** | **3.6** | **172** | **5.1** | **3.6** | **5.1** | **3.6** | **3.5** | **2.6** | **5.2** | **3.5** | **7.9** | **3.9** |
| 35 | Inflammatory diseases of the central nervous system | G00–G09 | Cat I | 7,642 | 5.6 | (5.2) | 4.0 | 127 | 5.9 | 4.1 | 5.4 | 4.0 | 3.8 | 2.9 | 6.2 | 4.1 | 8.5 | 4.2 |
| 36 | Systemic atrophies primarily affecting the central nervous system and other degenerative diseases | G10–G14, G30–G32 | Cat I | 10,401 | 7.3 | (5.5) | 4.0 | 41 | 7.4 | 4.0 | 7.1 | 4.0 | 4.0 | 3.1 | 6.9 | 4.0 | 8.2 | 3.7 |
| 37 | Parkinson’s disease ^c^ | G20, G21, G22, F02.3 | Cat I | 57,583 | 7.0 | (6.5) | 4.1 | 49 | 7.2 | 4.2 | 6.6 | 3.9 | 6.1 | 3.7 | 6.7 | 4.1 | 8.0 | 4.0 |
| 38 | Extrapyramidal and movement disorders | G23–G26 | Cat I | 10,837 | 6.8 | (5.8) | 4.3 | 55 | 7.0 | 4.3 | 6.6 | 4.2 | 4.7 | 3.4 | 6.7 | 4.2 | 8.9 | 4.2 |
| 39 | Sclerosis | G35 | Cat I | 13,284 | 4.7 | (4.4) | 3.1 | 196 | 4.8 | 3.2 | 4.4 | 3.1 | 3.9 | 2.5 | 4.9 | 3.2 | 6.6 | 3.9 |
| 40 | Demyelinating diseases of the central nervous system | G36–G37 | Cat I | 4,571 | 5.3 | (5.3) | 3.4 | 157 | 5.4 | 3.3 | 5.2 | 3.5 | 4.3 | 2.7 | 5.8 | 3.5 | 8.7 | 4.2 |
| 41 | Epilepsy ^c^ | G40–G41 | Cat III | 61,695 | 6.2 | (5.7) | 4.2 | 87 | 6.5 | 4.3 | 5.9 | 4.1 | 4.3 | 3.4 | 6.7 | 4.2 | 8.7 | 4.2 |
| 42 | Migraine ^c^ | G43 | Cat II | 149,866 | 4.4 | (4.3) | 3.2 | 204 | 4.4 | 3.2 | 4.2 | 3.3 | 3.4 | 2.5 | 4.7 | 3.3 | 7.7 | 4.1 |
| 43 | Other headache syndromes | G44 | Cat III | 16,469 | 5.4 | (5.6) | 3.7 | 153 | 5.5 | 3.7 | 5.1 | 3.7 | 4.1 | 2.9 | 6.2 | 3.9 | 9.8 | 4.5 |
| 44 | Transient cerebral ischaemic attacks and related syndromes and vascular syndromes of brain in cerebrovascular diseases | G45–G46 | Cat II | 43,977 | 7.1 | (5.6) | 4.0 | 44 | 7.3 | 4.0 | 6.9 | 3.9 | 4.7 | 3.2 | 6.5 | 3.8 | 8.4 | 4.0 |
| 45 | Sleep disorders | G47 | Cat III | 36,806 | 5.6 | (5.4) | 3.8 | 137 | 6.3 | 4.1 | 5.3 | 3.7 | 3.8 | 2.9 | 5.8 | 3.8 | 9.2 | 4.4 |
| 46 | Disorders of trigeminal nerve and facial nerve disorders | G50–G51 | Cat I | 21,488 | 5.3 | (4.5) | 3.9 | 162 | 5.6 | 4.0 | 4.8 | 3.7 | 3.2 | 2.6 | 5.5 | 3.8 | 8.2 | 4.2 |
| 47 | Disorders of other cranial nerves, cranial nerve disorders in diseases classified elsewhere, nerve root and plexus disorders and nerve root and plexus compressions in diseases classified elsewhere | G52–G55 | Cat I | 12,429 | 6.0 | (5.3) | 4.0 | 100 | 6.3 | 4.0 | 5.7 | 3.8 | 4.2 | 2.8 | 6.1 | 3.8 | 8.9 | 4.4 |
| 48 | Mononeuropathies of upper limb | G56 | Cat I | 122,395 | 5.2 | (4.5) | 3.6 | 171 | 5.3 | 3.6 | 5.0 | 3.6 | 3.5 | 2.6 | 5.2 | 3.5 | 7.9 | 4.1 |
| 49 | Mononeuropathies of lower limb, other mononeuropathies and mononeuropathy in diseases classified elsewhere | G57–G59 | Cat I | 18,627 | 5.7 | (4.9) | 3.9 | 124 | 6.0 | 3.9 | 5.3 | 3.8 | 3.8 | 2.8 | 5.8 | 3.8 | 8.6 | 4.3 |
| 50 | Polyneuropathies and other disorders of the peripheral nervous system | G60–G64 | Cat I | 30,289 | 7.3 | (5.9) | 4.4 | 38 | 7.7 | 4.5 | 7.1 | 4.3 | 4.5 | 3.3 | 7.2 | 4.3 | 9.1 | 4.4 |
| 51 | Diseases of myoneural junction and muscle | G70–G73 | Cat I | 5,758 | 5.8 | (5.4) | 4.1 | 113 | 6.1 | 4.3 | 5.5 | 4.0 | 3.8 | 2.9 | 6.5 | 4.2 | 8.5 | 4.3 |
| 52 | Cerebral palsy and other paralytic syndromes | G80–G83 | Cat I | 14,410 | 6.0 | (5.9) | 4.1 | 101 | 6.5 | 4.3 | 5.6 | 3.9 | 4.3 | 3.0 | 6.8 | 4.2 | 9.2 | 4.4 |
| 53 | Other disorders of the nervous system | G90–G99 | Cat I | 44,394 | 6.4 | (5.6) | 4.1 | 70 | 6.4 | 4.1 | 6.3 | 4.1 | 4.3 | 3.0 | 6.7 | 4.0 | 9.0 | 4.2 |
|  | **H – Diseases of the eye and adnexa and diseases of the ear and mastoid process** | **H02–H06; H17–H18; H25–H28; H31–H32; H34–H36; H40–55; H57; H80,H810; H93, H90–H93** |  | **448,176** | **5.6** | **(4.4)** | **3.6** | **129** | **5.8** | **3.6** | **5.4** | **3.6** | **3.4** | **2.6** | **5.3** | **3.4** | **6.8** | **3.7** |
| 54 | Disorders of eyelid, lacrimal system and orbit | H02–H06 | Cat IV | 13,191 | 5.6 | (4.3) | 3.8 | 138 | 5.5 | 3.7 | 5.7 | 3.9 | 3.3 | 2.5 | 5.3 | 3.6 | 7.7 | 3.9 |
| 55 | Corneal scars and opacities | H17 | Cat II | 2,173 | 5.6 | (4.6) | 4.0 | 135 | 6.1 | 4.1 | 5.2 | 3.9 | 3.1 | 2.4 | 5.5 | 3.9 | 8.0 | 3.9 |
| 56 | Other disorders of cornea | H18 | Cat II | 9,473 | 5.6 | (4.3) | 3.8 | 133 | 6.0 | 3.9 | 5.0 | 3.7 | 3.1 | 2.5 | 5.3 | 3.6 | 7.6 | 3.8 |
| 57 | Diseases of the eye lens (cataracts) | H25–H28 | Cat IV | 68,009 | 6.4 | (5.0) | 3.8 | 68 | 6.5 | 3.8 | 6.3 | 3.8 | 4.3 | 3.3 | 5.9 | 3.8 | 6.9 | 3.8 |
| 58 | Disorders of the choroid and retina | H31–H32 | Cat I | 1,900 | 5.6 | (4.5) | 3.9 | 136 | 5.8 | 3.9 | 5.3 | 3.8 | 3.2 | 2.6 | 5.5 | 3.6 | 7.9 | 4.0 |
| 59 | Retinal vascular occlusions | H34 | Cat I | 10,358 | 6.9 | (5.0) | 3.9 | 52 | 7.1 | 3.9 | 6.8 | 3.9 | 4.0 | 2.7 | 6.3 | 3.8 | 7.9 | 3.9 |
| 60 | Other retinal disorders | H35 | Cat I | 68,485 | 6.5 | (4.7) | 3.9 | 65 | 6.7 | 3.9 | 6.2 | 3.9 | 3.8 | 2.9 | 5.8 | 3.7 | 7.3 | 3.8 |
| 61 | Retinal disorders in diseases classified elsewhere | H36 | Cat III | 19,279 | 7.3 | (6.2) | 3.8 | 40 | 7.6 | 3.9 | 7.0 | 3.8 | 4.9 | 3.0 | 7.5 | 3.7 | 9.2 | 3.9 |
| 62 | Glaucoma ^c^ | H40–H42 | Cat III | 67,310 | 5.9 | (4.5) | 3.6 | 112 | 6.1 | 3.6 | 5.6 | 3.5 | 4.0 | 3.0 | 5.2 | 3.4 | 6.7 | 3.6 |
| 63 | Disorders of the vitreous body and globe | H43–H45 | Cat IV | 7,572 | 5.6 | (4.6) | 3.9 | 126 | 5.6 | 3.8 | 5.7 | 4.0 | 3.4 | 2.7 | 5.6 | 3.8 | 8.3 | 3.9 |
| 64 | Disorders of optic nerve and visual pathways | A: H46, H48 and B: H47 | A: Cat I  B: Cat III | 6,184 | 5.4 | (5.3) | 3.6 | 151 | 5.4 | 3.6 | 5.3 | 3.6 | 4.3 | 2.8 | 5.7 | 3.7 | 8.0 | 3.9 |
| 65 | Disorders of ocular muscles, binocular movement, accommodation and refraction | A: H49 and B: H50, H52 and C: H51 | A: Cat III B: Cat IV C: Cat I | 18,247 | 4.1 | (4.3) | 3.3 | 213 | 4.3 | 3.4 | 3.8 | 3.2 | 2.9 | 2.3 | 4.8 | 3.5 | 8.0 | 4.0 |
| 66 | Visual disturbances | H53 | Cat I | 22,232 | 6.2 | (5.3) | 4.1 | 91 | 6.5 | 4.2 | 5.8 | 4.0 | 4.0 | 3.1 | 6.3 | 4.0 | 8.4 | 4.1 |
| 67 | Blindness and partial sight | H54 | Cat I | 6,614 | 7.8 | (6.5) | 4.6 | 26 | 8.2 | 4.7 | 7.2 | 4.5 | 5.2 | 3.5 | 7.4 | 4.6 | 9.8 | 4.4 |
| 68 | Nystagmus and other irregular eye movements and other disorders of eye and adnexa | H55, H57 | Cat I | 11,133 | 5.7 | (5.1) | 4.0 | 120 | 5.9 | 4.1 | 5.4 | 3.8 | 3.9 | 2.9 | 6.0 | 3.9 | 8.4 | 4.3 |
| 69 | Otosclerosis | H80 | Cat II | 10,360 | 5.3 | (4.2) | 3.5 | 164 | 5.3 | 3.5 | 5.1 | 3.5 | 3.2 | 2.2 | 5.0 | 3.3 | 7.4 | 3.7 |
| 70 | Ménière’s disease ^c^ | H810 | Cat I | 10,003 | 6.2 | (4.8) | 3.8 | 88 | 6.3 | 3.8 | 6.0 | 3.8 | 3.8 | 2.6 | 5.6 | 3.5 | 8.1 | 4.0 |
| 71 | Other diseases of the inner ear | H83 | Cat II | 29,865 | 6.3 | (5.1) | 3.6 | 75 | 6.4 | 3.7 | 6.3 | 3.6 | 4.0 | 2.5 | 5.8 | 3.3 | 7.6 | 3.7 |
| 72 | Conductive and sensorineural hearing loss | H90 | Cat II | 43,238 | 5.9 | (4.6) | 3.7 | 110 | 6.0 | 3.8 | 5.7 | 3.7 | 3.4 | 2.4 | 5.7 | 3.6 | 7.7 | 3.8 |
| 73 | Other hearing loss and other disorders of ear, not elsewhere classified | H910, H912, H913, H918, H930, H932, H933 | Cat II | 8,306 | 6.3 | (5.3) | 3.8 | 81 | 6.4 | 3.8 | 6.2 | 3.9 | 4.2 | 2.9 | 6.1 | 3.6 | 8.2 | 4.0 |
| 74 | Presbycusis (age-related hearing loss) | H911 | Cat II | 80,659 | 7.0 | (5.0) | 3.7 | 45 | 7.2 | 3.7 | 6.9 | 3.7 | 4.1 | 2.7 | 6.3 | 3.6 | 7.3 | 3.7 |
| 75 | Hearing loss, unspecified | H919 | Cat II | 87,806 | 6.3 | (4.8) | 3.7 | 77 | 6.6 | 3.8 | 6.1 | 3.6 | 3.8 | 2.7 | 5.8 | 3.5 | 7.7 | 3.8 |
| 76 | Tinnitus | H931 | Cat I | 40,124 | 5.9 | (4.8) | 3.6 | 107 | 6.1 | 3.7 | 5.7 | 3.6 | 3.7 | 2.5 | 5.7 | 3.4 | 8.1 | 3.8 |
| 77 | Other specified disorders of ear | H938 | Cat II | 20,537 | 6.1 | (4.7) | 3.7 | 97 | 6.2 | 3.7 | 5.9 | 3.6 | 3.7 | 2.7 | 5.7 | 3.5 | 7.5 | 3.7 |
|  | **I – Diseases of the circulatory system** | **I05–I06; I10–28; I30–33;I36–141; I44–I52; I60–I88; I90–I94; I96–I99** |  | **1,254,427** | **4.9** | **(4.0)** | **3.3** | **188** | **4.9** | **3.3** | **4.8** | **3.2** | **3.3** | **2.5** | **4.6** | **3.1** | **6.2** | **3.5** |
| 78 | Aortic and mitral valve disease ^c^ | I05, I06, I34, I35 | Cat III | 30,123 | 8.0 | (5.8) | 4.2 | 17 | 8.3 | 4.3 | 7.8 | 4.1 | 4.5 | 3.4 | 7.4 | 4.1 | 8.9 | 4.1 |
| 79 | Hypertensive diseases ^c^ | I10–I15 | Cat III | 1,060,046 | 5.1 | (4.2) | 3.3 | 179 | 5.1 | 3.3 | 5.0 | 3.3 | 3.7 | 2.7 | 4.7 | 3.1 | 6.3 | 3.6 |
| 80 | Heart failure ^c^ | I11.0, I13.0, I13.2, I42.0, I42.6, I42.7, I42.9, I50.0, I50.1, I50.9 | Cat II | 37,540 | 8.8 | (7.0) | 4.2 | 3 | 9.2 | 4.3 | 8.6 | 4.1 | 5.9 | 3.5 | 8.3 | 4.1 | 9.7 | 4.1 |
| 80A | Ischaemic heart diseases | I20–I25 | Cat III | 139,173 | 7.9 | (6.2) | 4.0 | 22 | 8.4 | 4.2 | 7.6 | 3.8 | 5.2 | 3.6 | 7.3 | 3.8 | 9.3 | 3.9 |
| 81 | Angina pectoris | I20 | Cat III | 78,476 | 7.9 | (6.1) | 4.1 | 24 | 8.2 | 4.2 | 7.6 | 3.9 | 5.2 | 3.7 | 7.4 | 3.9 | 9.5 | 4.0 |
| 82 | Acute myocardial infarction and  subsequent myocardial infarction | I21–I22 | Cat III | 36,654 | 8.1 | (6.7) | 4.0 | 12 | 9.0 | 4.2 | 7.7 | 3.8 | 5.9 | 3.4 | 7.5 | 3.7 | 9.6 | 4.1 |
| 83 | AMI complex/other | I23–I24 | Cat III | 2,969 | 9.3 | (7.1) | 4.6 | 2 | 9.7 | 4.7 | 9.0 | 4.4 | 5.5 | 3.8 | 8.9 | 4.5 | 10.8 | 4.2 |
| 84 | Chronic ischaemic heart disease | I25 | Cat III | 84,592 | 8.8 | (7.1) | 4.0 | 6 | 9.5 | 4.2 | 8.4 | 3.8 | 6.5 | 3.7 | 8.2 | 3.8 | 9.9 | 4.0 |
| 85 | Pulmonary heart disease and diseases of pulmonary circulation | I26–I28 | Cat III | 15,352 | 7.7 | (6.1) | 4.5 | 30 | 7.9 | 4.5 | 7.4 | 4.4 | 4.6 | 3.4 | 7.4 | 4.3 | 9.2 | 4.4 |
| 86 | Acute pericarditis | I30 | Cat II | 5,563 | 5.1 | (5.1) | 4.0 | 175 | 6.4 | 4.4 | 4.6 | 3.8 | 3.2 | 2.8 | 5.7 | 4.0 | 8.8 | 4.3 |
| 87 | Other forms of heart disease | I31–I43, except I34–I35 and I42 | Cat II | 8,119 | 8.0 | (6.6) | 4.6 | 15 | 8.6 | 4.7 | 7.7 | 4.6 | 4.6 | 3.5 | 8.1 | 4.4 | 10.3 | 4.3 |
| 88 | Atrioventricular and left bundle branch block | I44 | Cat II | 14,604 | 7.9 | (5.6) | 4.2 | 21 | 7.9 | 4.3 | 7.9 | 4.2 | 4.3 | 3.5 | 7.3 | 4.2 | 8.7 | 4.1 |
| 89 | Other conduction disorders | I45–46 | Cat II | 11,823 | 7.6 | (6.0) | 4.5 | 31 | 7.8 | 4.6 | 7.5 | 4.4 | 4.2 | 3.1 | 7.5 | 4.3 | 9.6 | 4.3 |
| 90 | Paroxysmal tachycardia | I47 | Cat II | 39,510 | 6.7 | (5.3) | 4.1 | 60 | 6.6 | 4.1 | 6.9 | 4.1 | 3.9 | 2.9 | 6.6 | 3.9 | 9.1 | 4.1 |
| 91 | Atrial fibrillation and flutter | I48 | Cat II | 112,342 | 7.4 | (5.4) | 3.9 | 37 | 7.8 | 4.0 | 7.0 | 3.9 | 4.2 | 3.1 | 6.6 | 3.8 | 8.3 | 3.9 |
| 92 | Other cardiac arrhythmias | I49 | Cat II | 34,418 | 7.1 | (5.3) | 4.2 | 43 | 7.0 | 4.3 | 7.2 | 4.2 | 3.9 | 2.9 | 6.6 | 4.0 | 9.1 | 4.1 |
| 93 | Complications and ill-defined descriptions of heart disease and other heart disorders in diseases classified elsewhere | I51–52 | Cat II | 7,337 | 8.3 | (6.5) | 4.6 | 11 | 8.3 | 4.7 | 8.3 | 4.5 | 4.6 | 3.2 | 8.2 | 4.4 | 10.2 | 4.3 |
| 94 | Stroke | I60, I61,I63–I64, Z501 (rehabilitation) | Cat II | 72,606 | 7.5 | (6.2) | 3.9 | 32 | 7.9 | 3.9 | 7.3 | 3.8 | 5.3 | 3.4 | 7.1 | 3.8 | 8.4 | 3.8 |
| 95 | Cerebrovascular diseases | I62, I65–I68 | Cat II | 17,308 | 7.8 | (6.1) | 4.2 | 25 | 7.9 | 4.3 | 7.8 | 4.2 | 4.8 | 3.4 | 7.5 | 4.0 | 9.4 | 4.1 |
| 96 | Sequelae of cerebrovascular disease | I69 | Cat II | 50,952 | 8.8 | (7.3) | 4.0 | 7 | 9.0 | 4.1 | 8.5 | 3.9 | 6.5 | 3.6 | 8.4 | 4.0 | 9.4 | 3.9 |
| 97 | Atherosclerosis | I70 | Cat II | 32,064 | 8.7 | (6.7) | 4.4 | 8 | 8.9 | 4.4 | 8.6 | 4.4 | 5.7 | 4.1 | 8.3 | 4.3 | 9.5 | 4.3 |
| 98 | Aortic aneurysm and aortic dissection | I71 | Cat III | 10,296 | 7.9 | (5.8) | 4.0 | 20 | 8.2 | 4.1 | 7.8 | 3.9 | 4.6 | 3.0 | 7.3 | 3.9 | 8.8 | 4.0 |
| 99 | Diseases of arteries, arterioles and capillaries | I72, I74, I77–I79 | Cat III | 11,830 | 7.0 | (5.6) | 4.5 | 47 | 6.6 | 4.5 | 7.5 | 4.5 | 4.0 | 3.3 | 6.9 | 4.3 | 9.9 | 4.4 |
| 100 | Other peripheral vascular diseases | I73 | Cat III | 28,508 | 7.9 | (5.7) | 4.2 | 23 | 8.0 | 4.2 | 7.8 | 4.2 | 4.3 | 3.2 | 7.5 | 4.1 | 9.1 | 4.2 |
| 101 | Phlebitis, thrombosis of the portal vein and others | I80–I82 | Cat III | 37,388 | 6.2 | (5.1) | 4.1 | 89 | 6.5 | 4.3 | 5.8 | 4.0 | 4.1 | 3.2 | 6.0 | 4.0 | 8.1 | 4.2 |
| 102 | Varicose veins of lower extremities | I83 | Cat IV | 23,530 | 4.3 | (3.8) | 3.4 | 209 | 4.2 | 3.3 | 4.4 | 3.6 | 2.7 | 2.0 | 4.4 | 3.2 | 7.6 | 4.1 |
| 103 | Haemorrhoids ^c^ | I84 | Cat IV | 74,285 | 4.3 | (4.1) | 3.4 | 207 | 4.4 | 3.5 | 4.1 | 3.3 | 2.8 | 2.2 | 4.8 | 3.3 | 8.0 | 4.1 |
| 104 | Oesophageal varices (chronic), varicose veins of other sites, other disorders of veins, non-specific lymphadenitis, other non-infective disorders of lymphatic vessels and lymph nodes and other and unspecified disorders of the circulatory system | I85–I99, except I89 and I95 | Cat III | 15,194 | 6.1 | (5.3) | 4.4 | 93 | 6.6 | 4.4 | 5.7 | 4.3 | 3.3 | 2.8 | 6.7 | 4.2 | 9.3 | 4.4 |
|  | **J – Diseases of the respiratory system** | **J30.1; J40–J47; J60–J84; J95, J97–J99** |  | **1,210,598** | **4.2** | **(3.8)** | **3.3** | **210** | **4.4** | **3.3** | **4.0** | **3.2** | **2.6** | **2.0** | **4.6** | **3.2** | **7.3** | **3.7** |
| 105 | Respiratory allergy ^c^ | J30, except J30.0 | Cat I | 841,685 | 4.1 | (3.7) | 3.2 | 214 | 4.3 | 3.3 | 3.8 | 3.1 | 2.5 | 2.0 | 4.5 | 3.2 | 7.2 | 3.8 |
| 105A | Chronic lower respiratory diseases ^c^ | J40–J43, J47 | Cat I | 418,120 | 5.4 | (4.8) | 3.6 | 145 | 5.6 | 3.6 | 5.2 | 3.5 | 3.6 | 2.3 | 5.6 | 3.5 | 8.3 | 3.9 |
| 106 | Bronchitis, not specified as acute  or chronic, simple and  mucopurulent chronic bronchitis  and unspecified chronic bronchitis | J40–J42 | Cat II | 12,790 | 9.8 | (7.5) | 4.7 | 1 | 10.1 | 4.6 | 9.5 | 4.7 | 6.1 | 4.1 | 9.3 | 4.6 | 11.3 | 4.3 |
| 107 | Emphysema | J43 | Cat II | 5,557 | 8.6 | (6.8) | 4.2 | 9 | 9.0 | 4.3 | 8.2 | 4.1 | 5.4 | 3.2 | 8.3 | 4.1 | 10.2 | 4.1 |
| 108 | Chronic obstructive lung disease (COPD) ^c^ | J44, J96, J13–J18 | Cat I | 216,184 | 6.5 | (5.3) | 3.9 | 64 | 6.7 | 3.9 | 6.3 | 3.9 | 3.9 | 2.6 | 6.5 | 3.7 | 8.5 | 3.9 |
| 109 | Asthma, status asthmaticus ^c^ | J45–J46 | Cat I | 361,129 | 5.4 | (5.0) | 3.6 | 148 | 5.7 | 3.6 | 5.0 | 3.5 | 3.5 | 2.2 | 6.0 | 3.5 | 8.6 | 3.8 |
| 110 | Bronchiectasis | J47 | Cat II | 4,362 | 7.5 | (6.5) | 4.0 | 33 | 7.5 | 4.0 | 7.6 | 4.1 | 5.4 | 2.8 | 7.3 | 3.9 | 9.5 | 4.2 |
| 111 | Other diseases of the respiratory system | J60–J84; J95, J97–J99 | Cat II | 21,993 | 7.9 | (6.4) | 4.6 | 19 | 8.2 | 4.5 | 7.7 | 4.5 | 4.9 | 3.6 | 7.8 | 4.4 | 9.8 | 4.3 |
|  | **K – Diseases of the digestive system** | **K25–K27; K40, K43, K50–52; K58–K59; K71–K77; K86–K87** |  | **329,337** | **5.7** | **(4.8)** | **4.0** | **121** | **5.9** | **4.0** | **5.4** | **3.9** | **3.4** | **2.7** | **5.8** | **3.8** | **8.3** | **4.1** |
| 112 | Ulcers ^c^ | K25–K27 | Cat IV | 157,379 | 6.3 | (5.1) | 4.1 | 76 | 6.5 | 4.1 | 6.1 | 4.1 | 3.7 | 3.0 | 6.3 | 4.0 | 8.5 | 4.1 |
| 113 | Inguinal hernia | K40 | Cat IV | 25,032 | 4.3 | (3.8) | 3.3 | 208 | 5.2 | 3.8 | 4.2 | 3.2 | 2.4 | 1.9 | 4.1 | 3.1 | 6.8 | 3.8 |
| 114 | Ventral hernia | K43 | Cat IV | 7,941 | 6.5 | (5.3) | 4.3 | 63 | 6.6 | 4.3 | 6.4 | 4.2 | 4.0 | 3.2 | 6.7 | 4.1 | 8.8 | 4.2 |
| 115 | Crohn’s diease | K50 | Cat I | 18,913 | 4.9 | (4.9) | 3.6 | 187 | 5.2 | 3.7 | 4.5 | 3.5 | 3.6 | 2.6 | 5.7 | 3.8 | 8.6 | 4.3 |
| 116 | Ulcerative colitis | K51 | Cat II | 29,538 | 4.9 | (4.6) | 3.7 | 185 | 5.2 | 3.9 | 4.5 | 3.5 | 3.3 | 2.5 | 5.3 | 3.7 | 8.5 | 4.4 |
| 117 | Other non-infective gastroenteritis and colitis | K52 | Cat III | 20,844 | 7.0 | (5.8) | 4.5 | 48 | 7.3 | 4.5 | 6.5 | 4.4 | 4.1 | 3.0 | 7.2 | 4.3 | 9.5 | 4.4 |
| 118 | Irritable bowel syndrome (IBS) | K58 | Cat II | 37,593 | 5.2 | (4.9) | 3.8 | 170 | 5.3 | 3.9 | 4.7 | 3.7 | 3.6 | 2.7 | 5.8 | 3.8 | 8.8 | 4.4 |
| 119 | Other functional intestinal disorders | K59 | Cat III | 51,933 | 6.9 | (5.7) | 4.5 | 51 | 7.0 | 4.5 | 6.9 | 4.5 | 4.1 | 3.2 | 7.1 | 4.4 | 9.3 | 4.4 |
| 120 | Diseases of liver, biliary tract and pancreas | K71–K77; K86–K87 | Cat II | 26,956 | 6.6 | (5.7) | 4.2 | 62 | 6.8 | 4.2 | 6.3 | 4.0 | 4.4 | 3.4 | 6.8 | 4.1 | 8.6 | 4.3 |
|  | **L – Diseases of the skin and subcutaneous tissue** | **L40** |  | **65,469** | **4.7** | (4.0) | **3.5** | **194** | **4.9** | **3.6** | **4.4** | **3.4** | **2.8** | **2.2** | **4.8** | **3.4** | **7.6** | **4.0** |
| 121 | Psoriasis ^c^ | L40 | Cat I | 65,469 | 4.7 | (4.0) | 3.5 | 195 | 4.9 | 3.6 | 4.4 | 3.4 | 2.8 | 2.2 | 4.8 | 3.4 | 7.6 | 4.0 |
|  | **M – Diseases of the musculoskeletal system and connective tissue** | **M01–M25; M30–M36; M40–M54; M60.1–M99** |  | **1,032,808** | **4.7** | (3.9) | **3.4** | **193** | **4.9** | **3.4** | **4.4** | **3.4** | **2.9** | **2.2** | **4.7** | **3.2** | **6.9** | **3.7** |
| 122 | Infectious arthropathies | M01–M03 | Cat II | 9,402 | 5.1 | (4.9) | 3.7 | 176 | 5.3 | 3.7 | 4.9 | 3.7 | 3.5 | 2.5 | 5.7 | 3.8 | 8.5 | 4.2 |
| 122A | Inflammatory polyarthropathies and ankylosing spondylitis ^c^ | M05–M14, M45 | Cat I | 165,944 | 6.0 | (4.8) | 3.9 | 103 | 6.3 | 4.0 | 5.6 | 3.8 | 3.6 | 2.6 | 5.8 | 3.7 | 8.3 | 4.1 |
| 123 | Rheumatoid arthritis ^c^ | M05, M06, M07.1, M07.2, M07.3, M08, M09 | Cat I | 77,345 | 5.8 | (4.9) | 3.8 | 115 | 6.1 | 3.9 | 5.4 | 3.7 | 3.7 | 2.6 | 5.9 | 3.7 | 8.4 | 4.2 |
| 124 | Inflammatory polyarthropathies  – except rheumatoid arthritis ^c^ | M074–M079, M10–M14, M45 | Cat I | 115,945 | 6.3 | (5.2) | 4.0 | 78 | 6.9 | 4.0 | 5.9 | 3.8 | 4.0 | 2.7 | 6.0 | 3.7 | 8.5 | 4.1 |
| 125 | Polyarthrosis [arthrosis] | M15 | Cat I | 16,935 | 7.7 | (5.7) | 4.3 | 28 | 7.8 | 4.3 | 7.4 | 4.3 | 4.9 | 3.3 | 7.0 | 4.0 | 9.6 | 4.4 |
| 126 | Coxarthrosis [arthrosis of hip] | M16 | Cat I | 104,115 | 6.2 | (4.5) | 3.8 | 90 | 6.4 | 3.8 | 5.8 | 3.8 | 3.7 | 2.7 | 5.5 | 3.6 | 7.2 | 3.9 |
| 127 | Gonarthrosis [arthrosis of knee] | M17 | Cat I | 178,811 | 5.6 | (4.3) | 3.7 | 125 | 6.0 | 3.7 | 5.2 | 3.6 | 3.4 | 2.6 | 5.2 | 3.4 | 7.5 | 3.9 |
| 128 | Arthrosis of first carpometacarpal joint and other arthrosis | M18–M19 | Cat I | 91,101 | 6.1 | (4.8) | 4.0 | 96 | 6.6 | 4.1 | 5.5 | 3.8 | 3.6 | 2.6 | 5.8 | 3.7 | 8.6 | 4.3 |
| 129 | Acquired deformities of fingers and toes | M20 | Cat II | 55,730 | 5.0 | (4.2) | 3.6 | 182 | 5.0 | 3.6 | 4.9 | 3.6 | 3.0 | 2.3 | 5.1 | 3.4 | 7.8 | 4.0 |
| 130 | Other acquired deformities of limbs | M21 | Cat II | 20,584 | 5.5 | (4.8) | 3.8 | 143 | 5.8 | 3.9 | 4.9 | 3.7 | 3.3 | 2.5 | 5.8 | 3.8 | 8.5 | 4.0 |
| 131 | Disorders of patella (knee cap) | M22 | Cat II | 38,999 | 3.3 | (4.1) | 2.6 | 230 | 3.5 | 2.8 | 2.9 | 2.4 | 2.8 | 2.2 | 4.4 | 3.1 | 7.6 | 3.9 |
| 132 | Internal derangement of knee | M230, M231, M233, M235, M236, M238 | Cat IV | 9,192 | 3.6 | (4.4) | 2.7 | 225 | 4.0 | 2.9 | 3.3 | 2.5 | 2.9 | 2.0 | 4.8 | 3.2 | 7.9 | 3.9 |
| 133 | Derangement of meniscus due to old tear or injury | M232 | Cat IV | 36,374 | 4.0 | (4.2) | 2.9 | 215 | 4.6 | 3.1 | 3.6 | 2.6 | 2.9 | 2.0 | 4.6 | 3.0 | 7.5 | 3.8 |
| 134 | Internal derangement of knee, unspecified | M239 | Cat IV | 28,206 | 3.9 | (4.3) | 3.0 | 221 | 4.3 | 3.2 | 3.5 | 2.7 | 2.9 | 2.2 | 4.7 | 3.2 | 7.8 | 4.3 |
| 135 | Other specific joint derangements | M24, except M240–M241 | Cat IV | 5,923 | 3.7 | (4.6) | 3.0 | 223 | 4.5 | 3.3 | 3.2 | 2.6 | 2.8 | 2.2 | 5.2 | 3.4 | 8.3 | 3.8 |
| 136 | Other joint disorders, not elsewhere classified | M25 | Cat IV | 12,043 | 5.3 | (5.2) | 3.7 | 158 | 5.5 | 3.7 | 4.8 | 3.6 | 3.8 | 2.7 | 5.9 | 3.7 | 9.2 | 4.3 |
| 137 | Systemic connective tissue disorders | M30–M36, except M32,M34 | Cat I | 42,631 | 6.8 | (5.5) | 4.2 | 56 | 6.8 | 4.2 | 6.9 | 4.3 | 4.3 | 3.0 | 6.8 | 4.1 | 8.6 | 4.2 |
| 138 | Systemic lupus erythematosus | M32 | Cat I | 3,376 | 7.5 | (7.1) | 4.3 | 34 | 7.5 | 4.2 | 7.6 | 4.3 | 6.0 | 3.5 | 7.8 | 4.3 | 10.1 | 4.7 |
| 139 | Dermatopolymyositis | M33 | Cat I | 1,137 | 7.0 | (5.9) | 4.3 | 46 | 7.4 | 4.4 | 6.5 | 4.0 | 4.7 | 3.4 | 7.2 | 4.2 | 9.6 | 4.2 |
| 140 | Systemic sclerosis | M34 | Cat I | 1,675 | 7.8 | (6.5) | 4.5 | 27 | 7.8 | 4.4 | 7.6 | 5.0 | 5.0 | 3.5 | 8.0 | 4.4 | 10.0 | 4.6 |
| 141 | Kyphosis, lordosis | M40 | Cat I | 4,160 | 5.2 | (5.1) | 3.9 | 167 | 5.7 | 4.0 | 4.6 | 3.7 | 3.5 | 2.5 | 5.7 | 3.9 | 9.9 | 5.1 |
| 142 | Scoliosis | M41 | Cat I | 17,686 | 4.6 | (4.9) | (3.8 | 199 | 4.7 | 4.0 | 4.2 | 3.5 | 2.8 | 2.2 | 6.1 | 4.0 | 9.5 | 4.3 |
| 143 | Spinal osteochondrosis | M42 | Cat I | 8,034 | 5.1 | (5.1) | 3.8 | 177 | 5.9 | 4.2 | 4.6 | 3.5 | 3.4 | 2.6 | 5.7 | 3.9 | 9.2 | 4.4 |
| 144 | Other deforming dorsopathies | M43 | Cat I | 23,756 | 6.2 | (5.0) | 4.2 | 82 | 6.8 | 4.3 | 5.4 | 4.0 | 3.5 | 2.5 | 6.2 | 4.0 | 9.4 | 4.3 |
| 145 | Other inflammatory spondylopathies | M46 | Cat I | 7,086 | 6.1 | (5.5) | 4.1 | 99 | 6.3 | 4.0 | 5.8 | 4.1 | 4.2 | 2.8 | 6.5 | 4.1 | 9.2 | 4.3 |
| 146 | Spondylosis | M47 | Cat I | 61,999 | 6.8 | (5.4) | 4.2 | 59 | 7.2 | 4.2 | 6.3 | 4.0 | 4.3 | 2.8 | 6.3 | 3.9 | 9.3 | 4.3 |
| 147 | Other spondylopathies and spondylopathies in diseases classified elsewhere | M48, M49 | Cat II | 50,805 | 7.7 | (5.7) | 4.3 | 29 | 8.1 | 4.3 | 7.2 | 4.2 | 4.4 | 2.9 | 7.1 | 4.1 | 9.1 | 4.2 |
| 148 | Cervical disc disorders | M50 | Cat IV | 11,476 | 5.4 | (5.3) | 3.7 | 150 | 5.6 | 3.8 | 5.1 | 3.6 | 3.9 | 2.5 | 5.7 | 3.7 | 9.9 | 4.7 |
| 149 | Other intervertebral disc disorders | M51 | Cat IV | 40,161 | 5.4 | (5.1) | 3.9 | 152 | 5.9 | 4.0 | 4.8 | 3.6 | 3.5 | 2.6 | 5.9 | 3.8 | 9.5 | 4.4 |
| 150 | Other dorsopathies, not elsewhere classified | M53 | Cat IV | 7,246 | 5.5 | (5.4) | 3.9 | 140 | 5.8 | 4.0 | 5.2 | 3.8 | 3.9 | 2.7 | 6.2 | 4.0 | 9.7 | 4.6 |
| 151 | Dorsalgia | M54 | Cat IV | 40,780 | 5.7 | (5.3) | 4.1 | 123 | 6.1 | 4.2 | 5.1 | 3.9 | 3.7 | 2.7 | 6.2 | 4.0 | 9.7 | 4.5 |
| 152 | Soft tissue disorders | M60–M63, except M60.0 | Cat IV | 13,422 | 5.3 | (5.4) | 3.9 | 163 | 5.2 | 3.9 | 5.4 | 4.1 | 3.7 | 2.8 | 6.3 | 4.1 | 9.5 | 4.6 |
| 153 | Synovitis and tenosynovitis | M65 | Cat IV | 19,104 | 4.8 | (4.5) | 3.4 | 190 | 5.1 | 3.5 | 4.4 | 3.2 | 3.3 | 2.3 | 5.3 | 3.4 | 7.9 | 4.1 |
| 154 | Disorders of synovium and tendon | M66–68 | Cat IV | 19,669 | 4.0 | (4.3) | 3.1 | 216 | 4.3 | 3.2 | 3.7 | 2.8 | 3.0 | 2.2 | 4.8 | 3.2 | 7.8 | 4.4 |
| 155 | Soft tissue disorders related to use, overuse and pressure | M70 | Cat IV | 11,090 | 5.5 | (4.9) | 3.9 | 144 | 5.8 | 3.9 | 5.0 | 3.9 | 3.3 | 2.4 | 5.9 | 3.9 | 8.9 | 4.3 |
| 156 | Fibroblastic disorders | M72 | Cat I | 43,600 | 5.0 | (4.0) | 3.5 | 184 | 5.2 | 3.5 | 4.8 | 3.4 | 3.1 | 2.5 | 4.7 | 3.3 | 6.7 | 3.8 |
| 157 | Shoulder lesions | M75 | Cat IV | 58,112 | 4.6 | (4.3) | 3.3 | 198 | 4.9 | 3.4 | 4.3 | 3.2 | 3.1 | 2.3 | 4.8 | 3.3 | 8.0 | 4.1 |
| 158 | Enthesopathies of lower limb, excluding foot | M76 | Cat IV | 11,223 | 3.9 | (4.3) | 3.1 | 220 | 4.4 | 3.3 | 3.3 | 2.7 | 2.8 | 2.1 | 4.7 | 3.3 | 8.3 | 4.1 |
| 159 | Other enthesopathies | M77 | Cat IV | 10,500 | 4.5 | (4.5) | 3.2 | 201 | 4.9 | 3.3 | 3.9 | 3.0 | 3.3 | 2.3 | 5.0 | 3.3 | 8.2 | 4.3 |
| 160 | Rheumatism, unspecified | M790 | Cat II | 6,852 | 7.0 | (6.1) | 4.2 | 50 | 7.1 | 4.2 | 6.1 | 4.2 | 5.2 | 3.4 | 7.1 | 4.2 | 10.4 | 4.6 |
| 161 | Myalgia | M791 | Cat II | 10,168 | 6.1 | (5.5) | 4.3 | 92 | 6.4 | 4.2 | 5.6 | 4.3 | 4.2 | 3.0 | 6.4 | 4.2 | 9.6 | 4.6 |
| 162 | Other soft tissue disorders, not elsewhere classified | M792– M794; M798–M799 | Cat II | 7,939 | 5.6 | (5.3) | 4.2 | 132 | 5.8 | 4.1 | 5.3 | 4.2 | 3.6 | 2.7 | 6.3 | 4.1 | 9.9 | 5.1 |
| 163 | Other soft tissue disorders, not elsewhere classified: pain in limb | M796 | Cat IV | 22,201 | 5.3 | (4.9) | 4.0 | 154 | 5.6 | 4.0 | 4.9 | 3.9 | 3.4 | 2.6 | 5.9 | 3.9 | 9.0 | 4.5 |
| 164 | Fibromyalgia | M797 | Cat I | 3,399 | 6.9 | (6.7) | 4.0 | 54 | 6.9 | 4.0 | 6.5 | 4.0 | 5.5 | 3.2 | 7.4 | 4.1 | 10.6 | 4.2 |
| 165 | Osteoporosis ^c^ | M80–M81 | Cat I | 158,813 | 6.4 | (6.0) | 3.9 | 71 | 6.2 | 3.8 | 7.4 | 4.2 | 5.9 | 3.6 | 5.7 | 3.7 | 7.4 | 3.9 |
| 166 | Osteoporosis in diseases classified elsewhere | M82 | Cat I | 1,007 | 8.4 | (7.0) | 4.4 | 10 | 8.5 | 4.5 | 8.3 | 4.4 | 6.1 | 4.6 | 8.4 | 4.3 | 9.7 | 4.3 |
| 167 | Adult osteomalacia and other disorders of bone density and structure | M83, M85, except M833 | Cat I | 43,271 | 6.0 | (5.0) | 3.8 | 102 | 5.9 | 3.7 | 6.4 | 4.2 | 4.1 | 3.0 | 5.6 | 3.6 | 8.1 | 4.0 |
| 168 | Disorders of continuity of bone | M84 | Cat IV | 1,865 | 5.3 | (5.1) | 4.1 | 155 | 6.3 | 4.3 | 4.4 | 3.7 | 3.0 | 2.4 | 6.3 | 4.2 | 8.8 | 4.1 |
| 169 | Other osteopathies | M86–M90 | Cat I | 24,251 | 6.3 | (5.2) | 4.2 | 79 | 6.5 | 4.1 | 6.0 | 4.3 | 3.9 | 2.9 | 6.3 | 4.0 | 8.6 | 4.2 |
| 170 | Other disorders of the musculoskeletal system and connective tissue | M95–M99 | Cat I | 30,038 | 5.4 | (5.1) | 4.1 | 146 | 6.2 | 4.3 | 4.7 | 3.8 | 3.2 | 2.5 | 6.1 | 4.1 | 9.7 | 4.4 |
|  | **N – Diseases of the genitourinary system** | **N18** |  | **20,162** | **8.8** | **(6.9)** | **4.5** | **4** | **9.0** | **4.5** | **8.7** | **4.5** | **5.4** | **3.6** | **8.6** | **4.4** | **10.0** | **4.3** |
| 171 | Chronic renal failure (CRF) ^c^ | N18 | Cat I | 20,162 | 8.8 | (6.9) | 4.5 | 5 | 9.0 | 4.5 | 8.7 | 4.5 | 5.4 | 3.6 | 8.6 | 4.4 | 10.0 | 4.3 |
|  | **Q – Congenital malformations, deformations and chromosomal abnormalities** | **Q00–Q56; Q60–Q99** |  | **124,898** | **4.0** | (4.3) | **3.3** | **218** | **4.1** | **3.3** | **3.8** | **3.2** | **2.8** | **2.2** | **5.0** | **3.5** | **8.2** | **4.2** |
| 172 | Congenital malformations: of the nervous, circulatory and respiratory systems, cleft palate and cleft lip, urinary tract, bones and muscles, other and chromosomal abnormalities not elsewhere classified | Q00–Q07; Q20–Q37; Q60–Q99 | Cat I | 85,534 | 4.1 | (4.5) | 3.3 | 212 | 4.2 | 3.3 | 4.0 | 3.3 | 3.0 | 2.3 | 5.3 | 3.6 | 8.4 | 4.2 |
| 173 | Congenital malformations of eye, ear, face and neck | Q10–Q18 | Cat I | 19,689 | 3.4 | (3.9) | 2.8 | 229 | 3.4 | 2.9 | 3.2 | 2.8 | 2.6 | 2.0 | 4.4 | 3.2 | 7.8 | 4.1 |
| 174 | Other congenital malformations of the digestive system | Q38–Q45 | Cat I | 6,481 | 5.9 | (5.0) | 4.2 | 109 | 6.2 | 4.4 | 5.4 | 4.0 | 3.5 | 2.8 | 6.2 | 4.2 | 8.7 | 4.4 |
| 175 | Congenital malformations of the sexual organs | Q50–Q56 | Cat I | 16,192 | 3.5 | (3.8) | 2.9 | 227 | 3.5 | 2.7 | 3.5 | 3.0 | 2.6 | 2.1 | 4.2 | 3.1 | 7.1 | 3.9 |
|  | **F – Mental and behavioural disorders** | **F00–99** |  | **683,194** | **4.8** | **(4.5)** | **3.5** | **192** | **4.9** | **3.5** | **4.5** | **3.5** | **3.2** | **2.4** | **5.3** | **3.5** | **7.6** | **3.9** |
| 176 | Dementia ^c^ | F00, G30, F01, F02.0, F03.9, G31.8B, G31.8E, G31.9, G31.0B | Cat I | 36,803 | 7.4 | (6.8) | 3.8 | 36 | 7.3 | 3.8 | 7.5 | 3.9 | 6.5 | 3.7 | 7.4 | 4.1 | 7.3 | 3.7 |
| 177 | Organic, including symptomatic, mental disorders | F04–F09 | Cat I | 26,430 | 8.0 | (7.1) | 4.4 | 18 | 8.4 | 4.4 | 7.6 | 4.3 | 6.0 | 3.8 | 7.9 | 4.4 | 9.2 | 4.1 |
| 178 | Mental and behavioural disorders due to use of alcohol | F10 | Cat III | 59,143 | 5.9 | (5.9) | 3.9 | 104 | 6.6 | 4.1 | 5.6 | 3.8 | 4.3 | 3.1 | 6.6 | 3.9 | 8.5 | 4.2 |
| 179 | Mental and behavioural disorders due to psychoactive substance use | F11–F19 | Cat III | 53,669 | 5.8 | (6.0) | 4.0 | 119 | 6.0 | 4.2 | 5.5 | 3.7 | 4.5 | 3.1 | 6.9 | 4.3 | 9.1 | 4.4 |
| 180 | Schizophrenia ^c^ | F20 | Cat I | 29,422 | 5.9 | (6.1) | 3.7 | 105 | 6.7 | 4.1 | 5.3 | 3.3 | 5.5 | 3.3 | 6.2 | 4.0 | 7.3 | 4.1 |
| 181 | Schizotypal and delusional disorders | F21–F29 | Cat I | 39,694 | 6.1 | (6.2) | 3.8 | 94 | 6.7 | 4.0 | 5.6 | 3.5 | 5.3 | 3.2 | 6.6 | 4.1 | 8.2 | 4.2 |
| 182 | Bipolar affective disorder ^c^ | F30–F31 | Cat I | 22,669 | 6.9 | (6.5) | 4.0 | 53 | 7.2 | 4.1 | 6.4 | 3.9 | 5.6 | 3.3 | 7.2 | 4.2 | 8.9 | 4.3 |
| 183 | Depression ^c^ | F32, F33, F34.1, F06.32 | Cat III | 454,933 | 5.1 | (4.8) | 3.6 | 173 | 5.2 | 3.6 | 5.1 | 3.7 | 3.5 | 2.5 | 5.5 | 3.6 | 7.9 | 3.9 |
| 184 | Mood (affective) disorders | F340, F348–F349, F38–F39 | Cat I | 6,887 | 7.3 | (7.0) | 4.3 | 39 | 7.5 | 4.4 | 6.9 | 4.1 | 5.9 | 3.5 | 8.0 | 4.4 | 9.8 | 4.4 |
| 185 | Phobic anxiety disorders | F40 | Cat II | 14,324 | 5.2 | (6.1) | 3.3 | 165 | 5.3 | 3.3 | 5.1 | 3.3 | 4.7 | 2.9 | 6.6 | 3.9 | 9.3 | 4.5 |
| 186 | Other anxiety disorders | F41 | Cat II | 38,079 | 6.1 | (6.5) | 3.9 | 95 | 6.2 | 3.9 | 6.0 | 3.9 | 4.9 | 3.1 | 7.2 | 4.2 | 9.9 | 4.5 |
| 187 | Obsessive compulsive disorder (OCD) ^c^ | F42 | Cat II | 10,062 | 5.0 | (5.9) | 3.3 | 183 | 5.1 | 3.3 | 4.7 | 3.2 | 4.5 | 2.9 | 6.5 | 4.1 | 8.8 | 4.2 |
| 188 | Post-traumatic stress disorder | F431 | Cat I | 16,055 | 5.2 | (5.6) | 3.3 | 166 | 5.5 | 3.4 | 4.9 | 3.2 | 4.7 | 3.0 | 5.7 | 3.5 | 9.5 | 4.9 |
| 189 | Reactions to severe stress and adjustment disorders | F432–F439 | Cat III | 61,701 | 5.2 | (5.9) | 3.6 | 169 | 5.3 | 3.6 | 5.1 | 3.6 | 4.3 | 2.9 | 6.3 | 4.1 | 9.6 | 4.6 |
| 190 | Dissociative (conversion) disorders, somatoform disorders and other neurotic disorders | F44, F45, F48 | Cat I | 21,420 | 6.4 | (6.4) | 4.3 | 67 | 6.6 | 4.4 | 6.0 | 4.1 | 5.2 | 3.5 | 7.1 | 4.5 | 9.8 | 4.8 |
| 191 | Eating disorders | F50 | Cat III | 7,751 | 4.5 | (7.0) | 3.3 | 202 | 4.5 | 3.3 | 4.9 | 3.6 | 4.2 | 3.0 | 7.8 | 4.5 | 9.8 | 4.7 |
| 192 | Behavioural syndromes associated with physiological disturbances and physical factors | F51–F59 | Cat III | 6,163 | 4.5 | (5.3) | 3.6 | 200 | 4.7 | 3.7 | 4.3 | 3.5 | 3.7 | 2.9 | 5.7 | 4.1 | 9.5 | 4.2 |
| 193 | Emotionally unstable personality disorder | F603 | Cat I | 21,848 | 6.4 | (7.2) | 3.8 | 73 | 6.4 | 3.8 | 6.2 | 3.9 | 5.8 | 3.4 | 7.7 | 4.5 | 10.5 | 5.1 |
| 194 | Specific personality disorders | F602, F604–F609 | Cat I | 50,415 | 5.9 | (6.3) | 3.8 | 108 | 6.2 | 3.8 | 5.4 | 3.6 | 5.1 | 3.1 | 6.8 | 4.2 | 9.3 | 4.5 |
| 195 | Disorders of adult personality and behaviour | F61–F69 | Cat I | 17,533 | 6.2 | (6.7) | 3.9 | 85 | 6.6 | 4.0 | 5.7 | 3.7 | 5.6 | 3.4 | 7.0 | 4.3 | 9.8 | 4.8 |
| 196 | Mental retardation | F70–F79 | Cat I | 13,822 | 5.3 | (5.6) | 3.3 | 156 | 5.7 | 3.6 | 5.0 | 3.0 | 4.7 | 3.0 | 6.0 | 3.5 | 6.6 | 3.6 |
| 197 | Disorders of psychological development | F80–F89 | Cat I | 9,911 | 4.4 | (5.8) | 2.9 | 203 | 5.1 | 3.3 | 4.0 | 2.6 | 4.0 | 2.7 | 6.2 | 3.5 | 8.4 | 4.3 |
| 198 | Hyperkinetic disorders (ADHD) ^c^ | F90 | Cat I | 42,908 | 4.0 | (5.5) | 3.0 | 217 | 4.8 | 3.3 | 3.5 | 2.8 | 3.5 | 2.7 | 5.9 | 3.7 | 8.8 | 4.3 |
| 199 | Behavioural and emotional disorders with onset usually occurring in childhood and adolescence | F91–F99 | Cat I | 39,602 | 5.9 | (6.5) | 3.9 | 106 | 6.3 | 4.1 | 5.4 | 3.6 | 4.9 | 3.2 | 7.2 | 4.3 | 9.3 | 4.5 |
|  |  |  |  |  |  |  |  |  |  |  |  |  |  |  |  |  |  |  |
|  | **Having one or more chronic conditions** |  |  | **2,989,441** | **3.4** | **(3.1)** | **2.8** | **228** | **3.5** | **2.8** | **3.2** | **2.7** | **2.2** | **1.8** | **3.5** | **2.7** | **5.6** | **3.5** |
|  |  |  |  |  |  |  |  |  |  |  |  |  |  |  |  |  |  |  |
|  | Depression medicine ^c^ ** | ATC: N06A. | Cat IV | 529,918 | 4.8 | (4.4) | 3.7 | 189 | 4.9 | 3.6 | 4.7 | 3.7 | 3.1 | 2.6 | 5.2 | 3.6 | 7.6 | 3.9 |
|  | Antipsychotic medicine ^c^ ** | ATC: N05A. | Cat IV | 138,625 | 5.5 | (5.3) | 3.8 | 142 | 5.9 | 3.9 | 5.0 | 3.6 | 4.4 | 3.1 | 6.0 | 3.9 | 7.3 | 4.0 |
|  | Indication prescribed anxiety medicine ^c^ ** | All prescrib. w.indication codes 163 (for anxiety) or 371 (for anxiety, addictive) | Cat IV | 102,568 | 4.9 | (4.7) | 3.8 | 186 | 5.0 | 3.8 | 4.7 | 3.8 | 3.5 | 2.9 | 5.3 | 3.9 | 7.7 | 4.1 |
|  | Heart failure medication ^c^ ** | ATC: C01AA05, C03, C07 or C09A with indication code 430 (for heart failure) | Cat IV | 7,468 | 8.0 | (6.4) | 4.1 | 16 | 8.3 | 4.3 | 7.9 | 3.9 | 5.7 | 3.6 | 7.5 | 3.9 | 9.0 | 4.1 |
|  | Ischaemic heart medication ^c^ ** | ATC: C01A, C01B, C01D, C01E. | Cat IV | 129,484 | 7.4 | (5.6) | 4.1 | 35 | 7.6 | 4.1 | 7.2 | 4.0 | 4.7 | 3.8 | 6.9 | 4.0 | 8.0 | 4.0 |
|  | All five types of the medicine above |  |  | **688,006** | **5.1** | **(4.4)** | **3.7** | **178** | **5.1** | **3.7** | **5.0** | **3.7** | 3.2 | 2.6 | 5.3 | 3.6 | 7.4 | 3.9 |
|  |  |  |  |  |  |  |  |  |  |  |  |  |  |  |  |  |  |  |
|  | **Total population** |  |  | **4,555,439** | **2.2** | **(2.2)** | **2.8** | **n/a** | **2.4** | **2.9** | **2.0** | **2.6** | **1.1** | **1.6** | **2.7** | **2.8** | **5.3** | **3.6** |
|  |  |  |  |  |  |  |  |  |  |  |  |  |  |  |  |  |  |  |
|  | **Extra** |  |  |  |  |  |  |  |  |  |  |  |  |  |  |  |  |  |
|  | Ischaemic Heart Diseases broad | I05-I06; I11-I13; I20-I28; I30-I52 | Cat II+III | 315,901 | 6.8 | (5.2) | 3.8 | 57 | 7.1 | 3.9 | 6.6 | 3.7 | 4.0 | 3.0 | 6.5 | 3.6 | 8.1 | 3.8 |
|  | Artritis | M01-M03; M5-M9; M7-M14; M15-M20; M45 | Cat I+II | 505,792 | 5.4 | (4.3) | 3.6 | 149 | 5.6 | 3.6 | 5.1 | 3.5 | 3.4 | 2.5 | 5.1 | 3.4 | 7.3 | 3.8 |
|  | Arthrosis | M15-M19 | Cat I | 338,166 | 5.6 | (4.3) | 3.7 | 130 | 6.0 | 3.7 | 5.2 | 3.6 | 3.5 | 2.6 | 5.2 | 3.4 | 7.2 | 3.9 |
|  | Backconditions | M32-34;M41-M43;M46-49;M50-51;M53-M54 | Cat I,II,IV | 212,948 | 5.7 | (4.8) | 4.0 | 122 | 6.1 | 4.0 | 5.2 | 3.8 | 3.4 | 2.5 | 5.8 | 3.8 | 8.8 | 4.1 |
|  | Overweight, clinical (BMI >35) | E66 | Cat II | 220,928 | 3.9 | (4.3) | 3.7 | 222 | 3.5 | 3.5 | 5.3 | 4.0 | 2.2 | 2.5 | 5.3 | 3.8 | 8.1 | 4.3 |
|  | Endometriosis | N80 | Cat I | 29,190 | 2.9 | (2.0) | 2.9 | 231 | 2.9 | 2.9 | n/a | n/a | 2.2 | 2.4 | 3.3 | 3.0 | 7.0 | 3.9 |

Gender and age-standardised estimates (Std.) are in brackets.

ICD-10 International Statistical Classification of Diseases, 10^th^ Revision.

^c^ = complex defined conditions, see reference for further details [64].

Conditions marked ‘A’, overlap with other conditions and are thus not counted twice [64].

** 2-year prevalence. n/a: not available.
