# Supplementary material for: Catalogue of multimorbidity mean based severity and associational prevalence rates between 199+ chronic conditions—A nationwide register-based population study
Source: PLoS One. 2022 Sep 14;17(9):e0273850. doi: 10.1371/journal.pone.0273850 (PMC9473636; doi:10.1371/journal.pone.0273850)
Supplement: S3 Table — Number of patients, overall mean number of comorbidities and by sex and age in Denmark on 1 January 2013. Sorted by ICD-10 codes. (DOC) [file pone.0273850.s003.doc]

***S3 Table.*** Catalogue of mean NCCs and SDs of 29 common conditions and overweight: number of patients, overall mean number of comorbidities and by sex and age in Denmark on 1 January 2013. Sorted by ICD-10 codes.

| **Name of condition** | **ICD-10 code / definition** | **Category** | **Total Population** | | | | | **Sex and age** | | | | | | | | | |
| --- | --- | --- | --- | --- | --- | --- | --- | --- | --- | --- | --- | --- | --- | --- | --- | --- | --- |
|  |  |  |  |  |  |  |  | Female | | Male | | Age 16-44 | | Age 45-74 | | Age 75+ | |
|  |  |  |  | Means | | |  | Means | | Means | | Means | | Means | | Means | |
|  |  |  | *N* | *Raw* | *Std.* | *SD* | *Rank* | *Raw* | *SD* | *Raw* | *SD* | *Raw* | *SD* | *Raw* | *SD* | *Raw* | *SD* |
| Cancers | C00–C99; D32–D33; D35.2–D35.4; D42–D44 | Cat II | 229,331 | 5.4 | (4.2) | 3.6 | 20 | 5.4 | 3.6 | 5.5 | 3.6 | 3.2 | 2.4 | 5.0 | 3.4 | 7.0 | 3.8 |
| Diabetes type 1 ^c^ | E10 | Cat I | 23,062 | 4.7 | (4.8) | 3.2 | 26 | 5.1 | 3.4 | 4.3 | 3.0 | 3.2 | 2.5 | 5.5 | 3.2 | 7.4 | 3.4 |
| Diabetes type 2 ^c^ | E11 | Cat II | 242,177 | 6.2 | (5.1) | 3.6 | 10 | 6.4 | 3.6 | 6.0 | 3.5 | 4.2 | 3.0 | 5.9 | 3.4 | 7.5 | 3.7 |
| Migraine ^c^ | G43 | Cat II | 149,866 | 4.4 | (4.3) | 3.2 | 27 | 4.4 | 3.2 | 4.2 | 3.3 | 3.4 | 2.5 | 4.7 | 3.3 | 7.7 | 4.1 |
| Other headache syndromes | G44 | Cat III | 16,469 | 5.4 | (5.5) | 3.7 | 23 | 5.5 | 3.7 | 5.1 | 3.7 | 4.1 | 2.9 | 6.2 | 3.9 | 9.8 | 4.5 |
| Diseases of the eye lens (cataracts) | H25–H28 | Cat IV | 68,009 | 6.4 | (4.4) | 3.8 | 8 | 6.5 | 3.8 | 6.3 | 3.8 | 4.3 | 3.3 | 5.9 | 3.8 | 6.9 | 3.8 |
| Tinnitus | H931 | Cat I | 40,124 | 5.9 | (5.0) | 3.6 | 14 | 6.1 | 3.7 | 5.7 | 3.6 | 3.7 | 2.5 | 5.7 | 3.4 | 8.1 | 3.8 |
| Ischaemic Heart Diseases broad | I05-I06; I11-I13; I20-I28; I30-I52 | Cat II+III | 315,901 | 6.8 | (5.7) | 3.8 | 6 | 7.1 | 3.9 | 6.6 | 3.7 | 4.0 | 3.0 | 6.5 | 3.6 | 8.1 | 3.8 |
| Hypertensive diseases ^c^ | I10–I15 | Cat III | 1,060,046 | 5.1 | (4.3) | 3.3 | 24 | 5.1 | 3.3 | 5.0 | 3.3 | 3.7 | 2.7 | 4.7 | 3.1 | 6.3 | 3.6 |
| Heart failure ^c^ | I11.0, I13.0, I13.2, I42.0, I42.6, I42.7, I42.9, I50.0, I50.1, I50.9 | Cat II | 37,540 | 8.8 | (7.1) | 4.2 | 1 | 9.2 | 4.3 | 8.6 | 4.1 | 5.9 | 3.5 | 8.3 | 4.1 | 9.7 | 4.1 |
| Ischaemic heart diseases specifc | I20–I25 | Cat III | 139,173 | 7.9 | (6.6) | 4.0 | 2 | 8.4 | 4.2 | 7.6 | 3.8 | 5.2 | 3.6 | 7.3 | 3.8 | 9.3 | 3.9 |
| Angina pectoris | I20 | Cat III | 78,476 | 7.9 | (6.6) | 4.1 | 3 | 8.2 | 4.2 | 7.6 | 3.9 | 5.2 | 3.7 | 7.4 | 3.9 | 9.5 | 4.0 |
| Stroke | I60, I61,I63–I64, Z501 (rehabilitation) | Cat II | 72,606 | 7.5 | (6.0) | 3.9 | 4 | 7.9 | 3.9 | 7.3 | 3.8 | 5.3 | 3.4 | 7.1 | 3.8 | 8.4 | 3.8 |
| Respiratory allergy ^c^ | J30, except J30.0 | Cat I | 841,685 | 4.1 | (3.8) | 3.2 | 28 | 4.3 | 3.3 | 3.8 | 3.1 | 2.5 | 2.0 | 4.5 | 3.2 | 7.2 | 3.8 |
| Chronic lower respiratory diseases ^c^ | J40–J43, J47 | **Cat I** | 418,120 | 5.4 | (5.0) | 3.6 | 19 | 5.6 | 3.6 | 5.2 | 3.5 | 3.6 | 2.3 | 5.6 | 3.5 | 8.3 | 3.9 |
| Chronic obstructive lung disease (COPD) ^c^ | J44, J96, J13–J18 | Cat I | 216,184 | 6.5 | (5.6) | 3.9 | 7 | 6.7 | 3.9 | 6.3 | 3.9 | 3.9 | 2.6 | 6.5 | 3.7 | 8.5 | 3.9 |
| Asthma, status asthmaticus ^c^ | J45–J46 | Cat I | 361,129 | 5.4 | (5.1) | 3.6 | 21 | 5.7 | 3.6 | 5.0 | 3.5 | 3.5 | 2.2 | 6.0 | 3.5 | 8.6 | 3.8 |
| Artritis | M01-M03; M5-M9; M7-M14; M15-M20; M45 | Cat I+II | 505,792 | 5.4 | (4.5) | 3.6 | 22 | 5.6 | 3.6 | 5.1 | 3.5 | 3.4 | 2.5 | 5.1 | 3.4 | 7.3 | 3.8 |
| Inflammatory polyarthropathies and ankylosing spondylitis ^c^ | M05–M14, M45 | Cat I | 165,944 | 6.0 | (5.1) | 3.9 | 12 | 6.3 | 4.0 | 5.6 | 3.8 | 3.6 | 2.6 | 5.8 | 3.7 | 8.3 | 4.1 |
| Rheumatoid arthritis ^c^ | M05, M06, M07.1, M07.2, M07.3, M08, M09 | Cat I | 77,345 | 5.8 | (5.1) | 3.8 | 15 | 6.1 | 3.9 | 5.4 | 3.7 | 3.7 | 2.6 | 5.9 | 3.7 | 8.4 | 4.2 |
| Arthrosis | M15-M19 | Cat I | 338,166 | 5.6 | (4.5) | 3.7 | 18 | 6.0 | 3.7 | 5.2 | 3.6 | 3.5 | 2.6 | 5.2 | 3.4 | 7.2 | 3.9 |
| Gonarthrosis [arthrosis of knee] | M17 | Cat I | 178,811 | 5.6 | (4.4) | 3.7 | 17 | 6.0 | 3.7 | 5.2 | 3.6 | 3.4 | 2.6 | 5.2 | 3.4 | 7.5 | 3.9 |
| Backconditions | M32-34;M41-M43;M46-49;M50-51;M53-M54 | Cat I,II,IV | 212,948 | 5.7 | (5.1) | 4.0 | 16 | 6.1 | 4.0 | 5.2 | 3.8 | 3.4 | 2.5 | 5.8 | 3.8 | 8.8 | 4.1 |
| Osteoporosis ^c^ | M80–M81 | Cat I | 158,813 | 6.4 | (4.7) | 3.9 | 9 | 6.2 | 3.8 | 7.4 | 4.2 | 5.9 | 3.6 | 5.7 | 3.7 | 7.4 | 3.9 |
| Dementia ^c^ | F00, G30, F01, F02.0, F03.9, G31.8B, G31.8E, G31.9, G31.0B | Cat I | 36,803 | 7.4 | (4.7) | 3.8 | 5 | 7.3 | 3.8 | 7.5 | 3.9 | 6.5 | 3.7 | 7.4 | 4.1 | 7.3 | 3.7 |
| Schizophrenia ^c^ | F20 | Cat I | 29,422 | 5.9 | (6.2) | 3.7 | 13 | 6.7 | 4.1 | 5.3 | 3.3 | 5.5 | 3.3 | 6.2 | 4.0 | 7.3 | 4.1 |
| Depression ^c^ | F32, F33, F34.1, F06.32 | Cat III | 454,933 | 5.1 | (4.8) | 3.6 | 25 | 5.2 | 3.6 | 5.1 | 3.7 | 3.5 | 2.5 | 5.5 | 3.6 | 7.9 | 3.9 |
| Other anxiety disorders | F41 | Cat II | 38,079 | 6.1 | (6.3) | 3.9 | 11 | 6.2 | 3.9 | 6.0 | 3.9 | 4.9 | 3.1 | 7.2 | 4.2 | 9.9 | 4.5 |
| Hyperkinetic disorders (ADHD) ^c^ | F90 | Cat I | 42,908 | 4.0 | (5.0) | 3.0 | 29 | 4.8 | 3.3 | 3.5 | 2.8 | 3.5 | 2.7 | 5.9 | 3.7 | 8.8 | 4.3 |
| Overweight, clinical (BMI >35) | E66 | Cat II | 220,928 | 3.9 | (3.9) | 3.7 | 30 | 3.5 | 3.5 | 5.3 | 4.0 | 2.2 | 2.5 | 5.3 | 3.8 | 8.1 | 4.3 |
|  |  |  |  |  |  |  |  |  |  |  |  |  |  |  |  |  |  |
| **All the above conditions** | **-** |  | **2,564,764** | **3.7** | **(3.4**) | **2.9** | **n/a** | **3.7** | **2.9** | **3.6** | **2.8** | 2.4 | 1.9 | 3.8 | 2.8 | 5.8 | 3.5 |

Gender and age-standardised estimates (Std.) are in brackets.

ICD-10 International Statistical Classification of Diseases, 10^th^ Revision.

^c^ = complex defined conditions, see reference for further details [64].

n/a: not available.
