# Supplementary material for: Catalogue of multimorbidity mean based severity and associational prevalence rates between 199+ chronic conditions—A nationwide register-based population study
Source: PLoS One. 2022 Sep 14;17(9):e0273850. doi: 10.1371/journal.pone.0273850 (PMC9473636; doi:10.1371/journal.pone.0273850)
Supplement: S4 Table — Sorted by ICD-10 diagnosis. (DOCX) [file pone.0273850.s004.docx]

***S4 Table.*** Catalogue of correlational prevalence rates (per cent within conditions) of 199 chronic conditions, disease groups, medicines and overweight, by common conditions in Denmark on 1 January 2013. Sorted by ICD-10 diagnosis.

| **No.** | **Name of condition** | Cancers | Diabetes type 1 | Diabetes type 2 | Migraine | Other headache syndromes | Diseases of the eye lens (cataracts) | Tinnitus | Ischaemic heart diseases broad | Hypertensive diseases c | Heart failure | Ischaemic heart es specific | Angina pectoris | Stroke | Respiratory allergy c | Chronic lower respiratory diseases c | Chronic obstructive lung disease (COPD) c | Asthma, status asthmaticus | Artritis | Inflammatory polyarthropathies and ankylosing spondylitis c | Rheumatoid arthritis | Arthrosis | Gonarthrosis [arthrosis of knee] | Backconditions | Osteoporosis | Dementia | Schizophrenia | Depression | Other anxiety disorders | Hyperkinetic disorders (ADHD) c | Overweight, clinical (BMI >35) | Rank |
| --- | --- | --- | --- | --- | --- | --- | --- | --- | --- | --- | --- | --- | --- | --- | --- | --- | --- | --- | --- | --- | --- | --- | --- | --- | --- | --- | --- | --- | --- | --- | --- | --- |
|  | **B – Viral hepatitis and human immunodeficiency virus [HIV] disease** | 7.4 | 0.6 | 5.4 | 3.3 | 0.7 | 1.1 | 0.9 | 7.9 | 22.1 | 1.1 | 3.4 | 2.0 | 2.0 | 20.8 | 13.1 | 7.7 | 10.6 | 9.9 | 4.3 | 2.2 | 5.4 | 2.6 | 5.7 | 2.2 | 0.1 | 4.3 | 18.9 | 2.2 | 3.5 | 4.5 | 206 |
| 1 | Chronic viral hepatitis | 6.3 | 0.7 | 6.4 | 3.8 | 0.9 | 0.9 | 0.7 | 8.1 | 22.5 | 1.0 | 2.9 | 1.8 | 2.0 | 19.9 | 15.6 | 10.0 | 13.1 | 11.3 | 4.6 | 2.2 | 6.5 | 3.1 | 6.8 | 2.5 | 0.1 | 6.6 | 22.7 | 2.7 | 5.5 | 5.7 | 180 |
| 2 | Human immunodeficiency virus [HIV] disease | 8.6 | 0.6 | 4.1 | 2.5 | 0.6 | 1.4 | 1.1 | 8.1 | 21.2 | 1.2 | 4.0 | 2.3 | 1.9 | 22.2 | 10.8 | 5.8 | 8.1 | 8.6 | 4.1 | 2.1 | 4.7 | 2.2 | 4.3 | 1.9 | 0.2 | 2.3 | 14.8 | 1.5 | 1.2 | 3.1 | 219 |
|  | **C – Malignant neoplasms** | 100.0 | 0.5 | 11.2 | 4.6 | 0.4 | 4.2 | 1.9 | 17.9 | 49.7 | 2.3 | 7.8 | 4.0 | 4.0 | 23.9 | 14.8 | 10.7 | 11.3 | 22.2 | 7.5 | 3.0 | 15.8 | 8.1 | 7.9 | 10.5 | 2.1 | 0.5 | 14.9 | 0.9 | 0.4 | 5.9 | 147 |
| 3 | Malignant neoplasms of other and unspecified localizations | 100.0 | 0.6 | 10.5 | 3.8 | 0.5 | 3.9 | 2.1 | 16.0 | 45.4 | 2.2 | 7.1 | 3.6 | 3.8 | 24.3 | 14.7 | 11.1 | 11.3 | 19.9 | 7.3 | 2.9 | 13.7 | 7.2 | 8.1 | 7.5 | 1.4 | 0.7 | 15.4 | 1.3 | 0.5 | 5.3 | 111 |
| 4 | Malignant neoplasms of digestive organs | 100.0 | 0.8 | 14.2 | 2.9 | 0.5 | 4.2 | 1.7 | 24.7 | 57.0 | 2.9 | 11.5 | 5.7 | 5.1 | 24.3 | 16.1 | 13.0 | 12.3 | 25.0 | 9.1 | 2.8 | 17.7 | 8.0 | 9.0 | 10.1 | 1.9 | 0.7 | 18.0 | 1.3 | 0.5 | 5.8 | 58 |
| 5 | Malignant neoplasm of colon | 100.0 | 0.5 | 15.2 | 2.8 | 0.3 | 5.7 | 1.8 | 25.2 | 60.9 | 3.1 | 11.7 | 5.5 | 5.0 | 24.3 | 15.4 | 11.1 | 11.6 | 25.3 | 8.1 | 2.8 | 18.8 | 9.3 | 8.1 | 11.4 | 3.2 | 0.5 | 15.7 | 1.0 | 0.2 | 5.9 | 72 |
| 6 | Malignant neoplasms of rectosigmoid junction, rectum, anus and anal canal | 100.0 | 0.4 | 13.1 | 2.5 | 0.2 | 5.4 | 1.9 | 21.9 | 57.4 | 2.7 | 9.9 | 4.4 | 4.1 | 20.2 | 12.9 | 9.8 | 9.7 | 22.5 | 7.0 | 2.5 | 16.7 | 8.2 | 6.7 | 8.7 | 2.4 | 0.3 | 13.6 | 0.8 | 0.3 | 5.0 | 117 |
| 7 | Malignant neoplasm of bronchus and lung | 100.0 | 0.5 | 14.1 | 3.9 | 0.5 | 5.4 | 1.9 | 26.7 | 56.4 | 3.8 | 12.6 | 7.0 | 5.2 | 25.2 | 33.7 | 32.3 | 26.9 | 24.4 | 9.9 | 4.6 | 16.4 | 8.0 | 10.3 | 14.6 | 2.0 | 0.7 | 20.2 | 1.2 | 0.6 | 5.2 | 42 |
| 8 | Malignant melanoma of skin | 100.0 | 0.4 | 7.3 | 4.2 | 0.3 | 2.6 | 1.4 | 12.5 | 39.4 | 1.4 | 5.1 | 2.9 | 2.7 | 21.6 | 10.6 | 5.8 | 8.1 | 18.3 | 6.0 | 2.6 | 12.9 | 6.9 | 6.4 | 5.9 | 1.3 | 0.3 | 11.0 | 0.8 | 0.5 | 5.4 | 205 |
| 9 | Other malignant neoplasms of skin | 100.0 | 0.5 | 11.7 | 3.0 | 0.3 | 6.0 | 1.6 | 22.9 | 57.7 | 3.1 | 9.9 | 4.8 | 5.0 | 24.5 | 14.0 | 10.4 | 10.5 | 26.4 | 9.7 | 3.8 | 18.7 | 9.6 | 8.1 | 12.4 | 3.3 | 0.3 | 13.8 | 0.6 | 0.2 | 3.9 | 114 |
| 10 | Malignant neoplasm of breast | 100.0 | 0.4 | 9.6 | 8.5 | 0.3 | 4.1 | 1.3 | 13.2 | 48.0 | 1.5 | 5.0 | 2.7 | 2.9 | 26.4 | 15.0 | 9.0 | 11.3 | 22.7 | 5.8 | 3.3 | 16.9 | 9.0 | 7.5 | 17.0 | 2.0 | 0.5 | 16.9 | 1.0 | 0.3 | 6.2 | 168 |
| 11 | Malignant neoplasms of female genital organs | 100.0 | 0.7 | 8.9 | 6.7 | 0.4 | 3.6 | 1.3 | 13.9 | 45.9 | 1.5 | 5.5 | 2.5 | 2.7 | 25.1 | 14.5 | 8.5 | 11.5 | 20.2 | 5.7 | 3.2 | 14.6 | 7.5 | 7.3 | 11.3 | 1.5 | 0.6 | 16.6 | 1.2 | 0.5 | 12.7 | 160 |
| 12 | Malignant neoplasm of cervix uteri, corpus uteri and part unspecified | 100.0 | 0.5 | 12.7 | 5.9 | 0.4 | 3.8 | 1.3 | 13.6 | 49.8 | 1.3 | 5.5 | 2.7 | 2.4 | 24.7 | 13.0 | 6.8 | 9.7 | 21.5 | 5.8 | 3.1 | 16.2 | 8.8 | 6.5 | 9.5 | 2.0 | 0.6 | 15.4 | 1.2 | 0.4 | 19.5 | 181 |
| 13 | Malignant tumour of male genitalia | 100.0 | 0.5 | 5.4 | 1.5 | 0.3 | 0.9 | 1.8 | 8.1 | 20.6 | 1.1 | 4.0 | 2.3 | 1.5 | 17.1 | 8.3 | 4.2 | 7.1 | 10.2 | 4.2 | 1.5 | 6.3 | 3.2 | 4.4 | 1.2 | 0.5 | 0.7 | 9.0 | 0.7 | 0.7 | 2.2 | 226 |
| 14 | Malignant neoplasm of prostate | 100.0 | 0.3 | 13.8 | 1.7 | 0.1 | 5.1 | 2.5 | 23.8 | 59.9 | 3.2 | 11.3 | 6.0 | 5.1 | 22.1 | 13.1 | 11.1 | 9.7 | 25.2 | 9.0 | 2.4 | 18.6 | 9.9 | 8.6 | 4.8 | 2.4 | 0.1 | 11.4 | 0.5 | 0.2 | 2.8 | 139 |
| 15 | Malignant neoplasms of urinary tract | 100.0 | 0.5 | 16.3 | 2.5 | 0.4 | 4.9 | 2.2 | 25.1 | 62.3 | 3.5 | 11.9 | 5.8 | 5.6 | 21.4 | 15.3 | 13.6 | 12.3 | 24.8 | 9.9 | 2.7 | 16.8 | 8.3 | 8.6 | 8.4 | 2.4 | 0.4 | 14.7 | 0.7 | 0.3 | 5.5 | 83 |
| 16 | Brain cancer ^c^ | 100.0 | 0.6 | 10.5 | 5.1 | 1.5 | 3.4 | 5.1 | 14.5 | 43.4 | 1.8 | 6.5 | 3.4 | 6.3 | 24.9 | 14.3 | 9.8 | 10.9 | 18.6 | 6.1 | 2.4 | 13.0 | 6.7 | 9.5 | 8.3 | 1.9 | 0.8 | 17.6 | 1.4 | 0.7 | 6.2 | 84 |
| 17 | Malignant neoplasms of ill-defined, secondary and unspecified sites, and of independent (primary) multiple sites | 100.0 | 0.6 | 11.3 | 4.8 | 0.4 | 4.0 | 1.5 | 17.9 | 48.5 | 2.2 | 7.4 | 3.8 | 3.7 | 22.7 | 14.5 | 10.3 | 11.0 | 21.5 | 7.6 | 3.2 | 15.0 | 7.7 | 8.6 | 11.1 | 1.2 | 0.5 | 16.6 | 1.2 | 0.6 | 6.1 | 69 |
| 18 | Malignant neoplasms, stated or presumed to be primary, of lymphoid, haematopoietic and related tissue | 100.0 | 0.7 | 10.6 | 3.4 | 0.5 | 4.3 | 1.7 | 20.1 | 45.6 | 3.4 | 8.0 | 3.9 | 3.8 | 24.0 | 13.5 | 11.0 | 10.3 | 23.7 | 11.0 | 3.7 | 14.8 | 7.2 | 9.2 | 10.9 | 1.9 | 0.6 | 15.3 | 1.0 | 0.6 | 4.6 | 116 |
|  | **D – In situ and benign neoplasms, and neoplasms of uncertain or unknown behaviour and diseases of the blood and blood-forming organs and certain disorders involving the immune mechanism** | 21.0 | 0.8 | 14.1 | 5.0 | 0.7 | 4.1 | 1.4 | 25.1 | 51.4 | 4.4 | 11.6 | 5.7 | 6.9 | 26.0 | 17.6 | 12.6 | 14.1 | 25.6 | 11.2 | 4.6 | 16.7 | 8.6 | 9.9 | 12.5 | 3.8 | 1.0 | 20.6 | 1.6 | 0.8 | 9.7 | 66 |
| 19 | In situ neoplasms | 34.5 | 0.5 | 8.3 | 5.9 | 0.4 | 2.7 | 1.4 | 11.7 | 38.2 | 1.4 | 4.9 | 2.7 | 2.3 | 24.6 | 13.8 | 7.8 | 10.4 | 18.1 | 5.8 | 3.0 | 12.3 | 6.4 | 7.1 | 7.6 | 1.2 | 0.5 | 14.9 | 1.1 | 0.6 | 10.2 | 191 |
| 20 | Haemolytic anaemias | 13.1 | 0.7 | 10.4 | 4.8 | 0.7 | 3.0 | 1.4 | 18.1 | 36.9 | 3.5 | 7.6 | 4.0 | 3.4 | 25.7 | 12.5 | 8.1 | 10.9 | 20.1 | 10.3 | 4.9 | 11.4 | 5.7 | 8.8 | 9.6 | 1.5 | 1.0 | 15.2 | 1.4 | 0.8 | 9.7 | 141 |
| 21 | Aplastic and other anaemias | 30.3 | 1.0 | 17.9 | 4.1 | 0.5 | 5.3 | 1.6 | 34.1 | 64.1 | 6.5 | 17.0 | 7.4 | 8.1 | 26.1 | 17.7 | 14.7 | 13.6 | 33.5 | 14.8 | 5.6 | 23.5 | 12.1 | 11.3 | 16.4 | 5.9 | 1.0 | 23.4 | 1.6 | 0.6 | 9.6 | 14 |
| 22 | Other anaemias | 21.5 | 1.0 | 20.6 | 4.0 | 0.5 | 6.0 | 1.5 | 35.7 | 67.0 | 7.2 | 18.1 | 8.4 | 9.4 | 27.0 | 19.8 | 16.7 | 15.7 | 32.1 | 14.5 | 5.4 | 21.9 | 11.1 | 11.8 | 17.9 | 6.9 | 1.3 | 26.4 | 1.8 | 0.6 | 8.8 | 13 |
| 23 | Coagulation defects, purpura and other haemorrhagic conditions | 10.4 | 0.8 | 9.0 | 6.1 | 1.0 | 2.2 | 1.2 | 21.6 | 38.2 | 3.0 | 7.8 | 4.1 | 8.5 | 24.5 | 13.8 | 8.5 | 11.9 | 19.7 | 8.9 | 4.4 | 11.8 | 6.2 | 9.2 | 6.7 | 1.3 | 0.8 | 17.1 | 1.6 | 1.1 | 11.5 | 134 |
| 24 | Other diseases of blood and blood-forming organs | 23.7 | 0.7 | 12.8 | 5.7 | 0.9 | 3.3 | 1.6 | 21.5 | 50.0 | 3.5 | 9.1 | 4.8 | 6.1 | 28.1 | 19.9 | 14.7 | 16.8 | 25.5 | 14.4 | 5.9 | 13.5 | 7.0 | 10.7 | 11.2 | 1.7 | 1.6 | 19.6 | 1.9 | 0.9 | 8.9 | 61 |
| 25 | Certain disorders involving the immune mechanism | 14.7 | 1.1 | 9.8 | 5.5 | 0.9 | 2.6 | 1.5 | 15.7 | 35.6 | 2.2 | 6.4 | 3.9 | 2.5 | 30.3 | 29.9 | 16.1 | 24.6 | 23.7 | 13.0 | 5.4 | 11.3 | 6.0 | 8.8 | 12.9 | 0.6 | 0.8 | 15.5 | 1.3 | 1.1 | 9.8 | 118 |
|  | **E – Endocrine, nutritional and metabolic diseases** | 10.6 | 2.6 | 27.6 | 4.1 | 0.4 | 4.0 | 1.6 | 22.5 | 63.8 | 3.2 | 13.1 | 7.3 | 6.4 | 24.4 | 14.6 | 9.9 | 11.5 | 22.7 | 8.1 | 2.9 | 15.8 | 8.5 | 8.1 | 8.3 | 2.1 | 0.8 | 16.2 | 1.0 | 0.5 | 8.7 | 161 |
| 26 | Diseases of the thyroid ^c^ | 11.9 | 1.4 | 11.9 | 6.1 | 0.5 | 3.4 | 1.5 | 13.7 | 46.0 | 1.8 | 6.3 | 3.6 | 3.0 | 28.5 | 15.7 | 9.0 | 12.5 | 21.6 | 7.1 | 3.6 | 14.9 | 8.0 | 8.2 | 10.2 | 2.1 | 0.7 | 18.2 | 1.4 | 0.6 | 9.5 | 174 |
| 27 | Thyrotoxicosis ^c^ | 10.6 | 1.0 | 11.9 | 5.5 | 0.5 | 3.7 | 1.2 | 21.6 | 55.6 | 3.1 | 7.5 | 3.9 | 4.0 | 25.1 | 15.8 | 11.2 | 12.8 | 19.4 | 6.2 | 3.2 | 13.9 | 7.3 | 7.4 | 13.3 | 2.9 | 0.7 | 17.0 | 1.2 | 0.4 | 7.7 | 159 |
| 28 | Diabetes type 1 ^c^ | 4.8 | 100.0 | 0.0 | 2.3 | 0.3 | 4.1 | 0.8 | 9.7 | 44.8 | 1.3 | 5.7 | 3.3 | 2.7 | 19.3 | 9.0 | 4.3 | 7.7 | 12.2 | 5.8 | 2.5 | 6.6 | 2.8 | 4.6 | 4.2 | 0.8 | 0.9 | 13.2 | 0.8 | 1.1 | 5.6 | 197 |
| 29 | Diabetes type 2 ^c^ | 10.6 | 0.0 | 100.0 | 3.1 | 0.4 | 5.0 | 1.7 | 23.4 | 75.4 | 4.2 | 12.9 | 7.1 | 5.4 | 24.7 | 16.5 | 11.2 | 12.9 | 26.3 | 11.2 | 3.0 | 17.8 | 10.2 | 8.6 | 6.6 | 2.3 | 1.2 | 17.3 | 1.1 | 0.4 | 16.2 | 86 |
| 30 | Diabetes others ^c^ | 11.7 | 0.0 | 0.0 | 2.9 | 0.5 | 3.8 | 1.5 | 24.1 | 52.2 | 4.9 | 11.3 | 6.3 | 5.8 | 25.0 | 15.8 | 11.4 | 13.9 | 23.1 | 11.1 | 3.9 | 13.8 | 7.1 | 10.1 | 9.8 | 2.6 | 2.1 | 19.4 | 1.3 | 0.4 | 20.1 | 80 |
| 31 | Disorders of other endocrine glands | 17.3 | 1.4 | 16.9 | 5.9 | 1.1 | 2.6 | 1.2 | 16.1 | 39.7 | 2.4 | 8.4 | 4.8 | 3.3 | 25.4 | 14.4 | 8.5 | 12.7 | 17.6 | 7.5 | 3.3 | 11.1 | 5.7 | 8.5 | 12.5 | 1.6 | 1.3 | 19.2 | 2.0 | 1.4 | 17.8 | 128 |
| 32 | Metabolic disorders | 16.7 | 1.5 | 12.8 | 5.9 | 0.9 | 3.1 | 1.5 | 17.9 | 45.3 | 2.5 | 4.3 | 4.7 | 4.6 | 27.6 | 16.9 | 11.3 | 14.2 | 22.7 | 9.8 | 4.2 | 14.4 | 7.3 | 10.1 | 10.8 | 2.0 | 2.0 | 20.4 | 2.0 | 1.0 | 11.9 | 74 |
| 33 | Disturbances in lipoprotein circulation and other lipids ^c^ | 10.7 | 1.6 | 26.6 | 3.7 | 0.4 | 4.5 | 1.8 | 26.7 | 71.9 | 3.8 | 16.7 | 9.3 | 8.1 | 24.2 | 14.7 | 10.6 | 11.4 | 24.3 | 8.6 | 2.8 | 17.1 | 9.2 | 8.5 | 8.4 | 2.1 | 0.7 | 16.2 | 0.9 | 0.3 | 7.9 | 131 |
| 34 | Cystic fibrosis ^c^ | 5.8 | 9.4 | 6.2 | 4.5 | 0.0 | 1.4 | 0.0 | 7.5 | 23.9 | 1.2 | 2.2 | 1.6 | 2.0 | 31.4 | 0.0 | 0.0 | 0.0 | 30.1 | 28.3 | 5.8 | 5.5 | 2.7 | 5.8 | 6.4 | 0.5 | 0.0 | 13.6 | 1.5 | 1.7 | 7.5 | 211 |
|  | **G – Diseases of the nervous system** | 8.7 | 0.8 | 9.9 | 26.7 | 2.9 | 2.6 | 1.6 | 13.3 | 42.2 | 1.6 | 6.2 | 3.6 | 5.1 | 27.2 | 15.0 | 8.5 | 12.1 | 20.5 | 6.5 | 3.1 | 14.5 | 7.5 | 11.0 | 6.8 | 2.6 | 2.5 | 21.6 | 1.8 | 1.6 | 9.1 | 172 |
| 35 | Inflammatory diseases of the central nervous system | 11.0 | 0.7 | 9.8 | 6.1 | 2.1 | 2.5 | 2.3 | 14.1 | 35.4 | 1.6 | 5.4 | 2.7 | 6.8 | 24.8 | 13.7 | 8.3 | 10.8 | 17.7 | 6.9 | 3.4 | 11.5 | 6.2 | 12.4 | 7.0 | 2.4 | 1.2 | 19.4 | 1.7 | 1.3 | 7.1 | 127 |
| 36 | Systemic atrophies primarily affecting the central nervous system and other degenerative diseases | 11.8 | 0.6 | 12.3 | 3.0 | 0.6 | 4.0 | 1.8 | 21.4 | 53.2 | 2.9 | 8.6 | 3.2 | 8.3 | 25.9 | 14.4 | 11.6 | 10.3 | 22.2 | 7.0 | 2.8 | 16.6 | 8.2 | 11.9 | 13.1 | 55.3 | 1.0 | 41.1 | 1.7 | 0.8 | 3.7 | 41 |
| 37 | Parkinson’s disease ^c^ | 10.1 | 0.7 | 14.9 | 6.0 | 0.6 | 4.2 | 1.6 | 16.7 | 49.4 | 2.3 | 8.2 | 4.4 | 4.7 | 29.0 | 18.7 | 13.1 | 15.6 | 23.1 | 6.8 | 2.8 | 17.2 | 9.2 | 11.7 | 10.6 | 6.2 | 19.3 | 36.0 | 3.3 | 2.0 | 10.0 | 49 |
| 38 | Extrapyramidal and movement disorders | 10.4 | 0.8 | 10.3 | 8.1 | 4.5 | 4.1 | 1.9 | 17.4 | 49.8 | 2.3 | 8.2 | 4.4 | 5.7 | 29.9 | 18.4 | 12.0 | 14.6 | 22.6 | 7.5 | 3.3 | 16.4 | 8.3 | 15.5 | 10.6 | 5.3 | 2.7 | 30.3 | 3.1 | 2.1 | 7.1 | 55 |
| 39 | Sclerosis | 6.0 | 0.7 | 5.2 | 6.5 | 0.7 | 1.6 | 1.2 | 7.4 | 30.7 | 0.7 | 3.0 | 1.7 | 3.5 | 23.9 | 11.5 | 5.5 | 8.5 | 10.8 | 3.5 | 2.1 | 6.9 | 3.3 | 8.1 | 7.4 | 0.8 | 0.6 | 22.7 | 1.2 | 6.6 | 6.6 | 196 |
| 40 | Demyelinating diseases of the central nervous system | 7.1 | 0.7 | 5.6 | 7.4 | 1.2 | 1.3 | 1.5 | 7.9 | 29.3 | 0.8 | 3.5 | 2.2 | 4.0 | 24.9 | 12.4 | 4.8 | 9.9 | 11.4 | 4.0 | 2.3 | 7.1 | 3.7 | 10.7 | 5.1 | 0.8 | 1.0 | 21.8 | 2.1 | 4.5 | 8.0 | 157 |
| 41 | Epilepsy ^c^ | 10.0 | 0.8 | 9.7 | 7.3 | 1.4 | 2.7 | 1.4 | 14.0 | 38.8 | 1.8 | 6.0 | 3.1 | 9.4 | 25.8 | 14.8 | 9.6 | 12.8 | 16.4 | 5.4 | 2.3 | 11.3 | 5.8 | 9.7 | 7.4 | 3.9 | 5.4 | 33.3 | 3.7 | 3.2 | 8.0 | 87 |
| 42 | Migraine ^c^ | 7.0 | 0.4 | 5.1 | 100.0 | 3.9 | 1.4 | 1.3 | 7.2 | 39.6 | 0.5 | 3.2 | 2.2 | 1.9 | 31.2 | 15.4 | 6.4 | 12.6 | 15.8 | 4.7 | 3.0 | 10.5 | 5.5 | 9.0 | 5.2 | 0.5 | 0.7 | 20.9 | 1.9 | 1.4 | 9.7 | 204 |
| 43 | Other headache syndromes | 6.0 | 0.5 | 5.4 | 35.8 | 100.0 | 1.6 | 2.1 | 9.6 | 39.6 | 0.7 | 5.0 | 3.6 | 4.1 | 29.9 | 14.2 | 6.6 | 12.2 | 15.0 | 4.9 | 2.9 | 9.9 | 5.1 | 12.9 | 3.9 | 0.6 | 0.9 | 26.0 | 3.5 | 1.7 | 9.7 | 153 |
| 44 | Transient cerebral ischaemic attacks and related syndromes and vascular syndromes of brain in cerebrovascular diseases | 12.5 | 0.7 | 6.0 | 5.5 | 0.9 | 4.9 | 2.2 | 30.2 | 67.4 | 3.5 | 13.9 | 7.4 | 22.0 | 25.1 | 15.2 | 11.3 | 11.7 | 25.4 | 8.8 | 3.2 | 18.4 | 9.5 | 10.1 | 10.5 | 4.3 | 0.4 | 20.5 | 1.3 | 0.4 | 5.8 | 44 |
| 45 | Sleep disorders | 7.6 | 0.7 | 17.0 | 5.0 | 1.1 | 2.0 | 2.2 | 17.5 | 48.1 | 2.6 | 9.5 | 6.1 | 3.3 | 36.6 | 16.5 | 10.7 | 14.6 | 22.0 | 8.4 | 2.6 | 15.1 | 8.7 | 9.9 | 3.6 | 0.8 | 0.9 | 23.3 | 2.1 | 4.9 | 20.3 | 137 |
| 46 | Disorders of trigeminal nerve and facial nerve disorders | 9.7 | 0.7 | 11.1 | 7.1 | 2.4 | 2.9 | 2.3 | 13.4 | 41.0 | 1.6 | 6.8 | 4.1 | 4.6 | 28.3 | 15.0 | 8.1 | 11.4 | 19.4 | 6.6 | 3.1 | 13.5 | 7.0 | 10.1 | 6.7 | 1.5 | 0.8 | 18.0 | 1.6 | 0.9 | 8.8 | 162 |
| 47 | Disorders of other cranial nerves, cranial nerve disorders in diseases classified elsewhere, nerve root and plexus disorders and nerve root and plexus compressions in diseases classified elsewhere | 10.8 | 0.8 | 10.1 | 6.9 | 1.2 | 2.4 | 2.0 | 13.7 | 41.0 | 1.5 | 6.9 | 4.4 | 3.6 | 27.4 | 16.0 | 9.1 | 12.4 | 24.1 | 7.5 | 3.3 | 17.8 | 8.9 | 28.2 | 6.3 | 1.1 | 0.7 | 18.5 | 1.4 | 0.7 | 8.4 | 100 |
| 48 | Mononeuropathies of upper limb | 7.9 | 1.4 | 11.6 | 6.1 | 0.7 | 2.6 | 1.6 | 12.2 | 41.2 | 1.4 | 6.3 | 3.9 | 2.7 | 26.3 | 15.9 | 8.7 | 12.6 | 28.2 | 8.3 | 4.3 | 21.5 | 10.9 | 13.4 | 6.8 | 0.9 | 0.5 | 18.0 | 1.1 | 1.0 | 11.4 | 171 |
| 49 | Mononeuropathies of lower limb, other mononeuropathies and mononeuropathy in diseases classified elsewhere | 9.0 | 0.8 | 10.0 | 6.9 | 0.8 | 2.5 | 1.9 | 13.1 | 40.4 | 1.5 | 6.4 | 3.9 | 3.0 | 27.5 | 16.2 | 9.0 | 13.0 | 31.3 | 8.6 | 4.4 | 22.2 | 11.3 | 16.1 | 7.8 | 1.0 | 0.6 | 17.4 | 1.3 | 0.8 | 8.7 | 124 |
| 50 | Polyneuropathies and other disorders of the peripheral nervous system | 14.1 | 2.7 | 19.9 | 4.6 | 0.7 | 5.0 | 2.2 | 22.3 | 56.5 | 3.3 | 10.9 | 6.1 | 6.2 | 27.8 | 17.9 | 13.0 | 13.6 | 30.9 | 12.5 | 4.8 | 21.4 | 10.9 | 19.5 | 11.5 | 2.8 | 0.9 | 23.4 | 1.6 | 0.8 | 8.6 | 38 |
| 51 | Diseases of myoneural junction and muscle | 9.0 | 1.0 | 11.1 | 5.3 | 0.7 | 3.3 | 1.5 | 17.7 | 39.7 | 3.4 | 7.0 | 3.4 | 3.5 | 25.2 | 15.0 | 10.3 | 12.1 | 19.2 | 8.6 | 4.3 | 11.9 | 5.8 | 17.2 | 11.6 | 1.0 | 0.8 | 16.7 | 1.3 | 1.1 | 8.9 | 113 |
| 52 | Cerebral palsy and other paralytic syndromes | 8.9 | 0.7 | 8.2 | 5.0 | 0.9 | 2.0 | 1.0 | 11.5 | 33.6 | 1.6 | 5.0 | 2.6 | 8.0 | 23.0 | 13.3 | 8.3 | 11.5 | 17.6 | 5.6 | 2.5 | 11.6 | 5.3 | 22.2 | 6.7 | 1.5 | 1.5 | 23.1 | 1.5 | 1.9 | 7.6 | 101 |
| 53 | Other disorders of the nervous system | 13.6 | 0.8 | 9.9 | 7.3 | 2.3 | 2.9 | 2.2 | 18.2 | 43.7 | 2.4 | 8.2 | 3.9 | 11.9 | 26.4 | 14.5 | 9.3 | 12.1 | 20.1 | 7.3 | 3.1 | 13.7 | 6.9 | 14.7 | 7.3 | 4.2 | 1.5 | 24.4 | 2.4 | 1.7 | 7.8 | 70 |
|  | **H – Diseases of the eye and adnexa and diseases of the ear and mastoid process** | 12.2 | 2.0 | 14.7 | 3.7 | 0.6 | 15.2 | 9.0 | 18.8 | 53.1 | 2.6 | 8.8 | 4.8 | 4.9 | 26.6 | 14.9 | 10.3 | 11.8 | 23.8 | 7.7 | 3.2 | 17.4 | 9.1 | 8.6 | 10.5 | 3.1 | 0.5 | 15.7 | 0.9 | 0.5 | 5.5 | 129 |
| 54 | Disorders of eyelid, lacrimal system and orbit | 12.1 | 0.8 | 10.9 | 5.3 | 0.8 | 12.8 | 1.8 | 15.7 | 47.0 | 1.6 | 7.5 | 4.5 | 3.6 | 31.3 | 14.9 | 9.3 | 11.8 | 22.5 | 6.9 | 3.0 | 16.2 | 8.5 | 9.1 | 9.8 | 1.4 | 0.4 | 16.2 | 1.1 | 0.6 | 6.1 | 138 |
| 55 | Corneal scars and opacities | 10.1 | 1.0 | 12.2 | 3.8 | 0.5 | 14.8 | 1.6 | 16.0 | 41.4 | 2.2 | 7.9 | 4.0 | 3.9 | 25.2 | 13.7 | 8.6 | 12.1 | 20.1 | 7.9 | 3.2 | 13.8 | 7.4 | 8.5 | 8.4 | 1.7 | 0.9 | 15.0 | 1.0 | 0.8 | 5.6 | 135 |
| 56 | Other disorders of cornea | 10.4 | 1.6 | 10.9 | 4.0 | 0.5 | 14.4 | 1.6 | 16.8 | 45.6 | 2.2 | 7.7 | 4.3 | 3.9 | 28.8 | 15.1 | 9.3 | 12.6 | 21.4 | 7.0 | 3.5 | 15.5 | 8.2 | 7.8 | 10.9 | 2.7 | 0.5 | 15.2 | 0.8 | 0.6 | 5.2 | 133 |
| 57 | Diseases of the eye lens (cataracts) | 14.2 | 1.4 | 17.9 | 3.1 | 0.4 | 100.0 | 1.9 | 23.6 | 64.0 | 3.5 | 11.1 | 6.0 | 5.4 | 26.6 | 17.4 | 14.0 | 13.6 | 28.3 | 9.1 | 3.7 | 21.3 | 11.0 | 9.3 | 14.9 | 2.9 | 0.4 | 15.5 | 0.7 | 0.2 | 5.0 | 68 |
| 58 | Disorders of the choroid and retina | 16.0 | 1.6 | 11.5 | 3.9 | 0.8 | 7.6 | 2.4 | 16.3 | 44.3 | 2.2 | 7.5 | 4.0 | 3.6 | 26.5 | 14.3 | 8.7 | 11.1 | 20.2 | 7.2 | 3.4 | 13.8 | 6.9 | 8.1 | 8.5 | 2.1 | 0.3 | 14.7 | 0.0 | 0.8 | 5.9 | 136 |
| 59 | Retinal vascular occlusions | 13.4 | 1.1 | 18.7 | 2.8 | 0.4 | 9.2 | 2.2 | 25.9 | 71.9 | 3.5 | 12.1 | 6.3 | 8.9 | 26.2 | 14.7 | 11.4 | 11.5 | 26.7 | 9.6 | 3.3 | 19.1 | 10.1 | 9.3 | 11.7 | 3.7 | 0.3 | 15.9 | 0.8 | 0.2 | 5.3 | 52 |
| 60 | Other retinal disorders | 14.1 | 1.7 | 15.9 | 3.0 | 0.4 | 14.7 | 2.1 | 24.5 | 65.7 | 3.6 | 11.1 | 5.6 | 6.3 | 27.8 | 16.6 | 12.9 | 12.6 | 27.9 | 9.3 | 3.8 | 20.7 | 10.7 | 9.5 | 15.5 | 4.6 | 0.3 | 17.2 | 0.8 | 0.3 | 4.7 | 65 |
| 61 | Retinal disorders in diseases classified elsewhere | 8.8 | 32.4 | 65.3 | 2.5 | 0.4 | 9.2 | 1.3 | 23.1 | 73.0 | 4.5 | 14.8 | 8.5 | 6.2 | 22.1 | 12.8 | 8.2 | 10.5 | 22.2 | 10.4 | 2.9 | 13.5 | 7.1 | 7.5 | 5.3 | 1.7 | 0.9 | 17.1 | 0.9 | 0.7 | 17.9 | 40 |
| 62 | Glaucoma ^c^ | 12.6 | 1.3 | 16.8 | 3.1 | 0.4 | 11.9 | 1.8 | 19.5 | 60.1 | 2.3 | 8.6 | 4.6 | 4.9 | 27.6 | 14.7 | 10.6 | 11.4 | 24.8 | 7.9 | 3.3 | 18.3 | 9.4 | 8.5 | 12.7 | 3.5 | 0.4 | 15.4 | 0.7 | 0.3 | 4.5 | 112 |
| 63 | Disorders of the vitreous body and globe | 9.6 | 4.2 | 14.1 | 4.5 | 0.6 | 20.9 | 2.0 | 15.4 | 45.1 | 2.0 | 7.4 | 4.3 | 3.8 | 26.4 | 13.2 | 7.6 | 10.5 | 21.5 | 7.4 | 3.3 | 14.9 | 7.5 | 8.0 | 8.1 | 0.9 | 0.4 | 13.2 | 0.9 | 0.5 | 6.2 | 126 |
| 64 | Disorders of optic nerve and visual pathways | 9.9 | 1.0 | 8.3 | 6.2 | 1.8 | 4.2 | 1.1 | 10.2 | 32.4 | 1.2 | 4.6 | 2.7 | 3.9 | 24.6 | 13.5 | 6.6 | 10.6 | 14.3 | 5.5 | 2.5 | 8.8 | 4.4 | 8.0 | 5.6 | 1.0 | 0.9 | 18.1 | 1.6 | 3.1 | 9.3 | 151 |
| 65 | Disorders of ocular muscles, binocular movement, accommodation and refraction | 6.2 | 0.7 | 5.5 | 5.2 | 1.0 | 5.3 | 1.3 | 7.0 | 24.2 | 0.7 | 2.8 | 1.7 | 3.3 | 24.3 | 11.0 | 5.0 | 9.7 | 12.4 | 4.4 | 2.3 | 7.7 | 4.0 | 6.5 | 3.9 | 0.7 | 0.8 | 14.0 | 1.1 | 1.4 | 6.5 | 213 |
| 66 | Visual disturbances | 13.3 | 0.9 | 11.2 | 6.4 | 1.4 | 6.4 | 2.2 | 17.1 | 45.3 | 2.2 | 7.9 | 4.6 | 10.8 | 27.0 | 15.0 | 9.2 | 12.2 | 21.0 | 7.5 | 3.5 | 14.5 | 7.5 | 9.4 | 9.5 | 2.4 | 1.0 | 19.2 | 1.6 | 1.1 | 7.0 | 91 |
| 67 | Blindness and partial sight | 15.3 | 2.0 | 14.2 | 5.3 | 1.0 | 7.4 | 2.2 | 22.9 | 52.7 | 3.4 | 11.4 | 5.9 | 9.3 | 29.1 | 18.2 | 11.4 | 14.3 | 28.2 | 8.7 | 4.1 | 21.7 | 9.2 | 12.2 | 13.0 | 6.4 | 1.8 | 26.1 | 2.9 | 1.1 | 9.0 | 26 |
| 68 | Nystagmus and other irregular eye movements and other disorders of eye and adnexa | 11.4 | 2.8 | 10.7 | 6.8 | 1.9 | 6.8 | 1.5 | 14.1 | 39.6 | 1.8 | 6.8 | 4.2 | 4.8 | 28.9 | 14.1 | 8.0 | 11.8 | 20.3 | 8.3 | 4.8 | 12.9 | 6.7 | 9.8 | 7.7 | 1.2 | 0.9 | 17.0 | 1.7 | 1.3 | 7.2 | 120 |
| 69 | Otosclerosis | 9.4 | 0.5 | 8.1 | 4.8 | 0.5 | 3.3 | 9.1 | 12.8 | 40.5 | 1.5 | 5.9 | 3.3 | 2.9 | 26.5 | 13.6 | 8.0 | 10.7 | 19.7 | 5.9 | 2.8 | 14.2 | 7.4 | 7.7 | 9.1 | 1.9 | 0.4 | 14.4 | 0.9 | 0.6 | 5.2 | 164 |
| 70 | Ménière’s disease ^c^ | 11.4 | 0.5 | 11.4 | 6.2 | 0.7 | 4.2 | 16.3 | 18.4 | 52.7 | 2.2 | 8.8 | 5.2 | 4.3 | 32.4 | 16.3 | 10.0 | 12.7 | 24.2 | 7.6 | 3.4 | 17.9 | 9.5 | 10.0 | 10.0 | 2.5 | 0.2 | 17.6 | 1.3 | 0.2 | 5.8 | 88 |
| 71 | Other diseases of the inner ear | 13.1 | 0.5 | 13.9 | 2.1 | 0.4 | 4.6 | 23.1 | 21.7 | 53.3 | 2.7 | 11.1 | 6.0 | 4.8 | 23.1 | 13.9 | 11.2 | 11.0 | 26.3 | 8.5 | 2.5 | 19.8 | 10.8 | 9.5 | 4.6 | 2.5 | 0.2 | 12.3 | 0.5 | 0.3 | 3.7 | 75 |
| 72 | Conductive and sensorineural hearing loss | 12.1 | 0.6 | 11.3 | 3.8 | 0.6 | 4.4 | 9.6 | 16.3 | 45.1 | 2.2 | 7.9 | 4.4 | 4.0 | 27.2 | 14.3 | 9.9 | 11.6 | 21.6 | 6.6 | 2.7 | 16.0 | 8.5 | 7.8 | 8.7 | 2.4 | 0.5 | 14.7 | 0.9 | 0.7 | 5.2 | 110 |
| 73 | Other hearing loss and other disorders of ear, not elsewhere classified | 15.1 | 0.8 | 10.7 | 3.9 | 0.7 | 3.8 | 20.1 | 17.0 | 45.7 | 2.3 | 8.1 | 4.2 | 5.0 | 25.8 | 13.8 | 8.9 | 11.1 | 20.3 | 6.6 | 2.7 | 14.8 | 7.9 | 8.3 | 8.1 | 2.3 | 0.7 | 16.3 | 1.2 | 0.8 | 5.4 | 81 |
| 74 | Presbycusis (age-related hearing loss) | 16.2 | 0.4 | 15.0 | 2.1 | 0.3 | 8.1 | 8.2 | 28.6 | 67.7 | 4.1 | 12.8 | 6.2 | 6.9 | 28.2 | 17.0 | 13.7 | 12.8 | 30.8 | 8.9 | 3.2 | 24.1 | 12.4 | 9.6 | 15.5 | 7.0 | 0.2 | 17.8 | 0.6 | 0.1 | 3.3 | 45 |
| 75 | Hearing loss, unspecified | 13.8 | 0.6 | 13.1 | 3.5 | 0.5 | 5.4 | 15.5 | 19.8 | 52.8 | 2.7 | 9.6 | 5.3 | 4.9 | 27.9 | 15.3 | 10.7 | 11.9 | 25.4 | 7.8 | 3.0 | 19.0 | 10.1 | 9.2 | 9.9 | 3.4 | 0.5 | 15.9 | 0.9 | 0.4 | 5.0 | 77 |
| 76 | Tinnitus | 10.9 | 0.4 | 10.0 | 5.0 | 0.9 | 3.3 | 100.0 | 14.7 | 44.3 | 1.6 | 7.3 | 4.5 | 3.2 | 29.6 | 14.5 | 8.7 | 11.8 | 22.3 | 6.9 | 2.9 | 16.4 | 8.9 | 10.1 | 6.9 | 1.6 | 0.7 | 16.8 | 1.4 | 0.7 | 5.3 | 107 |
| 77 | Other specified disorders of ear | 11.9 | 0.6 | 12.2 | 3.9 | 0.4 | 4.5 | 13.2 | 17.5 | 50.2 | 2.1 | 8.6 | 4.6 | 4.1 | 26.6 | 14.9 | 10.4 | 11.8 | 24.7 | 6.8 | 2.9 | 18.8 | 10.1 | 8.7 | 10.0 | 2.7 | 0.4 | 15.5 | 0.7 | 0.4 | 5.7 | 97 |
|  | **I – Diseases of the circulatory system** | 10.4 | 0.9 | 15.2 | 5.3 | 0.6 | 3.8 | 1.6 | 25.2 | 84.5 | 3.0 | 11.1 | 6.3 | 5.8 | 24.7 | 14.7 | 9.7 | 11.6 | 22.6 | 7.8 | 2.9 | 15.9 | 8.6 | 8.2 | 8.0 | 2.2 | 0.6 | 15.8 | 1.1 | 0.5 | 7.9 | 188 |
| 78 | Aortic and mitral valve disease ^c^ | 14.8 | 0.6 | 17.6 | 3.0 | 0.4 | 6.8 | 1.9 | 100.0 | 82.0 | 12.7 | 33.3 | 17.4 | 8.4 | 25.4 | 17.6 | 15.7 | 13.7 | 31.2 | 12.0 | 3.9 | 22.5 | 11.7 | 10.7 | 14.6 | 3.9 | 0.4 | 18.0 | 0.9 | 0.3 | 6.4 | 17 |
| 79 | Hypertensive diseases ^c^ | 10.8 | 1.0 | 17.2 | 5.6 | 0.6 | 4.1 | 1.7 | 22.6 | 100.0 | 3.4 | 11.0 | 6.1 | 5.1 | 25.3 | 15.2 | 10.1 | 11.9 | 24.0 | 8.4 | 3.0 | 17.0 | 9.2 | 8.4 | 8.4 | 2.3 | 0.5 | 16.0 | 1.0 | 0.4 | 8.3 | 179 |
| 80 | Heart failure ^c^ | 14.1 | 0.8 | 26.8 | 2.1 | 0.3 | 6.4 | 1.7 | 100.0 | 96.0 | 100.0 | 46.3 | 19.6 | 8.0 | 24.4 | 22.8 | 22.4 | 18.0 | 32.8 | 17.7 | 3.7 | 19.9 | 10.4 | 9.6 | 11.0 | 3.5 | 0.7 | 18.5 | 1.2 | 0.3 | 10.6 | 3 |
| 80A | Ischaemic heart diseases | 12.9 | 0.9 | 22.4 | 3.5 | 0.6 | 5.4 | 2.1 | 100.0 | 83.8 | 12.5 | 100.0 | 56.4 | 7.1 | 25.7 | 18.9 | 16.5 | 15.1 | 29.0 | 11.6 | 3.6 | 20.3 | 10.6 | 11.6 | 9.9 | 2.9 | 0.6 | 18.7 | 1.3 | 0.4 | 10.0 | 22 |
| 81 | Angina pectoris | 11.8 | 1.0 | 21.8 | 4.2 | 0.8 | 5.2 | 2.3 | 100.0 | 82.0 | 9.4 | 100.0 | 100.0 | 5.8 | 27.0 | 19.2 | 15.9 | 15.3 | 28.9 | 11.0 | 3.7 | 20.5 | 10.9 | 12.4 | 9.1 | 1.7 | 0.6 | 18.4 | 1.5 | 0.4 | 10.6 | 24 |
| 82 | Acute myocardial infarction and subsequent myocardial infarction | 11.7 | 0.9 | 20.4 | 2.5 | 0.4 | 4.8 | 1.8 | 100.0 | 85.8 | 15.4 | 100.0 | 33.9 | 6.4 | 21.8 | 16.3 | 14.9 | 13.0 | 25.2 | 10.4 | 3.3 | 17.3 | 8.7 | 9.5 | 8.6 | 2.5 | 0.5 | 17.2 | 1.2 | 0.3 | 8.4 | 12 |
| 83 | AMI complex/other | 13.3 | 0.8 | 22.1 | 4.2 | 0.8 | 4.9 | 1.7 | 100.0 | 82.5 | 17.5 | 100.0 | 39.1 | 8.2 | 25.9 | 19.8 | 16.8 | 15.7 | 28.7 | 12.2 | 4.3 | 19.3 | 9.8 | 11.7 | 8.7 | 2.6 | 0.9 | 21.1 | 2.2 | 0.6 | 11.9 | 2 |
| 84 | Chronic ischaemic heart disease | 14.3 | 1.0 | 25.9 | 2.7 | 0.5 | 6.1 | 2.2 | 100.0 | 90.1 | 16.7 | 100.0 | 42.2 | 8.7 | 25.4 | 19.5 | 18.0 | 15.4 | 30.9 | 13.3 | 3.8 | 21.3 | 11.0 | 11.9 | 10.6 | 3.7 | 0.5 | 19.5 | 1.3 | 0.3 | 11.0 | 6 |
| 85 | Pulmonary heart disease and diseases of pulmonary circulation | 18.8 | 0.4 | 14.9 | 4.3 | 0.6 | 5.4 | 1.5 | 100.0 | 62.8 | 8.9 | 18.0 | 8.9 | 6.2 | 25.4 | 25.4 | 24.3 | 21.2 | 29.3 | 11.9 | 4.7 | 20.6 | 11.2 | 11.4 | 14.1 | 3.4 | 1.0 | 22.1 | 1.8 | 0.8 | 11.8 | 30 |
| 86 | Acute pericarditis | 8.1 | 0.7 | 8.5 | 3.4 | 0.7 | 2.2 | 1.3 | 100.0 | 36.9 | 3.3 | 15.6 | 8.6 | 2.9 | 21.2 | 13.1 | 9.5 | 11.0 | 17.6 | 8.4 | 4.1 | 10.4 | 5.4 | 8.4 | 4.6 | 0.7 | 1.0 | 13.7 | 1.7 | 1.5 | 4.8 | 175 |
| 87 | Other forms of heart disease | 14.4 | 0.8 | 16.4 | 3.2 | 0.6 | 4.6 | 1.3 | 100.0 | 68.0 | 14.8 | 25.8 | 12.1 | 7.7 | 24.1 | 17.8 | 15.3 | 13.9 | 27.4 | 14.0 | 5.0 | 16.5 | 8.8 | 12.2 | 9.6 | 2.7 | 1.1 | 19.2 | 1.4 | 1.1 | 7.7 | 15 |
| 88 | Atrioventricular and left bundle branch block | 14.7 | 0.6 | 21.1 | 2.2 | 0.4 | 6.4 | 1.7 | 100.0 | 78.0 | 16.0 | 28.8 | 13.3 | 8.4 | 24.6 | 17.2 | 15.3 | 13.3 | 30.5 | 12.8 | 3.2 | 21.0 | 10.8 | 9.4 | 11.6 | 5.5 | 0.5 | 18.1 | 1.0 | 0.3 | 6.6 | 21 |
| 89 | Other conduction disorders | 11.6 | 0.7 | 15.4 | 3.1 | 0.7 | 4.4 | 1.8 | 100.0 | 67.4 | 12.2 | 30.6 | 12.4 | 7.4 | 23.9 | 16.4 | 12.8 | 13.2 | 23.4 | 9.2 | 2.8 | 15.9 | 8.3 | 9.0 | 8.3 | 3.9 | 0.9 | 19.0 | 1.7 | 0.8 | 7.5 | 31 |
| 90 | Paroxysmal tachycardia | 11.0 | 0.5 | 12.6 | 4.3 | 0.6 | 4.1 | 1.8 | 100.0 | 69.2 | 10.0 | 20.1 | 9.9 | 5.6 | 26.1 | 16.7 | 11.9 | 13.4 | 23.7 | 9.0 | 3.2 | 16.0 | 8.7 | 9.0 | 8.8 | 2.1 | 0.8 | 16.7 | 2.2 | 0.8 | 7.7 | 60 |
| 91 | Atrial fibrillation and flutter | 15.3 | 0.4 | 19.5 | 2.5 | 0.3 | 6.3 | 1.8 | 100.0 | 83.6 | 12.9 | 22.0 | 10.4 | 10.3 | 25.0 | 18.0 | 15.8 | 14.0 | 31.4 | 12.7 | 3.3 | 22.1 | 12.0 | 9.0 | 11.7 | 4.5 | 0.4 | 16.6 | 0.9 | 0.2 | 7.5 | 37 |
| 92 | Other cardiac arrhythmias | 11.9 | 0.5 | 14.0 | 4.0 | 0.7 | 4.8 | 2.0 | 100.0 | 70.3 | 9.9 | 22.3 | 11.0 | 6.5 | 27.0 | 16.0 | 5.9 | 12.8 | 26.2 | 9.6 | 3.2 | 18.5 | 9.9 | 9.8 | 10.2 | 3.3 | 0.5 | 17.2 | 1.6 | 0.6 | 7.4 | 43 |
| 93 | Complications and ill-defined descriptions of heart disease and other heart disorders in diseases classified elsewhere | 15.7 | 0.9 | 18.5 | 4.2 | 0.6 | 4.7 | 1.7 | 100.0 | 74.4 | 16.6 | 31.4 | 17.0 | 9.6 | 26.8 | 20.0 | 17.3 | 16.4 | 29.2 | 12.9 | 4.0 | 19.8 | 10.7 | 12.1 | 11.1 | 2.7 | 0.9 | 20.3 | 2.0 | 1.0 | 12.3 | 11 |
| 94 | Stroke | 12.5 | 0.9 | 17.9 | 4.0 | 0.9 | 5.1 | 1.8 | 31.3 | 73.8 | 4.1 | 13.6 | 6.2 | 100.0 | 23.3 | 15.3 | 12.3 | 11.5 | 24.6 | 9.2 | 3.1 | 17.2 | 8.7 | 9.5 | 10.6 | 5.6 | 0.7 | 28.5 | 1.3 | 0.4 | 6.1 | 32 |
| 95 | Cerebrovascular diseases | 12.4 | 0.6 | 16.4 | 5.7 | 1.8 | 4.9 | 2.5 | 29.7 | 68.8 | 4.0 | 16.1 | 8.3 | 34.0 | 25.4 | 16.6 | 12.6 | 12.2 | 24.4 | 9.6 | 3.4 | 16.9 | 8.3 | 11.5 | 10.5 | 5.4 | 0.5 | 24.6 | 1.5 | 0.5 | 7.3 | 25 |
| 96 | Sequelae of cerebrovascular disease | 14.0 | 0.9 | 20.3 | 4.0 | 1.2 | 5.6 | 2.0 | 37.3 | 77.3 | 5.4 | 17.7 | 7.7 | 53.9 | 25.4 | 17.1 | 14.8 | 12.9 | 27.5 | 10.7 | 3.4 | 19.2 | 9.6 | 11.0 | 14.5 | 10.8 | 0.8 | 36.3 | 1.7 | 0.5 | 6.8 | 7 |
| 97 | Atherosclerosis | 14.9 | 2.2 | 27.4 | 2.8 | 0.4 | 6.9 | 1.9 | 41.1 | 79.6 | 8.2 | 26.4 | 12.9 | 11.6 | 25.0 | 20.4 | 19.6 | 15.7 | 32.8 | 14.0 | 4.2 | 22.3 | 10.2 | 16.7 | 14.7 | 4.4 | 0.6 | 22.4 | 1.0 | 0.3 | 8.3 | 8 |
| 98 | Aortic aneurysm and aortic dissection | 18.5 | 0.2 | 16.2 | 2.5 | 0.4 | 6.4 | 2.4 | 45.5 | 80.6 | 7.5 | 25.7 | 12.2 | 8.8 | 23.9 | 19.3 | 20.5 | 14.6 | 27.7 | 11.6 | 3.5 | 18.6 | 9.0 | 12.6 | 12.4 | 3.4 | 0.4 | 17.6 | 0.8 | 0.2 | 5.2 | 20 |
| 99 | Diseases of arteries, arterioles and capillaries | 13.9 | 0.9 | 13.0 | 4.9 | 0.9 | 4.1 | 1.9 | 34.0 | 56.4 | 5.7 | 21.0 | 12.1 | 7.7 | 26.6 | 16.3 | 12.6 | 12.6 | 24.6 | 10.4 | 4.4 | 16.1 | 8.0 | 11.4 | 10.0 | 1.8 | 0.7 | 17.5 | 1.4 | 0.9 | 7.6 | 47 |
| 100 | Other peripheral vascular diseases | 13.5 | 1.8 | 23.2 | 2.8 | 0.4 | 6.1 | 1.9 | 35.2 | 74.8 | 6.6 | 23.5 | 13.0 | 7.9 | 23.3 | 19.0 | 18.8 | 14.7 | 28.6 | 11.8 | 3.8 | 19.7 | 8.7 | 16.1 | 12.9 | 1.9 | 0.4 | 18.2 | 0.8 | 0.4 | 6.3 | 23 |
| 101 | Phlebitis, thrombosis of the portal vein and others | 13.7 | 0.6 | 12.0 | 4.7 | 0.7 | 4.0 | 1.5 | 24.9 | 50.3 | 3.0 | 9.2 | 4.9 | 4.5 | 24.9 | 6.5 | 12.0 | 14.1 | 30.5 | 10.3 | 4.3 | 22.7 | 13.7 | 10.8 | 9.5 | 2.8 | 1.1 | 17.8 | 1.6 | 0.9 | 11.8 | 89 |
| 102 | Varicose veins of lower extremities | 7.0 | 0.4 | 7.2 | 5.4 | 0.4 | 2.2 | 1.1 | 11.5 | 36.4 | 1.6 | 4.5 | 2.8 | 1.8 | 24.0 | 12.3 | 6.6 | 9.4 | 19.8 | 5.5 | 2.6 | 14.4 | 8.2 | 7.4 | 6.8 | 0.9 | 0.4 | 11.8 | 0.8 | 0.5 | 11.6 | 209 |
| 103 | Haemorrhoids ^c^ | 7.3 | 0.4 | 6.5 | 5.8 | 0.7 | 1.8 | 1.4 | 10.0 | 39.0 | 1.1 | 4.7 | 2.8 | 2.1 | 28.6 | 13.3 | 6.5 | 11.1 | 15.4 | 5.0 | 2.4 | 10.4 | 5.4 | 7.7 | 5.1 | 1.1 | 0.7 | 16.1 | 1.6 | 1.1 | 9.2 | 207 |
| 104 | Oesophageal varices (chronic), varicose veins of other sites, other disorders of veins, non-specific lymphadenitis, other non-infective disorders of lymphatic vessels and lymph nodes and other and unspecified disorders of the circulatory system | 19.1 | 0.7 | 12.5 | 4.1 | 0.6 | 3.1 | 1.5 | 21.9 | 47.7 | 4.0 | 10.6 | 5.7 | 4.3 | 24.4 | 15.1 | 11.3 | 12.8 | 21.9 | 8.9 | 3.5 | 14.8 | 8.1 | 9.2 | 8.6 | 2.2 | 0.9 | 17.6 | 1.8 | 1.2 | 8.7 | 93 |
|  | **J – Diseases of the respiratory system** | 7.0 | 0.5 | 7.8 | 5.1 | 0.6 | 2.3 | 1.3 | 10.8 | 33.3 | 1.5 | 5.0 | 2.9 | 2.3 | 69.5 | 34.5 | 17.9 | 29.8 | 16.0 | 5.4 | 2.5 | 10.9 | 5.7 | 7.1 | 6.1 | 1.2 | 0.8 | 15.0 | 1.3 | 1.1 | 7.2 | 210 |
| 105 | Respiratory allergy ^c^ | 6.5 | 0.5 | 7.1 | 5.6 | 0.6 | 2.2 | 1.4 | 9.3 | 31.9 | 1.1 | 4.3 | 2.5 | 2.0 | 100.0 | 21.4 | 8.9 | 20.0 | 15.7 | 5.2 | 2.5 | 10.7 | 5.7 | 7.0 | 5.5 | 1.1 | 0.7 | 14.6 | 1.2 | 1.0 | 7.0 | 214 |
| 105A | Chronic lower respiratory diseases ^c^ | 8.1 | 0.5 | 9.6 | 5.5 | 0.6 | 2.8 | 1.4 | 13.3 | 38.5 | 2.0 | 6.3 | 3.6 | 2.7 | 43.1 | 100.0 | 29.0 | 57.7 | 18.4 | 6.4 | 2.9 | 12.6 | 6.6 | 8.2 | 8.2 | 1.3 | 0.9 | 17.1 | 1.5 | 1.1 | 9.0 | 145 |
| 106 | Bronchitis, not specified as acute or chronic, simple and mucopurulent chronic bronchitis and unspecified chronic bronchitis | 17.4 | 0.5 | 19.0 | 4.9 | 0.6 | 6.1 | 2.2 | 36.2 | 65.5 | 7.6 | 20.4 | 10.9 | 7.5 | 36.7 | 99.9 | 60.3 | 61.4 | 32.3 | 12.4 | 4.9 | 23.2 | 10.6 | 14.4 | 21.7 | 3.9 | 1.6 | 26.9 | 3.0 | 0.8 | 13.2 | 1 |
| 107 | Emphysema | 16.2 | 0.5 | 9.8 | 4.3 | 0.6 | 6.0 | 1.8 | 31.4 | 53.0 | 5.6 | 15.7 | 8.3 | 4.9 | 29.0 | 99.8 | 65.3 | 51.4 | 22.9 | 9.9 | 5.3 | 14.9 | 6.4 | 11.6 | 24.3 | 2.3 | 0.8 | 22.5 | 1.8 | 0.4 | 5.1 | 9 |
| 108 | Chronic obstructive lung disease (COPD) ^c^ | 11.3 | 0.5 | 12.5 | 4.5 | 0.5 | 4.4 | 1.6 | 21.2 | 49.6 | 3.9 | 10.6 | 5.8 | 4.1 | 34.8 | 56.1 | 100.0 | 56.2 | 21.5 | 7.8 | 3.3 | 15.0 | 7.4 | 9.4 | 12.8 | 2.1 | 1.1 | 19.8 | 1.5 | 0.8 | 7.7 | 64 |
| 109 | Asthma, status asthmaticus ^c^ | 7.2 | 0.5 | 8.6 | 5.2 | 0.6 | 2.6 | 1.3 | 12.2 | 34.9 | 1.9 | 5.8 | 3.3 | 2.3 | 46.7 | 66.8 | 33.6 | 100.0 | 16.6 | 5.7 | 2.6 | 11.4 | 5.9 | 7.5 | 7.6 | 1.1 | 1.0 | 16.9 | 1.5 | 1.4 | 8.8 | 148 |
| 110 | Bronchiectasis | 16.8 | 0.6 | 8.4 | 6.0 | 0.6 | 4.3 | 2.0 | 19.2 | 42.9 | 2.6 | 9.2 | 6.1 | 2.9 | 38.9 | 99.4 | 45.0 | 47.4 | 24.4 | 9.8 | 5.7 | 15.9 | 7.4 | 10.4 | 17.9 | 1.0 | 0.6 | 17.1 | 1.0 | 0.7 | 5.7 | 33 |
| 111 | Other diseases of the respiratory system | 20.8 | 0.9 | 15.7 | 4.2 | 0.7 | 4.9 | 1.9 | 34.2 | 57.2 | 8.3 | 16.5 | 7.9 | 7.1 | 28.9 | 35.8 | 33.8 | 29.5 | 27.8 | 13.7 | 6.8 | 17.1 | 8.4 | 12.2 | 16.2 | 3.2 | 1.4 | 23.0 | 1.8 | 0.9 | 9.0 | 19 |
|  | **K – Diseases of the digestive system** | 11.3 | 0.8 | 11.7 | 5.8 | 0.8 | 3.2 | 1.6 | 17.8 | 45.5 | 2.5 | 9.3 | 5.1 | 4.2 | 28.4 | 17.1 | 11.1 | 14.3 | 24.0 | 9.6 | 4.7 | 16.1 | 8.5 | 11.0 | 9.4 | 2.4 | 1.2 | 21.5 | 1.9 | 1.1 | 8.9 | 121 |
| 112 | Ulcers ^c^ | 12.1 | 0.7 | 13.8 | 6.2 | 0.9 | 3.8 | 1.8 | 21.7 | 52.7 | 3.3 | 11.9 | 6.5 | 5.2 | 30.2 | 19.2 | 13.2 | 16.1 | 27.2 | 10.1 | 4.4 | 19.4 | 10.6 | 12.7 | 10.8 | 3.0 | 1.3 | 24.2 | 2.0 | 1.1 | 9.5 | 76 |
| 113 | Inguinal hernia | 10.0 | 0.3 | 6.9 | 2.3 | 0.3 | 2.6 | 1.7 | 16.3 | 37.9 | 2.2 | 7.8 | 4.1 | 3.1 | 19.9 | 11.3 | 7.9 | 9.5 | 17.8 | 5.6 | 2.0 | 13.2 | 6.3 | 6.7 | 4.2 | 1.4 | 0.3 | 9.2 | 0.6 | 0.5 | 3.4 | 208 |
| 114 | Ventral hernia | 22.9 | 0.7 | 14.6 | 5.4 | 0.6 | 3.6 | 1.9 | 20.6 | 53.5 | 2.4 | 11.0 | 6.5 | 3.4 | 27.3 | 19.5 | 13.5 | 16.5 | 26.1 | 9.1 | 3.8 | 19.1 | 10.7 | 10.6 | 8.0 | 1.1 | 1.1 | 21.3 | 1.8 | 1.0 | 21.9 | 63 |
| 115 | Crohn’s diease | 7.1 | 0.6 | 6.0 | 6.4 | 0.7 | 1.7 | 1.0 | 9.6 | 27.8 | 1.1 | 4.3 | 2.4 | 1.9 | 26.0 | 15.1 | 7.6 | 12.8 | 22.1 | 14.7 | 9.7 | 8.8 | 4.2 | 8.1 | 7.1 | 0.8 | 0.9 | 16.9 | 1.6 | 1.2 | 7.6 | 187 |
| 116 | Ulcerative colitis | 7.6 | 0.7 | 7.8 | 5.6 | 0.6 | 2.0 | 1.2 | 11.4 | 32.7 | 1.2 | 5.4 | 3.2 | 2.3 | 26.3 | 14.3 | 7.7 | 12.2 | 26.2 | 16.9 | 12.6 | 11.3 | 5.7 | 8.1 | 7.3 | 1.2 | 0.6 | 15.5 | 1.3 | 1.0 | 6.8 | 185 |
| 117 | Other non-infective gastroenteritis and colitis | 15.0 | 1.2 | 13.4 | 6.3 | 0.7 | 4.2 | 1.6 | 21.3 | 50.9 | 3.5 | 11.0 | 5.6 | 5.4 | 31.1 | 19.0 | 13.3 | 15.8 | 27.3 | 11.5 | 5.4 | 18.1 | 9.1 | 13.0 | 13.8 | 3.1 | 1.1 | 26.9 | 2.3 | 1.2 | 9.0 | 48 |
| 118 | Irritable bowel syndrome (IBS) | 6.7 | 0.5 | 7.2 | 7.9 | 1.2 | 2.1 | 1.7 | 11.1 | 34.1 | 1.1 | 5.7 | 3.7 | 2.2 | 32.5 | 16.7 | 8.6 | 14.3 | 19.0 | 6.6 | 3.6 | 12.6 | 6.3 | 11.3 | 6.9 | 1.0 | 0.8 | 22.0 | 2.7 | 1.4 | 9.8 | 170 |
| 119 | Other functional intestinal disorders | 15.1 | 1.0 | 12.5 | 6.4 | 1.1 | 4.0 | 1.8 | 21.5 | 49.4 | 3.1 | 11.4 | 6.2 | 5.9 | 30.5 | 18.7 | 13.4 | 15.7 | 25.0 | 8.9 | 4.0 | 17.8 | 8.7 | 14.2 | 13.3 | 4.3 | 1.7 | 28.5 | 3.1 | 1.5 | 9.9 | 51 |
| 120 | Diseases of liver, biliary tract and pancreas | 12.7 | 2.7 | 20.1 | 5.1 | 0.7 | 3.3 | 1.6 | 17.7 | 52.3 | 2.8 | 8.3 | 4.6 | 4.4 | 27.3 | 17.5 | 12.6 | 14.1 | 23.5 | 10.7 | 4.5 | 14.6 | 7.3 | 10.5 | 11.4 | 1.8 | 1.8 | 23.9 | 2.4 | 1.1 | 11.0 | 62 |
|  | **L – Diseases of the skin and subcutaneous tissue** | 8.1 | 0.6 | 10.8 | 4.2 | 0.5 | 2.7 | 1.4 | 12.2 | 38.5 | 1.7 | 5.8 | 3.3 | 3.0 | 25.9 | 14.2 | 8.5 | 11.3 | 25.4 | 15.4 | 11.2 | 13.0 | 6.7 | 7.7 | 6.5 | 1.7 | 0.9 | 14.6 | 1.1 | 0.8 | 7.5 | 194 |
| 121 | Psoriasis ^c^ | 8.1 | 0.6 | 10.8 | 4.2 | 0.5 | 2.7 | 1.4 | 12.2 | 38.5 | 1.7 | 5.8 | 3.3 | 3.0 | 25.9 | 14.2 | 8.5 | 11.3 | 25.4 | 15.4 | 11.2 | 13.0 | 6.7 | 7.7 | 6.5 | 1.7 | 0.9 | 14.6 | 1.1 | 0.8 | 7.5 | 195 |
|  | **M – Diseases of the musculoskeletal system and connective tissue** | 8.9 | 0.6 | 9.6 | 4.9 | 0.6 | 3.1 | 1.6 | 13.7 | 41.1 | 1.7 | 6.3 | 3.6 | 3.0 | 25.3 | 14.6 | 8.8 | 11.8 | 49.0 | 15.8 | 7.5 | 32.7 | 17.3 | 20.6 | 15.4 | 1.7 | 0.4 | 15.0 | 1.0 | 0.8 | 7.2 | 193 |
| 122 | Infectious arthropathies | 6.8 | 0.8 | 7.0 | 4.8 | 0.6 | 1.8 | 1.3 | 11.7 | 30.8 | 1.5 | 5.3 | 3.0 | 2.4 | 24.8 | 13.3 | 6.5 | 11.0 | 100.0 | 39.5 | 25.0 | 17.5 | 9.5 | 9.7 | 6.4 | 0.8 | 0.3 | 13.1 | 1.1 | 1.0 | 7.1 | 176 |
| 122A | Inflammatory polyarthropathies and ankylosing spondylitis ^c^ | 10.3 | 0.8 | 16.3 | 4.2 | 0.5 | 3.7 | 1.7 | 20.2 | 53.9 | 4.0 | 9.7 | 5.2 | 4.0 | 26.3 | 16.1 | 10.2 | 12.5 | 97.8 | 100.0 | 46.6 | 23.1 | 12.1 | 12.1 | 11.1 | 1.7 | 0.4 | 14.5 | 0.8 | 0.4 | 8.7 | 103 |
| 123 | Rheumatoid arthritis ^c^ | 9.0 | 0.7 | 9.3 | 5.8 | 0.6 | 3.3 | 1.5 | 14.1 | 41.7 | 1.8 | 6.6 | 3.7 | 2.9 | 27.4 | 15.9 | 9.2 | 12.3 | 100.0 | 100.0 | 100.0 | 23.1 | 11.2 | 13.4 | 16.0 | 1.4 | 0.4 | 14.5 | 1.0 | 0.5 | 7.5 | 115 |
| 124 | Inflammatory polyarthropathies – except rheumatoid arthritis ^c^ | 10.8 | 0.9 | 18.8 | 3.7 | 0.4 | 3.9 | 1.7 | 22.8 | 58.8 | 5.1 | 11.1 | 5.8 | 4.4 | 25.9 | 16.2 | 10.8 | 12.7 | 100.0 | 100.0 | 27.9 | 23.4 | 12.4 | 12.3 | 9.8 | 1.8 | 0.4 | 14.5 | 0.8 | 0.4 | 9.3 | 78 |
| 125 | Polyarthrosis [arthrosis] | 13.1 | 0.7 | 13.9 | 7.0 | 0.7 | 5.7 | 2.4 | 20.8 | 60.3 | 2.7 | 10.3 | 6.0 | 4.2 | 35.1 | 19.6 | 12.4 | 15.5 | 100.0 | 28.8 | 18.5 | 100.0 | 26.7 | 23.2 | 19.6 | 3.1 | 0.2 | 21.0 | 1.2 | 0.4 | 9.6 | 28 |
| 126 | Coxarthrosis [arthrosis of hip] | 12.5 | 0.4 | 13.5 | 3.6 | 0.4 | 5.6 | 1.8 | 21.8 | 60.1 | 3.0 | 9.8 | 5.2 | 4.6 | 25.3 | 15.6 | 11.0 | 12.1 | 100.0 | 11.1 | 4.9 | 100.0 | 20.9 | 17.0 | 12.5 | 3.3 | 0.3 | 15.7 | 0.8 | 0.3 | 7.5 | 90 |
| 127 | Gonarthrosis [arthrosis of knee] | 10.4 | 0.4 | 13.8 | 4.6 | 0.5 | 4.2 | 2.0 | 18.0 | 54.5 | 2.2 | 8.3 | 4.8 | 3.5 | 27.0 | 15.4 | 9.0 | 11.9 | 100.0 | 11.2 | 4.8 | 100.0 | 100.0 | 12.3 | 9.2 | 2.1 | 0.3 | 14.6 | 0.8 | 0.4 | 10.9 | 125 |
| 128 | Arthrosis of first carpometacarpal joint and other arthrosis | 10.4 | 0.6 | 11.5 | 6.0 | 0.6 | 3.9 | 2.2 | 16.7 | 49.5 | 1.9 | 8.5 | 5.1 | 3.5 | 29.4 | 17.5 | 10.3 | 13.7 | 100.0 | 13.6 | 7.1 | 100.0 | 18.6 | 15.6 | 11.1 | 1.7 | 0.3 | 17.0 | 1.1 | 0.6 | 9.3 | 96 |
| 129 | Acquired deformities of fingers and toes | 8.1 | 0.5 | 6.9 | 7.0 | 0.6 | 3.1 | 1.6 | 10.8 | 38.8 | 1.0 | 4.9 | 3.1 | 2.4 | 28.5 | 14.7 | 7.4 | 11.6 | 100.0 | 10.3 | 6.8 | 23.9 | 11.5 | 10.0 | 10.0 | 1.1 | 0.5 | 16.2 | 1.1 | 0.7 | 7.1 | 182 |
| 130 | Other acquired deformities of limbs | 7.9 | 0.7 | 9.5 | 6.0 | 0.6 | 3.0 | 1.6 | 12.0 | 38.9 | 1.4 | 5.4 | 3.2 | 2.8 | 28.2 | 14.7 | 8.0 | 12.8 | 39.4 | 9.9 | 5.5 | 24.7 | 12.1 | 12.2 | 9.4 | 1.3 | 0.7 | 16.9 | 1.2 | 1.0 | 9.4 | 143 |
| 131 | Disorders of patella (knee cap) | 2.1 | 0.5 | 2.4 | 5.4 | 0.8 | 0.4 | 0.7 | 3.7 | 13.0 | 0.2 | 1.4 | 1.0 | 0.5 | 23.3 | 10.8 | 3.9 | 10.6 | 15.7 | 3.3 | 1.7 | 12.1 | 9.7 | 6.1 | 1.1 | 0.1 | 0.7 | 13.8 | 1.4 | 2.1 | 8.9 | 230 |
| 132 | Internal derangement of knee | 2.6 | 0.3 | 2.8 | 4.2 | 0.6 | 0.6 | 0.9 | 4.5 | 14.6 | 0.3 | 1.7 | 1.2 | 0.8 | 20.5 | 9.3 | 3.5 | 9.0 | 21.8 | 3.6 | 1.4 | 18.7 | 16.3 | 5.6 | 1.3 | 0.1 | 0.4 | 9.3 | 0.8 | 1.3 | 5.7 | 225 |
| 133 | Derangement of meniscus due to old tear or injury | 4.4 | 0.4 | 4.5 | 4.7 | 0.6 | 0.9 | 1.2 | 6.6 | 24.2 | 0.4 | 3.0 | 1.9 | 1.0 | 23.4 | 11.4 | 4.4 | 9.8 | 33.3 | 4.6 | 2.1 | 29.9 | 26.7 | 7.2 | 2.7 | 0.1 | 0.3 | 10.8 | 0.8 | 1.0 | 7.4 | 215 |
| 134 | Internal derangement of knee, unspecified | 3.6 | 0.5 | 4.4 | 4.7 | 0.7 | 0.9 | 0.9 | 6.3 | 21.6 | 0.4 | 2.8 | 1.9 | 1.0 | 22.9 | 11.1 | 4.7 | 10.1 | 23.6 | 4.9 | 2.1 | 19.5 | 15.7 | 7.4 | 2.5 | 0.2 | 0.4 | 11.7 | 0.9 | 1.4 | 7.4 | 221 |
| 135 | Other specific joint derangements | 3.1 | 0.6 | 3.0 | 4.3 | 0.7 | 0.8 | 1.0 | 5.6 | 16.1 | 0.4 | 2.2 | 1.3 | 1.2 | 21.6 | 10.6 | 4.6 | 9.9 | 19.1 | 4.6 | 2.2 | 15.3 | 6.1 | 7.6 | 2.2 | 0.3 | 0.6 | 11.0 | 1.2 | 1.9 | 5.6 | 223 |
| 136 | Other joint disorders, not elsewhere classified | 7.1 | 0.7 | 7.2 | 7.1 | 1.0 | 2.0 | 1.4 | 10.0 | 31.0 | 1.0 | 4.8 | 3.1 | 1.6 | 28.0 | 15.0 | 7.2 | 13.2 | 35.5 | 16.8 | 9.6 | 20.7 | 10.4 | 15.0 | 6.7 | 0.5 | 0.4 | 17.0 | 1.5 | 1.4 | 9.9 | 158 |
| 137 | Systemic connective tissue disorders | 11.2 | 0.6 | 12.7 | 6.8 | 1.0 | 5.1 | 1.8 | 20.3 | 51.6 | 2.7 | 9.1 | 5.1 | 4.5 | 33.3 | 19.1 | 11.7 | 15.2 | 37.1 | 19.3 | 12.9 | 22.3 | 10.6 | 18.6 | 26.3 | 2.8 | 0.4 | 20.3 | 1.4 | 0.9 | 8.7 | 56 |
| 138 | Systemic lupus erythematosus | 9.2 | 0.6 | 7.2 | 8.4 | 1.3 | 2.4 | 1.5 | 19.3 | 48.3 | 2.3 | 7.6 | 4.6 | 4.7 | 32.9 | 18.0 | 11.3 | 13.7 | 63.4 | 56.0 | 52.3 | 15.0 | 6.9 | 100.0 | 23.5 | 1.0 | 0.4 | 20.1 | 1.6 | 0.5 | 9.7 | 34 |
| 139 | Dermatopolymyositis | 12.6 | 0.5 | 13.4 | 4.7 | 0.6 | 4.5 | 1.5 | 20.7 | 49.1 | 3.3 | 8.9 | 4.8 | 3.3 | 26.4 | 15.5 | 9.8 | 10.6 | 35.8 | 20.3 | 13.6 | 19.1 | 10.0 | 100.0 | 28.8 | 0.6 | 0.0 | 16.8 | 1.3 | 0.6 | 7.0 | 46 |
| 140 | Systemic sclerosis | 13.2 | 1.0 | 9.7 | 7.1 | 0.9 | 3.2 | 1.6 | 26.9 | 58.4 | 3.6 | 10.8 | 6.6 | 4.1 | 32.5 | 19.5 | 13.4 | 13.6 | 47.6 | 35.4 | 29.4 | 20.2 | 8.5 | 100.0 | 23.6 | 1.4 | 0.0 | 16.6 | 0.9 | 0.4 | 7.3 | 27 |
| 141 | Kyphosis, lordosis | 7.0 | 0.4 | 6.7 | 6.3 | 0.9 | 1.8 | 2.0 | 9.9 | 29.9 | 1.3 | 5.1 | 2.9 | 2.1 | 26.8 | 15.0 | 7.6 | 11.2 | 20.9 | 8.0 | 3.7 | 14.5 | 6.5 | 100.0 | 8.6 | 0.9 | 0.9 | 17.8 | 1.8 | 1.4 | 6.7 | 167 |
| 142 | Scoliosis | 5.1 | 0.5 | 3.9 | 5.6 | 0.7 | 1.9 | 1.0 | 8.4 | 24.0 | 0.9 | 3.5 | 2.0 | 1.8 | 23.3 | 12.1 | 6.9 | 11.0 | 16.9 | 4.7 | 2.5 | 12.7 | 5.5 | 100.0 | 8.4 | 1.0 | 0.8 | 15.4 | 1.3 | 1.6 | 5.1 | 199 |
| 143 | Spinal osteochondrosis | 5.9 | 0.6 | 7.1 | 6.0 | 1.2 | 1.6 | 1.8 | 10.1 | 30.3 | 1.2 | 5.3 | 3.3 | 2.3 | 27.0 | 15.2 | 7.9 | 12.0 | 22.2 | 7.7 | 3.4 | 15.5 | 7.0 | 100.0 | 5.4 | 0.6 | 0.9 | 18.0 | 1.7 | 1.8 | 8.2 | 177 |
| 144 | Other deforming dorsopathies | 8.8 | 0.4 | 10.1 | 6.9 | 0.8 | 3.2 | 2.1 | 14.6 | 44.7 | 1.6 | 7.7 | 4.8 | 3.1 | 29.1 | 17.4 | 10.2 | 13.6 | 32.2 | 9.6 | 4.9 | 25.4 | 12.8 | 100.0 | 10.2 | 1.6 | 0.4 | 19.4 | 1.2 | 0.8 | 9.4 | 82 |
| 145 | Other inflammatory spondylopathies | 8.5 | 0.9 | 9.2 | 6.4 | 0.8 | 2.3 | 1.4 | 14.9 | 37.1 | 1.9 | 6.5 | 3.8 | 2.7 | 27.5 | 14.9 | 8.4 | 12.2 | 41.0 | 27.3 | 15.2 | 17.7 | 8.4 | 100.0 | 7.9 | 1.1 | 0.6 | 18.2 | 1.2 | 1.3 | 8.8 | 99 |
| 146 | Spondylosis | 10.2 | 0.5 | 12.5 | 7.1 | 1.1 | 3.9 | 2.6 | 18.4 | 52.3 | 2.1 | 10.0 | 6.1 | 4.3 | 30.2 | 18.6 | 12.0 | 14.5 | 35.7 | 9.7 | 4.1 | 29.3 | 14.5 | 100.0 | 11.0 | 2.2 | 0.3 | 21.4 | 1.3 | 0.6 | 8.8 | 59 |
| 147 | Other spondylopathies and spondylopathies in diseases classified elsewhere | 13.0 | 0.5 | 15.6 | 5.8 | 0.7 | 5.8 | 2.5 | 23.4 | 63.3 | 3.1 | 12.7 | 7.2 | 5.1 | 31.5 | 19.9 | 14.0 | 15.1 | 41.7 | 11.8 | 5.0 | 34.4 | 16.7 | 100.0 | 15.3 | 2.8 | 0.3 | 22.2 | 1.1 | 0.5 | 8.8 | 29 |
| 148 | Cervical disc disorders | 5.7 | 0.6 | 6.7 | 8.2 | 2.0 | 1.1 | 1.9 | 9.3 | 32.2 | 0.6 | 5.2 | 3.5 | 2.0 | 27.1 | 15.8 | 6.9 | 12.2 | 21.3 | 6.6 | 3.3 | 15.2 | 7.1 | 100.0 | 4.5 | 0.3 | 0.3 | 19.0 | 1.4 | 0.9 | 7.8 | 150 |
| 149 | Other intervertebral disc disorders | 6.3 | 0.5 | 8.1 | 6.8 | 1.0 | 1.8 | 1.8 | 10.6 | 34.1 | 0.9 | 5.7 | 3.8 | 2.0 | 26.3 | 14.9 | 7.5 | 12.1 | 22.5 | 6.6 | 3.1 | 16.8 | 7.8 | 100.0 | 6.1 | 0.5 | 0.5 | 18.5 | 1.4 | 1.1 | 9.6 | 152 |
| 150 | Other dorsopathies, not elsewhere classified | 6.0 | 0.4 | 6.9 | 8.3 | 2.0 | 1.5 | 1.4 | 10.7 | 32.5 | 0.9 | 5.5 | 3.8 | 2.2 | 27.7 | 15.1 | 7.5 | 12.6 | 21.0 | 5.9 | 2.7 | 15.5 | 7.2 | 100.0 | 6.8 | 0.6 | 0.4 | 20.2 | 1.5 | 1.4 | 10.7 | 140 |
| 151 | Dorsalgia | 7.5 | 0.5 | 7.8 | 7.2 | 1.4 | 2.2 | 1.7 | 12.9 | 34.8 | 1.4 | 6.7 | 4.1 | 2.8 | 27.3 | 15.6 | 8.6 | 12.8 | 22.8 | 7.2 | 3.3 | 16.8 | 7.9 | 100.0 | 9.3 | 1.2 | 0.6 | 20.6 | 2.0 | 1.2 | 9.8 | 123 |
| 152 | Soft tissue disorders | 6.2 | 0.6 | 7.1 | 7.6 | 3.1 | 1.7 | 1.4 | 12.9 | 30.6 | 1.2 | 7.5 | 4.9 | 2.5 | 27.7 | 15.1 | 7.9 | 13.4 | 17.6 | 6.6 | 3.5 | 11.6 | 5.6 | 22.1 | 5.2 | 0.7 | 0.9 | 20.6 | 2.8 | 1.8 | 13.6 | 163 |
| 153 | Synovitis and tenosynovitis | 6.8 | 2.1 | 8.1 | 5.8 | 0.6 | 2.1 | 1.6 | 8.8 | 32.0 | 0.8 | 4.4 | 2.9 | 1.6 | 25.9 | 13.1 | 6.1 | 11.1 | 28.6 | 7.4 | 3.6 | 22.2 | 12.9 | 9.5 | 5.7 | 0.3 | 0.3 | 14.1 | 0.9 | 1.2 | 7.8 | 190 |
| 154 | Disorders of synovium and tendon | 4.5 | 0.6 | 4.4 | 5.2 | 0.6 | 1.1 | 1.1 | 6.3 | 22.4 | 0.5 | 2.8 | 1.9 | 1.1 | 23.7 | 11.0 | 5.0 | 10.0 | 23.1 | 5.7 | 3.1 | 17.7 | 11.8 | 7.3 | 3.4 | 0.2 | 0.3 | 12.0 | 1.0 | 1.5 | 7.2 | 216 |
| 155 | Soft tissue disorders related to use, overuse and pressure | 7.6 | 0.6 | 8.7 | 5.9 | 0.7 | 2.8 | 1.7 | 13.2 | 37.6 | 1.4 | 6.8 | 4.3 | 2.4 | 26.8 | 15.0 | 8.9 | 13.2 | 37.3 | 9.3 | 3.9 | 30.7 | 11.0 | 17.3 | 7.7 | 0.8 | 0.4 | 15.3 | 1.1 | 1.0 | 8.1 | 144 |
| 156 | Fibroblastic disorders | 10.7 | 1.9 | 11.4 | 3.6 | 0.4 | 3.7 | 1.9 | 15.8 | 45.2 | 1.9 | 7.3 | 4.3 | 3.6 | 23.6 | 13.6 | 9.2 | 10.8 | 24.1 | 7.8 | 2.9 | 17.2 | 8.9 | 8.3 | 6.2 | 1.5 | 0.3 | 12.4 | 0.7 | 0.4 | 5.5 | 184 |
| 157 | Shoulder lesions | 5.9 | 1.3 | 8.4 | 6.0 | 0.8 | 1.6 | 1.8 | 10.0 | 32.8 | 0.8 | 5.0 | 3.4 | 1.9 | 25.6 | 13.6 | 6.7 | 10.8 | 28.5 | 6.0 | 2.6 | 23.7 | 8.9 | 13.1 | 4.7 | 0.3 | 0.3 | 14.2 | 0.9 | 0.9 | 7.7 | 198 |
| 158 | Enthesopathies of lower limb, excluding foot | 3.9 | 0.4 | 4.6 | 5.1 | 0.6 | 1.2 | 1.1 | 6.3 | 22.4 | 0.5 | 2.9 | 1.8 | 1.2 | 25.1 | 11.7 | 4.4 | 10.1 | 20.3 | 5.0 | 2.3 | 15.4 | 7.8 | 9.7 | 2.9 | 0.2 | 0.3 | 10.5 | 1.0 | 0.9 | 6.9 | 220 |
| 159 | Other enthesopathies | 4.6 | 0.7 | 5.2 | 6.4 | 1.0 | 1.2 | 1.5 | 7.0 | 25.6 | 0.5 | 3.3 | 2.4 | 1.3 | 27.0 | 13.3 | 5.6 | 11.2 | 26.4 | 7.1 | 4.1 | 16.8 | 7.4 | 13.1 | 4.0 | 0.2 | 0.3 | 14.4 | 1.2 | 0.9 | 8.6 | 201 |
| 160 | Rheumatism, unspecified | 8.2 | 0.7 | 11.0 | 14.5 | 2.1 | 2.2 | 2.3 | 14.0 | 44.9 | 1.4 | 7.8 | 5.2 | 2.7 | 40.0 | 24.0 | 10.4 | 18.4 | 36.3 | 17.8 | 11.8 | 22.2 | 10.6 | 21.3 | 10.1 | 0.8 | 0.6 | 29.9 | 3.1 | 1.6 | 16.8 | 50 |
| 161 | Myalgia | 8.2 | 0.7 | 10.6 | 8.3 | 2.0 | 2.8 | 1.8 | 19.1 | 42.8 | 2.1 | 11.6 | 7.3 | 3.5 | 30.4 | 18.5 | 10.6 | 15.3 | 26.1 | 9.6 | 5.2 | 18.0 | 8.7 | 18.3 | 8.4 | 1.3 | 0.8 | 23.1 | 2.9 | 1.2 | 11.5 | 92 |
| 162 | Other soft tissue disorders, not elsewhere classified | 8.3 | 0.7 | 8.3 | 8.3 | 1.5 | 2.5 | 1.6 | 12.4 | 34.8 | 1.6 | 6.7 | 4.2 | 3.6 | 28.0 | 15.6 | 8.6 | 12.7 | 25.6 | 9.0 | 4.8 | 17.3 | 7.9 | 18.0 | 6.8 | 0.8 | 0.4 | 20.6 | 1.8 | 1.0 | 10.4 | 132 |
| 163 | Other soft tissue disorders, not elsewhere classified: pain in limb | 7.0 | 0.6 | 8.2 | 5.6 | 0.9 | 2.4 | 1.7 | 12.3 | 35.5 | 1.6 | 6.0 | 3.6 | 2.9 | 26.1 | 14.8 | 8.0 | 12.5 | 30.0 | 7.8 | 3.3 | 23.2 | 12.0 | 14.7 | 7.0 | 1.2 | 0.6 | 16.8 | 1.4 | 1.2 | 9.3 | 154 |
| 164 | Fibromyalgia | 6.5 | 0.3 | 8.9 | 17.0 | 3.1 | 1.5 | 1.9 | 11.0 | 39.8 | 0.5 | 6.4 | 4.3 | 1.8 | 41.5 | 24.4 | 9.4 | 19.2 | 31.9 | 16.4 | 11.2 | 17.7 | 7.5 | 20.6 | 6.9 | 0.3 | 0.3 | 39.6 | 3.9 | 2.0 | 20.8 | 54 |
| 165 | Osteoporosis ^c^ | 15.1 | 0.6 | 10.0 | 4.9 | 0.4 | 6.4 | 1.8 | 20.3 | 56.2 | 2.6 | 8.7 | 4.5 | 4.9 | 29.3 | 21.5 | 17.4 | 17.3 | 30.8 | 11.6 | 7.8 | 21.7 | 10.3 | 12.9 | 100.0 | 4.5 | 0.4 | 19.8 | 0.9 | 0.3 | 3.8 | 71 |
| 166 | Osteoporosis in diseases classified elsewhere | 19.9 | 2.5 | 11.2 | 4.0 | 0.0 | 5.0 | 1.9 | 23.9 | 52.8 | 4.4 | 11.2 | 6.0 | 4.4 | 28.5 | 27.1 | 23.1 | 21.5 | 37.8 | 24.9 | 17.9 | 20.7 | 7.9 | 15.0 | 70.3 | 3.5 | 0.6 | 20.3 | 1.3 | 0.0 | 4.0 | 10 |
| 167 | Adult osteomalacia and other disorders of bone density and structure | 14.3 | 0.7 | 9.2 | 6.5 | 0.5 | 3.9 | 1.7 | 14.8 | 45.6 | 1.4 | 6.7 | 4.1 | 3.0 | 29.4 | 19.5 | 13.7 | 16.2 | 30.4 | 12.1 | 8.4 | 20.3 | 10.0 | 12.4 | 34.0 | 1.6 | 0.4 | 17.4 | 0.9 | 0.5 | 5.0 | 102 |
| 168 | Disorders of continuity of bone | 9.4 | 0.9 | 7.7 | 4.3 | 0.5 | 2.0 | 1.1 | 10.9 | 32.5 | 1.1 | 4.9 | 2.8 | 2.7 | 23.7 | 14.5 | 10.2 | 12.7 | 28.7 | 6.8 | 3.4 | 22.9 | 10.2 | 11.6 | 12.2 | 1.1 | 1.2 | 19.9 | 1.4 | 1.8 | 8.1 | 155 |
| 169 | Other osteopathies | 14.6 | 1.2 | 12.4 | 5.6 | 0.8 | 4.7 | 1.8 | 16.4 | 46.7 | 2.4 | 7.3 | 3.9 | 3.5 | 27.2 | 17.1 | 11.0 | 13.8 | 37.3 | 12.9 | 5.7 | 27.3 | 11.3 | 13.7 | 20.7 | 2.1 | 0.7 | 19.0 | 1.2 | 0.7 | 7.5 | 79 |
| 170 | Other disorders of the musculoskeletal system and connective tissue | 7.1 | 0.6 | 7.9 | 5.8 | 0.9 | 2.2 | 1.7 | 11.7 | 33.6 | 1.4 | 6.2 | 3.8 | 2.8 | 27.9 | 15.1 | 8.2 | 12.3 | 25.1 | 6.7 | 3.1 | 19.5 | 9.0 | 31.1 | 7.0 | 1.3 | 0.9 | 18.3 | 1.5 | 1.4 | 7.3 | 146 |
|  | **N – Diseases of the genitourinary system** | 18.0 | 2.7 | 27.9 | 3.0 | 0.5 | 6.8 | 1.6 | 44.9 | 89.4 | 12.9 | 25.1 | 12.0 | 9.2 | 27.1 | 18.3 | 17.0 | 14.1 | 41.1 | 29.1 | 4.3 | 19.4 | 9.6 | 11.0 | 11.3 | 3.6 | 0.7 | 20.2 | 0.9 | 0.4 | 10.0 | 4 |
| 171 | Chronic renal failure (CRF) ^c^ | 18.0 | 2.7 | 27.9 | 3.0 | 0.5 | 6.8 | 1.6 | 44.9 | 89.4 | 12.9 | 25.1 | 12.0 | 9.2 | 27.1 | 18.3 | 17.0 | 14.1 | 41.1 | 29.1 | 4.3 | 19.4 | 9.6 | 11.0 | 11.3 | 3.6 | 0.7 | 20.2 | 0.9 | 0.4 | 10.0 | 5 |
|  | **Q – Congenital malformations, deformations and chromosomal abnormalities** | 6.1 | 0.6 | 4.9 | 5.0 | 0.8 | 1.4 | 1.1 | 9.3 | 24.0 | 1.0 | 3.6 | 2.1 | 2.2 | 23.4 | 11.6 | 5.4 | 10.1 | 14.9 | 4.7 | 2.2 | 9.6 | 4.2 | 8.7 | 3.7 | 0.7 | 0.8 | 13.6 | 1.4 | 1.4 | 8.4 | 218 |
| 172 | Congenital malformations: of the nervous, circulatory and respiratory systems, cleft palate and cleft lip, urinary tract, bones and muscles, other and chromosomal abnormalities not elsewhere classified | 5.8 | 0.6 | 4.8 | 5.3 | 0.8 | 1.3 | 1.1 | 10.1 | 24.4 | 1.1 | 3.5 | 2.1 | 2.5 | 23.7 | 11.7 | 5.4 | 10.3 | 16.3 | 5.0 | 2.4 | 10.5 | 4.3 | 9.9 | 3.8 | 0.8 | 0.8 | 13.8 | 1.4 | 1.5 | 9.0 | 212 |
| 173 | Congenital malformations of eye, ear, face and neck | 5.9 | 0.6 | 3.6 | 4.5 | 0.7 | 1.6 | 1.0 | 5.1 | 17.0 | 0.5 | 2.1 | 1.2 | 1.1 | 21.8 | 10.0 | 4.1 | 8.8 | 9.0 | 3.2 | 1.6 | 5.5 | 2.8 | 5.0 | 2.4 | 0.4 | 0.8 | 12.1 | 1.4 | 1.3 | 7.5 | 229 |
| 174 | Other congenital malformations of the digestive system | 11.3 | 0.5 | 9.7 | 6.3 | 1.1 | 2.7 | 1.5 | 16.0 | 43.5 | 1.8 | 8.9 | 5.8 | 3.4 | 29.4 | 18.6 | 11.3 | 15.5 | 22.4 | 8.1 | 3.9 | 15.5 | 8.1 | 11.3 | 8.9 | 1.8 | 1.1 | 20.4 | 2.5 | 1.2 | 9.7 | 109 |
| 175 | Congenital malformations of the sexual organs | 5.5 | 0.8 | 5.3 | 4.0 | 0.6 | 1.2 | 1.3 | 7.2 | 21.7 | 0.6 | 3.2 | 1.9 | 1.4 | 21.7 | 10.1 | 4.5 | 9.1 | 11.1 | 3.9 | 1.7 | 7.2 | 3.8 | 6.1 | 2.1 | 0.5 | 0.9 | 12.1 | 1.2 | 1.1 | 6.4 | 227 |
|  | **F – Mental and behavioural disorders** | 6.9 | 0.7 | 8.3 | 5.8 | 0.8 | 2.0 | 1.4 | 11.2 | 33.0 | 1.4 | 5.2 | 2.8 | 3.9 | 24.5 | 14.4 | 8.5 | 12.4 | 14.6 | 4.8 | 2.2 | 9.9 | 5.0 | 7.9 | 5.9 | 5.4 | 4.3 | 66.6 | 5.6 | 6.3 | 8.8 | 192 |
| 176 | Dementia ^c^ | 12.9 | 0.5 | 15.4 | 1.9 | 0.3 | 5.4 | 1.7 | 27.9 | 65.5 | 3.6 | 11.0 | 3.6 | 11.1 | 26.2 | 15.0 | 12.1 | 10.7 | 26.4 | 7.8 | 2.9 | 20.4 | 10.1 | 8.9 | 19.3 | 100.0 | 0.9 | 51.0 | 1.6 | 0.4 | 2.9 | 36 |
| 177 | Organic, including symptomatic, mental disorders | 11.2 | 1.0 | 13.7 | 4.5 | 1.4 | 4.0 | 2.2 | 22.9 | 50.5 | 3.5 | 10.0 | 4.2 | 11.1 | 27.0 | 17.0 | 13.2 | 13.7 | 20.7 | 6.9 | 2.6 | 14.8 | 7.4 | 9.9 | 12.6 | 20.5 | 7.7 | 49.1 | 5.4 | 3.0 | 7.2 | 18 |
| 178 | Mental and behavioural disorders due to use of alcohol | 7.2 | 0.9 | 8.8 | 2.8 | 0.5 | 2.0 | 1.0 | 13.1 | 34.8 | 1.9 | 5.9 | 3.2 | 5.2 | 19.9 | 15.7 | 12.3 | 13.5 | 14.4 | 5.9 | 1.6 | 9.0 | 4.1 | 7.3 | 4.9 | 2.8 | 7.0 | 42.0 | 7.5 | 5.0 | 6.0 | 104 |
| 179 | Mental and behavioural disorders due to psychoactive substance use | 7.9 | 0.7 | 7.0 | 5.0 | 0.8 | 1.4 | 0.9 | 12.7 | 27.1 | 1.7 | 7.3 | 3.7 | 2.8 | 19.3 | 16.3 | 10.9 | 14.5 | 12.4 | 4.3 | 1.9 | 8.3 | 3.7 | 7.9 | 4.4 | 1.1 | 9.9 | 33.8 | 7.2 | 9.6 | 12.3 | 119 |
| 180 | Schizophrenia ^c^ | 3.7 | 0.7 | 10.0 | 3.5 | 0.5 | 0.8 | 0.9 | 6.5 | 18.9 | 0.9 | 2.6 | 1.5 | 1.7 | 19.2 | 12.7 | 8.0 | 12.5 | 6.1 | 2.1 | 1.0 | 3.6 | 1.7 | 3.4 | 1.9 | 1.1 | 100.0 | 37.6 | 6.9 | 4.4 | 11.2 | 105 |
| 181 | Schizotypal and delusional disorders | 4.7 | 0.6 | 9.0 | 4.0 | 0.6 | 1.2 | 1.2 | 7.5 | 22.7 | 1.0 | 3.1 | 1.8 | 2.1 | 21.0 | 13.2 | 7.8 | 12.2 | 8.6 | 3.0 | 1.4 | 5.3 | 2.6 | 5.0 | 3.3 | 3.0 | 37.0 | 41.9 | 8.7 | 5.0 | 9.4 | 94 |
| 182 | Bipolar affective disorder ^c^ | 6.9 | 0.6 | 10.5 | 5.8 | 0.6 | 2.0 | 1.3 | 10.8 | 33.2 | 1.5 | 4.9 | 2.9 | 3.4 | 25.2 | 15.7 | 9.9 | 13.3 | 14.0 | 4.3 | 1.9 | 9.4 | 4.9 | 6.9 | 5.0 | 4.6 | 10.3 | 63.7 | 9.3 | 6.1 | 11.0 | 53 |
| 183 | Depression ^c^ | 7.5 | 0.7 | 9.2 | 6.9 | 0.9 | 2.3 | 1.5 | 12.0 | 37.4 | 1.5 | 5.7 | 3.2 | 4.5 | 27.0 | 15.7 | 9.4 | 13.4 | 16.4 | 5.3 | 2.5 | 11.3 | 5.8 | 8.8 | 6.9 | 4.1 | 2.4 | 100.0 | 5.1 | 3.5 | 9.7 | 173 |
| 184 | Mood (affective) disorders | 6.8 | 0.9 | 9.9 | 7.8 | 1.4 | 1.8 | 1.6 | 11.4 | 34.1 | 1.4 | 5.8 | 3.2 | 4.2 | 28.2 | 17.4 | 9.9 | 14.4 | 15.0 | 4.8 | 2.3 | 9.8 | 5.3 | 9.3 | 5.2 | 4.1 | 8.8 | 67.2 | 12.4 | 6.4 | 13.0 | 39 |
| 185 | Phobic anxiety disorders | 2.8 | 0.6 | 4.3 | 6.2 | 0.9 | 0.4 | 1.0 | 5.1 | 18.8 | 0.4 | 2.1 | 1.3 | 1.0 | 23.2 | 12.7 | 5.3 | 11.9 | 6.8 | 2.6 | 1.4 | 3.5 | 1.8 | 5.5 | 1.5 | 0.3 | 4.5 | 57.7 | 29.8 | 7.4 | 13.4 | 165 |
| 186 | Other anxiety disorders | 5.6 | 0.5 | 7.0 | 7.4 | 1.5 | 1.3 | 1.5 | 10.8 | 29.0 | 1.2 | 4.9 | 3.1 | 2.5 | 26.9 | 16.3 | 8.8 | 14.7 | 11.5 | 3.7 | 1.9 | 7.6 | 3.7 | 7.5 | 3.9 | 1.5 | 5.4 | 61.3 | 100.0 | 6.0 | 12.0 | 95 |
| 187 | Obsessive compulsive disorder (OCD) ^c^ | 2.5 | 0.5 | 3.4 | 5.3 | 1.0 | 0.4 | 1.0 | 4.1 | 14.1 | 0.4 | 1.4 | 0.9 | 1.1 | 23.5 | 10.4 | 4.2 | 11.0 | 6.1 | 2.1 | 1.0 | 3.1 | 1.5 | 4.4 | 1.4 | 0.8 | 7.0 | 59.4 | 19.1 | 9.8 | 9.8 | 183 |
| 188 | Post-traumatic stress disorder | 3.1 | 0.4 | 7.9 | 9.1 | 2.0 | 0.7 | 2.0 | 7.7 | 23.9 | 0.5 | 4.5 | 3.2 | 1.3 | 25.6 | 13.0 | 6.2 | 12.5 | 10.3 | 3.2 | 1.7 | 6.4 | 3.4 | 9.2 | 1.4 | 0.4 | 5.3 | 54.1 | 9.3 | 3.3 | 10.2 | 166 |
| 189 | Reactions to severe stress and adjustment disorders | 4.4 | 0.7 | 5.0 | 6.0 | 1.2 | 0.8 | 1.1 | 7.1 | 20.4 | 0.7 | 3.4 | 2.1 | 2.2 | 22.0 | 12.7 | 6.0 | 12.3 | 9.6 | 3.3 | 1.7 | 5.8 | 2.9 | 7.1 | 2.4 | 0.9 | 5.0 | 55.9 | 10.1 | 7.0 | 10.2 | 169 |
| 190 | Dissociative (conversion) disorders, somatoform disorders and other neurotic disorders | 6.1 | 0.6 | 8.4 | 10.4 | 2.8 | 1.5 | 2.6 | 11.2 | 32.5 | 1.0 | 5.6 | 3.8 | 3.5 | 31.4 | 18.8 | 9.4 | 16.6 | 15.6 | 5.3 | 3.0 | 10.1 | 5.0 | 12.1 | 5.1 | 1.5 | 6.3 | 42.5 | 13.4 | 3.5 | 11.8 | 67 |
| 191 | Eating disorders | 1.7 | 1.1 | 1.9 | 4.7 | 0.8 | 0.3 | 0.3 | 3.1 | 9.1 | 0.2 | 0.7 | 0.4 | 0.3 | 19.8 | 8.6 | 3.4 | 10.6 | 4.4 | 1.7 | 1.1 | 1.7 | 0.6 | 3.7 | 1.5 | 0.2 | 4.9 | 50.4 | 10.5 | 5.8 | 9.3 | 202 |
| 192 | Behavioural syndromes associated with physiological disturbances and physical factors | 5.9 | 0.9 | 4.1 | 6.5 | 1.4 | 0.7 | 1.4 | 6.9 | 19.7 | 0.7 | 3.2 | 2.0 | 1.5 | 26.6 | 11.7 | 5.2 | 11.3 | 9.8 | 3.3 | 1.9 | 5.8 | 2.8 | 6.7 | 2.2 | 0.7 | 2.6 | 30.1 | 5.6 | 5.0 | 8.6 | 200 |
| 193 | Emotionally unstable personality disorder | 2.6 | 0.7 | 5.4 | 7.8 | 1.1 | 0.5 | 0.8 | 5.2 | 17.5 | 0.4 | 2.1 | 1.3 | 1.1 | 26.4 | 17.6 | 7.3 | 16.9 | 8.0 | 2.6 | 1.5 | 4.5 | 2.3 | 6.1 | 1.3 | 0.2 | 13.8 | 57.7 | 13.4 | 12.1 | 16.0 | 73 |
| 194 | Specific personality disorders | 3.6 | 0.7 | 2.4 | 6.8 | 1.0 | 0.8 | 1.2 | 6.5 | 21.7 | 0.6 | 3.0 | 1.9 | 1.6 | 24.8 | 16.0 | 7.6 | 14.3 | 9.4 | 3.0 | 1.5 | 5.7 | 2.9 | 6.7 | 2.6 | 0.9 | 10.0 | 54.6 | 14.2 | 8.6 | 12.3 | 108 |
| 195 | Disorders of adult personality and behaviour | 3.7 | 0.8 | 7.2 | 7.5 | 1.1 | 0.8 | 1.4 | 6.7 | 22.0 | 0.7 | 3.4 | 2.3 | 1.5 | 25.2 | 15.4 | 7.1 | 14.4 | 9.6 | 3.3 | 1.6 | 5.9 | 3.0 | 7.4 | 2.3 | 0.8 | 8.8 | 54.6 | 14.3 | 9.3 | 12.5 | 85 |
| 196 | Mental retardation | 3.2 | 0.8 | 7.2 | 2.6 | 0.3 | 1.4 | 0.4 | 4.8 | 16.7 | 0.8 | 1.5 | 0.8 | 1.7 | 23.4 | 9.6 | 5.4 | 10.1 | 7.2 | 2.0 | 0.7 | 4.3 | 1.8 | 4.7 | 2.5 | 2.6 | 10.5 | 28.4 | 4.7 | 8.4 | 7.9 | 156 |
| 197 | Disorders of psychological development | 1.5 | 1.1 | 2.5 | 2.8 | 0.3 | 0.4 | 0.5 | 2.8 | 7.7 | 0.3 | 0.8 | 0.4 | 0.9 | 18.4 | 6.4 | 3.0 | 9.1 | 3.2 | 1.1 | 0.6 | 1.6 | 0.7 | 3.3 | 1.0 | 0.6 | 8.2 | 33.2 | 6.2 | 22.9 | 6.2 | 203 |
| 198 | Hyperkinetic disorders (ADHD) ^c^ | 2.1 | 0.6 | 2.4 | 4.7 | 0.6 | 0.3 | 0.6 | 3.5 | 11.0 | 0.2 | 1.2 | 0.8 | 0.7 | 19.6 | 10.6 | 4.2 | 12.1 | 5.5 | 1.7 | 0.9 | 3.3 | 1.5 | 4.6 | 0.9 | 0.3 | 3.0 | 37.3 | 5.3 | 100.0 | 7.2 | 217 |
| 199 | Behavioural and emotional disorders with onset usually occurring in childhood and adolescence | 4.0 | 0.7 | 6.2 | 5.4 | 0.9 | 1.0 | 1.0 | 7.4 | 20.3 | 0.9 | 3.2 | 1.9 | 2.1 | 22.1 | 13.5 | 7.4 | 13.0 | 8.7 | 2.9 | 1.4 | 5.4 | 2.7 | 5.9 | 2.7 | 1.9 | 15.9 | 47.7 | 10.9 | 12.5 | 10.6 | 106 |
|  |  |  |  |  |  |  |  |  |  |  |  |  |  |  |  |  |  |  |  |  |  |  |  |  |  |  |  |  |  |  |  |  |
|  | **Total population** | **7.7** | **0.8** | **8.1** | **5.0** | **0.6** | **2.3** | **1.3** | **10.6** | **35.5** | **1.3** | **4.7** | **2.6** | **2.4** | **28.2** | **14.0** | **7.2** | **12.1** | **16.9** | **5.5** | **2.6** | **11.3** | **6.0** | **7.1** | **5.3** | **1.2** | **1.0** | **15.2** | **1.3** | **1.4** | **7.4** | **n/a** |
|  |  |  |  |  |  |  |  |  |  |  |  |  |  |  |  |  |  |  |  |  |  |  |  |  |  |  |  |  |  |  |  |  |
|  | Depression medicine ^c^ ** | 7.9 | 0.7 | 9.5 | 7.0 | 1.1 | 2.5 | 1.5 | 12.2 | 38.4 | 1.5 | 5.8 | 3.3 | 4.4 | 27.0 | 15.8 | 9.5 | 13.3 | 17.0 | 5.5 | 2.6 | 11.8 | 6.0 | 10.1 | 7.1 | 3.9 | 2.2 | 70.9 | 4.7 | 3.1 | 9.2 | 189 |
|  | Antipsychotic medicine ^c^ ** | 6.3 | 0.6 | 10.2 | 6.3 | 1.0 | 1.9 | 1.3 | 10.5 | 33.3 | 1.3 | 4.7 | 2.6 | 3.3 | 25.1 | 15.5 | 9.8 | 13.8 | 13.1 | 4.2 | 1.8 | 8.9 | 4.6 | 7.3 | 5.5 | 6.0 | 16.0 | 57.2 | 8.3 | 7.4 | 9.9 | 142 |
|  | Indication prescrib anxiety medicine ^c^ ** | 7.0 | 0.6 | 8.6 | 7.1 | 0.9 | 2.0 | 1.4 | 11.5 | 36.1 | 1.4 | 5.4 | 3.1 | 3.7 | 27.9 | 16.6 | 9.8 | 14.3 | 14.4 | 4.6 | 2.2 | 9.9 | 5.0 | 8.5 | 6.2 | 3.3 | 3.6 | 61.4 | 12.1 | 4.4 | 9.9 | 186 |
|  | Heart failure medication ^c^ ** | 14.0 | 0.8 | 24.0 | 2.3 | 0.3 | 5.8 | 1.7 | 88.0 | 97.0 | 52.0 | 52.5 | 24.2 | 7.9 | 23.1 | 19.3 | 19.4 | 14.8 | 28.8 | 14.0 | 3.2 | 18.3 | 9.9 | 9.2 | 9.6 | 2.8 | 0.6 | 17.7 | 1.0 | 0.3 | 8.6 | 16 |
|  | Ischaemic heart medication ^c^ ** | 13.9 | 0.6 | 23.1 | 3.3 | 0.5 | 6.5 | 2.5 | 72.9 | 85.7 | 12.7 | 42.3 | 26.3 | 7.5 | 27.8 | 21.2 | 18.1 | 16.9 | 31.0 | 12.3 | 3.6 | 21.8 | 11.6 | 10.8 | 12.4 | 4.0 | 0.5 | 19.1 | 1.1 | 0.3 | 8.7 | 35 |
|  | All five types of the medicine above | **8.6** | **0.6** | **11.3** | **6.1** | **0.9** | 3.0 | **1.6** | **20.8** | **44.6** | **3.3** | **10.9** | **6.4** | **4.5** | **26.3** | **16.1** | **10.4** | **13.4** | **18.5** | **6.3** | **2.6** | **12.8** | **6.6** | **9.7** | **7.5** | **3.6** | **3.5** | **56.0** | **4.0** | **3.0** | **8.7** | 178 |
|  |  |  |  |  |  |  |  |  |  |  |  |  |  |  |  |  |  |  |  |  |  |  |  |  |  |  |  |  |  |  |  |  |
|  | **Extra** |  |  |  |  |  |  |  |  |  |  |  |  |  |  |  |  |  |  |  |  |  |  |  |  |  |  |  |  |  |  |  |
|  | Ischaemic Heart Diseases | 13.0 | 0.7 | 18.0 | 3.4 | 0.5 | 5.1 | 1.9 | 100.0 | 75.8 | 11.9 | 44.1 | 24.8 | 7.2 | 24.9 | 17.6 | 14.5 | 14.0 | 27.3 | 10.6 | 3.4 | 19.1 | 10.2 | 9.9 | 10.2 | 3.3 | 0.6 | 17.3 | 1.3 | 0.5 | 8.3 | 57 |
|  | Artritis | 10.0 | 0.6 | 12.6 | 4.7 | 0.5 | 3.8 | 1.8 | 17.0 | 50.4 | 2.4 | 8.0 | 4.5 | 3.5 | 26.2 | 15.2 | 9.2 | 11.8 | 100.0 | 32.1 | 15.3 | 66.9 | 35.4 | 12.1 | 9.7 | 1.9 | 0.4 | 14.8 | 0.9 | 0.5 | 8.4 | 149 |
|  | Arthrosis | 10.7 | 0.4 | 12.7 | 4.7 | 0.5 | 4.3 | 1.9 | 17.8 | 53.3 | 2.2 | 8.3 | 4.8 | 3.7 | 26.7 | 15.6 | 9.6 | 12.1 | 100.0 | 11.3 | 5.3 | 100.0 | 52.9 | 13.4 | 10.2 | 2.2 | 0.3 | 15.2 | 0.9 | 0.4 | 9.1 | 130 |
|  | Backconditions | 8.5 | 0.5 | 9.8 | 6.3 | 1.0 | 3.0 | 1.9 | 14.7 | 41.9 | 1.7 | 7.6 | 4.6 | 3.2 | 27.7 | 16.1 | 9.6 | 12.8 | 28.7 | 9.5 | 4.9 | 21.3 | 10.3 | 100.0 | 9.6 | 1.5 | 0.5 | 18.9 | 1.3 | 0.9 | 8.4 | 122 |
|  | Overweight | 6.2 | 0.6 | 17.7 | 6.6 | 0.7 | 1.6 | 1.0 | 11.8 | 39.7 | 1.8 | 6.3 | 3.8 | 2.0 | 26.6 | 17.0 | 7.5 | 14.4 | 19.3 | 6.5 | 2.6 | 13.9 | 8.8 | 8.1 | 2.8 | 0.5 | 1.5 | 19.9 | 2.1 | 1.4 | 100.0 | 222 |
|  | Endometriosis | 5.7 | 0.4 | 4.3 | 10.3 | 1.1 | 0.7 | 0.9 | 5.1 | 25.5 | 0.3 | 1.9 | 1.4 | 1.1 | 28.8 | 13.8 | 4.6 | 11.2 | 12.5 | 4.1 | 2.8 | 7.4 | 3.9 | 7.8 | 2.9 | 0.1 | 0.5 | 17.8 | 1.6 | 1.0 | 14.4 | 231 |

^c^ = complex defined conditions, see reference for further details [64].

** 2-year prevalence. n/a: not available.
