# Supplementary material for: Catalogue of multimorbidity mean based severity and associational prevalence rates between 199+ chronic conditions—A nationwide register-based population study
Source: PLoS One. 2022 Sep 14;17(9):e0273850. doi: 10.1371/journal.pone.0273850 (PMC9473636; doi:10.1371/journal.pone.0273850)
Supplement: S7 Table — Means by age and sex groups in Denmark on 1 January 2013. Sorted by ICD-10 codes. (DOC) [file pone.0273850.s007.doc]

***S7 Table.*** Catalogue of raw mean NCCs and SDs of 199 conditions: means by age and sex groups in Denmark on 1 January 2013. Sorted by ICD-10 codes.

| **No.** | **Name of condition** | **Men 16-24** | | **Men 25-34** | | **Men 35-44** | | **Men 45-54** | | **Men 55-64** | | **Men 65-74** | | **Men 75+** | | **Women**  **16-24** | | **Women**  **25-34** | | **Women**  **35-44** | | **Women**  **45-54** | | **Women**  **55-64** | | **Women**  **65-74** | | **Women**  **75+** | |
| --- | --- | --- | --- | --- | --- | --- | --- | --- | --- | --- | --- | --- | --- | --- | --- | --- | --- | --- | --- | --- | --- | --- | --- | --- | --- | --- | --- | --- | --- |
|  |  | Raw | SD | Raw | SD | Raw | SD | Raw | SD | Raw | SD | Raw | SD | Raw | SD | Raw | SD | Raw | SD | Raw | SD | Raw | SD | Raw | SD | Raw | SD | Raw | SD |
|  | **B – Viral hepatitis and human immunodeficiency virus [HIV] disease** | **2.5** | **2.3** | **3.3** | **2.8** | **3.9** | **3.2** | **4.2** | **3.2** | **5.3** | **3.7** | **5.9** | **3.8** | **8.6** | **4.0** | **2.4** | **2.0** | **2.9** | **2.7** | **3.6** | **3.0** | **4.7** | **3.6** | **5.9** | **3.8** | **6.9** | **4.3** | **8.4** | **4.7** |
| 1 | Chronic viral hepatitis | 2.4 | 2.2 | 3.8 | 3.2 | 4.8 | 3.6 | 5.3 | 3.6 | 6.0 | 3.8 | 7.1 | 4.0 | 10.9 | 4.0 | 2.7 | 2.3 | 3.2 | 3.0 | 4.3 | 3.5 | 5.6 | 4.1 | 6.2 | 3.9 | 7.6 | 4.7 | 8.6 | 5.1 |
| 2 | Human immunodeficiency virus [HIV] disease | 2.8 | 2.3 | 2.8 | 2.3 | 3.4 | 2.8 | 3.7 | 2.7 | 4.9 | 3.7 | 5.5 | 3.6 | 7.2 | 3.1 | 2.1 | 1.3 | 2.4 | 2.1 | 3.3 | 3.2 | 4.2 | 3.3 | 5.6 | 3.9 | 6.0 | 3.7 | 8.2 | 4.0 |
|  | **C – Malignant neoplasms** | **2.5** | **1.8** | **2.8** | **2.0** | **3.1** | **2.3** | **3.9** | **2.8** | **4.8** | **3.3** | **5.6** | **3.5** | **6.9** | **3.8** | **2.9** | **2.2** | **3.2** | **2.4** | **3.5** | **2.5** | **4.0** | **2.9** | **4.8** | **3.2** | **5.6** | **3.5** | **7.1** | **3.8** |
| 3 | Malignant neoplasms of other and unspecified localizations | 2.9 | 2.1 | 3.1 | 2.0 | 3.7 | 2.5 | 4.5 | 2.9 | 5.4 | 3.4 | 6.4 | 3.8 | 7.5 | 3.9 | 3.0 | 2.1 | 3.6 | 2.3 | 3.9 | 2.5 | 4.7 | 3.1 | 5.8 | 3.6 | 6.6 | 3.8 | 7.9 | 3.9 |
| 4 | Malignant neoplasms of digestive organs | 4.7 | 3.5 | 4.7 | 2.7 | 4.3 | 2.4 | 4.7 | 3.1 | 6.0 | 3.7 | 6.9 | 3.9 | 8.1 | 4.2 | 2.3 | 1.1 | 4.4 | 3.1 | 4.9 | 3.3 | 5.7 | 3.4 | 6.4 | 3.9 | 7.0 | 4.0 | 8.0 | 4.0 |
| 5 | Malignant neoplasm of colon | 1.9 | 0.8 | 3.2 | 2.3 | 3.6 | 2.8 | 4.1 | 2.9 | 5.1 | 3.5 | 6.0 | 3.7 | 7.5 | 3.9 | 3.0 | 2.7 | 3.5 | 2.5 | 4.3 | 2.6 | 4.4 | 3.1 | 5.3 | 3.4 | 6.1 | 3.8 | 7.4 | 3.9 |
| 6 | Malignant neoplasms of rectosigmoid junction, rectum, anus and anal canal | n/a | n/a | 3.6 | 2.2 | 3.4 | 2.4 | 4.1 | 2.7 | 4.7 | 3.0 | 5.5 | 3.4 | 6.8 | 3.6 | n/a | n/a | 2.6 | 1.5 | 3.9 | 2.4 | 4.2 | 2.9 | 4.8 | 3.0 | 5.6 | 3.5 | 7.1 | 3.8 |
| 7 | Malignant neoplasm of bronchus and lung | 4.2 | 3.2 | 2.9 | 2.0 | 4.3 | 3.2 | 4.9 | 3.3 | 6.1 | 3.7 | 7.0 | 3.9 | 8.3 | 4.2 | 4.1 | 3.3 | 4.2 | 2.5 | 4.9 | 3.2 | 5.9 | 3.7 | 6.6 | 3.9 | 7.4 | 3.9 | 8.8 | 4.1 |
| 8 | Malignant melanoma of skin | 1.9 | 1.3 | 2.2 | 1.6 | 2.4 | 1.7 | 3.0 | 2.2 | 4.0 | 2.7 | 5.2 | 3.2 | 7.0 | 3.7 | 2.2 | 1.5 | 2.5 | 1.8 | 2.8 | 1.9 | 3.2 | 2.3 | 4.0 | 2.8 | 5.1 | 3.2 | 6.7 | 3.6 |
| 9 | Other malignant neoplasms of skin | 2.0 | 1.3 | 2.5 | 2.1 | 2.9 | 2.4 | 3.2 | 2.6 | 4.5 | 3.3 | 5.5 | 3.6 | 7.1 | 3.9 | 3.2 | 2.0 | 2.9 | 2.3 | 3.0 | 2.4 | 3.8 | 2.9 | 4.7 | 3.2 | 5.6 | 3.5 | 7.2 | 3.9 |
| 10 | Malignant neoplasm of breast | n/a | n/a | n/a | n/a | 4.0 | 3.0 | 3.4 | 2.4 | 5.0 | 3.5 | 5.7 | 3.8 | 7.5 | 3.5 | 1.5 | 0.8 | 2.8 | 2.0 | 3.2 | 2.2 | 3.7 | 2.5 | 4.5 | 3.0 | 5.4 | 3.3 | 7.0 | 3.7 |
| 11 | Malignant neoplasms of female genital organs | n/a | n/a | n/a | n/a | n/a | n/a | n/a | n/a | n/a | n/a | n/a | n/a | n/a | n/a | 2.5 | 1.7 | 3.3 | 2.6 | 3.5 | 2.5 | 4.1 | 2.9 | 4.8 | 3.2 | 5.7 | 3.3 | 7.1 | 3.7 |
| 12 | Malignant neoplasm of cervix uteri, corpus uteri and part unspecified | n/a | n/a | n/a | n/a | n/a | n/a | n/a | n/a | n/a | n/a | n/a | n/a | n/a | n/a | 2.9 | 2.0 | 2.8 | 2.2 | 3.3 | 2.4 | 3.8 | 2.6 | 4.6 | 3.1 | 5.3 | 3.3 | 6.8 | 3.5 |
| 13 | Malignant tumour of male genitalia | 2.2 | 1.5 | 2.5 | 1.7 | 2.7 | 2.1 | 3.3 | 2.5 | 4.3 | 3.0 | 5.6 | 3.6 | 7.3 | 4.3 | n/a | n/a | n/a | n/a | n/a | n/a | n/a | n/a | n/a | n/a | n/a | n/a | n/a | n/a |
| 14 | Malignant neoplasm of prostate | n/a | n/a | n/a | n/a | 3.3 | 1.6 | 3.4 | 2.3 | 4.2 | 2.9 | 5.1 | 3.2 | 6.6 | 3.7 | n/a | n/a | n/a | n/a | n/a | n/a | n/a | n/a | n/a | n/a | 6.8 | 5.4 | n/a | n/a |
| 15 | Malignant neoplasms of urinary tract | 2.0 | 1.5 | 3.1 | 2.0 | 3.8 | 2.3 | 4.3 | 2.9 | 5.1 | 3.3 | 5.9 | 3.5 | 7.2 | 3.9 | 2.1 | 1.3 | 3.3 | 1.9 | 4.5 | 2.8 | 5.1 | 3.3 | 5.7 | 3.6 | 6.5 | 3.7 | 7.6 | 3.8 |
| 16 | Brain cancer ^c^ | 3.3 | 1.9 | 3.8 | 2.4 | 4.2 | 2.4 | 4.8 | 3.0 | 6.1 | 3.5 | 7.0 | 3.7 | 8.3 | 4.0 | 3.9 | 2.6 | 4.5 | 3.0 | 4.9 | 3.1 | 5.5 | 3.5 | 6.3 | 3.7 | 7.1 | 3.9 | 8.3 | 4.0 |
| 17 | Malignant neoplasms of ill-defined, secondary and unspecified sites, and of independent (primary) multiple sites | 3.2 | 1.6 | 3.8 | 2.0 | 4.0 | 2.5 | 4.8 | 2.7 | 5.9 | 3.3 | 7.0 | 3.6 | 8.1 | 3.8 | 3.5 | 2.1 | 4.1 | 2.4 | 4.5 | 2.6 | 5.1 | 2.9 | 6.0 | 3.2 | 6.9 | 3.6 | 8.2 | 3.7 |
| 18 | Malignant neoplasms, stated or presumed to be primary, of lymphoid, haematopoietic and related tissue | 2.4 | 1.8 | 3.0 | 2.2 | 3.5 | 2.5 | 4.3 | 3.0 | 5.1 | 3.4 | 6.2 | 3.6 | 7.6 | 3.9 | 2.9 | 2.2 | 3.8 | 2.7 | 4.0 | 2.8 | 4.7 | 3.3 | 5.6 | 3.5 | 6.4 | 3.7 | 7.7 | 3.9 |
|  | **D – In situ and benign neoplasms, and neoplasms of uncertain or unknown behaviour and diseases of the blood and blood-forming organs and certain disorders involving the immune mechanism** | **2.6** | **2.0** | **3.3** | **2.6** | **4.2** | **3.1** | **5.4** | **3.6** | **6.7** | **4.1** | **7.9** | **4.3** | **9.0** | **4.2** | **2.9** | **2.3** | **3.3** | **2.6** | **3.8** | **3.0** | **4.8** | **3.5** | **6.2** | **4.1** | **7.7** | **4.4** | **8.9** | **4.1** |
| 19 | In situ neoplasms | 2.9 | 2.5 | 2.5 | 1.6 | 3.3 | 2.7 | 3.9 | 2.7 | 4.8 | 3.3 | 6.1 | 3.7 | 7.6 | 4.0 | 2.4 | 1.9 | 2.8 | 2.1 | 3.0 | 2.2 | 3.7 | 2.6 | 4.7 | 3.1 | 5.8 | 3.5 | 7.5 | 3.8 |
| 20 | Haemolytic anaemias | 2.6 | 1.8 | 3.4 | 2.7 | 3.9 | 2.5 | 5.0 | 3.8 | 6.0 | 4.3 | 8.0 | 4.2 | 9.2 | 4.4 | 2.6 | 1.9 | 3.2 | 2.4 | 3.7 | 3.0 | 5.2 | 3.7 | 7.0 | 4.5 | 8.1 | 4.3 | 8.9 | 3.8 |
| 21 | Aplastic and other anaemias | 4.2 | 2.6 | 4.3 | 2.9 | 5.4 | 3.5 | 6.9 | 4.0 | 8.0 | 4.5 | 8.8 | 4.4 | 1n/a | 4.4 | 3.9 | 3.0 | 3.8 | 3.1 | 4.2 | 3.3 | 5.2 | 3.9 | 7.0 | 4.3 | 9.1 | 4.6 | 9.5 | 4.3 |
| 22 | Other anaemias | 3.6 | 2.5 | 4.4 | 3.5 | 5.7 | 3.9 | 6.6 | 4.3 | 7.8 | 4.5 | 8.8 | 4.5 | 9.4 | 4.3 | 3.1 | 2.5 | 4.1 | 3.4 | 4.6 | 3.6 | 5.3 | 3.9 | 7.4 | 4.6 | 9.0 | 4.6 | 9.2 | 4.2 |
| 23 | Coagulation defects, purpura and other haemorrhagic conditions | 2.4 | 1.8 | 3.1 | 2.6 | 4.2 | 3.1 | 5.3 | 3.4 | 6.4 | 4.0 | 7.6 | 4.4 | 9.4 | 4.4 | 2.8 | 2.3 | 3.4 | 2.6 | 3.9 | 3.1 | 5.3 | 3.6 | 7.0 | 4.4 | 8.2 | 4.6 | 9.4 | 4.3 |
| 24 | Other diseases of blood and blood-forming organs | 2.8 | 2.1 | 3.7 | 3.2 | 4.8 | 3.4 | 5.7 | 3.6 | 6.6 | 3.9 | 7.6 | 4.1 | 8.9 | 4.2 | 3.5 | 2.6 | 4.2 | 2.9 | 5.1 | 3.7 | 5.7 | 3.7 | 6.8 | 4.0 | 7.8 | 4.3 | 8.9 | 4.2 |
| 25 | Certain disorders involving the immune mechanism | 3.0 | 2.1 | 3.2 | 2.3 | 3.8 | 2.8 | 5.0 | 3.4 | 6.3 | 3.8 | 8.2 | 4.6 | 9.2 | 4.4 | 3.5 | 2.3 | 3.8 | 2.6 | 4.7 | 3.4 | 5.9 | 3.9 | 7.3 | 4.2 | 7.9 | 4.0 | 9.6 | 4.5 |
|  | **E – Endocrine, nutritional and metabolic diseases** | **2.3** | **1.7** | **3.2** | **2.4** | **3.7** | **2.6** | **4.2** | **2.7** | **4.8** | **2.9** | **5.4** | **3.2** | **6.8** | **3.6** | **2.6** | **2.2** | **3.2** | **2.6** | **3.8** | **2.8** | **4.5** | **3.0** | **4.9** | **3.1** | **5.4** | **3.3** | **6.8** | **3.6** |
| 26 | Diseases of the thyroid ^c^ | 2.7 | 1.8 | 3.0 | 2.3 | 3.5 | 2.7 | 4.1 | 2.9 | 5.0 | 3.5 | 6.2 | 3.8 | 7.6 | 4.1 | 2.6 | 2.2 | 3.0 | 2.4 | 3.4 | 2.5 | 3.9 | 2.8 | 4.9 | 3.2 | 5.7 | 3.5 | 7.2 | 3.8 |
| 27 | Thyrotoxicosis ^c^ | 2.2 | 1.4 | 2.8 | 2.1 | 3.3 | 2.6 | 3.9 | 3.0 | 5.2 | 3.5 | 6.5 | 3.9 | 7.9 | 4.1 | 2.5 | 1.9 | 3.0 | 2.2 | 3.3 | 2.3 | 3.9 | 2.8 | 4.8 | 3.2 | 5.9 | 3.6 | 7.2 | 3.7 |
| 28 | Diabetes type 1 ^c^ | 2.0 | 1.4 | 2.8 | 1.9 | 3.6 | 2.5 | 4.5 | 2.8 | 5.3 | 2.9 | 6.3 | 3.3 | 7.2 | 3.4 | 2.4 | 1.9 | 3.8 | 2.6 | 4.5 | 2.9 | 5.3 | 3.1 | 6.3 | 3.4 | 6.9 | 3.4 | 7.6 | 3.3 |
| 29 | Diabetes type 2 ^c^ | 2.7 | 2.1 | 3.9 | 3.0 | 4.2 | 2.8 | 4.8 | 2.9 | 5.5 | 3.1 | 6.2 | 3.4 | 7.4 | 3.7 | 4.0 | 3.5 | 3.8 | 3.1 | 4.4 | 3.1 | 5.6 | 3.4 | 6.2 | 3.5 | 6.6 | 3.5 | 7.5 | 3.7 |
| 30 | Diabetes others ^c^ | 2.2 | 1.7 | 3.0 | 1.9 | 3.6 | 2.7 | 5.6 | 4.2 | 6.6 | 3.6 | 7.1 | 3.7 | 9.4 | 4.3 | 3.4 | 3.3 | 3.4 | 2.3 | 3.5 | 2.6 | 5.8 | 3.4 | 7.5 | 4.3 | 9.1 | 5.3 | 9.0 | 4.6 |
| 31 | Disorders of other endocrine glands | 2.6 | 2.0 | 3.9 | 2.8 | 4.4 | 3.0 | 5.8 | 3.7 | 6.9 | 4.0 | 8.1 | 4.3 | 9.6 | 4.4 | 2.8 | 2.3 | 3.3 | 2.6 | 4.3 | 3.1 | 5.6 | 3.7 | 6.7 | 4.1 | 7.8 | 4.1 | 9.4 | 4.3 |
| 32 | Metabolic disorders | 2.6 | 1.8 | 3.7 | 2.8 | 4.4 | 3.1 | 5.5 | 3.8 | 7.0 | 4.2 | 8.1 | 4.4 | 9.6 | 4.5 | 3.5 | 2.4 | 4.1 | 2.9 | 4.5 | 3.2 | 5.7 | 3.7 | 6.9 | 4.0 | 8.2 | 4.3 | 9.4 | 4.1 |
| 33 | Disturbances in lipoprotein circulation and other lipids ^c^ | 3.1 | 2.5 | 3.8 | 2.6 | 4.1 | 2.7 | 4.3 | 2.8 | 4.8 | 2.9 | 5.5 | 3.2 | 6.9 | 3.6 | 3.5 | 2.8 | 4.9 | 3.7 | 5.3 | 3.4 | 5.2 | 3.2 | 5.2 | 3.2 | 5.5 | 3.3 | 6.9 | 3.6 |
| 34 | Cystic fibrosis ^c^ | 3.1 | 1.7 | 2.8 | 2.2 | 3.7 | 2.7 | 4.4 | 3.2 | 5.7 | 3.5 | 6.4 | 4.3 | 7.1 | 3.6 | 3.8 | 2.3 | 3.1 | 2.4 | 3.4 | 2.4 | 5.0 | 3.7 | 6.9 | 4.7 | 7.1 | 4.2 | 8.8 | 4.1 |
|  | **G – Diseases of the nervous system** | **2.6** | **2.0** | **3.2** | **2.4** | **3.5** | **2.6** | **4.2** | **3.0** | **5.2** | **3.4** | **6.4** | **3.8** | **7.8** | **4.0** | **2.9** | **2.2** | **3.5** | **2.7** | **3.8** | **2.7** | **4.3** | **3.0** | **5.2** | **3.4** | **6.4** | **3.8** | **7.9** | **3.9** |
| 35 | Inflammatory diseases of the central nervous system | 3.0 | 2.7 | 3.3 | 2.5 | 3.8 | 2.8 | 4.6 | 3.5 | 6.2 | 3.9 | 7.3 | 4.0 | 8.5 | 4.4 | 3.0 | 2.6 | 3.6 | 2.6 | 4.6 | 3.4 | 5.3 | 3.7 | 6.3 | 3.9 | 7.7 | 4.4 | 8.5 | 4.0 |
| 36 | Systemic atrophies primarily affecting the central nervous system and other degenerative diseases | 3.4 | 2.0 | 4.0 | 2.6 | 4.4 | 3.3 | 5.5 | 3.7 | 6.4 | 3.9 | 7.4 | 4.0 | 8.2 | 3.8 | 3.8 | 2.8 | 3.5 | 2.5 | 4.4 | 3.6 | 6.0 | 4.5 | 7.0 | 4.0 | 7.5 | 3.9 | 8.2 | 3.7 |
| 37 | Parkinson’s disease ^c^ | 5.2 | 2.5 | 5.8 | 3.1 | 5.8 | 3.3 | 5.8 | 3.6 | 6.2 | 3.9 | 6.8 | 4.1 | 7.8 | 4.1 | 6.4 | 3.5 | 7.2 | 4.2 | 6.2 | 4.1 | 6.5 | 4.1 | 7.0 | 4.2 | 7.3 | 4.2 | 8.1 | 4.0 |
| 38 | Extrapyramidal and movement disorders | 3.4 | 2.7 | 4.1 | 2.9 | 4.9 | 3.4 | 5.5 | 3.8 | 6.3 | 3.9 | 7.5 | 4.2 | 8.9 | 4.4 | 4.0 | 2.9 | 4.9 | 3.5 | 5.3 | 3.6 | 5.9 | 3.9 | 6.6 | 4.2 | 7.7 | 4.4 | 8.8 | 4.1 |
| 39 | Sclerosis | 3.1 | 2.3 | 3.3 | 2.1 | 3.6 | 2.6 | 4.0 | 2.7 | 4.8 | 3.2 | 5.6 | 3.4 | 6.3 | 3.7 | 3.4 | 2.0 | 3.7 | 2.2 | 4.1 | 2.7 | 4.5 | 2.9 | 5.1 | 3.3 | 5.8 | 3.5 | 6.7 | 3.9 |
| 40 | Demyelinating diseases of the central nervous system | 3.7 | 2.3 | 3.7 | 2.5 | 4.1 | 2.7 | 4.9 | 3.4 | 5.9 | 3.5 | 7.2 | 3.9 | 8.8 | 4.5 | 3.7 | 2.8 | 4.1 | 2.4 | 4.7 | 2.9 | 5.1 | 3.1 | 6.1 | 3.4 | 7.3 | 3.9 | 8.7 | 4.1 |
| 41 | Epilepsy ^c^ | 2.9 | 2.2 | 3.9 | 3.0 | 4.6 | 3.3 | 5.4 | 3.7 | 6.4 | 4.0 | 7.6 | 4.2 | 8.6 | 4.3 | 3.2 | 2.7 | 4.7 | 3.6 | 5.3 | 3.8 | 6.0 | 4.1 | 7.0 | 4.3 | 8.0 | 4.3 | 8.9 | 4.2 |
| 42 | Migraine ^c^ | 2.4 | 1.8 | 3.0 | 2.3 | 3.4 | 2.5 | 3.9 | 2.9 | 4.8 | 3.4 | 5.9 | 3.8 | 7.7 | 4.2 | 2.6 | 2.0 | 3.3 | 2.5 | 3.6 | 2.6 | 4.1 | 2.9 | 4.9 | 3.3 | 6.0 | 3.7 | 7.7 | 4.1 |
| 43 | Other headache syndromes | 2.9 | 2.2 | 3.4 | 2.4 | 4.1 | 2.8 | 5.0 | 3.2 | 6.3 | 4.0 | 7.8 | 4.4 | 9.9 | 4.5 | 3.2 | 2.1 | 4.3 | 2.9 | 5.0 | 3.2 | 5.7 | 3.4 | 6.8 | 4.0 | 8.1 | 4.4 | 9.8 | 4.6 |
| 44 | Transient cerebral ischaemic attacks and related syndromes and vascular syndromes of brain in cerebrovascular diseases | 3.8 | 3.0 | 3.8 | 2.6 | 4.5 | 3.0 | 5.3 | 3.3 | 6.1 | 3.6 | 6.9 | 3.8 | 8.4 | 4.0 | 4.6 | 3.4 | 5.0 | 3.3 | 5.0 | 3.4 | 5.7 | 3.6 | 6.4 | 3.9 | 7.1 | 3.9 | 8.5 | 3.9 |
| 45 | Sleep disorders | 3.0 | 2.3 | 3.1 | 2.4 | 3.6 | 2.7 | 4.4 | 3.1 | 5.6 | 3.4 | 7.0 | 4.1 | 9.0 | 4.3 | 3.6 | 2.7 | 4.6 | 3.2 | 5.4 | 3.6 | 5.9 | 3.8 | 6.5 | 3.9 | 7.7 | 4.2 | 9.4 | 4.5 |
| 46 | Disorders of trigeminal nerve and facial nerve disorders | 2.1 | 1.8 | 2.6 | 2.1 | 2.9 | 2.4 | 3.9 | 2.9 | 5.2 | 3.5 | 6.4 | 4.0 | 8.1 | 4.2 | 2.6 | 2.1 | 3.3 | 2.6 | 3.9 | 3.0 | 4.7 | 3.5 | 5.8 | 3.8 | 6.9 | 4.2 | 8.2 | 4.2 |
| 47 | Disorders of other cranial nerves, cranial nerve disorders in diseases classified elsewhere, nerve root and plexus disorders and nerve root and plexus compressions in diseases classified elsewhere | 3.0 | 2.1 | 3.6 | 2.7 | 4.0 | 2.9 | 4.6 | 3.1 | 5.6 | 3.5 | 7.1 | 3.9 | 8.6 | 4.4 | 3.7 | 2.2 | 4.1 | 2.5 | 4.7 | 3.0 | 5.3 | 3.3 | 6.5 | 4.0 | 7.9 | 4.4 | 9.1 | 4.4 |
| 48 | Mononeuropathies of upper limb | 2.2 | 1.6 | 2.7 | 2.1 | 3.2 | 2.5 | 4.0 | 2.9 | 5.1 | 3.5 | 6.4 | 3.9 | 7.9 | 4.1 | 3.0 | 2.1 | 3.5 | 2.6 | 3.8 | 2.7 | 4.5 | 3.1 | 5.2 | 3.4 | 6.3 | 3.8 | 7.9 | 4.1 |
| 49 | Mononeuropathies of lower limb, other mononeuropathies and mononeuropathy in diseases classified elsewhere | 2.6 | 1.9 | 3.0 | 2.2 | 3.6 | 2.8 | 4.1 | 3.0 | 5.5 | 3.6 | 6.9 | 4.1 | 8.5 | 4.4 | 3.2 | 2.5 | 3.7 | 2.7 | 4.3 | 3.0 | 5.0 | 3.4 | 6.0 | 3.7 | 7.0 | 4.0 | 8.7 | 4.2 |
| 50 | Polyneuropathies and other disorders of the peripheral nervous system | 3.1 | 2.4 | 3.7 | 2.6 | 4.4 | 3.2 | 5.7 | 3.7 | 6.8 | 4.0 | 7.7 | 4.3 | 8.9 | 4.4 | 3.4 | 2.7 | 4.5 | 3.3 | 5.3 | 3.6 | 6.3 | 4.1 | 7.5 | 4.5 | 8.3 | 4.5 | 9.4 | 4.4 |
| 51 | Diseases of myoneural junction and muscle | 3.1 | 2.1 | 3.6 | 2.6 | 3.8 | 2.9 | 4.9 | 3.6 | 6.5 | 4.1 | 7.4 | 4.3 | 7.9 | 4.1 | 3.5 | 2.8 | 4.0 | 3.1 | 4.4 | 3.1 | 5.4 | 3.6 | 6.8 | 4.3 | 7.9 | 4.7 | 9.0 | 4.4 |
| 52 | Cerebral palsy and other paralytic syndromes | 3.5 | 2.3 | 3.9 | 2.6 | 4.4 | 3.0 | 5.5 | 3.6 | 6.6 | 4.0 | 7.8 | 4.3 | 8.9 | 4.3 | 3.8 | 2.5 | 4.7 | 3.2 | 5.3 | 3.5 | 6.3 | 4.2 | 7.2 | 4.4 | 8.2 | 4.4 | 9.4 | 4.4 |
| 53 | Other disorders of the nervous system | 3.4 | 2.4 | 3.9 | 2.7 | 4.3 | 2.9 | 5.2 | 3.4 | 6.5 | 3.8 | 7.6 | 4.1 | 9.0 | 4.3 | 3.7 | 2.6 | 4.5 | 3.1 | 4.8 | 3.1 | 5.6 | 3.6 | 6.8 | 4.1 | 7.9 | 4.2 | 9.1 | 4.2 |
|  | **H – Diseases of the eye and adnexa and diseases of the ear and mastoid process** | **2.4** | **1.8** | **2.9** | **2.2** | **3.3** | **2.5** | **4.1** | **2.9** | **4.9** | **3.2** | **5.7** | **3.5** | **6.8** | **3.7** | **2.9** | **2.2** | **3.6** | **2.7** | **3.9** | **2.9** | **4.6** | **3.2** | **5.3** | **3.5** | **5.9** | **3.6** | **6.9** | **3.7** |
| 54 | Disorders of eyelid, lacrimal system and orbit | 2.0 | 1.5 | 2.8 | 2.0 | 3.0 | 2.3 | 3.9 | 3.0 | 5.0 | 3.4 | 6.3 | 3.8 | 7.8 | 3.9 | 2.7 | 1.9 | 3.6 | 2.8 | 3.6 | 2.5 | 4.2 | 3.0 | 5.3 | 3.5 | 6.2 | 3.7 | 7.7 | 3.9 |
| 55 | Corneal scars and opacities | 2.9 | 1.9 | 2.8 | 2.1 | 3.0 | 2.1 | 4.0 | 3.1 | 4.8 | 3.5 | 6.7 | 4.1 | 8.0 | 3.9 | 3.3 | 3.3 | 3.3 | 2.2 | 3.9 | 2.8 | 5.1 | 4.8 | 5.6 | 3.5 | 6.9 | 3.8 | 8.1 | 3.8 |
| 56 | Other disorders of cornea | 2.3 | 1.8 | 2.6 | 2.1 | 3.0 | 2.4 | 3.8 | 2.7 | 4.9 | 3.3 | 6.1 | 3.9 | 7.6 | 3.8 | 3.0 | 3.0 | 3.3 | 2.7 | 3.8 | 2.8 | 4.2 | 3.1 | 5.4 | 3.5 | 6.4 | 3.8 | 7.6 | 3.9 |
| 57 | Diseases of the eye lens (cataracts) | 3.1 | 2.2 | 4.1 | 3.4 | 3.9 | 3.1 | 4.6 | 3.5 | 5.3 | 3.6 | 6.1 | 3.7 | 7.0 | 3.8 | 3.9 | 2.7 | 4.7 | 3.8 | 4.9 | 3.4 | 5.3 | 3.6 | 5.8 | 3.8 | 6.2 | 3.8 | 6.9 | 3.7 |
| 58 | Disorders of the choroid and retina | 2.3 | 2.1 | 2.8 | 2.4 | 3.1 | 2.4 | 4.2 | 2.9 | 5.0 | 3.4 | 6.8 | 4.0 | 8.0 | 4.0 | 3.0 | 1.9 | 3.2 | 2.3 | 3.9 | 3.3 | 4.8 | 3.9 | 5.5 | 3.4 | 6.4 | 3.5 | 7.9 | 4.0 |
| 59 | Retinal vascular occlusions | 2.8 | 2.1 | 3.1 | 1.8 | 3.6 | 2.2 | 4.8 | 3.1 | 5.8 | 3.7 | 6.7 | 3.9 | 7.9 | 4.0 | 2.9 | 2.2 | 4.5 | 3.3 | 4.5 | 2.9 | 5.7 | 3.6 | 6.0 | 3.6 | 6.7 | 3.9 | 7.9 | 3.9 |
| 60 | Other retinal disorders | 2.8 | 2.1 | 3.2 | 2.4 | 3.5 | 2.7 | 4.1 | 3.2 | 5.2 | 3.5 | 6.1 | 3.7 | 7.3 | 3.9 | 3.4 | 2.3 | 4.0 | 3.1 | 4.4 | 3.2 | 5.1 | 3.8 | 5.6 | 3.7 | 6.3 | 3.8 | 7.3 | 3.8 |
| 61 | Retinal disorders in diseases classified elsewhere | 3.0 | 1.4 | 3.9 | 2.3 | 5.1 | 2.9 | 6.2 | 3.2 | 7.1 | 3.5 | 8.1 | 3.8 | 9.2 | 4.0 | 3.1 | 1.6 | 5.1 | 3.1 | 5.9 | 3.3 | 7.0 | 3.5 | 7.9 | 3.8 | 8.4 | 3.8 | 9.2 | 3.9 |
| 62 | Glaucoma ^c^ | 2.6 | 1.8 | 3.3 | 2.7 | 3.5 | 2.7 | 4.0 | 3.0 | 4.6 | 3.2 | 5.4 | 3.4 | 6.6 | 3.6 | 3.9 | 3.1 | 4.8 | 3.4 | 4.6 | 3.3 | 4.7 | 3.4 | 5.1 | 3.4 | 5.7 | 3.5 | 6.7 | 3.6 |
| 63 | Disorders of the vitreous body and globe | 2.3 | 1.7 | 2.7 | 2.0 | 3.9 | 3.0 | 4.7 | 3.5 | 5.2 | 3.6 | 6.6 | 4.1 | 8.2 | 4.0 | 2.5 | 2.0 | 3.6 | 2.8 | 3.8 | 2.8 | 4.4 | 3.2 | 5.3 | 3.6 | 6.2 | 3.8 | 8.3 | 3.9 |
| 64 | Disorders of optic nerve and visual pathways | 4.3 | 3.1 | 4.1 | 2.5 | 4.3 | 2.8 | 4.5 | 3.1 | 5.9 | 3.9 | 6.5 | 3.8 | 8.0 | 3.8 | 3.9 | 2.7 | 4.2 | 2.7 | 4.5 | 2.9 | 5.0 | 3.2 | 6.2 | 3.9 | 7.0 | 3.9 | 8.1 | 4.0 |
| 65 | Disorders of ocular muscles, binocular movement, accommodation and refraction | 2.3 | 1.9 | 2.4 | 2.0 | 2.8 | 2.2 | 3.5 | 2.7 | 5.0 | 3.5 | 6.3 | 3.8 | 7.7 | 4.0 | 2.7 | 2.3 | 3.0 | 2.4 | 3.3 | 2.5 | 4.0 | 3.0 | 5.5 | 3.6 | 7.0 | 4.0 | 8.3 | 4.0 |
| 66 | Visual disturbances | 3.0 | 2.3 | 3.1 | 2.5 | 3.6 | 2.6 | 4.8 | 3.3 | 5.9 | 3.7 | 7.2 | 4.1 | 8.5 | 4.1 | 3.6 | 2.7 | 4.5 | 3.2 | 4.9 | 3.6 | 5.7 | 3.9 | 6.4 | 4.1 | 7.2 | 4.1 | 8.3 | 4.1 |
| 67 | Blindness and partial sight | 4.7 | 3.0 | 4.6 | 3.4 | 4.9 | 3.3 | 6.1 | 4.1 | 6.9 | 4.2 | 8.0 | 4.3 | 9.6 | 4.4 | 4.6 | 3.4 | 5.6 | 3.6 | 5.9 | 3.8 | 6.8 | 4.4 | 7.6 | 4.7 | 8.8 | 5.0 | 1n/a | 4.4 |
| 68 | Nystagmus and other irregular eye movements and other disorders of eye and adnexa | 2.9 | 2.2 | 3.3 | 2.4 | 3.9 | 2.9 | 4.7 | 3.2 | 5.8 | 3.8 | 7.0 | 4.0 | 8.1 | 4.1 | 3.3 | 2.3 | 4.1 | 3.1 | 4.6 | 3.3 | 5.2 | 3.5 | 6.0 | 4.0 | 7.2 | 4.2 | 8.6 | 4.4 |
| 69 | Otosclerosis | 2.4 | 1.6 | 2.9 | 2.1 | 3.0 | 2.2 | 3.7 | 2.6 | 4.7 | 3.3 | 5.8 | 3.4 | 7.3 | 3.8 | 3.0 | 2.1 | 3.2 | 2.1 | 3.5 | 2.4 | 4.1 | 2.9 | 5.1 | 3.3 | 5.9 | 3.5 | 7.4 | 3.7 |
| 70 | Ménière’s disease ^c^ | 2.8 | 1.4 | 3.2 | 1.7 | 3.4 | 2.4 | 4.1 | 2.8 | 5.1 | 3.2 | 6.3 | 3.6 | 7.9 | 3.9 | 3.9 | 3.0 | 4.2 | 3.1 | 4.0 | 2.6 | 4.7 | 3.2 | 5.6 | 3.3 | 6.6 | 3.7 | 8.2 | 4.0 |
| 71 | Other diseases of the inner ear | 3.3 | 2.4 | 3.7 | 2.4 | 3.9 | 2.3 | 4.5 | 2.8 | 5.3 | 3.1 | 6.2 | 3.4 | 7.6 | 3.7 | 3.8 | 2.8 | 4.1 | 2.8 | 4.7 | 2.9 | 5.1 | 3.2 | 6.2 | 3.3 | 7.1 | 3.6 | 8.2 | 3.8 |
| 72 | Conductive and sensorineural hearing loss | 2.5 | 1.8 | 3.1 | 2.1 | 3.5 | 2.4 | 4.3 | 2.9 | 5.3 | 3.4 | 6.3 | 3.6 | 7.6 | 3.8 | 2.9 | 2.0 | 3.7 | 2.5 | 4.0 | 2.7 | 4.8 | 3.2 | 5.6 | 3.5 | 6.6 | 3.8 | 7.8 | 3.8 |
| 73 | Other hearing loss and other disorders of ear, not elsewhere classified | 3.4 | 2.3 | 4.2 | 2.7 | 4.1 | 2.9 | 4.7 | 3.1 | 5.8 | 3.8 | 6.9 | 3.8 | 8.1 | 4.0 | 3.7 | 2.5 | 4.5 | 3.3 | 4.7 | 3.0 | 5.4 | 3.5 | 6.2 | 3.5 | 6.7 | 3.6 | 8.2 | 4.0 |
| 74 | Presbycusis (age-related hearing loss) | 2.7 | 1.8 | 3.4 | 2.2 | 4.1 | 2.6 | 4.5 | 3.0 | 5.6 | 3.4 | 6.4 | 3.6 | 7.3 | 3.7 | 3.5 | 2.5 | 5.0 | 3.0 | 4.3 | 2.8 | 5.3 | 3.5 | 5.9 | 3.7 | 6.6 | 3.7 | 7.4 | 3.7 |
| 75 | Hearing loss, unspecified | 2.7 | 2.0 | 3.4 | 2.4 | 3.8 | 2.4 | 4.4 | 2.9 | 5.3 | 3.2 | 6.1 | 3.5 | 7.5 | 3.8 | 3.2 | 2.1 | 4.3 | 3.1 | 4.4 | 2.9 | 5.1 | 3.3 | 5.7 | 3.4 | 6.5 | 3.7 | 7.9 | 3.8 |
| 76 | Tinnitus | 2.8 | 2.2 | 3.1 | 2.1 | 3.5 | 2.4 | 4.3 | 2.8 | 5.4 | 3.2 | 6.5 | 3.6 | 8.0 | 3.8 | 3.5 | 2.5 | 3.6 | 2.4 | 4.2 | 2.8 | 4.9 | 3.2 | 5.7 | 3.4 | 6.7 | 3.7 | 8.2 | 3.8 |
| 77 | Other specified disorders of ear | 2.1 | 1.4 | 3.3 | 2.3 | 3.7 | 2.6 | 4.4 | 3.1 | 5.2 | 3.2 | 6.0 | 3.5 | 7.4 | 3.7 | 3.1 | 2.2 | 4.2 | 3.1 | 4.2 | 2.8 | 4.8 | 3.3 | 5.6 | 3.4 | 6.3 | 3.7 | 7.5 | 3.7 |
|  | **I – Diseases of the circulatory system** | **2.4** | **1.8** | **2.9** | **2.2** | **3.4** | **2.5** | **3.8** | **2.7** | **4.4** | **2.9** | **5.1** | **3.2** | **6.2** | **3.6** | **2.8** | **2.2** | **3.2** | **2.5** | **3.6** | **2.6** | **4.1** | **2.9** | **4.6** | **3.1** | **5.2** | **3.3** | **6.2** | **3.5** |
| 78 | Aortic and mitral valve disease ^c^ | 3.3 | 2.3 | 3.8 | 3.0 | 4.6 | 3.4 | 5.6 | 3.5 | 6.7 | 3.8 | 7.8 | 4.0 | 8.9 | 4.0 | 2.9 | 1.9 | 4.3 | 3.0 | 5.5 | 4.1 | 6.4 | 4.0 | 7.2 | 4.2 | 8.1 | 4.3 | 9.0 | 4.1 |
| 79 | Hypertensive diseases ^c^ | 2.9 | 2.1 | 3.4 | 2.5 | 3.7 | 2.7 | 4.0 | 2.7 | 4.5 | 2.9 | 5.2 | 3.2 | 6.3 | 3.6 | 3.1 | 2.3 | 3.5 | 2.6 | 3.9 | 2.7 | 4.2 | 2.9 | 4.7 | 3.1 | 5.2 | 3.3 | 6.2 | 3.6 |
| 80 | Heart failure ^c^ | 4.3 | 2.6 | 4.9 | 2.9 | 6.1 | 3.3 | 6.9 | 3.4 | 7.7 | 3.7 | 8.7 | 4.1 | 9.7 | 4.1 | 4.5 | 2.3 | 5.8 | 3.5 | 6.6 | 4.1 | 7.5 | 4.2 | 8.4 | 4.3 | 9.2 | 4.4 | 9.8 | 4.2 |
| 80A | Ischaemic heart diseases | 2.7 | 2.1 | 4.2 | 3.2 | 5.1 | 3.3 | 5.9 | 3.3 | 6.8 | 3.4 | 7.8 | 3.7 | 9.1 | 3.9 | 4.6 | 3.1 | 5.3 | 4.3 | 5.9 | 3.9 | 6.6 | 3.8 | 7.4 | 4.0 | 8.3 | 4.1 | 9.5 | 4.0 |
| 81 | Angina pectoris | 2.3 | 1.9 | 4.0 | 3.2 | 5.1 | 3.4 | 6.0 | 3.4 | 6.9 | 3.5 | 7.9 | 3.8 | 9.4 | 4.0 | 4.8 | 3.2 | 4.9 | 4.1 | 5.9 | 4.0 | 6.5 | 3.8 | 7.3 | 4.0 | 8.2 | 4.1 | 9.6 | 4.1 |
| 82 | Acute myocardial infarction and subsequent myocardial infarction | 3.7 | 2.5 | 4.7 | 2.9 | 5.7 | 3.1 | 6.2 | 3.0 | 6.9 | 3.3 | 7.9 | 3.8 | 9.3 | 4.0 | 4.3 | 3.8 | 6.2 | 4.2 | 6.9 | 4.0 | 7.2 | 3.7 | 8.0 | 4.0 | 8.9 | 4.2 | 9.8 | 4.1 |
| 83 | AMI complex/other | 2.7 | 1.4 | 5.2 | 3.1 | 5.8 | 4.1 | 7.3 | 4.0 | 8.2 | 3.9 | 9.9 | 4.5 | 10.6 | 4.2 | 4.4 | 3.0 | 4.1 | 2.5 | 6.2 | 4.0 | 7.3 | 4.6 | 9.1 | 4.3 | 10.5 | 4.6 | 11.1 | 4.3 |
| 84 | Chronic ischaemic heart disease | 3.3 | 2.0 | 5.3 | 3.7 | 6.3 | 3.3 | 6.8 | 3.3 | 7.5 | 3.4 | 8.4 | 3.8 | 9.7 | 3.9 | 4.6 | 2.3 | 6.9 | 4.9 | 7.2 | 4.1 | 7.8 | 4.0 | 8.5 | 4.1 | 9.3 | 4.2 | 10.1 | 4.1 |
| 85 | Pulmonary heart disease and diseases of pulmonary circulation | 3.8 | 2.5 | 4.5 | 3.2 | 4.8 | 3.6 | 5.8 | 3.7 | 6.7 | 3.9 | 7.8 | 4.4 | 9.0 | 4.4 | 3.4 | 2.5 | 4.6 | 3.3 | 5.1 | 3.7 | 6.2 | 3.8 | 8.1 | 4.5 | 8.5 | 4.4 | 9.3 | 4.4 |
| 86 | Acute pericarditis | 2.3 | 1.6 | 2.8 | 2.3 | 3.4 | 2.9 | 4.2 | 3.3 | 5.4 | 3.7 | 6.9 | 4.3 | 8.6 | 4.1 | 3.5 | 2.7 | 3.9 | 3.2 | 4.6 | 4.2 | 5.6 | 3.8 | 6.6 | 4.1 | 7.3 | 4.2 | 9.2 | 4.5 |
| 87 | Other forms of heart disease | 3.0 | 2.2 | 3.6 | 2.8 | 4.9 | 3.5 | 6.3 | 3.8 | 7.7 | 4.2 | 9.1 | 4.5 | 10.2 | 4.2 | 4.2 | 3.2 | 5.5 | 3.4 | 6.1 | 4.2 | 7.1 | 4.2 | 8.2 | 4.6 | 9.2 | 4.4 | 10.4 | 4.5 |
| 88 | Atrioventricular and left bundle branch block | 2.9 | 1.9 | 4.0 | 3.2 | 4.4 | 3.3 | 5.2 | 3.7 | 6.9 | 4.0 | 7.9 | 4.3 | 8.7 | 4.0 | 3.4 | 2.4 | 4.8 | 3.8 | 4.9 | 4.0 | 5.5 | 3.6 | 6.7 | 4.0 | 7.9 | 4.3 | 8.8 | 4.1 |
| 89 | Other conduction disorders | 3.2 | 2.3 | 3.6 | 2.7 | 4.4 | 3.1 | 5.9 | 3.5 | 7.2 | 4.0 | 8.2 | 4.1 | 9.6 | 4.3 | 3.3 | 2.2 | 4.6 | 3.4 | 5.1 | 3.6 | 6.0 | 3.8 | 7.6 | 4.4 | 9.0 | 4.7 | 9.6 | 4.2 |
| 90 | Paroxysmal tachycardia | 2.6 | 1.9 | 3.4 | 2.6 | 4.2 | 3.0 | 5.2 | 3.4 | 6.4 | 3.7 | 7.7 | 4.0 | 9.2 | 4.1 | 3.2 | 2.5 | 3.8 | 2.8 | 4.5 | 3.1 | 5.1 | 3.4 | 6.2 | 3.7 | 7.4 | 4.0 | 9.1 | 4.1 |
| 91 | Atrial fibrillation and flutter | 3.1 | 2.1 | 3.4 | 2.6 | 4.0 | 2.8 | 4.8 | 3.1 | 6.0 | 3.5 | 7.0 | 3.8 | 8.2 | 3.9 | 3.6 | 2.9 | 5.0 | 3.6 | 5.3 | 3.7 | 5.8 | 3.6 | 6.6 | 3.9 | 7.5 | 4.0 | 8.4 | 3.9 |
| 92 | Other cardiac arrhythmias | 2.9 | 1.9 | 3.3 | 2.7 | 3.9 | 2.9 | 4.9 | 3.1 | 6.5 | 3.8 | 7.7 | 4.0 | 9.0 | 4.1 | 3.4 | 2.5 | 4.0 | 3.1 | 4.2 | 3.0 | 4.9 | 3.4 | 6.0 | 3.7 | 7.5 | 4.1 | 9.1 | 4.1 |
| 93 | Complications and ill-defined descriptions of heart disease and other heart disorders in diseases classified elsewhere | 3.6 | 2.5 | 3.7 | 2.5 | 5.0 | 3.3 | 6.6 | 3.8 | 7.8 | 4.2 | 9.1 | 4.4 | 10.1 | 4.3 | 4.0 | 2.7 | 4.4 | 2.6 | 5.5 | 3.9 | 6.7 | 3.9 | 8.3 | 4.9 | 8.9 | 4.4 | 10.2 | 4.3 |
| 94 | Stroke | 3.8 | 2.5 | 4.6 | 3.3 | 5.1 | 3.1 | 6.0 | 3.5 | 6.6 | 3.6 | 7.4 | 3.7 | 8.2 | 3.8 | 5.3 | 3.6 | 5.3 | 3.5 | 5.7 | 3.5 | 6.7 | 3.7 | 7.2 | 3.9 | 7.9 | 4.0 | 8.6 | 3.8 |
| 95 | Cerebrovascular diseases | 3.5 | 2.7 | 4.1 | 3.2 | 4.6 | 3.2 | 5.9 | 3.6 | 7.1 | 3.7 | 8.2 | 4.0 | 9.3 | 4.1 | 4.1 | 2.9 | 4.9 | 3.4 | 5.3 | 3.6 | 6.1 | 3.6 | 7.2 | 4.0 | 8.6 | 4.2 | 9.5 | 4.1 |
| 96 | Sequelae of cerebrovascular disease | 4.6 | 2.7 | 5.8 | 3.6 | 6.3 | 3.3 | 7.4 | 3.8 | 7.8 | 3.8 | 8.6 | 3.9 | 9.3 | 3.9 | 5.9 | 3.3 | 6.5 | 3.5 | 7.0 | 3.9 | 7.9 | 4.0 | 8.5 | 4.2 | 9.1 | 4.2 | 9.5 | 4.0 |
| 97 | Atherosclerosis | 3.7 | 3.1 | 4.0 | 2.5 | 6.3 | 4.4 | 6.8 | 4.2 | 7.5 | 4.0 | 8.7 | 4.3 | 9.6 | 4.3 | 4.0 | 3.5 | 5.4 | 4.4 | 6.1 | 4.0 | 7.2 | 4.4 | 8.2 | 4.4 | 8.8 | 4.4 | 9.4 | 4.2 |
| 98 | Aortic aneurysm and aortic dissection | 4.0 | 2.0 | 4.4 | 2.0 | 4.6 | 3.1 | 5.4 | 3.2 | 6.6 | 3.6 | 7.7 | 3.9 | 8.7 | 4.0 | 4.5 | 3.5 | 3.8 | 2.5 | 5.2 | 3.6 | 5.2 | 3.3 | 6.8 | 3.8 | 8.1 | 4.0 | 9.2 | 4.0 |
| 99 | Diseases of arteries, arterioles and capillaries | 2.7 | 1.9 | 3.4 | 2.7 | 5.0 | 4.0 | 5.5 | 3.7 | 6.9 | 3.9 | 8.5 | 4.4 | 9.8 | 4.4 | 3.0 | 2.7 | 3.8 | 3.3 | 4.1 | 3.3 | 5.0 | 3.7 | 6.6 | 4.2 | 8.0 | 4.3 | 1n/a | 4.4 |
| 100 | Other peripheral vascular diseases | 2.5 | 1.7 | 3.2 | 2.2 | 5.0 | 3.5 | 5.9 | 3.7 | 6.8 | 3.8 | 7.9 | 4.1 | 9.1 | 4.3 | 3.0 | 2.2 | 3.8 | 2.7 | 4.9 | 3.5 | 6.2 | 3.8 | 7.5 | 4.2 | 8.1 | 4.1 | 9.1 | 4.1 |
| 101 | Phlebitis, thrombosis of the portal vein and others | 3.1 | 2.2 | 3.6 | 2.6 | 3.9 | 3.2 | 4.6 | 3.3 | 5.5 | 3.7 | 6.4 | 4.0 | 7.9 | 4.3 | 3.2 | 2.5 | 4.2 | 3.3 | 4.6 | 3.5 | 5.3 | 3.8 | 6.6 | 4.2 | 7.3 | 4.2 | 8.3 | 4.2 |
| 102 | Varicose veins of lower extremities | 2.1 | 1.7 | 2.1 | 1.7 | 2.7 | 2.2 | 3.4 | 2.7 | 4.4 | 3.2 | 5.7 | 3.8 | 7.5 | 4.3 | 2.2 | 1.7 | 2.6 | 1.8 | 2.8 | 2.1 | 3.4 | 2.5 | 4.5 | 3.1 | 5.4 | 3.4 | 7.6 | 4.1 |
| 103 | Haemorrhoids ^c^ | 2.0 | 1.6 | 2.4 | 1.9 | 2.8 | 2.2 | 3.5 | 2.6 | 4.7 | 3.2 | 5.9 | 3.6 | 7.6 | 4.1 | 2.4 | 1.9 | 2.7 | 2.0 | 3.2 | 2.4 | 4.1 | 2.8 | 5.1 | 3.3 | 6.3 | 3.8 | 8.2 | 4.1 |
| 104 | Oesophageal varices (chronic), varicose veins of other sites, other disorders of veins, non-specific lymphadenitis, other non-infective disorders of lymphatic vessels and lymph nodes and other and unspecified disorders of the circulatory system | 2.1 | 1.5 | 2.5 | 2.0 | 3.6 | 3.1 | 5.3 | 3.7 | 6.5 | 3.9 | 7.8 | 4.4 | 9.2 | 4.3 | 3.0 | 2.3 | 4.0 | 3.0 | 4.3 | 3.3 | 5.5 | 3.9 | 7.0 | 4.1 | 7.8 | 4.3 | 9.4 | 4.4 |
|  | **J – Diseases of the respiratory system** | **1.8** | **1.3** | **2.2** | **1.7** | **2.6** | **2.0** | **3.3** | **2.5** | **4.5** | **3.1** | **5.7** | **3.5** | **7.2** | **3.8** | **2.2** | **1.7** | **2.7** | **2.1** | **3.1** | **2.3** | **3.7** | **2.7** | **4.7** | **3.1** | **5.7** | **3.5** | **7.3** | **3.7** |
| 105 | Respiratory allergy ^c^ | 1.8 | 1.3 | 2.2 | 1.6 | 2.5 | 2.0 | 3.2 | 2.5 | 4.3 | 3.1 | 5.6 | 3.5 | 7.2 | 3.8 | 2.1 | 1.6 | 2.7 | 2.1 | 3.0 | 2.3 | 3.7 | 2.7 | 4.7 | 3.1 | 5.6 | 3.5 | 7.2 | 3.8 |
| 105A | Chronic lower respiratory diseases ^c^ | 2.9 | 1.5 | 3.0 | 1.8 | 3.5 | 2.2 | 4.2 | 2.7 | 5.5 | 3.3 | 6.9 | 3.7 | 8.3 | 3.9 | 3.3 | 1.9 | 3.6 | 2.3 | 4.0 | 2.6 | 4.5 | 2.9 | 5.8 | 3.4 | 7.0 | 3.7 | 8.4 | 3.8 |
| 106 | Bronchitis, not specified as acute or chronic, simple and  mucopurulent chronic bronchitis and unspecified chronic bronchitis | 4.0 | 1.5 | 4.6 | 2.5 | 5.6 | 3.5 | 7.4 | 4.4 | 8.6 | 4.4 | 1n/a | 4.8 | 11.0 | 4.3 | 5.2 | 3.4 | 6.2 | 3.6 | 7.2 | 5.0 | 8.3 | 4.5 | 9.1 | 4.3 | 10.3 | 4.5 | 11.5 | 4.2 |
| 107 | Emphysema | 3.8 | 1.9 | 4.0 | 2.1 | 5.4 | 3.3 | 6.6 | 3.5 | 7.4 | 3.5 | 8.9 | 4.1 | 10.1 | 4.1 | 4.6 | 3.1 | 5.1 | 2.5 | 6.6 | 3.4 | 7.0 | 3.7 | 8.5 | 4.1 | 9.6 | 4.3 | 10.4 | 4.0 |
| 108 | Chronic obstructive lung disease (COPD) ^c^ | 2.7 | 1.5 | 3.4 | 1.9 | 4.1 | 2.6 | 4.9 | 3.2 | 6.0 | 3.6 | 7.1 | 3.9 | 8.4 | 4.0 | 3.1 | 1.9 | 4.1 | 2.6 | 4.8 | 3.1 | 5.6 | 3.4 | 6.5 | 3.6 | 7.3 | 3.8 | 8.6 | 3.9 |
| 109 | Asthma, status asthmaticus ^c^ | 2.3 | 1.4 | 3.2 | 1.8 | 3.6 | 2.2 | 4.4 | 2.7 | 5.7 | 3.3 | 7.0 | 3.7 | 8.5 | 3.9 | 2.7 | 1.8 | 3.8 | 2.4 | 4.3 | 2.6 | 5.1 | 3.0 | 6.3 | 3.4 | 7.3 | 3.6 | 8.7 | 3.8 |
| 110 | Bronchiectasis | 4.4 | 2.5 | 4.9 | 2.3 | 5.6 | 2.7 | 6.4 | 3.3 | 7.1 | 3.6 | 8.2 | 4.2 | 10.3 | 4.5 | 4.9 | 2.2 | 5.1 | 2.2 | 5.9 | 3.2 | 6.8 | 3.8 | 7.1 | 4.1 | 7.6 | 3.8 | 9.1 | 4.0 |
| 111 | Other diseases of the respiratory system | 3.5 | 2.4 | 4.0 | 2.9 | 4.8 | 3.4 | 6.1 | 3.8 | 7.4 | 4.4 | 8.5 | 4.4 | 9.8 | 4.4 | 4.3 | 3.1 | 5.0 | 3.6 | 6.0 | 4.2 | 6.7 | 4.1 | 7.9 | 4.4 | 8.7 | 4.5 | 9.9 | 4.3 |
|  | **K – Diseases of the digestive system** | **2.3** | **1.8** | **2.8** | **2.2** | **3.4** | **2.7** | **4.3** | **3.2** | **5.4** | **3.6** | **6.5** | **3.9** | **8.1** | **4.1** | **2.8** | **2.2** | **3.6** | **2.7** | **4.2** | **3.0** | **5.0** | **3.4** | **6.1** | **3.8** | **7.1** | **4.0** | **8.4** | **4.0** |
| 112 | Ulcers ^c^ | 2.3 | 1.9 | 3.0 | 2.4 | 3.8 | 2.9 | 4.8 | 3.4 | 6.0 | 3.8 | 7.1 | 4.1 | 8.5 | 4.2 | 2.7 | 2.3 | 3.8 | 3.1 | 4.5 | 3.3 | 5.4 | 3.6 | 6.5 | 3.9 | 7.4 | 4.1 | 8.5 | 4.0 |
| 113 | Inguinal hernia | 1.8 | 1.3 | 2.2 | 1.6 | 2.4 | 1.8 | 3.0 | 2.4 | 3.8 | 2.8 | 4.8 | 3.3 | 6.6 | 3.7 | 2.2 | 1.8 | 2.7 | 2.3 | 3.4 | 2.5 | 3.7 | 2.8 | 5.4 | 3.6 | 6.0 | 3.6 | 7.9 | 4.1 |
| 114 | Ventral hernia | 2.1 | 1.8 | 3.3 | 2.6 | 4.2 | 3.4 | 4.8 | 3.5 | 6.3 | 3.9 | 7.4 | 4.1 | 8.7 | 4.2 | 2.8 | 2.0 | 4.0 | 3.3 | 4.3 | 3.4 | 5.6 | 3.8 | 6.9 | 4.1 | 8.1 | 4.3 | 9.0 | 4.2 |
| 115 | Crohn’s diease | 2.4 | 1.6 | 3.0 | 2.1 | 3.5 | 2.6 | 4.3 | 3.1 | 5.5 | 3.7 | 6.8 | 4.0 | 8.4 | 4.5 | 3.2 | 2.3 | 3.9 | 2.7 | 4.2 | 2.9 | 5.0 | 3.5 | 6.0 | 3.8 | 7.5 | 4.4 | 8.6 | 4.2 |
| 116 | Ulcerative colitis | 2.4 | 1.6 | 2.8 | 2.0 | 3.2 | 2.4 | 4.0 | 2.9 | 5.0 | 3.4 | 6.5 | 3.9 | 8.2 | 4.3 | 2.9 | 2.1 | 3.6 | 2.7 | 3.8 | 2.8 | 4.4 | 3.2 | 5.8 | 3.7 | 7.2 | 4.2 | 8.7 | 4.4 |
| 117 | Other non-infective gastroenteritis and colitis | 2.7 | 1.8 | 3.5 | 2.6 | 4.2 | 3.0 | 5.5 | 3.7 | 6.8 | 4.2 | 7.9 | 4.4 | 9.5 | 4.4 | 3.4 | 2.5 | 4.2 | 2.9 | 5.2 | 3.5 | 6.2 | 4.1 | 7.2 | 4.3 | 8.2 | 4.4 | 9.5 | 4.3 |
| 118 | Irritable bowel syndrome (IBS) | 2.3 | 1.6 | 3.0 | 2.2 | 3.4 | 2.6 | 4.1 | 3.0 | 5.4 | 3.6 | 6.6 | 4.0 | 8.7 | 4.5 | 2.8 | 2.1 | 3.6 | 2.6 | 4.3 | 3.1 | 5.1 | 3.4 | 6.1 | 3.8 | 7.3 | 4.2 | 8.9 | 4.4 |
| 119 | Other functional intestinal disorders | 2.7 | 2.2 | 3.5 | 2.6 | 4.4 | 3.3 | 5.5 | 3.9 | 6.8 | 4.2 | 8.1 | 4.5 | 9.3 | 4.4 | 3.2 | 2.5 | 4.2 | 3.2 | 5.0 | 3.7 | 6.1 | 4.1 | 7.1 | 4.5 | 8.1 | 4.6 | 9.3 | 4.3 |
| 120 | Diseases of liver, biliary tract and pancreas | 2.8 | 2.2 | 3.8 | 3.0 | 4.6 | 3.3 | 5.7 | 3.6 | 6.7 | 3.9 | 7.5 | 4.2 | 8.4 | 4.3 | 3.7 | 3.1 | 4.3 | 3.3 | 5.0 | 3.7 | 6.0 | 3.9 | 6.9 | 4.1 | 7.6 | 4.2 | 8.7 | 4.3 |
|  | **L – Diseases of the skin and subcutaneous tissue** | **1.8** | **1.4** | **2.3** | **1.8** | **2.6** | **2.1** | **3.4** | **2.6** | **4.5** | **3.2** | **5.7** | **3.6** | **7.5** | **4.0** | **2.2** | **1.7** | **3.0** | **2.3** | **3.3** | **2.6** | **4.1** | **3.0** | **5.0** | **3.4** | **6.0** | **3.7** | **7.6** | **4.0** |
| 121 | Psoriasis ^c^ | 1.8 | 1.4 | 2.3 | 1.8 | 2.6 | 2.1 | 3.4 | 2.6 | 4.5 | 3.2 | 5.7 | 3.6 | 7.5 | 4.0 | 2.2 | 1.7 | 3.0 | 2.3 | 3.3 | 2.6 | 4.1 | 3.0 | 5.0 | 3.4 | 6.0 | 3.7 | 7.6 | 4.0 |
|  | **M – Diseases of the musculoskeletal system and connective tissue** | **2.0** | **1.4** | **2.4** | **1.8** | **2.8** | **2.1** | **3.5** | **2.6** | **4.4** | **3.1** | **5.6** | **3.5** | **7.1** | **3.8** | **2.3** | **1.8** | **3.2** | **2.4** | **3.6** | **2.5** | **4.1** | **2.8** | **4.7** | **3.1** | **5.5** | **3.4** | **6.9** | **3.6** |
| 122 | Infectious arthropathies | 2.3 | 1.4 | 3.0 | 2.0 | 3.3 | 2.3 | 4.3 | 3.1 | 5.6 | 3.4 | 7.3 | 4.4 | 8.6 | 4.2 | 3.1 | 2.2 | 3.8 | 2.9 | 4.2 | 2.8 | 4.9 | 3.2 | 6.0 | 3.7 | 7.2 | 4.4 | 8.5 | 4.1 |
| 122A | Inflammatory polyarthropathies and ankylosing spondylitis ^c^ | 2.6 | 1.7 | 3.1 | 2.1 | 3.3 | 2.5 | 4.1 | 2.9 | 5.2 | 3.4 | 6.4 | 3.8 | 8.1 | 4.1 | 3.1 | 2.2 | 3.9 | 2.7 | 4.2 | 2.9 | 5.1 | 3.3 | 6.1 | 3.7 | 7.2 | 4.0 | 8.5 | 4.1 |
| 123 | Rheumatoid arthritis ^c^ | 2.6 | 1.7 | 3.3 | 1.9 | 3.3 | 2.3 | 4.3 | 2.9 | 5.3 | 3.4 | 6.6 | 3.9 | 8.2 | 4.2 | 3.0 | 2.1 | 3.9 | 2.6 | 4.1 | 2.8 | 5.0 | 3.2 | 6.1 | 3.6 | 7.1 | 4.0 | 8.5 | 4.1 |
| 124 | Inflammatory polyarthropathies  – except rheumatoid arthritis ^c^ | 2.9 | 1.7 | 3.3 | 2.1 | 3.6 | 2.6 | 4.3 | 2.9 | 5.4 | 3.4 | 6.6 | 3.8 | 8.3 | 4.1 | 3.5 | 2.3 | 4.2 | 2.8 | 4.8 | 3.0 | 5.5 | 3.4 | 6.6 | 3.7 | 7.6 | 4.1 | 8.8 | 4.2 |
| 125 | Polyarthrosis [arthrosis] | 2.5 | 1.4 | 3.6 | 2.2 | 4.7 | 3.4 | 5.5 | 3.5 | 6.3 | 3.8 | 7.7 | 4.2 | 9.4 | 4.5 | 4.0 | 2.9 | 5.1 | 3.5 | 5.2 | 3.4 | 5.7 | 3.5 | 6.6 | 3.8 | 7.6 | 4.1 | 9.6 | 4.4 |
| 126 | Coxarthrosis [arthrosis of hip] | 2.6 | 1.9 | 3.1 | 2.2 | 3.4 | 2.6 | 3.9 | 3.0 | 4.8 | 3.3 | 5.8 | 3.7 | 7.1 | 3.9 | 2.9 | 1.8 | 3.9 | 2.8 | 4.2 | 3.0 | 4.7 | 3.2 | 5.5 | 3.6 | 6.2 | 3.8 | 7.2 | 3.8 |
| 127 | Gonarthrosis [arthrosis of knee] | 2.4 | 1.8 | 2.7 | 2.1 | 3.0 | 2.2 | 3.6 | 2.6 | 4.5 | 3.1 | 5.7 | 3.5 | 7.3 | 3.9 | 2.9 | 1.8 | 3.9 | 2.7 | 4.0 | 2.9 | 4.5 | 3.1 | 5.3 | 3.4 | 6.1 | 3.6 | 7.5 | 3.9 |
| 128 | Arthrosis of first carpometacarpal joint and other arthrosis | 2.3 | 1.6 | 2.7 | 2.0 | 3.3 | 2.4 | 4.1 | 3.0 | 5.1 | 3.4 | 6.4 | 3.9 | 8.2 | 4.3 | 3.2 | 2.2 | 4.0 | 2.7 | 4.4 | 2.9 | 5.0 | 3.3 | 5.8 | 3.6 | 6.9 | 4.0 | 8.8 | 4.3 |
| 129 | Acquired deformities of fingers and toes | 2.2 | 1.8 | 2.7 | 2.2 | 3.1 | 2.4 | 3.9 | 2.9 | 4.9 | 3.3 | 6.3 | 3.8 | 8.0 | 4.2 | 2.3 | 1.8 | 2.9 | 2.2 | 3.3 | 2.4 | 4.0 | 2.8 | 5.0 | 3.3 | 6.0 | 3.6 | 7.8 | 4.0 |
| 130 | Other acquired deformities of limbs | 2.3 | 1.6 | 3.0 | 2.1 | 3.4 | 2.4 | 4.2 | 3.1 | 5.4 | 3.4 | 6.8 | 3.9 | 8.4 | 4.0 | 2.7 | 2.0 | 3.6 | 2.6 | 4.2 | 2.9 | 4.8 | 3.3 | 5.8 | 3.6 | 7.1 | 4.1 | 8.5 | 4.0 |
| 131 | Disorders of patella (knee cap) | 2.0 | 1.6 | 2.4 | 1.8 | 2.8 | 2.1 | 3.5 | 2.6 | 4.4 | 2.9 | 5.7 | 3.7 | 7.1 | 3.6 | 2.4 | 1.8 | 3.2 | 2.4 | 3.7 | 2.7 | 4.3 | 3.0 | 5.3 | 3.5 | 6.2 | 3.7 | 8.0 | 4.0 |
| 132 | Internal derangement of knee | 2.1 | 1.3 | 2.6 | 1.7 | 3.2 | 2.0 | 3.9 | 2.7 | 4.9 | 3.0 | 6.4 | 3.7 | 8.1 | 4.3 | 2.4 | 1.7 | 3.3 | 2.4 | 3.9 | 2.5 | 4.3 | 2.8 | 5.5 | 3.3 | 6.9 | 3.7 | 7.7 | 3.5 |
| 133 | Derangement of meniscus due to old tear or injury | 2.1 | 1.4 | 2.6 | 1.7 | 2.9 | 1.9 | 3.5 | 2.4 | 4.5 | 2.8 | 5.6 | 3.2 | 7.5 | 3.8 | 2.5 | 1.8 | 3.4 | 2.4 | 3.7 | 2.4 | 4.3 | 2.9 | 5.3 | 3.1 | 6.4 | 3.5 | 7.5 | 3.8 |
| 134 | Internal derangement of knee, unspecified | 2.0 | 1.3 | 2.5 | 1.8 | 3.0 | 2.1 | 3.5 | 2.4 | 4.6 | 3.0 | 5.8 | 3.4 | 7.9 | 4.4 | 2.5 | 1.8 | 3.5 | 2.5 | 3.8 | 2.7 | 4.3 | 2.9 | 5.4 | 3.4 | 6.4 | 3.7 | 7.8 | 4.2 |
| 135 | Other specific joint derangements | 1.9 | 1.2 | 2.5 | 2.0 | 3.1 | 2.3 | 3.8 | 2.5 | 5.1 | 3.3 | 6.6 | 3.5 | 8.2 | 3.8 | 2.6 | 1.9 | 3.8 | 2.7 | 4.1 | 2.7 | 4.8 | 3.3 | 5.8 | 3.4 | 7.3 | 4.0 | 8.4 | 3.8 |
| 136 | Other joint disorders, not elsewhere classified | 2.4 | 1.5 | 3.1 | 2.2 | 3.6 | 2.4 | 4.6 | 3.0 | 5.8 | 3.6 | 7.0 | 4.0 | 9.1 | 4.5 | 2.9 | 2.0 | 4.3 | 3.0 | 4.7 | 3.0 | 5.3 | 3.2 | 6.3 | 3.8 | 7.7 | 4.2 | 9.3 | 4.2 |
| 137 | Systemic connective tissue disorders | 3.0 | 2.1 | 3.4 | 2.5 | 4.3 | 2.9 | 5.3 | 3.4 | 6.6 | 4.3 | 7.1 | 4.2 | 8.4 | 4.3 | 3.3 | 2.4 | 4.3 | 2.9 | 4.8 | 3.1 | 5.7 | 3.6 | 6.9 | 4.0 | 7.7 | 4.2 | 8.6 | 4.2 |
| 138 | Systemic lupus erythematosus | 4.5 | 2.7 | 5.4 | 2.9 | 7.2 | 5.1 | 6.6 | 3.8 | 8.0 | 3.6 | 8.7 | 4.5 | 9.9 | 4.7 | 4.7 | 2.7 | 6.3 | 3.6 | 6.2 | 3.4 | 6.9 | 4.0 | 8.0 | 4.4 | 8.9 | 4.4 | 10.2 | 4.7 |
| 139 | Dermatopolymyositis | 2.2 | 2.2 | 4.1 | 2.7 | 4.7 | 3.2 | 5.3 | 3.7 | 6.8 | 4.0 | 7.7 | 4.0 | 9.0 | 4.0 | 3.4 | 2.5 | 5.2 | 3.9 | 5.5 | 3.7 | 5.9 | 3.3 | 7.3 | 4.1 | 8.6 | 4.7 | 9.8 | 4.3 |
| 140 | Systemic sclerosis | 2.8 | 3.2 | 4.2 | 3.1 | 5.3 | 3.1 | 7.4 | 5.3 | 7.7 | 5.1 | 9.1 | 4.5 | 10.5 | 4.8 | 3.3 | 3.3 | 5.7 | 4.1 | 5.2 | 3.3 | 6.8 | 3.7 | 8.1 | 4.5 | 8.7 | 4.1 | 1n/a | 4.5 |
| 141 | Kyphosis, lordosis | 2.9 | 2.2 | 2.9 | 1.7 | 3.4 | 2.7 | 4.3 | 3.0 | 5.4 | 3.7 | 7.2 | 4.4 | 9.6 | 5.2 | 3.6 | 2.9 | 3.9 | 2.2 | 4.1 | 2.7 | 4.7 | 3.1 | 6.4 | 4.1 | 7.8 | 4.2 | 1n/a | 5.1 |
| 142 | Scoliosis | 2.4 | 1.9 | 2.9 | 2.1 | 3.5 | 2.4 | 4.2 | 2.8 | 5.9 | 3.8 | 7.5 | 4.1 | 9.5 | 4.2 | 2.2 | 1.7 | 3.0 | 2.3 | 3.7 | 2.6 | 4.7 | 3.2 | 6.3 | 3.8 | 8.1 | 4.5 | 9.5 | 4.3 |
| 143 | Spinal osteochondrosis | 2.5 | 1.8 | 3.0 | 2.2 | 3.5 | 2.6 | 4.2 | 2.8 | 5.7 | 3.7 | 7.1 | 4.3 | 8.4 | 4.6 | 2.8 | 2.1 | 4.0 | 3.2 | 4.2 | 2.8 | 5.2 | 3.5 | 6.7 | 4.3 | 8.1 | 4.7 | 9.7 | 4.2 |
| 144 | Other deforming dorsopathies | 2.2 | 1.5 | 2.8 | 2.1 | 3.3 | 2.3 | 4.1 | 2.8 | 5.5 | 3.6 | 7.3 | 4.2 | 9.2 | 4.4 | 2.7 | 1.9 | 3.9 | 2.7 | 4.1 | 2.8 | 5.0 | 3.2 | 6.5 | 3.9 | 8.1 | 4.2 | 9.5 | 4.3 |
| 145 | Other inflammatory spondylopathies | 3.2 | 2.2 | 3.2 | 1.9 | 4.1 | 2.8 | 5.1 | 3.4 | 6.1 | 3.9 | 7.9 | 4.7 | 8.8 | 4.2 | 3.2 | 1.9 | 4.4 | 2.7 | 4.9 | 3.1 | 5.5 | 3.2 | 7.0 | 4.1 | 8.2 | 4.6 | 9.6 | 4.4 |
| 146 | Spondylosis | 2.7 | 1.8 | 3.6 | 2.4 | 3.9 | 2.6 | 4.5 | 3.0 | 5.7 | 3.6 | 7.3 | 4.2 | 9.0 | 4.3 | 3.6 | 3.0 | 4.7 | 3.1 | 4.7 | 2.9 | 5.4 | 3.3 | 6.5 | 3.9 | 7.9 | 4.2 | 9.4 | 4.2 |
| 147 | Other spondylopathies and spondylopathies in diseases classified elsewhere | 3.6 | 2.7 | 3.8 | 2.5 | 4.2 | 2.8 | 5.1 | 3.3 | 6.2 | 3.7 | 7.6 | 4.1 | 8.9 | 4.3 | 3.6 | 2.1 | 4.4 | 3.0 | 5.0 | 3.2 | 6.0 | 3.6 | 7.2 | 4.0 | 8.3 | 4.3 | 9.2 | 4.2 |
| 148 | Cervical disc disorders | 2.8 | 1.6 | 3.3 | 2.3 | 3.6 | 2.3 | 4.4 | 2.9 | 5.8 | 3.7 | 7.5 | 4.3 | 9.8 | 4.8 | 3.7 | 2.7 | 4.2 | 2.6 | 4.3 | 2.7 | 5.1 | 3.3 | 6.5 | 3.8 | 8.0 | 4.7 | 9.9 | 4.5 |
| 149 | Other intervertebral disc disorders | 2.3 | 1.7 | 2.8 | 1.9 | 3.3 | 2.4 | 4.1 | 2.8 | 5.5 | 3.5 | 7.5 | 4.2 | 9.2 | 4.4 | 3.1 | 2.4 | 3.9 | 2.8 | 4.1 | 2.7 | 5.1 | 3.3 | 6.6 | 4.0 | 8.2 | 4.2 | 9.6 | 4.3 |
| 150 | Other dorsopathies, not elsewhere classified | 2.5 | 1.9 | 3.2 | 2.2 | 3.8 | 2.8 | 4.5 | 3.1 | 5.9 | 3.6 | 8.2 | 4.6 | 9.7 | 4.5 | 2.7 | 1.8 | 4.1 | 2.7 | 4.5 | 2.9 | 5.4 | 3.3 | 6.9 | 4.1 | 8.5 | 4.5 | 9.7 | 4.7 |
| 151 | Dorsalgia | 2.3 | 1.7 | 3.0 | 2.1 | 3.6 | 2.5 | 4.5 | 3.0 | 5.9 | 3.8 | 8.0 | 4.5 | 9.8 | 4.5 | 2.9 | 2.2 | 3.9 | 2.8 | 4.4 | 2.9 | 5.3 | 3.4 | 6.8 | 4.1 | 8.4 | 4.5 | 9.7 | 4.5 |
| 152 | Soft tissue disorders | 2.4 | 1.8 | 3.2 | 2.4 | 4.0 | 3.0 | 5.1 | 3.6 | 6.5 | 4.1 | 8.0 | 4.5 | 9.0 | 4.3 | 2.9 | 2.3 | 3.8 | 2.7 | 4.4 | 3.0 | 5.3 | 3.5 | 6.6 | 4.3 | 8.3 | 4.5 | 9.7 | 4.8 |
| 153 | Synovitis and tenosynovitis | 2.3 | 1.4 | 2.8 | 1.9 | 3.3 | 2.3 | 4.1 | 2.7 | 4.9 | 3.2 | 6.3 | 3.7 | 7.5 | 4.0 | 2.7 | 1.8 | 3.5 | 2.5 | 4.2 | 2.8 | 4.7 | 3.1 | 5.4 | 3.4 | 6.5 | 3.8 | 8.1 | 4.1 |
| 154 | Disorders of synovium and tendon | 2.2 | 1.6 | 2.7 | 1.9 | 3.0 | 2.0 | 3.6 | 2.5 | 4.8 | 3.0 | 5.8 | 3.6 | 7.7 | 4.3 | 2.5 | 1.7 | 3.3 | 2.4 | 3.7 | 2.7 | 4.3 | 2.8 | 5.3 | 3.4 | 6.2 | 3.5 | 7.9 | 4.5 |
| 155 | Soft tissue disorders related to use, overuse and pressure | 2.2 | 1.4 | 2.7 | 1.8 | 3.2 | 2.3 | 3.9 | 2.9 | 5.5 | 3.7 | 7.1 | 4.0 | 9.3 | 4.4 | 2.6 | 1.7 | 3.9 | 2.6 | 4.2 | 2.7 | 4.9 | 3.2 | 6.4 | 4.0 | 7.6 | 4.1 | 8.6 | 4.1 |
| 156 | Fibroblastic disorders | 2.1 | 1.6 | 2.5 | 1.9 | 2.9 | 2.3 | 3.5 | 2.6 | 4.3 | 3.1 | 5.1 | 3.4 | 6.6 | 3.7 | 2.6 | 1.9 | 3.3 | 2.6 | 3.7 | 2.8 | 4.1 | 2.9 | 5.1 | 3.4 | 5.8 | 3.6 | 7.0 | 3.8 |
| 157 | Shoulder lesions | 2.0 | 1.3 | 2.5 | 1.8 | 3.0 | 2.2 | 3.7 | 2.7 | 4.6 | 3.1 | 5.9 | 3.6 | 7.6 | 4.0 | 2.4 | 1.7 | 3.4 | 2.5 | 3.8 | 2.7 | 4.2 | 2.9 | 5.1 | 3.3 | 6.6 | 3.9 | 8.4 | 4.1 |
| 158 | Enthesopathies of lower limb, excluding foot | 2.0 | 1.3 | 2.5 | 1.7 | 2.7 | 1.9 | 3.2 | 2.4 | 4.6 | 3.2 | 6.1 | 3.6 | 9.0 | 4.5 | 2.3 | 1.6 | 3.5 | 2.7 | 3.7 | 2.6 | 4.2 | 2.9 | 5.4 | 3.4 | 7.1 | 3.8 | 7.9 | 3.9 |
| 159 | Other enthesopathies | 2.2 | 1.5 | 2.7 | 1.8 | 3.1 | 2.1 | 3.8 | 2.6 | 5.0 | 3.3 | 6.6 | 3.7 | 8.2 | 4.0 | 2.5 | 1.7 | 3.7 | 2.5 | 3.9 | 2.5 | 4.4 | 2.8 | 5.7 | 3.4 | 7.3 | 4.1 | 8.2 | 4.4 |
| 160 | Rheumatism, unspecified | 2.9 | 2.4 | 3.4 | 2.5 | 4.6 | 3.1 | 5.4 | 3.4 | 7.1 | 4.3 | 7.6 | 4.6 | 9.5 | 4.4 | 3.9 | 2.6 | 5.3 | 3.3 | 5.6 | 3.5 | 6.4 | 3.8 | 7.3 | 4.1 | 8.7 | 4.5 | 10.6 | 4.6 |
| 161 | Myalgia | 2.4 | 1.7 | 3.2 | 2.6 | 3.9 | 2.8 | 4.7 | 3.5 | 6.1 | 4.1 | 7.7 | 4.6 | 9.8 | 4.7 | 3.3 | 2.3 | 4.4 | 3.2 | 4.9 | 3.2 | 5.7 | 3.6 | 6.7 | 4.0 | 8.0 | 4.9 | 9.5 | 4.6 |
| 162 | Other soft tissue disorders, not elsewhere classified | 2.3 | 1.5 | 2.7 | 1.9 | 3.6 | 2.7 | 4.7 | 3.3 | 6.1 | 4.1 | 7.8 | 4.7 | 9.8 | 4.8 | 3.0 | 2.3 | 3.5 | 2.4 | 4.5 | 3.0 | 5.4 | 3.5 | 6.8 | 4.1 | 7.9 | 4.5 | 9.9 | 5.2 |
| 163 | Other soft tissue disorders, not elsewhere classified: pain in limb | 2.2 | 1.6 | 2.8 | 2.1 | 3.5 | 2.6 | 4.3 | 3.0 | 5.5 | 3.8 | 7.2 | 4.2 | 9.3 | 4.9 | 2.5 | 1.8 | 4.0 | 2.9 | 4.3 | 3.0 | 5.1 | 3.5 | 6.3 | 3.9 | 7.6 | 4.3 | 8.8 | 4.3 |
| 164 | Fibromyalgia | 5.3 | 2.6 | 4.5 | 2.6 | 5.4 | 3.4 | 7.8 | 4.5 | 10.3 | 2.9 | 10.9 | 2.9 | 4.0 | 2.2 | 5.4 | 3.3 | 5.8 | 3.3 | 6.4 | 3.5 | 7.6 | 4.1 | 10.2 | 4.8 | 10.6 | 4.3 | n/a | n/a |
| 165 | Osteoporosis ^c^ | 5.3 | 2.7 | 5.3 | 2.7 | 5.3 | 3.2 | 5.9 | 3.7 | 6.4 | 3.9 | 7.3 | 4.2 | 8.4 | 4.2 | 5.5 | 2.6 | 6.8 | 3.9 | 6.3 | 4.0 | 5.2 | 3.6 | 5.1 | 3.4 | 5.7 | 3.6 | 7.2 | 3.8 |
| 166 | Osteoporosis in diseases classified elsewhere | 3.6 | 1.8 | 4.9 | 2.4 | 5.9 | 3.3 | 7.3 | 4.7 | 8.1 | 3.6 | 9.8 | 4.5 | 9.5 | 4.1 | 3.7 | 2.2 | 5.9 | 3.8 | 7.8 | 6.1 | 7.9 | 4.0 | 8.0 | 4.3 | 8.4 | 4.1 | 9.8 | 4.4 |
| 167 | Adult osteomalacia and other disorders of bone density and structure | 2.7 | 1.9 | 3.3 | 2.3 | 3.7 | 2.7 | 4.9 | 3.4 | 6.2 | 3.9 | 7.4 | 4.2 | 8.9 | 4.3 | 3.4 | 2.2 | 4.3 | 3.1 | 4.8 | 3.4 | 4.7 | 3.3 | 5.1 | 3.3 | 6.1 | 3.7 | 7.9 | 4.0 |
| 168 | Disorders of continuity of bone | 2.2 | 1.8 | 2.4 | 2.1 | 3.5 | 2.3 | 4.6 | 3.8 | 5.7 | 3.6 | 7.4 | 4.1 | 8.7 | 4.2 | 3.0 | 2.3 | 3.8 | 2.4 | 4.5 | 3.3 | 5.6 | 4.1 | 6.6 | 4.2 | 8.1 | 4.4 | 8.9 | 4.1 |
| 169 | Other osteopathies | 2.3 | 2.0 | 3.2 | 2.3 | 3.9 | 3.1 | 5.0 | 3.7 | 6.0 | 3.9 | 7.6 | 4.4 | 9.0 | 4.4 | 3.3 | 2.5 | 3.9 | 2.7 | 4.7 | 3.3 | 5.3 | 3.5 | 6.1 | 3.8 | 6.9 | 4.1 | 8.5 | 4.1 |
| 170 | Other disorders of the musculoskeletal system and connective tissue | 2.0 | 1.4 | 2.6 | 1.9 | 3.3 | 2.5 | 4.3 | 3.0 | 5.8 | 3.8 | 7.6 | 4.3 | 9.4 | 4.5 | 2.6 | 1.8 | 3.5 | 2.6 | 4.2 | 2.8 | 5.3 | 3.5 | 6.6 | 4.1 | 8.2 | 4.5 | 9.9 | 4.3 |
|  | **N – Diseases of the genitourinary system** | **3.4** | **2.5** | **4.5** | **2.9** | **5.7** | **3.4** | **7.1** | **3.9** | **8.2** | **4.3** | **9.2** | **4.5** | **9.8** | **4.3** | **4.0** | **2.8** | **5.2** | **3.4** | **6.7** | **4.2** | **7.7** | **4.4** | **8.6** | **4.5** | **9.4** | **4.4** | **10.2** | **4.3** |
| 171 | Chronic renal failure (CRF) ^c^ | 3.4 | 2.5 | 4.5 | 2.9 | 5.7 | 3.4 | 7.1 | 3.9 | 8.2 | 4.3 | 9.2 | 4.5 | 9.8 | 4.3 | 4.0 | 2.8 | 5.2 | 3.4 | 6.7 | 4.2 | 7.7 | 4.4 | 8.6 | 4.5 | 9.4 | 4.4 | 10.2 | 4.3 |
|  | **Q – Congenital malformations, deformations and chromosomal abnormalities** | 2.2 | 1.7 | 2.5 | 2.0 | 3.0 | 2.3 | 3.8 | 2.8 | 5.1 | 3.4 | 6.3 | 3.9 | 8.0 | 4.2 | 2.4 | 1.9 | 2.9 | 2.2 | 3.3 | 2.5 | 4.2 | 3.0 | 5.5 | 3.6 | 6.7 | 4.0 | 8.4 | 4.2 |
| 172 | Congenital malformations: of the nervous, circulatory and respiratory systems, cleft palate and cleft lip, urinary tract, bones and muscles, other and chromosomal abnormalities not elsewhere classified | 2.3 | 1.8 | 2.7 | 2.0 | 3.3 | 2.5 | 4.2 | 2.9 | 5.5 | 3.5 | 6.7 | 4.0 | 8.4 | 4.3 | 2.6 | 1.9 | 3.0 | 2.2 | 3.4 | 2.5 | 4.4 | 3.1 | 5.7 | 3.6 | 6.9 | 4.0 | 8.4 | 4.1 |
| 173 | Congenital malformations of eye, ear, face and neck | 1.9 | 1.4 | 2.3 | 1.8 | 2.7 | 2.1 | 3.3 | 2.3 | 4.5 | 3.1 | 5.6 | 3.6 | 7.6 | 4.0 | 2.1 | 1.7 | 2.7 | 2.0 | 3.0 | 2.2 | 3.8 | 2.8 | 4.9 | 3.3 | 6.1 | 3.8 | 7.8 | 4.2 |
| 174 | Other congenital malformations of the digestive system | 2.9 | 2.2 | 3.1 | 2.4 | 3.6 | 2.9 | 4.5 | 3.4 | 5.6 | 3.6 | 6.8 | 4.4 | 8.4 | 4.2 | 3.1 | 2.5 | 3.6 | 2.9 | 4.1 | 2.9 | 5.2 | 3.7 | 6.3 | 4.1 | 7.8 | 4.5 | 8.9 | 4.5 |
| 175 | Congenital malformations of the sexual organs | 2.2 | 1.8 | 2.3 | 1.9 | 2.6 | 2.1 | 3.4 | 2.8 | 4.4 | 3.2 | 5.5 | 3.4 | 7.2 | 3.9 | 2.6 | 2.0 | 2.7 | 2.0 | 3.0 | 2.4 | 3.6 | 2.7 | 4.4 | 3.1 | 5.0 | 2.9 | 7.1 | 3.8 |
|  | **F – Mental and behavioural disorders** | **2.3** | **1.6** | **3.0** | **2.1** | **3.5** | **2.5** | **4.2** | **2.9** | **5.3** | **3.4** | **6.7** | **3.9** | **7.8** | **4.0** | **2.7** | **2.0** | **3.4** | **2.4** | **3.8** | **2.6** | **4.5** | **3.0** | **5.4** | **3.5** | **6.6** | **3.8** | **7.5** | **3.8** |
| 176 | Dementia ^c^ | 5.6 | 1.9 | 6.1 | 3.3 | 6.1 | 3.9 | 5.7 | 2.9 | 6.8 | 3.9 | 7.5 | 4.0 | 7.5 | 3.8 | n/a | n/a | n/a | n/a | 7.4 | 3.9 | 6.2 | 3.9 | 6.9 | 4.4 | 7.6 | 4.2 | 7.3 | 3.7 |
| 177 | Organic, including symptomatic, mental disorders | 4.8 | 2.8 | 5.5 | 3.4 | 6.0 | 3.6 | 6.5 | 3.9 | 7.6 | 4.3 | 8.7 | 4.4 | 9.3 | 4.2 | 5.9 | 3.7 | 6.6 | 4.2 | 6.6 | 4.3 | 7.5 | 4.3 | 8.2 | 4.6 | 9.1 | 4.5 | 9.1 | 4.1 |
| 178 | Mental and behavioural disorders due to use of alcohol | 2.6 | 2.0 | 4.0 | 2.8 | 5.0 | 3.1 | 5.5 | 3.5 | 6.4 | 3.8 | 7.4 | 4.1 | 8.4 | 4.3 | 3.4 | 2.6 | 5.3 | 3.5 | 6.1 | 3.7 | 6.4 | 3.8 | 7.4 | 4.1 | 8.0 | 4.3 | 8.8 | 4.1 |
| 179 | Mental and behavioural disorders due to psychoactive substance use | 3.4 | 2.1 | 4.3 | 2.7 | 5.2 | 3.3 | 5.8 | 3.7 | 6.9 | 4.0 | 7.7 | 4.4 | 8.8 | 4.5 | 3.9 | 2.6 | 4.6 | 3.4 | 5.1 | 3.7 | 6.1 | 4.0 | 7.5 | 4.5 | 8.6 | 4.8 | 9.3 | 4.4 |
| 180 | Schizophrenia ^c^ | 4.3 | 2.1 | 4.8 | 2.7 | 5.2 | 3.1 | 5.4 | 3.4 | 5.9 | 4.0 | 6.6 | 4.1 | 6.9 | 4.0 | 5.6 | 3.2 | 6.7 | 3.7 | 6.6 | 4.0 | 6.6 | 4.2 | 7.0 | 4.5 | 7.1 | 4.3 | 7.4 | 4.2 |
| 181 | Schizotypal and delusional disorders | 4.2 | 2.2 | 4.8 | 2.7 | 5.2 | 3.1 | 5.8 | 3.5 | 6.6 | 4.1 | 7.3 | 4.4 | 8.2 | 4.3 | 5.2 | 3.0 | 6.1 | 3.5 | 6.1 | 3.8 | 6.5 | 4.0 | 7.1 | 4.3 | 7.5 | 4.3 | 8.2 | 4.1 |
| 182 | Bipolar affective disorder ^c^ | 4.2 | 2.4 | 5.1 | 2.8 | 5.5 | 3.2 | 6.1 | 3.6 | 6.8 | 4.0 | 7.7 | 4.4 | 8.9 | 4.6 | 5.0 | 3.0 | 5.9 | 3.3 | 6.3 | 3.5 | 6.8 | 3.9 | 7.5 | 4.2 | 8.4 | 4.6 | 9.0 | 4.1 |
| 183 | Depression ^c^ | 2.9 | 2.0 | 3.2 | 2.3 | 3.6 | 2.6 | 4.4 | 3.0 | 5.5 | 3.5 | 7.0 | 4.0 | 8.3 | 4.1 | 3.0 | 2.1 | 3.6 | 2.6 | 3.9 | 2.7 | 4.6 | 3.1 | 5.5 | 3.5 | 6.7 | 3.9 | 7.7 | 3.8 |
| 184 | Mood (affective) disorders | 4.5 | 2.5 | 5.0 | 2.8 | 5.9 | 3.5 | 6.9 | 4.0 | 7.7 | 4.3 | 8.7 | 4.4 | 10.3 | 4.7 | 5.1 | 3.2 | 6.2 | 3.7 | 6.5 | 3.8 | 7.3 | 4.2 | 8.5 | 4.6 | 9.1 | 4.8 | 9.6 | 4.3 |
| 185 | Phobic anxiety disorders | 3.6 | 2.0 | 4.3 | 2.4 | 5.2 | 3.1 | 6.0 | 3.6 | 7.0 | 4.3 | 7.9 | 3.9 | 8.9 | 5.1 | 4.2 | 2.3 | 4.9 | 3.0 | 4.9 | 3.1 | 6.1 | 3.6 | 7.2 | 3.9 | 8.6 | 4.4 | 9.5 | 4.3 |
| 186 | Other anxiety disorders | 3.9 | 2.3 | 4.6 | 2.8 | 5.3 | 3.2 | 6.3 | 3.8 | 7.5 | 4.4 | 8.9 | 4.7 | 10.4 | 4.9 | 4.3 | 2.6 | 5.0 | 3.1 | 5.4 | 3.3 | 6.4 | 3.8 | 7.5 | 4.2 | 8.9 | 4.6 | 9.8 | 4.3 |
| 187 | Obsessive compulsive disorder (OCD) ^c^ | 3.5 | 2.2 | 4.3 | 2.8 | 4.9 | 2.8 | 5.7 | 3.6 | 7.0 | 4.4 | 7.4 | 4.2 | 9.2 | 5.4 | 4.0 | 2.6 | 4.8 | 2.9 | 5.4 | 3.3 | 6.1 | 3.8 | 7.1 | 4.1 | 8.0 | 4.7 | 8.6 | 3.4 |
| 188 | Post-traumatic stress disorder | 3.6 | 2.4 | 3.9 | 2.6 | 4.4 | 2.7 | 5.2 | 3.2 | 5.8 | 3.5 | 6.9 | 4.2 | 9.3 | 5.4 | 4.7 | 2.9 | 5.1 | 3.4 | 5.1 | 3.1 | 5.7 | 3.4 | 6.2 | 3.7 | 6.9 | 4.1 | 9.6 | 4.7 |
| 189 | Reactions to severe stress and adjustment disorders | 3.3 | 2.1 | 4.1 | 2.6 | 4.8 | 3.1 | 5.5 | 3.5 | 6.8 | 4.3 | 8.3 | 4.7 | 1n/a | 5.0 | 3.7 | 2.4 | 4.7 | 3.0 | 5.1 | 3.3 | 5.8 | 3.7 | 6.6 | 4.1 | 8.5 | 4.6 | 9.5 | 4.4 |
| 190 | Dissociative (conversion) disorders, somatoform disorders and other neurotic disorders | 3.9 | 2.5 | 4.5 | 3.1 | 5.1 | 3.5 | 6.1 | 4.0 | 6.9 | 4.3 | 8.3 | 4.8 | 1n/a | 5.2 | 4.4 | 3.1 | 5.4 | 3.6 | 5.7 | 3.7 | 6.5 | 4.1 | 7.6 | 4.5 | 8.7 | 5.2 | 9.8 | 4.7 |
| 191 | Eating disorders | 3.5 | 2.1 | 5.4 | 4.0 | 6.0 | 3.1 | 6.0 | 3.6 | 9.9 | 5.5 | 10.3 | 3.8 | 10.1 | 5.2 | 3.3 | 2.4 | 4.7 | 3.1 | 5.6 | 3.5 | 7.0 | 4.0 | 9.8 | 5.1 | 9.6 | 5.4 | 9.6 | 4.6 |
| 192 | Behavioural syndromes associated with physiological disturbances and physical factors | 3.3 | 2.3 | 3.1 | 2.5 | 3.5 | 3.0 | 5.0 | 3.7 | 5.9 | 4.0 | 6.4 | 3.8 | 8.9 | 3.8 | 3.5 | 2.6 | 3.8 | 2.9 | 4.4 | 3.3 | 5.4 | 4.0 | 6.5 | 4.3 | 8.0 | 5.1 | 1n/a | 4.5 |
| 193 | Emotionally unstable personality disorder | 4.7 | 2.5 | 5.3 | 3.2 | 6.0 | 3.7 | 6.8 | 4.1 | 8.1 | 4.9 | 8.3 | 4.4 | 12.2 | 6.4 | 5.2 | 2.7 | 5.9 | 3.3 | 6.4 | 3.8 | 7.4 | 4.3 | 8.4 | 4.7 | 9.2 | 4.9 | 1n/a | 4.6 |
| 194 | Specific personality disorders | 4.2 | 2.3 | 4.5 | 2.8 | 5.0 | 3.2 | 5.8 | 3.7 | 6.7 | 4.4 | 7.9 | 4.6 | 8.8 | 4.5 | 4.9 | 2.7 | 5.3 | 3.1 | 5.6 | 3.5 | 6.5 | 3.9 | 7.4 | 4.3 | 8.6 | 4.7 | 9.4 | 4.5 |
| 195 | Disorders of adult personality and behaviour | 4.2 | 2.4 | 5.1 | 3.1 | 5.3 | 3.3 | 5.9 | 3.7 | 6.8 | 4.4 | 8.0 | 4.6 | 10.1 | 5.2 | 5.1 | 3.0 | 6.0 | 3.3 | 6.3 | 3.8 | 6.9 | 4.1 | 7.8 | 4.4 | 9.1 | 5.1 | 9.6 | 4.6 |
| 196 | Mental retardation | 3.8 | 2.2 | 4.5 | 2.7 | 5.0 | 3.0 | 5.5 | 3.1 | 5.7 | 3.2 | 6.4 | 3.5 | 6.0 | 3.2 | 4.5 | 2.8 | 5.3 | 3.4 | 5.6 | 3.8 | 5.9 | 3.7 | 6.3 | 3.7 | 7.4 | 4.0 | 7.0 | 3.8 |
| 197 | Disorders of psychological development | 3.2 | 1.9 | 4.0 | 2.6 | 4.9 | 2.9 | 5.6 | 3.2 | 5.9 | 3.4 | 7.0 | 3.8 | 7.7 | 4.1 | 4.0 | 2.5 | 5.5 | 3.4 | 5.6 | 3.9 | 6.1 | 3.1 | 7.0 | 4.2 | 7.8 | 3.5 | 9.0 | 4.3 |
| 198 | Hyperkinetic disorders (ADHD) ^c^ | 2.3 | 1.7 | 3.7 | 2.5 | 4.5 | 2.9 | 4.9 | 3.3 | 6.1 | 3.8 | 7.2 | 4.3 | 9.2 | 4.5 | 3.2 | 2.3 | 4.9 | 3.0 | 5.2 | 3.1 | 5.8 | 3.5 | 6.7 | 3.8 | 8.0 | 4.4 | 8.6 | 4.2 |
| 199 | Behavioural and emotional disorders with onset usually occurring in childhood and adolescence | 3.5 | 2.1 | 4.5 | 2.8 | 5.5 | 3.4 | 6.2 | 3.7 | 7.3 | 4.4 | 7.9 | 4.4 | 9.0 | 4.7 | 4.4 | 2.7 | 5.5 | 3.4 | 6.1 | 3.8 | 7.0 | 4.1 | 7.8 | 4.6 | 8.8 | 4.6 | 9.4 | 4.4 |
|  |  |  |  |  |  |  |  |  |  |  |  |  |  |  |  |  |  |  |  |  |  |  |  |  |  |  |  |  |  |
|  | **Having one or more chronic conditions** | **1.7** | **1.2** | **2.0** | **1.5** | **2.3** | **1.8** | **2.7** | **2.2** | **3.4** | **2.6** | **4.2** | **3.0** | **5.6** | **3.5** | **1.9** | **1.5** | **2.3** | **1.8** | **2.5** | **2.0** | **3.0** | **2.3** | **3.6** | **2.7** | **4.3** | **3.1** | **5.6** | **3.5** |
|  |  |  |  |  |  |  |  |  |  |  |  |  |  |  |  |  |  |  |  |  |  |  |  |  |  |  |  |  |  |
|  | Depression medicine ^c^ ** | 2.3 | 2.0 | 2.7 | 2.3 | 3.2 | 2.6 | 4.0 | 3.1 | 5.2 | 3.5 | 6.7 | 4.0 | 7.9 | 4.1 | 2.5 | 2.2 | 3.2 | 2.6 | 3.6 | 2.8 | 4.3 | 3.1 | 5.3 | 3.5 | 6.4 | 3.9 | 7.4 | 3.8 |
|  | Antipsychotic medicine ^c^ ** | 2.8 | 2.2 | 3.9 | 2.7 | 4.5 | 3.0 | 5.0 | 3.4 | 5.7 | 3.8 | 6.7 | 4.2 | 7.5 | 4.2 | 4.0 | 2.8 | 5.1 | 3.3 | 5.2 | 3.4 | 5.7 | 3.7 | 6.3 | 4.0 | 6.8 | 4.2 | 7.3 | 3.9 |
|  | Indication prescribed anxiety medicine ^c^ ** | 2.5 | 2.2 | 3.1 | 2.6 | 3.6 | 3.0 | 4.3 | 3.4 | 5.5 | 4.0 | 6.8 | 4.2 | 8.1 | 4.4 | 2.9 | 2.5 | 3.7 | 3.0 | 3.9 | 3.1 | 4.5 | 3.5 | 5.4 | 3.8 | 6.5 | 4.3 | 7.5 | 4.0 |
|  | Heart failure medication ^c^ ** | 4.3 | 2.8 | 4.9 | 2.4 | 5.8 | 3.1 | 6.4 | 3.5 | 7.2 | 3.7 | 7.9 | 3.8 | 9.0 | 4.0 | 2.5 | 1.5 | 5.1 | 3.1 | 7.2 | 4.7 | 6.8 | 3.6 | 7.2 | 4.0 | 8.4 | 4.4 | 9.0 | 4.2 |
|  | Ischaemic heart medication ^c^ ** | 3.2 | 2.7 | 3.6 | 3.4 | 4.8 | 3.5 | 5.7 | 3.6 | 6.4 | 3.7 | 7.2 | 3.9 | 8.0 | 4.0 | 3.5 | 3.3 | 4.2 | 4.0 | 5.2 | 4.0 | 6.1 | 4.0 | 6.9 | 4.2 | 7.6 | 4.1 | 8.0 | 4.0 |
|  | All five types of the medicine above | 2.3 | 2.0 | 2.8 | 2.4 | 3.3 | 2.7 | 4.1 | 3.1 | 5.3 | 3.5 | 6.6 | 3.8 | 7.6 | 3.9 | 2.6 | 2.2 | 3.3 | 2.7 | 3.6 | 2.8 | 4.3 | 3.1 | 5.3 | 3.5 | 6.4 | 3.8 | 7.3 | 3.8 |
|  |  |  |  |  |  |  |  |  |  |  |  |  |  |  |  |  |  |  |  |  |  |  |  |  |  |  |  |  |  |
|  | **Total population** | **0.5** | **1.0** | **0.9** | **1.4** | **1.2** | **1.7** | **1.7** | **2.2** | **2.6** | **2.7** | **3.7** | **3.2** | **5.3** | **3.7** | **0.7** | **1.3** | **1.3** | **1.8** | **1.7** | **2.0** | **2.2** | **2.4** | **3.0** | **2.8** | **3.8** | **3.2** | **5.4** | **3.6** |
|  |  |  |  |  |  |  |  |  |  |  |  |  |  |  |  |  |  |  |  |  |  |  |  |  |  |  |  |  |  |
|  | **Extra** |  |  |  |  |  |  |  |  |  |  |  |  |  |  |  |  |  |  |  |  |  |  |  |  |  |  |  |  |
|  | Ischaemic Heart Diseases broad | 2.7 | 2.0 | 3.2 | 2.5 | 4.1 | 3.0 | 5.1 | 3.1 | 6.0 | 3.3 | 6.9 | 3.6 | 8.0 | 3.7 | 3.2 | 2.4 | 4.0 | 3.1 | 4.6 | 3.3 | 5.6 | 3.6 | 6.5 | 3.8 | 7.3 | 3.9 | 8.2 | 3.8 |
|  | Artritis | 2.4 | 1.7 | 2.8 | 2.0 | 3.1 | 2.3 | 3.7 | 2.7 | 4.6 | 3.1 | 5.7 | 3.6 | 7.2 | 3.9 | 2.7 | 2.0 | 3.5 | 2.6 | 3.9 | 2.7 | 4.5 | 3.0 | 5.2 | 3.3 | 5.9 | 3.6 | 7.3 | 3.8 |
|  | Arthrosis | 2.4 | 1.7 | 2.8 | 2.1 | 3.1 | 2.3 | 3.7 | 2.7 | 4.6 | 3.1 | 5.7 | 3.6 | 7.1 | 3.9 | 3.0 | 2.0 | 3.9 | 2.7 | 4.1 | 2.9 | 4.6 | 3.1 | 5.3 | 3.4 | 6.0 | 3.6 | 7.3 | 3.8 |
|  | Backconditions | 2.3 | 1.7 | 2.8 | 2.0 | 3.3 | 2.4 | 4.0 | 2.8 | 5.3 | 3.4 | 7.0 | 4.0 | 8.6 | 4.2 | 2.5 | 2.0 | 3.6 | 2.6 | 4.1 | 2.8 | 4.9 | 3.2 | 6.2 | 3.7 | 7.5 | 4.1 | 8.9 | 4.1 |
|  | Overweight, clinical (BMI >35) | 1.8 | 2.1 | 2.7 | 2.7 | 3.3 | 3.0 | 4.4 | 3.3 | 5.9 | 3.8 | 7.1 | 4.1 | 8.7 | 4.5 | 1.9 | 2.3 | 1.9 | 2.2 | 2.4 | 2.6 | 4.0 | 3.3 | 5.5 | 3.8 | 6.4 | 4.0 | 7.9 | 4.2 |
|  | Endometriosis | n/a | n/a | n/a | n/a | n/a | n/a | n/a | n/a | n/a | n/a | n/a | n/a | n/a | n/a | 1.9 | 2.2 | 2.1 | 2.3 | 2.3 | 2.4 | 3.0 | 2.9 | 3.7 | 3.1 | 4.4 | 3.3 | 7.0 | 3.9 |

Gender and age-standardised estimates (Std.) are in brackets.

ICD-10 International Statistical Classification of Diseases, 10^th^ Revision.

^c^ = complex defined conditions, see reference for further details [64].

Conditions marked ‘A’, overlap with other conditions and are thus not counted twice [64].

** 2-year prevalence. n/a: not available.
